# Mathematical Modeling and Validation of the Ergosterol Pathway in *Saccharomyces cerevisiae*

**DOI:** 10.1371/journal.pone.0028344

**Published:** 2011-12-14

**Authors:** Fernando Alvarez-Vasquez, Howard Riezman, Yusuf A. Hannun, Eberhard O. Voit

**Affiliations:** 1 Department of Biochemistry and Molecular Biology, Medical University of South Carolina, Charleston, South Carolina, United States of America; 2 Department of Biochemistry, University of Geneva, Geneva, Switzerland; 3 Wallace H. Coulter Department of Biomedical Engineering, Georgia Institute of Technology, Atlanta, Georgia, United States of America; 4 Department of Veterinary Integrative Biosciences, College of Veterinary Medicine and Biomedical Sciences, Texas A&M University, College Station, Texas, United States of America; Nanjing Agricultural University, China

## Abstract

The *de novo* biosynthetic machinery for both sphingolipid and ergosterol production in yeast is localized in the endoplasmic reticulum (ER) and Golgi. The interconnections between the two pathways are still poorly understood, but they may be connected in specialized membrane domains, and specific knockouts strongly suggest that both routes have different layers of mutual control and are co-affected by drugs. With the goal of shedding light on the functional integration of the yeast sphingolipid-ergosterol (SL-E) pathway, we constructed a dynamic model of the ergosterol pathway using the guidelines of Biochemical Systems Theory (BST) (Savageau., *J. theor. Biol.*, **25**, 365–9, 1969). The resulting model was merged with a previous mathematical model of sphingolipid metabolism in yeast (Alvarez-Vasquez *et al*., *J. theor. Biol.*, **226**, 265–91, 2004; Alvarez-Vasquez *et al*., *Nature* **433**, 425–30, 2005). The S-system format within BST was used for analyses of consistency, stability, and sensitivity of the SL-E model, while the GMA format was used for dynamic simulations and predictions. Model validation was accomplished by comparing predictions from the model with published results on sterol and sterol-ester dynamics in yeast. The validated model was used to predict the metabolomic dynamics of the SL-E pathway after drug treatment. Specifically, we simulated the action of drugs affecting sphingolipids in the endoplasmic reticulum and studied changes in ergosterol associated with microdomains of the plasma membrane (PM).

## Introduction

Sphingolipids and sterols constitute two of the major classes of eukaryotic lipids. Although, for the most part, these two classes can be studied metabolically and functionally in isolation, there is a growing body of evidence to suggest more intimate connections between the two. In particular, the two pathways are subject to cross- and co-regulation under many conditions [1], [2]. Moreover, interactions between the two classes have been implicated in the action and resistance to many anti-fungal agents. Indeed, of particular therapeutic interest among the new classes of drugs are those that affect the biosynthesis of ergosterol and complex sphingolipids (CS) and those that have been proposed to require the formation of sphingolipid/ergosterol microdomains that constitute anchors for fundamental homeostatic fungal proteins like Pma1p [3], [4].

Because the ergosterol and sphingolipid pathways constitute complex systems, it is difficult to predict with intuition alone the effects of changes in one pathway on the operation of the other; moreover, it is difficult to predict which specific steps could provide most efficacious drug targets and what the sequelae of alterations in particular enzyme activities could be. To achieve sufficient understanding of the dynamic interactions between the two biosynthetic pathways and their regulatory effects on the formation and destruction of putative microdomains, it seems necessary to work toward reliable mathematical and computational systems models. Such models may eventually help us appreciate differences in the structure and function of membrane microdomains of normal or cancer cells, and other pathological conditions [5]–[7]. The models may also aid in interpreting the dynamics of microdomains, which are presently investigated mainly through visualization by optical microscopy with molecular markers [8]. Furthermore, dynamic simulations of microdomains may provide rational clues about drug-membrane interactions and aid the development of therapeutic agents [9].

It should be noted that the existence of biological microdomains in cells has not been firmly established, in contrast to the physical demonstration of microdomains in experimental models of membranes. In yeast, the plasma membrane has at least three different domains, one containing a variety of solute transporters, another with the plasma membrane H^+^-ATPase [10] and still another with associated TOR complex 2 [11]. Studies using filipin staining suggest that some plasma membrane (PM) microdomains may be enriched in ergosterol [12], and since sterols and sphingolipids can form complexes *in vitro*, perhaps these domains are also enriched in complex sphingolipids, and microdomain-associated proteins, such as solute transporters, Pma1p, and probably several glycosylphosphatidylinositols (GPI's)-linked proteins [13]–[15]. A possible regulator of the PM localization and stability of Pma1 is the presence of very long chain fatty acids associated with detergent-insoluble membranes (DIM). This was recently demonstrated in yeast mutants unable to produce complex sphingolipids but able to constitute functional Pma1p DIM microdomains in association with alternative compensatory very-long chains glycerolipids [16].

Any metabolic model of microdomain dynamics requires better insights into the biosynthesis of sphingolipids and sterols. The biosynthetic sterol route in fungi and vertebrates has been described in the literature with different names, including the mevalonate pathway [17], [18], the isoprenoid route [19], or the sterol biosynthetic pathway [20]. Throughout this paper, we simply refer to this route as the “ergosterol pathway.” It is well known that the ergosterol pathway is closely connected with sphingolipid metabolism, which has important structural and signaling functions in yeast and higher organisms (*e.g.*, [1], [2], [21]–[28]). The aim of our study is the elucidation of the combined “sphingolipid-ergosterol” (SL-E) system with a dynamic mathematical model.

**Figure 1:**
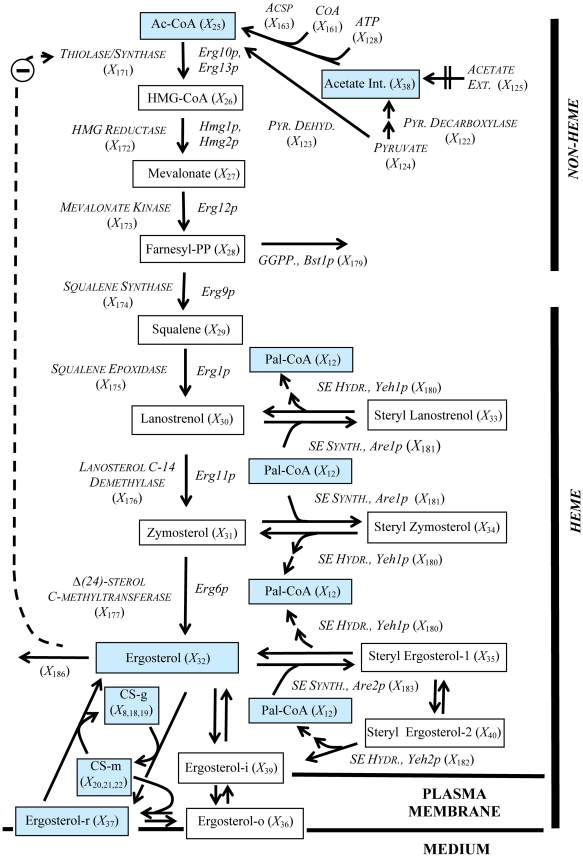
Ergosterol model for yeast. Solid boxes represent time dependent variables, italics represent time independent variables, consecutive arrows represent multiple enzymatic reactions taking place, and the dashed arrow indicates an inhibitory feedback signal. Blue boxes represent metabolites in common with the sphingolipid pathway from Fig. 2. Abbreviations for the time dependent variables: Palmitoyl-CoA (Pal-CoA), *X_12_*; Acetyl-CoA (Ac-CoA), *X_25_*; 3-hydroxy-3-methylglutaryl-coenzyme A (HMG-CoA), *X_26_*; Mevalonate,*X_27_*; Farnesyl pyrophosphate (Farnesyl-PP), *X_28_*; Squalene,*X_29_*; Lanosterol,*X_30_*; Zymosterol,*X_31_*; Endoplasmic reticulum ergosterol (Ergosterol), *X_32_*; Steryl Lanosterol, *X_33_*; Steryl Zymosterol, *X_34_*; Steryl Ergosterol in Bulk Lipid Particles (Steryl Ergosterol-1), *X*_35_; Outer Leaflet Ergosterol in the Plasma Membrane (Ergosterol-o), *X*_36_; Detergent insoluble plasma associated ergosterol (Ergosterol-r), *X*_37_; Internal Acetate (Acetate Int.), *X*_38_; Inner Leaflet Ergosterol in the Plasma Membrane (Ergosterol-i), *X*_39_; Steryl Ergosterol in Plasma Membrane Associated Lipid Particles (Steryl Ergosterol-2), *X*_40_; Golgi Associated Complex Sphingolipids (CS-g), *X*_8_+*X*_18_+*X*_19_; Plasma Membrane Associated Complex Sphingolipids (CS-m), *X*_20_+*X*_21_+*X*_22_.

The ergosterol pathway is readily divided into two functional parts, namely the heme-dependent and the heme-independent sections (Fig. 1). The heme-independent route yields squalene, while the heme-dependent route results in metabolites from lanosterol to ergosterol [19], [20]. The heme-dependent section utilizes molecular oxygen as co-substrate for the epoxidation of squalene by Erg1p and cytochrome hemoproteins, which are involved in the key demethylation and desaturation reactions [20], [29]. Interestingly the sphingolipid hydroxylation through the Sur2p also uses molecular oxygen, a shared feature with ergosterol biosynthesis [30].

Cholesterol and ergosterol are the final products of the sterol biosynthetic routes for mammalian and yeast cells, respectively. These sterol end products are essential components of lipid membranes, and their production is tightly regulated at multiple cellular levels [31]. Indeed, ergosterol is the main sterol component in the yeast plasma membrane, followed by other sterols such as zymosterol, fecosterol, and episterol, which are present in lower concentrations [32], [33]. The importance of sterols for yeast survival is evidenced by the large number of antifungal compounds affecting this route [34], [35]. Also, *ERG11* knockouts and strains with a non-functional Erg11p enzyme are non-viable unless exogenous ergosterol is supplied in the medium [36]. By contrast, strains with mutations occurring after the Erg6p step are viable, as the sterol intermediates can migrate to the PM and supplant the essential ergosterol functions; however, these mutants display alterations in the sensitivity to drugs and stress situations [1], [36], [37].

In this work, we construct and validate a mathematical model of the biosynthetic ergosterol pathway in *Saccharomyces cerevisiae* and integrate it with an existing sphingolipid-glycerolipid model that was formulated with methods of Biochemical Systems Theory (BST) [38], [39]. The combined, validated SL-E model is used to predict the metabolite dynamics of the system when key enzymes of the pathway are affected by drugs.

A mathematical representation of the heme-dependent section of the ergosterol pathway had been established previously with methods of Metabolic Flux Analysis [40]; however, this approach did not consider the heme-independent section, sub-pools of ergosterol, or feedback regulations within the pathway. Radioactive tracer experiments have suggested that these features are important for the sterol and sterol-ester dynamics [41], [42]. We therefore revisit the flux model and reformulate it to include these features.

## Results

**Figure 2:**
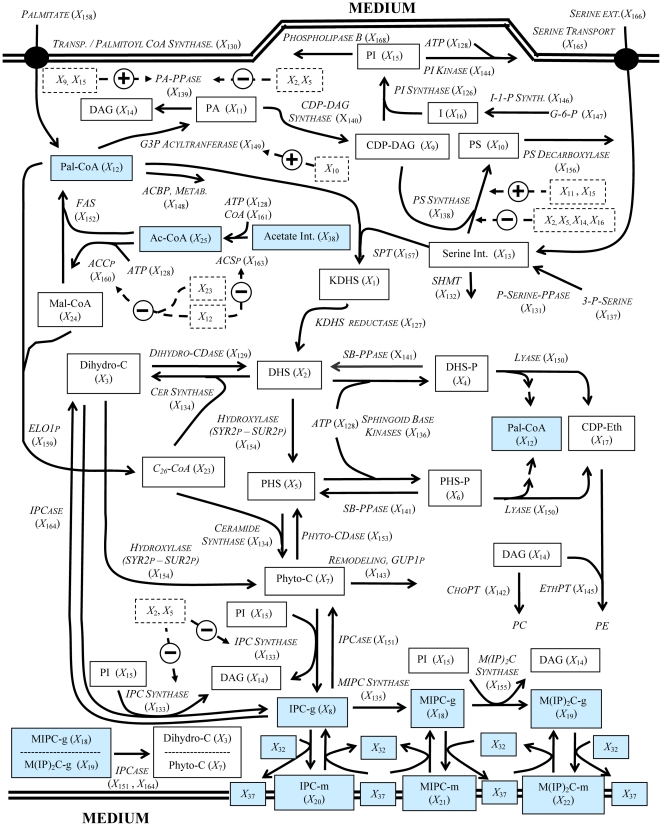
Sphingolipid model for yeast. Solid boxes represent time dependent variables, italics represent variables assumed to be time independent, dashed boxes represent variables with inhibitory or activating effects, dashed arrows represent more than one enzymatic reaction taking place. Blue boxes represent metabolites in common with the ergosterol pathway from Fig. 1. Abbreviations for the time dependent variables: 3-Keto-Sphinganine (KDHS), *X_1_*; Sphinganine (DHS), *X_2_*; Dihydroceramide (Dihydro-C), *X_3_*; Sphinganine-1P (DHS-P), *X_4_*; 4-OH-Sphinganine (PHS), *X_5_*; Phytosphingosine-1P (PHS-P), *X_6_*; Phytoceramide (Phyto-C), *X_7_*; Inositol Phosphorylceramide (IPC-g), *X_8_*; CDP-Diacylglycerol (CDP-DAG), *X_9_*; Phosphatidylserine (PS), *X_10_*; Phosphatidic Acid (PA), *X_11_*; Palmitoyl-CoA (Pal-CoA), *X_12_*; Serine, *X_13_*; *sn*-1,2-Diacylglycerol (DAG), *X_14_*; Phosphatidylinositol (PI), *X_15_*; Inositol (I), *X_16_*; Cytidine Diphosphate-Ethanolamine (CDP-Eth), *X_17_*; Mannosylinositol Phosphorylceramide (MIPC-g), *X_18_*; Mannosyldiinositol Phosphorylceramide (M(IP)_2_C-g), *X_19_*; Inositol Phosphorylceramide in Plasma Membrane (IPC-m), *X_20_*; Mannosylinositol Phosphorylceramide in Plasma Membrane (MIPC-m), *X_21_*; Mannosyldinositol Phosphorylceramide in Plasma Membrane (M(IP)_2_C-m), *X_22_*; Very Long Chain Fatty Acid (C_26_-CoA), *X_23_*; Malonyl-CoA (Mal-CoA), *X_24_*; Acetyl-CoA (Ac-CoA), *X_25_*; Endoplasmic reticulum ergosterol (Ergosterol), *X_32_*; Plasma Associated, Detergent Insoluble ergosterol (Ergosterol-r), *X*_37_; Internal Acetate (Acetate Int.), *X*_38_.

Sterol biosynthesis is localized in the ER [29] and the sterol and sphingolipid pathways share acetyl-CoA as a building block for fatty acyl and mevalonate formation, respectively (Figs. 1 and 2). It is therefore clear where the ergosterol pathway and the earlier sphingolipid model overlap and how they have to be integrated in a mathematical model. We used for this integration purpose the modeling framework of Biochemical Systems Theory [43]–[46], which allows a maximal degree of flexibility, while requiring a minimum of assumptions. The details of model design, assumptions, diagnostics, and analysis are presented in the *Methods* section and *Tables S1, S2, S3, S4, Materials S1 and S2, Equations S1, S2, S3, S4, S5, S6*.

### Steryl-ester dynamics after perturbations in external acetate

**Figure 3:**
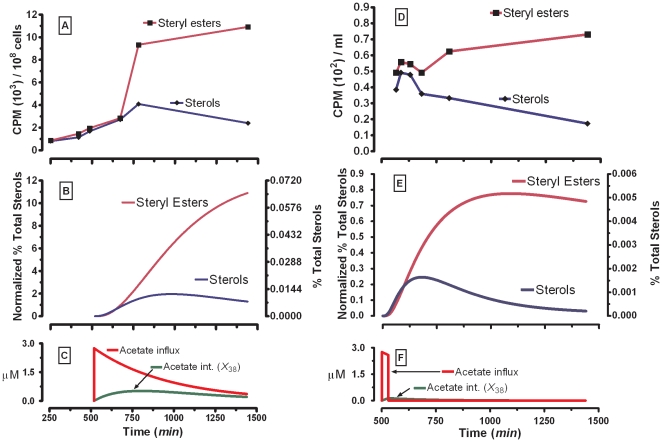
Experimental data *vs.* SL-E model simulation results for steryl-esters and total sterols. After an external pulse bolus (Panels *A–C*). (***A***) Dynamics adapted from Taylor and Parks [42] after pulse-chase bolus of [^14^ *C*]-Acetate. (***B***) Simulation for total steryl-esters (*X*_33_+*X*_34_+*X*_35_+*X*_40_) and total sterols (*X*_30_+*X*_31_+*X*_32_+*X*_36_+*X*_37_+*X*_39_) after pulse bolus of labeled acetate. (***C***) Transported external label acetate (*X*_125_) and cytoplasmic acetate (*X*_38_). The initial label of external acetate was 125 µM. After 30 min pulse-chase with external [^14^ *C*]-acetate (Panels *D*–*F*). (***D***) Dynamics adapted from Taylor and Parks [42] after pulse-chase bolus of [^14^ *C*]-Acetate. (***E***) Model simulation for steryl-esters (*X*_33_+*X*_34_+*X*_35_+*X*_40_) and total sterols (*X*_30_+*X*_31_+*X*_32_+*X*_36_+*X*_37_+*X*_39_) after pulse-chase bolus of labeled acetate. (***F***) Transported external label acetate (*X*_125_) and cytoplasmic acetate (*X*_38_). The initial label of external acetate was 125 µM. The % Total Sterols was calculated as percent with respect to the total sterol amount for the *S. cerevisiae* wild type strain. In Fig. 3B and E, the “Normalized % Total Sterols” were normalized against the last experimental values for the steryl-esters from Figs. A and D.

**Figure 4:**
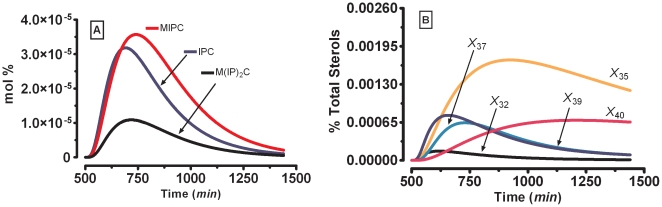
SL-E model results for an external pulse-chase bolus similar to the one in **Fig. 3F**. (***A***) **Complex sphingolipids**: Golgi compartment plus plasma membrane IPC (*X*_8_+*X*_20_), Golgi compartment plus plasma membrane MIPC (*X*_18_+*X*_21_), Golgi compartment plus plasma membrane M(IP)_2_C (*X*_19_+*X*_22_). (***B***) **Ergosterol sub-populations**: ergosterol in endoplasmic reticulum (*X*_32_), ergosterol steryl-ester-1 (*X*_35_), ergosterol associated with the complex sphingolipids (*X*_37_), plasma membrane ergosterol in inner leaflet (*X*_39_), ergosterol steryl-ester-2 (*X*_40_).

**Figure 5:**
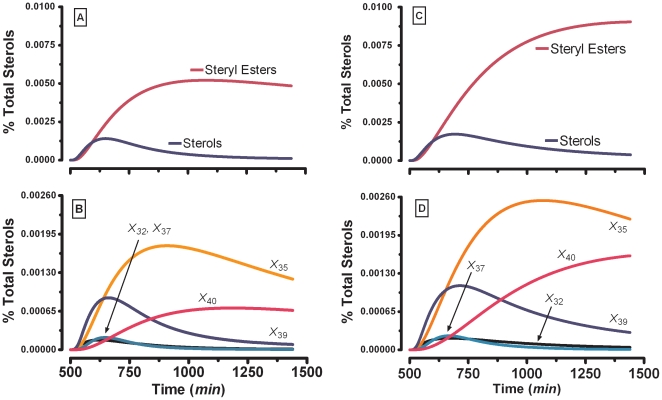
SL-E model results for an external pulse-chase bolus similar to the one in **Fig. 3F**. **Pools**: Steryl-esters (*X*_33_+*X*_34_+*X*_35_+*X*_40_), Sterol (*X*_30_+*X*_31_+*X*_32_+*X*_36_+*X*_37_+*X*_39_). **Ergosterol sub-populations**: ergosterol in endoplasmic reticulum (*X*_32_), ergosterol steryl-ester 1 (*X*_35_), ergosterol associated with the complex sphingolipids (*X*_37_), plasma membrane ergosterol in inner leaflet (*X*_39_), ergosterol steryl-ester 2 (*X*_40_). (***A***) Decrease to 1% in IPC synthase activity (*X*_133_). Dynamic simulation for the steryl-esters pool and total sterols after pulse-chase bolus with labeled acetate. (***B***) Decrease to 1% in IPC synthase activity (*X*_133_). (***C***) Decrease to a 1% in serine palmitoyl transferase activity (*X*_157_). Dynamics simulation for the steryl- esters pool and total sterols after pulse-chase bolus with labeled acetate. (***D***) Decrease to a 1% in serine palmitoyl transferase activity (*X*_157_).

As a first analysis, the ability of the model to predict dynamic changes in sterol metabolism was evaluated. Taylor and Parks conducted [^14^ *C*]-acetate bolus (Fig. 3A) and pulse-chase (Fig. 3D) experiments quantifying the levels of *de novo* sterols and steryl-esters produced during exponential growth and with glucose in the medium [42]. For comparisons between the sterol and the steryl-ester pools, they corrected the radioactive fatty acids incorporated in the steryl-ester fraction by multiplying the counts per minute by 27/43. We executed simulations of this scenario (Figs. 3B, 3E, 4B, and 5) by suppressing the incorporation of labeled palmitoyl-CoA (*L*_12_) at the stage of steryl-esters (*L*_33_, *L*_34_, *L*_35_), which we achieved by alteration of the steryl-ester synthase enzymes (*X*_181_, *X*_183_) (see *Equations S1, S2, S3, S4, S5, S6* for details of this specific implementation). When the labeled palmitoyl-CoA (*L*_12_) is included as co-substrate for the formation of labeled steryl-esters, the model indicates a moderate increase in the maximal amplitude (MA) of the steryl-ester pools (simulation results not shown).

The SL-E model dynamics for sterols and steryl-esters after an external acetate bolus (Fig. 3B) shows qualitative concordance with the experimental results (Fig. 3A) at least for the middle exponential and steady-state growth phases quantified in parallel by Taylor and Parks (see Figs. 1B and 2A in ref [42]). Both the experiments and simulations reproduce the increase and slow decrease in the steryl-ester pool.

Fig. 3E represents the SL-E model dynamics for sterol and steryl-ester when the model is perturbed with an external acetate pulse-chase (Fig. 3F). The simulations (Fig. 3E) show qualitative concordance with Taylor and Parks' experiments (Fig. 3D), at least in the early phase of the experiment.

Fig. 4A represents the dynamics of CS, following a simulated 30-min labeled acetate pulse-chase bolus, as shown in Fig. 3F. Even that they are not directily compared, because the radioactive labeling is different, the MAs of inositol phosphorylceramide (IPC, *X*_20_), mannosylinositol phosphorylceramide (MIPC, *X*_21_), and mannosyldiinositol phosphorylceramide (M(IP)_2_C, *X*_22_) exhibit the same order rank to those reported for a 30-min [2-^3^ *H*]-inositol pulse-chase experiment [47] during exponential growth. In the simulation (Fig. 4B), the inner PM (*X*_39_) shows an early increase which is probably due to the contribution of the flux through Yeh2p (*X*_182_). The CS ergosterol pool (*X*_37_) increases mostly as a consequence of the external acetate label, given as a pulse-chase as in Fig. 3F. Ergosterol-ester sub-pool-1 (*X*_35_) is the steryl-ester with the early sustained increase, while the steryl-ester in close contact with the PM (*X*_40_) shown a latter sustained increase. The outer PM ergosterol (*X*_36_) is not represented because of its low value. The Steryl Lanosterol (*X*_33_) and Steryl Zymosterol (*X*_34_) present an initial rise and posterior plateau starting in 700 min. At the end of the simulation the Steryl Lanosterol (*X*_33_) and Steryl Zymosterol (*X*_34_) present higher values than the Steryl Ergosterols pools (*X*_35_ and *X*_40_) (dynamics not shown).

The SL-E model is available in PLAS [48] and Matlab® [49] codes in *Materials S3 and S4* and *Materials S5 and S6* respectively. *Table S11* presents the PLAS [48] into Matlab® [49] conversion equivalence for the SL-E model dependent variables, pyruvate, external acetate, and palmitate.

### Effects of drugs on the SL-E pathway

In order to predict the sterol and steryl-ester dynamics when key sphingolipid biosynthetic enzymes are impaired, the effects of specific drugs affecting enzymes of the sphingolipid biosynthetic route were decreased by 99% in their specific activities. In the first analysis, we decreased IPC synthase (*X*_133_) activity, emulating a pathway block exerted by drugs such as aureobasidin, galbonolide, or khafrefungin [50]. Figs. 5A and 5B present the dynamics of sterols and steryl-esters, as well as other intermediate metabolites, in the SL-E model with reduced IPC synthase activity (*X*_133_) for a 30-min external labeled acetate pulse-chase perturbation. The knockout conditions and posterior perturbations where done with one minute of difference assuming the drug have reached their enzyme site of action after that time. The comparison of Fig. 5A with Fig. 3E (control) indicates that the sterols and the steryl-ester pools are not affected by the decreased IPC synthase activity.

The comparison of ergosterol sub-populations in Figs. 4B and 5B shows that the decrease in the IPC synthase activity produces a 75% decrease in the MA of ergosterol associated with CS (*X*_37_). This suggests that the vesicular co-transport of ergosterol and CS is principally responsible for the decrease in CS ergosterol (*X*_37_). The inner PM ergosterol (*X*_39_) presents an MA increase of 14% which indicates a redistribution of the ergosterol from the outer to the inner PM leaflet. Pools not predominantly affected by a decreased IPC synthase activity are: ergosterol-ester-1 (*X*_35_), ergosterol-ester-2 (*X*_40_), the endoplasmic reticulum ergosterol (*X*_32_), and the outer PM ergosterol not associated with DIMs (*X*_36_) (not shown).

The second set of simulations involved drugs such as myriocin, which inhibit serine palmitoyltransferase (SPT; *X*_157_) [50]. Figs. 5C and 5D show the effects of the corresponding perturbation in the SL-E model, which was implemented in a fashion similar to the experiment in Fig. 3F. When comparing Figs. 5C and 5D with their respective controls (Figs. 3E and 4B), three aspects stand out. First, the sterol and steryl-ester dynamics take more time for recovery from the pulse-chase perturbation. Second, between Fig. 5C and Fig. 3E, there is a significant increase in the sterol-ester pool. Third, between Fig. 5D and Fig. 4B, the MA of the CS-associated ergosterol pool (*X*_37_) decreases by 63%. As in the case of IPC synthase inhibition, much of this decrease in the CS-associated ergosterol pool (*X*_37_) can be explained by the impaired contribution of the vesicular transport of ergosterol and CS from this pool. Fourth, there is a 43% increase in MA of ergosterol at the inner side of PM (*X*_39_), which reflects the ergosterol redistribution dynamics inside the PM when the ergosterol associated with the CS is impaired. This increase is qualitatively consistent with the increase in free ergosterol in PM under *lcb1-100* conditions in Bauman's model (see Fig. 4B in ref [25]). To our knowledge, this increase at the inner leaflet of the PM has not been experimentally tested for *Saccharomyces cerevisiae*, and it seems difficult to validate the model result because it is not easy to quantify lipids within lipid rafts. Some technologies have been developed to analyze larger detergent resistant structures and to quantify lipids at the inner and outer leaflets of mammalian cells [51]–[53], but, in spite of recent progress [54], a crisp lipid characterization of rafts in yeast is still no possible. Fifth, the endoplasmic reticulum ergosterol (*X*_32_) increased slightly (Figs. 5D vs. 4B) as predicted by Baumann's model (see Fig. 4B in ref [25]).

The modest 26% increase in ER ergosterol (*X*_32_) from Fig. 5D vs Fig. 4B is in accordance with the difficulty to visualize the intracellular ER ergosterol redistribution for *lcb1-100* strains at restrictive temperatures by fluorescent filipin-stained sterols [25]. Specifically, Baumann and collaborators recorded, for *lcb1-100* strains at a restrictive temperature, an increase in the intracellular ergosterol as detected by the fluorescent ergosterol-binding protein filipin, but they were unable to visualize a change in the endoplasmic reticulum filipin staining pattern. In addition, they observed a slower equilibration rate for the ER-PM [^3^ *H*]-ergosterol in *lcb1-100* strains at both permissive and restrictive temperatures. This slower ER ergosterol (*X*_32_) recovery dynamics can also be observed in our simulation (Fig. 5D *vs.* 4B). Baumann and collaborators proposed that this slower *lcb1-100* recovery is to be expected for a non-vesicular ergosterol flux transport, which in the wild type strain is already at maximal capacity. However the *lcb1-100* ergosterol redistribution, based solely on the filipin-stained results, needs to be taken with caution because of recent reports that filipin principally stains ergosterol not associated with sphingolipids [55].

In summary, the modified SL-E model with reduced SPT activity (*X*_157_) shows a rise in the PM inner ergosterol (*X*_39_) and ER ergosterol (*X*_32_), a decrease in the PM outer ergosterol associated with the complex sphingolipids (*X*_37_), and a general slower recovery dynamics, which can be explained as a consequence of the decrease in the vesicular ergosterol flux and the increase of the non-vesicular ergosterol moving through the fluxes *v*_32,39_ and *v*_39,32_ (see Fig. 1).

### Total mass simulations

A total mass analysis was executed in order to validate the SL-E model against literature experimental data in which the total amount of sterols sub-populations and complex sphingolipids was quantified under different experimental conditions [3], [25], [47].

The simulation of the effects of a greatly reduced activity (1%) of SPT; (*X*_157_) on the total mass in the SL-E model shows a 26% and 55% decrease in the CS-associated ergosterol (*X*_37_) after 60 min and 120 min of the computational experiment, respectively (Table 1).

**Table 1:** Steady-state total mass of ergosterol sub-populations in the SL-E model under different experimental conditions, after 60 min.

	ER Erg.	PM Outer Erg.	DIM Erg.	PM Inner Erg.
	*X*_32_	*X*_36_	*X*_37_	*X*_39_
Wild Type (% total sterols)	9,51	4.75	42.80	47.55
1% SPT	*1.09*	*1.20*	*0.74*	*1.13*
1% SPT (120 min)	*1.20*	*1.28*	*0.45*	*1.23*
1% IPC synthase	*1.05*	*1.19*	*0.72*	*1.11*
12.5% Ceramide synthase	*1.03*	*1.04*	*0.95*	*1.03*

Wild type condition is represented with basal values; the other experimental conditions correspond to fold changes with respect to the wild type after decreasing the specific activity of serine palmitoyl transferase (*X*_157_), IPC synthase (*X*_133_), and ceramide synthase (*X*_134_) to 1% or 12.5%.

When the 1% SPT condition is implemented in the SL-E model (used for the dynamic simulations from Figs. 3, 4, and 5), there is a 20% and 44% decrease in the CS-associated ergosterol (*X*_37_) after 60 and 120 minutes, respectively (not shown). In the literature a similar 23% decrease in the DIM associated ergosterol (see Table 1 in ref [25]) and a 53% decrease in the DIGs (see Fig. 4b in ref [3]) had been recorded at the restrictive temperature for the *lcb1-100* relative to wild type cells after 60 and 120 minutes, respectively.

Table 1 ergosterol DIM associated (*X*_37_) reduction occurs in spite of the reduced vesicular flux, which does not represent more than 2% of the total ergosterol forward-backward flux (see *Table S3* for flux values).

Simulations with 1% IPC synthase activity (Table 1) show results similar to those obtained with 1% SPT. Both exhibit decrease of the CS-associated ergosterol (*X*_37_) and increases in the non-CS associated ergosterol sub-populations.

Ceramide synthase (*X*_134_) was decreased to 12.5% of its basal value (Table 1), simulating a decrease in enzymatic activity that was reported when 100 µM of Fumonisin B1 was added to the medium (see Fig. 6A in ref [47]). The effects of this modification on the ergosterol sub-populations were modest and changes in these pools of ergosterol did not go beyond 5%, thus, demonstrating minimal effects.

**Figure 6:**
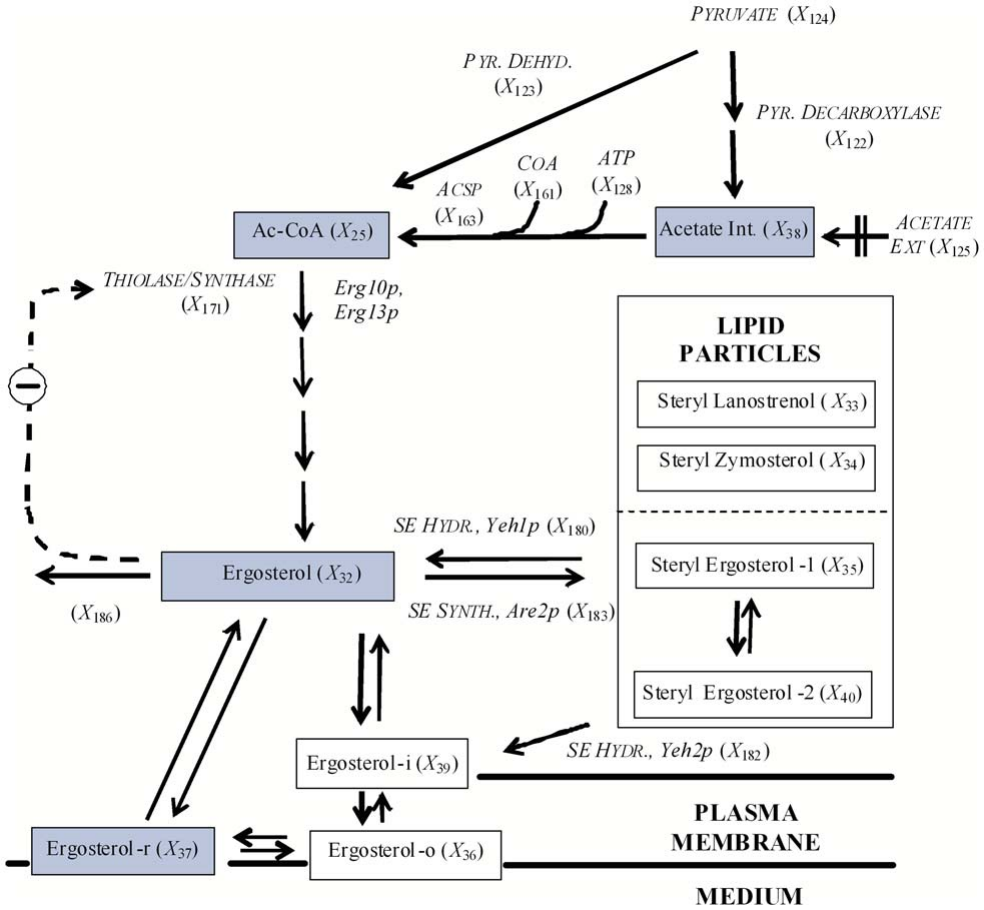
Yeast ergosterol diagram based on the highest sterol related SL-E logarithmic gains (metabolites) from Table S6. The diagram summarizes the ten time dependent metabolites of the ergosterol pathway associated with the highest sum of logarithmic gain magnitudes that are bigger than 1 (see Fig. 1 and Table S6). Solid boxes show time dependent variables and italics represent time independent variables. Consecutive arrows represent multiple enzymatic reactions, and the dashed arrow indicates a feedback signal. Blue boxes represent metabolites that also appear in the sphingolipid pathway (Fig. 2). Abbreviations for the time dependent variables: Acetyl-CoA (Ac-CoA), *X_25_*; Endoplasmic reticulum ergosterol (Ergosterol), *X_32_*; Steryl Lanosterol, *X_33_*; Steryl Zymosterol, *X_34_*; Steryl Ergosterol in Bulk Lipid Particles (Steryl Ergosterol-1), *X*_35_; Outer Leaflet Ergosterol in the Plasma Membrane (Ergosterol-o), *X*_36_; Detergent insoluble plasma associated ergosterol (Ergosterol-r), *X*_37_; Internal Acetate (Acetate Int.), *X*_38_; Inner Leaflet Ergosterol in the Plasma Membrane (Ergosterol-i), *X*_39_; Steryl Ergosterol in Plasma Membrane Associated Lipid Particles (Steryl Ergosterol-2), *X*_40_; Golgi Associated Complex Sphingolipids (CS-g), *X*_8_+*X*_18_+*X*_19_; Plasma Membrane Associated Complex Sphingolipids (CS-m), *X*_20_+*X*_21_+*X*_22_.

With the exception of the ceramide synthase experiments, which result in values within 5% of the basal values for any of the tested conditions, the remaining non-CS associated ergosterol sub-populations (free ergosterol) present a slight but consistent increase under the reduced SPT and IPC synthase conditions (Table 1).

The first column from Table 2 shows a decrease of the total PM ergosterol of 4 and 11% for the SPT condition at 60 and 120 minutes respectively. This decrease is also reflected in the second and fourth columns for the total ergosterol sub-populations and total sterols respectively.

**Table 2:** Steady-state values of PM ergosterol, total ergosterol, total ergosterol-esters, total sterols, and total steryl-esters in the SL-E model under different experimental conditions, after 60 minutes.

	PM Ergosterol	Total Ergosterol	Total Erg. Esters	Total Sterols	Total Steryl Esters
	*X*_36_,*X*_37_, *X*_39_	*X*_32_, *X*_36_, *X*_37_, *X*_39_	*X*_35_, *X*_40_	*X*_30_, *X*_31_, *X*_32_, *X*_36_, *X*_37_, *X*_39_	*X*_33_, *X*_34_, *X*_35_, *X*_40_
Wild Type (% total sterols)	95.1	104.61	45.7	112.91	62.2
1% SPT	*0.96*	*0.97*	*1.05*	*0.98*	*1.05*
1% SPT (120 min)	*0.89*	*0.92*	*1.11*	*0.94*	*1.12*
1% IPC synthase	*0.94*	*0.95*	*1.02*	*0.95*	*1.01*
12.5% Ceramide synthase	*1.00*	*1.00*	*1.01*	*1.00*	*1.01*

Wild type condition is represented by the sum of the basal values; the other experimental conditions correspond to fold changes with respect to the wild type after decreasing the specific activity of serine palmitoyl transferase (*X*_157_), IPC synthase (*X*_133_), and ceramide synthase (*X*_134_) to 1% or 12.5%.

The Table 2 reduction in total PM ergosterol, under the condition of the decreased SPT, at 60 minutes, is smaller than reported for the *lcb-100* strain after 60 minutes at the restrictive temperature, using immunoblotting Gas1p antibodies (see Fig. 5A in ref [25]). The reason for this difference may be that the marker Gas1p is not properly delivered to the plasma membrane in *lcb1-100* cells [56].

With the exception of the condition with decreased SPT at 120 minutes for PM ergosterol, the rest of the metabolites in Table 2 show small variations with respect to the wild type basal values in the first row. Similar to the simulations, no significant variations are reported experimentally when one compares the total ergosterol and ergosterol-esters from wild type vs. *lcb1-100* strains at permissive or restrictive temperatures (see Table 1 in ref [25]).

In spite of the congruence between the SL-E model predictions and experiments addressing total ergosterol and ergosterol-ester [25], it must be cautioned that the SL-E model does not include other organelles rich in sterols, such as endosomes, or vacuoles that might contribute to the total ergosterol mass [57].

In general, a decrease in complex sphingolipids was observed for all simulated conditions (Table 3). The complex sphingolipids of the golgi and, in particular the IPC (*X*_8_) are the metabolites most affected in all computationally tested conditions (not shown individually). Table 3 decreases in complex sphingolipids are expected because the simulations affect the enzymes of *de novo* sphingolipid synthesis that are needed for the formation of complex sphingolipids. The finding is indirectly consistent with results of labeling the *lip1* mutant (ceramide synthase) [58], and even more with labeling after myriocin treatment [59], which suggest a decrease in sphingolipid biosynthesis that is less evident when one examines MIP_2_C rather than the IPC.

**Table 3:** Steady-state total mass of complex sphingolipid sub-populations in SL-E model under different experimental conditions, after 60 minutes.

	IPC	MIPC	M(IP)_2_C	CS
	*X*_8_, *X*_20_	*X*_18_, *X*_21_	*X*_19_, *X*_22_	*X*_8_, *X*_18_, *X*_19_, *X*_20_, *X*_21_, *X*_22_
Wild Type (mol%)	1.02	1.40	0.085	2.505
1% SPT	*0.34*	*0.68*	*0.57*	*0.54*
1% SPT (120 min)	*0.08*	*0.49*	*0.54*	*0.32*
1% IPC synthase	*0.35*	*0.66*	*0.63*	*0.53*
12.5% Ceramide synthase	*0.83*	*0.92*	*0.94*	*0.89*

Wild type condition is represented by the sum of the basal values; the other experimental conditions correspond to fold changes with respect to the wild type after decreasing the specific activity of serine palmitoyl transferase (*X*_157_), IPC synthase (*X*_133_), and ceramide synthase (*X*_134_) to 1% or 12.5%. Complex sphingolipids: IPC in Golgi compartment plus plasma membrane (*X*_8_+*X*_20_), MIPC in Golgi compartment plus plasma membrane (*X*_18_+*X*_21_), M(IP)_2_C in Golgi compartment plus plasma membrane (*X*_19_+*X*_22_). CS represents the total of complex sphingolipids.

Table 3 most significant variation in complex sphingolipids is observed for reduced SPT activity, which results in a 66% and 92% decrease in IPC synthase at 60 and 120 minutes respectively. Overall, this condition reduces the complex sphingolipids levels by 46% and 68% at 60 and 120 minutes, respectively.

Table 3 shows similar reductions in complex sphingolipids under 1% IPC and 1% SPT conditions. A decrease in ceramide synthase activity leads to a much smaller decrease in the complex sphingolipids after 60 min. The simulated effect of fumonisin B1 is significantly smaller than reported in an experimental study (see Fig. 7B in ref [47]); however, in this study the complex sphingolipid levels were measured after longer fumonisin B1 application (6 generations).

## Discussion

For the first time, the sphingolipid and the ergosterol biosynthetic routes have been integrated into a single mathematical model.

Model validation was accomplished by comparing the dynamics of sterols and sterol esters with published time courses. Under different conditions and strains, the sphingolipid-ergosterol model (SL-E) was able to simulate the experimental levels of specific ergosterol sub-populations obtained with the *lcb1-100* strain, a temperature conditional mutant for the SPT, the first enzyme of the sphingolipid route.

The validated SL-E model was used to predict the metabolomic dynamics of the sphingolipid-ergosterol pathways after drug treatment. Specifically, we simulated the effect of drugs affecting sphingolipid and sterol enzymes and studied subsequent changes in sterols and sterol esters sub-populations.

Sensitivity analysis indicates that the SL-E model is robust, showing generally low sensitivities for the parameters with respect to concentrations and fluxes (see Tables S5, S6, S7, S8, and S9). The higher sensitivities are mostly associated with metabolites that are also involved in other pathways, which are not modeled here, a phenomenon that was similarly observed in the sphingolipid system [38].

Fig. 6 presents the mechanisms involved in the ER-plasma membrane ergosterol transport with the highest log-gain sensitivities, including thiolase/synthase (*X*_171_). Because the thiolase/synthase is a common enzyme shared with mammalian cells, only the mechanisms associated with ergosterol transport are interesting as antifungal drug targets. These ergosterol processes are in fact the target of polyenes antifungals such as Nystatin and Amphotericin B [35]. The SL-E model was subjected to gross and fine tuning validations. First, it was tested against measurements of the dynamics of total sterol and steryl-esters [42], and indeed the simulated dynamic trends were qualitatively consistent with experimental data describing the dynamics of *de novo* synthesis of sterols and steryl-esters after perturbations with external radioactive acetate (Fig. 3). Second, with more refined data under different experimental conditions and strains [3], [25], the SL-E model was able to simulate correctly the levels of specific ergosterol sub-populations mimicking the experimental results obtained in the *lcb1-100* strain (which shows near total loss of SPT activity at restrictive temperatures) by decreasing the SPT (*X*_157_) to 1% their basal value. This suggests that the vesicular co-transport of ergosterol and CS is principally responsible for the decrease in CS-associated ergosterol (*X*_37_). Interestingly, a similar 34% decrease for the newly synthesized cholesterol in rafts was reported in mammalian kidney cells when the vesicular transport from the Golgi compartment to the cell surface was impaired by decreasing the incubation temperature from 37°C to 15°C [60].

Also, both the *lcb1-100* mutant strain [25] and the SL-E model showed a 50% decrease in complex sphingolipids (CS) with respect to the wild type strain after 60 minutes (Table 3). At first glance, this CS decrease may suggest a proportional 50% reduction in the DIM-associated ergosterol. However, this is not supported by experimental results as reported by the Bauman group [25] nor by the simulation results with the SL-E model. Instead, both show a decrease of around 30% in CS-associated ergosterol (*X*_37_) (Table 1). This result highlights the importance of the biosynthetic pathways as complex systems that are able to affect the PM ergosterol levels. Given that the experimental observations were not used for model construction or parameterization, the favorable comparisons between data and simulation results thus provide a significant measure of validation.

As shown in Fig. 1, the SL-E model does not include the PM's inner microdomains. This raises the question of whether the percent reductions for the *lcb1-100* DIM-associated ergosterol [25] and the 1% SPT CS-associated ergosterol (*X*_37_) (Table 1) are both similar, since the inner microdomain ergosterol can probably contribute to the DIM-associated ergosterol. As noted in the introduction, the ergosterol-phospholipid interactions of the plasma membrane's inner microdomains depend principally on the degree of fatty acid saturation of the phospholipids. Devaux and Morris comment that the spatial-temporal coexistence of the rafts at both sides of the membranes probably depends on the transmembrane proteins and/or the degree of membrane curvature [61]. We suggest here that their co-localization probably also requires the interdigitations of the inner lipids with the very long CS fatty acid chains tails for their formation and stabilization [26]. However, until we gain further knowledge, it is not known if these forces are strong enough to be maintained during DIM purification. Another likely reason for the similarity between the simulations and the experimental results for the CS-associated ergosterol after decreasing the SPT activity is that the proportion for the inner/outer plasma membrane ergosterol microdomains is not affected in the *lcb1-100* mutants and/or by the different experimental conditions reported by Baumann and coworkers [25]. More experimental evidence is necessary to clarify which ergosterol sub-population(s) are extracted within the DIM pool.

It is also important to remember that the comparison of simulated SPT inhibition (Figs. 5C–5D) with *lcb1-100* results [25] needs to be considered with caution. The *lcb1-100* strain exhibits lower complex sphingolipid levels even at a permissive temperature [25], and has a greater sensitivity to stress [62]–[64].

As a whole, from the predictions from Fig. 5 and Tables 1, 2 and 3, we can conclude that a decrease in the CS plasma membrane levels will concomitantly produce: *a*) a reduction in the plasma membrane CS-ergosterol level, *b*) a moderate increase of the free ergosterol sub-populations, and *c*) slightly longer sterol recovery times after [^14^ *C*]-acetate perturbations. The steryl-ester dynamics are affected only when the specific activity of SPT was decreased.

Predictions in Table 2 showed that the levels of the sterols and steryl-esters are not significantly modified when key enzymes of the sphingolipid pathway are affected. This homeostatic phenomenon has been observed in yeast [36] and mammalian cells [65], which by diverse strategies are able to maintain the sterol and steryl-ester sub-populations within an optimum range. With the exception of 1% SPT at 120 minutes, Table 2 indicates that the sterols and steryl esters pools are inside a few percent (6%) of their respective basal values in spite of some aggressive *in silico* conditions tested, such as in the *lcb1-100* temperature conditional mutant.

The SL-E model is able to reproduce the very efficient homeostatic sterol balance in yeast that has been observed under different experimental conditions (*e.g*. [25]). The SL-E model homeostasis encompasses the ergosterol biosynthesis precursors, end product feedback regulation, sub-cellular sterol compartmentalization, the plasma membrane ergosterol flip-flop, and the back and forth movement of microdomain ergosterol in the plasma membrane. The model also includes sub-populations of lipid particles as important depositories of esterified sterols. Finally, the ergosterol portion of the model shares precursor metabolites and co-transport processes with the sphingolipid-glycerolipid pathway.

The SL-E simulation of labeled acetate pulse chase (Fig. 3D) does not entirely match the results of [^14^ *C*]-acetate chase experiments for the sterol and steryl-ester pools, especially for the steryl-esters (Fig. 3E). These differences between experimentation and simulations can be caused by diverse factors inside and/or outside the modeling framework. Within the modeling construction, two main factors could affect the dynamics of sterols and steryl-esters. The first is that Taylor and Parks' pulse chase [^14^ *C*]-acetate experiments included labeled contributions from not modeled organelles that may contain sterols, such as the mitochondria, endosomes, the nucleus, and the vacuoles [33], [57]. The other probable reason is that the model does not include the long list of all sterols [29], [66] and steryl-esters [40], [41], [67] that are present along the ergosterol biosynthetic route in yeast.

Outside the modeling framework, four main causes could contribute to the observed difference between the experiments and simulations. The first relates to the CPM correction factor of 27/43 employed in the experiments of Taylor and Parks (calculated with 16 palmitoyl-CoA and 27 sterol carbons) [42], which does not totally subtract the CPM contribution of the fatty-acids from the steryl-esters because the steryl-ester synthase (*X*_181_, *X*_183_) can catalyze the incorporation of fatty-acyl chains of different lengths [68]. The second factor is the assumption that the steryl-ester fatty acids and the sterols have the same specific activity which, as Taylor and Parks state, is not necessarily true, especially when pulse-chase experiments are conducted (Fig. 3D). A third factor, that possibly affected Taylor and Parks' dynamic pulse-chase experiments (Fig. 3D), was the procedure of washing the cells with unlabeled medium to remove the [*U*-^14^C] potassium acetate after 30 minutes of labeling. This process raises the possibility that the chase contributes to the sharp decrease in the radioactivity incorporation rates observed at the third and fourth time points for the sterols and steryl-esters [42]. Fourth, the time points of initiating the [^14^ *C*]acetate pulse and the pulse-chase experiments in the studies of Taylor and Parks are not clear, and the first documented time points correspond to 250 minutes (Fig. 3A) and 566 minutes (Fig. 3D) (see Figs. 1C and 2B in ref [42]). Paralleling the time course used by Taylor and Parks (see Figs. 1B and 2A in ref [42]), the SL-E simulations reflect initiation of treatment in the middle of the exponential growth phase (Figs. 3B, 3E, 4, 5, and Fig. S2).

During the development and implementation of the SL-E model, two aspects of the sterol and steryl-esters biology were highlighted. First, in spite of the fact that the esterified pools and associated enzymes are not essential for yeast viability [69], [70], these studies show that the dynamics of sterols and steryl-esters depends profoundly on the lipid particles (LP) organelles. Second, the importance of clarifying the relationship between the LP sub-pool-2 (*X*_40_) and the Plasma Membrane Associated structures described by the Daum group was realized [71], since these two lipidic structures are both rich in sterol enzymes (as the Erg1p and the Erg6p) [32], [72] and both are in close association with the PM [69].

The scope of the SL-E model is quite broad. Foremost, it can complement experimental research as a tool aiding our understanding of yeast lipidomics under different conditions. For instance, the comprehensive sensitivity analysis of the model (see *Tables S5, S6, S7, S8, and S9* for details) facilitates the identification of processes that influence the pathway more than others. *Tables S5, S6, S7, S8, and S9* shows that there are three principal groups of sterol related metabolites with different degrees of sensitivity, namely the sterol precursors, the ergosterol sub-populations, and the steryl-ester sub-populations, of which the last group exhibits the highest sensitivities. These three blocks of sterol-related metabolites are expected to be more influenced by perturbations from the rest of the SL-E model.

The model is also a valuable tool for testing hypotheses regarding the dynamics of sterols and their acylated forms after specific changes in sphingolipid and/or sterol associated enzymes from the SL-E model (*e.g*., *Table S10*). An example of this type of analysis was recently executed with the sphingolipid pathway alone [38]. Finally, the proposed model could serve as the basis for a global SL-E model, which could support predictions of changes in lipid levels under untested conditions. An analysis of this type with a sphingolipid model helped with the interpretation of microarray data characterizing the diauxic shift [73].

As pathway models become more comprehensive, it will become increasingly difficult to keep track of all details and to test, diagnose, and validate them. This difficulty may be addressed in different ways, for instance, by focusing on the modularity of biological systems, complexity reduction per time scale separation, or by designing tools for the creation, analysis, and sharing of models that are based on biologically motivated rules [74]. These strategies should also include techniques for the nontrivial representation of polymerization processes in pathway systems where chemical species are attached to or removed from larger polymeric molecules, such as complex carbohydrates, fatty acids, ribonucleic acids or membranes. Such techniques will be especially relevant for processes such as the ones described here, where the dynamics of fatty acids and their interactions with lipid membranes are critical.

## Methods

### Considerations for Model Construction

#### General Assumptions

We know that sterol biosynthesis is localized in the ER [29] and that both the sterol and the sphingolipid pathways share acetyl-CoA as a building block for fatty acyl and mevalonate formation, respectively (Figs. 1 and 2). The SL-E model considers palmitoyl-CoA as the only initial substrate (Fig. 1) and does not account for different acyl chain lengths.

Ultimately both pathways probably converge to form cargo vesicles and plasma membrane microdomains composed of ergosterol and complex sphingolipids [75] (Figs. 1 and 2).

Under aerobic exponential growth, the metabolites involved in the heme-independent section are present at very low levels, which is assumed to be a consequence of an efficient conversion into sterols by the heme-dependent enzymatic reactions downstream [76]. A consequence of the low concentration is a paucity of literature information about metabolites preceding lanosterol. To circumvent this issue, the presence of heme-independent metabolites is often experimentally characterized with strains containing specific gene deletions or with the inhibition of enzymatic activity by means of drugs and toxins [20], but it is uncertain to what degree the results from such experiments are representative for the wild type strain. Because of the lack of sufficient and relevant information, the heme-independent section of the model was limited to metabolites reported under aerobic conditions (Fig. 1).

In the downstream heme-dependent section, we specifically included the enzymes Erg1p, Erg11p, and Erg6p. The reasons for this inclusion are the following: First, gene over-expression studies of Veen and collaborators suggest that Erg1p and Erg11p are the major regulatory steps for the ergosterol biosynthetic route [77]. Second, Erg11p is the crucial juncture for functional sterol molecules, because enzymatic disruptions at or before this point result in cessation of sterol prototrophy, while gene knockout mutants between Erg6p and the end of the route are viable even if significant changes are observed in the membranes of these strains [29], [36], [78].

It became evident during the modeling process that it would be beneficial to subdivide the total ergosterol pool into sub-pools corresponding to free ergosterol and ergosterol associated with detergent-insoluble membranes (DIM), which could have different properties and functions (*e.g*., [25]). The DIM pool is considered as a single pool, although in reality it is likely a conglomerate of individual plasma membrane microdomains [54].

Preliminary model analysis implied the need to include the end product feedback regulation of ergosterol. Furthermore, it is reported that ergosterol promotes transcriptional feedback regulation upon *ERG10*, the gene coding for acetoacetyl-CoA thiolase [79]. Other feedback regulation, such as the translational repression of mevalonate HMG-CoA reductase by its product mevalonate [80] or external allosteric ergosterol inhibition [81], were not included because these regulatory mechanisms are only active under conditions that are different from those addressed by the model. The effect of genetic regulation by mevalonate on the HMG-CoA reductase isoenzymes *HMG1* and *HMG2* occurs during anaerobic growth and in cells moving from log-growth to stationary phase. Specifically, *HMG1* is preferentially expressed during logarithmic aerobic growth, and *HMG2* is translated during the stationary phase and/or under anaerobic growth [80]. Allosteric inhibition of Erg10p by ergosterol is exerted by external ergosterol [81], but only under anaerobic conditions. When oxygen is available in the medium, *S. cerevisiae* preferentially synthesizes its own ergosterol even in the presence of external sterols [82].

The sphingolipid component of the SL-E model includes the *de novo* and salvage production of ceramides, as well as the formation and transport of complex sphingolipids between the Golgi and the plasma membrane. It also includes glycerolipids directly related with the sphingolipid pathway and the formation of CS from phytoceramide (*X*_7_) and dihydroceramide (*X*_3_), with phosphatidylinositol (PI; *X*_15_) as a cosubstrate, through the inositol phosphorylceramide synthase (*X*_133_) reactions (Fig. 2). Predictions with the sphingolipid model pointed toward the importance of PI for the dynamics of the complex sphingolipids, and this finding was confirmed experimentally by thin layer chromatography (TLC) time course data after pulse labeling with inositol [38]. Moreover, the sphingolipid model properly simulated the dynamics of long chain base phosphates LCB-P's (*X*_4_, *X*_6_) for the wild type and the sphingosine-phosphate lyase (*X*_150_) knockout [38].

#### Vesicular and non-vesicular ergosterol transport

Due to the lack of specific data for the proteins associated with the vesicular transport, the SL-E model does not include a specific protein (for example Sec1p, Sec14p, or Sec23p) involved in the transport of ergosterol and CS from vesicular ER to PM through the fluxes *v*_32,37_ and *v*_37,32_ [83], [84]. Instead, we described these transport processes with simple first-order mass action reactions, which had previously been shown to represent the kinetics of these mechanisms quite well [38].

The non-vesicular lipid transport is associated with transfer and transport proteins (generically called *transferases* and *flippases*), which facilitate the movement of lipids between membranes and the trans-bilayer forward-backward ER-PM ergosterol flip-flop. These flippases play an important role in maintaining ergosterol homeostasis at both sides of the membrane (*e.g*., [85]).

Because the lack of detailed kinetic information for the proteins associated with the non-vesicular lipid transport [84], [86] mass action formulations have been used in the literature for their description showing good agreement with experimental data [87], [88]. In the SL-E model, the mass action representation of non-vesicular transport also seemed appropriate, as judged by comparisons with experiments characterizing the sterol dynamics with *de novo* external [^14^ *C*]-acetate perturbations [42] and wild type vs. *lcb1-100* strain experiments measuring the mass of ergosterol in the plasma membrane [3], [25] (see result section).

The SL-E model does not include a trans-bilayer inner-outer leaflet flip-flop movement for the ergosterol DIM lipid-ordered pool (*X*_37_) because it seems that the contribution of this ergosterol pool is low due to its strong association with complex sphingolipids [87].

#### Assumptions regarding ergosterol and complex sphingolipids in raft formation

It is known that enzymatic reactions taking place in crowded lipid solutions behave differently than reactions occurring in water soluble media [89], [90]. Lipid-embedded enzymes can catalyze lipid translocations and microdomain association and dissociation [85], [86], [91], possibly leading to fractal kinetics, which is well described with power-law formulations that are at the core of BST [92]–[94]. We used such fractal kinetic representation for flux *v*_36,37_, which represents the efflux of the free outer leaflet ergosterol and their association with the complex sphingolipids from the plasma membrane.

No specific references were found about the possible localization of complex sphingolipids outside microdomains, but it has been reported that the bulk of ceramide is localized in ordered lipid domains with a small concentration of ceramide not associated with rafts [95]. The SL-E model initially assumes that complex sphingolipids of the PM are present in the compartment associated with the ergosterol in DIMs (*X*_37_) (see Fig. 1).

In addition to ergosterol, the inner leaflet of the yeast plasma membrane contains other abundant lipids, such as the anionic phosphatidylinositol (PI) and phosphatidylserine (PS) and the neutral phosphatidylethanolamine (PE) [96], [97].

To summarize these findings and assumptions, the current SL-E model represents a number of processes at the membrane, but does not account for specific structures at the inner leaflet of PM or for transmembrane proteins that could contribute to the formation of inner leaflet microdomains.

#### Lipid particles (LP)

No direct experimental evidence is available in the literature of how Yeh2p gets access to its substrate or if the sterol products of the Yeh2p reaction are used for PM biogenesis [69]. Thus, the SL-E model assumes that the entire flux going through Yeh2p (*X*_182_) contributes to the ergosterol pool (*X*_39_) at the PM inner leaflet (Fig. 1).

#### Total mass experiments

For the total mass simulations (Tables 1, 2, and 3), the SL-E model was balanced for the plasma membrane and for bidirectional lipid particle fluxes, which are assumed to be in equilibrium when the cells are no longer growing. This balancing is not entirely trivial, because the contributing compartments have different volumes, which complicate concentration and flux estimations (see *Table S3* for details). Moreover, because the yeast population at steady-state contains the same numbers of cells growing and decaying, and because yeast cell size [98] and organelles such as the vacuole [99] increase in size during aging, it was assumed that the steady-state fluxes associated with the ER, PM, and LP balance to zero. Finally, in the theorical treatment proposed by Baumann et al the transport of free sterols between the ER and PM, and between the pools of free and raft sterols are assumed both in equilibrium [25].

Specifically, the vesicular co-transport of ergosterol and CS and the fluxes of lipid particles were balanced. The flux *v*_40,39_, which is catalyzed by the plasma membrane hydrolase Yep2p (*X*_182_), was set to zero because Yep1p is the sole active steryl-ester hydrolase under heme-deficient conditions, while Yeh2p and Tgl1p are inactivated [100].

### Mathematical Methods

#### Modeling Framework and Equations

There are no *a priori* guidelines for setting up mathematical models of complex systems. In this situation, Biochemical System Theory (BST) [44], [46] has proven to be a very useful first default approach, especially if quantitative information is scarce. Generically, the dynamic of a metabolic pathway is modeled as a system of ordinary differential equations, namely
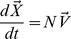
(1)


Where 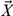 is the vector of metabolites, 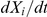 is the vector of derivates, *N* is the stoichiometric matrix, and 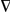
is the vector containing the fluxes *ν_i_* in the metabolic system. In BST, these fluxes are represented as power-law functions of the form
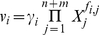
(2)


Each term contains exclusively those variables that have a direct effect on this flux. Each variable is raised to a power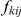, called a kinetic order, that quantifies the strength and direction of the effect, and the product of variables is multiplied with a rate constant 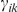 that quantifies the turn-over rate of the process [46], [101], [102].

Thus, each BST equation in the generalized mass action (GMA) format reads
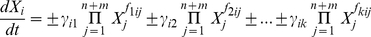
(3)(*e.g.*, [38], [39], [46], [102]). There are *n* equations, one for each *dependent* metabolite variable, and the products may also include up to *m* further *independent* variables whose concentrations remain constant over time. Typical examples are constant substrates and enzyme activities.

It is straightforward to design a GMA model from a metabolic diagram. In fact, this step can be accomplished with computer software [103]. Each metabolite of interest, *X_i_* (*i* = 1,…*n*), is represented by a differential equation that contains the difference between all influxes and effluxes, and these are formulated as specific power-law functions, as discussed above.

We therefore used our earlier GMA model of the sphingolipid-glycerolipid system [38], [39] and expand it to include the ergosterol pathway with the same methods (see *Materials S1* for details).

For stability and sensitivity analysis we linearized this system in a logarithmic coordinate system (cf. [43], [46]).

In order to compare our simulation results with data on sterol and steryl-ester dynamics reported in the literature [42], labeled and unlabeled metabolites were distinguished as separate variables within the same system. Methods for this type of dynamic labeling analysis were presented elsewhere [104], [105]. This approach to tracking labeled molecules through metabolic pathways was illustrated with the sphingolipid pathway in yeast [38] and the pentose pathway in *Zymomonas mobilis* [105]. Specific details are found in *Materials S1*.

#### Treatment of Sterol Sub-Pools

The ergosterol pool was subdivided into four sub-populations: ergosterol in the ER (*X*_32_), ergosterol associated with the inner and outer plasma membrane (PM) not in microdomains (*X*_36_, *X*_39_), and the CS associated ergosterol pool (*X*_37_). In *S. cerevisiae*, the highest ergosterol concentration is found in the PM [32], [33]. To assure consistency between the ergosterol pools in ER and PM, the ER ergosterol concentration was calculated from a known relationship between the two [25], [32] (see *Tables S1 and S2* for details). One ER ergosterol sub-pool is in close contact with the PM and associated with the PAM (PM associated membrane) structures described by Pichler and collaborators [71]. However, this PAM ergosterol concentration is similar to that estimated for ER bulk ergosterol, and is composed of the same ergosterol that is not associated with detergent-insoluble sphingolipid enriched complexes (DIGs) [3], which suggested a representation of ER ergosterol as a single time dependent variable (*X*_32_).

With no data available for the sterol plasma membrane trans-bilayer distribution in yeast [61], [106], we assigned equal ergosterol concentrations to both sides of the lipid bilayer. The reasons for this equal distribution are two: First, in spite of the asymmetric phospholipid distribution in the plasma membrane [96], it is known that the anionic glycerophospholipids (PI and PS) and the neutral or zwitterionic phospholipids (PC, PE, PC) can accommodate sterols equally well [106], [107]. This broad lipid affinity of the sterols is mainly caused by their affinity according with the degree of saturation of the fatty acyl chains, but the information about the asymmetry of saturated acyl chains in the literature is scarce. To a minor degree, the small polar head of the sterol can also contribute to the non-covalent sterol interactions, which occur mainly with the more homogeneous phosphatides and the hydrophobic fatty acid chains of the lipids, and to a lesser degree with the highly variable hydrophilic head groups. Second, the rapid flip-flop of ergosterol reported for different preparations and conditions [25], [86] contributes to the fast equilibration of free ergosterol.

It has been postulated that the inner plasma membrane ergosterol forms rafts with surrounding phospholipids and proteins, but their stability, degree of symmetric coexistence with the outer microdomains, and the extent of the forces that produce and maintain their existence, are not well understood [61], [106], [108]. As highlighted by Silvius' group [95] and others [26], the inner microdomains may be formed by other contributing factors outside the inner monolayer, such as the trans-bilayer penetration of very-long acyl chains [26], and transmembrane proteins associated with rafts [109]. Therefore, it was decided not to include microdomains at the cytoplasmic side of the plasma membrane in the SL-E model.

Finally, Baumann and collaborators established a 9∶1 relationship between free sterol and ergosterol associated with DIMs [25]. This relationship was used in the SL-E model for ergosterol on the outer side of the plasma membrane (PM), which is associated with CS (*X*_37_), thus leaving a smaller proportion of non-complex sphingolipid associated ergosterol (*X*_36_) at this side of the PM.

#### Treatment of Steryl-Ester Sub-Pools

The steryl-esters are confined to structures known as lipid particles (LP), which are composed principally of triacylglycerols, sterol-esters, and moderate quantities of triacylglycerol and ergosterol biosynthetic enzymes [67], [69], [110].

Similar to the treatment of the different ergosterol sub-populations, and assuring the correct relative concentrations between the different organelles, the steryl-esters levels where calculated from an observed linear relationship between the endoplasmic reticulum sterols [32] and the steryl-esters in LPs [40] (see *Tables S1, and S2* for details).

The ergosterol ester in LP was split into two sub-populations (*X*_35_ and *X*_40_) in a ratio of 10∶1, with the smaller pool (*X*_40_) in direct contact with the PM. The existence of LP sub-populations had been reported by different laboratories and by different approaches. First, Daum and collaborators observed two sub-populations of differently sized LPs [110]. Second, electron micrographs and indirect immunofluorescence microscopy showed that not all LP populations are PM associated [72], [111]. Third, Leber and collaborators proposed the possible existence of LP sub-populations in order to explain the differential Erg1p association with LP subsets [72]. Fourth, the LP ergosterol sub-pool (*X*_40_) is a consequence of the high STE hydrolase steryl ester activity (*X*_182_) detected in the PM [33], [110], [112], [113]. Finally, the observation that Yeh2p in yeh1*Δ*/tgl1*Δ* double mutant strains does not efficiently mobilize steryl-esters *in vivo* is consistent with limited access of Yeh2p to the full intracellular LP steryl-ester pool, as is predicted from the localization and topology of Yeh2p [112]. Very interestingly, the subdivision of steryl esters into sub-populations turned out to be necessary in our model to assure stability.

#### Representation of Fluxes

During exponential growth, the fluxes of complex sphingolipids in and out of the plasma membrane are not in steady state and move preferentially into the plasma membrane [38]. This dominant flux direction is also true for ergosterol, which moves back and forth in cargo vesicles together with the complex sphingolipids, a transport process that is indirectly modulated by Arv1p [2], [114] and mediated by vesicle associated proteins from the *SEC* family [83], [84].

The major ergosterol flux moves material from the ER to other membranes through close contact of large sections of the ER with the plasma membrane and with other organelles such as the plasma membrane and the mitochondria [71], [84]. For our modeling purposes, the total bidirectional flux of ER ergosterol toward the plasma membrane was estimated from experimental data of Sullivan and collaborators [115]. The total bidirectional flux of non-vesicular ergosterol from the ER to PM was subtracted from the vesicular flux, and the ergosterol ester hydrolysis flux through Yeh2p (see Fig. 1 and *Table S3* for details).

Fluxes, such as the bidirectional flux between ER and LP, were estimated from the assumption of a classic Michaelis-Menten process. The calculated fluxes between ER and PM indicate a net flux toward the steryl-ester formation, which is consistent with data addressing the exponential growth phase [41], [42], [82], [116]. Furthermore, two fluxes divert ergosterol from the biosynthetic route to other organelles that are not part of the model. The first one uses farnesyl diphosphate (*X*_28_) through the farnesyltransferase (*X*_179_) reaction to the formation of multiple isoprenoid metabolites, while the second flux channels ergosterol from the ER to the mitochondria and other organelles (*X*_186_). The latter flux is assumed to occur through direct ergosterol translocation, because reports [71], [84] show that multiple ER sections are in close contact with the mitochondria and other organelles. The remaining fluxes of the biosynthetic ergosterol pathway were computed from the stoichiometry of the system and the fluxes described before (see *Table S3* for details).

#### Acetate Influx

In the presence of glucose, *S. cerevisiae* represses expression of acetyl energy dependent transporters [117], [118]; under these conditions, the uptake of acetate is proportional to the external acetate concentration [119], [120]. The acetate uptake rate and the percent of incorporated, undissociated acetate were based on literature data [42], [121]. For baker's yeast growing in minimal medium supplemented with yeast extracts, the external pH ranges between 4.5 and 5 [119] and was reported to decrease steadily over the lifetime of the culture [119], [122], (Matmati, Nabil. 2010, personal communication). For our simulations with labeled acetate, we assumed a medium pH range between 4 and 4.5 and a proportion of 93% undissociated acetate (which is consistent with the pK_a_ of acetate). Thus, the uptake rate constant of undissociated acetate was set to 0.0022 [121] (see *Fig. S1* for details).

#### Model Diagnostics

Based on previously published models for the sphingolipid-glycerolipid pathway [38], [39], [73], [102], one should expect the SL-E model to be stable and only moderately sensitive. We tested and confirmed stability with standard methods of eigenvalue analysis, which yielded exclusively negative real parts (see *Table S4* for details). The presence of some non-zero imaginary eigenvalues permits oscillations in response to specific perturbations to the system; some of these oscillations were observed in the dynamics of some simulation results (*e.g.*, Figs. 3, 4, and 5, S2). Global model robustness is generally evidenced by low sensitivities for the majority of parameters with respect to metabolites and fluxes (see *Table S5* for details). The high percentage of sensitivities smaller than one in magnitude indicates that the vast majority of perturbations in parameters are attenuated by the model.

## Supporting Information

Figure S1 **Experimentally observed exponential decay trend line for external radioactive acetate.** Experimental points (triangles) data adapted from Wang *et al*. (see Fig. 2 in ref [121]). For all simulations presented in this manuscript, 100% of the acetate in the medium (*X*_125_) was replaced with radioactive acetate (*L*_125_).
(TIF)

Figure S2 **SL-E model dynamics after external radioactive acetate pulse bolus perturbation.** Perturbation similar to the one in Fig. 3C. Pools: Steryl-esters (*X*_33_+*X*_34_+*X*_35_+*X*_40_), Sterol (*X*_30_+*X*_31_+*X*_32_+*X*_36_+*X*_37_+*X*_39_). Ergosterol sub-populations: ergosterol in endoplasmic reticulum (*X*_32_), ergosterol steryl-ester-1 (*X*_35_), plasma membrane ergosterol in outer leaflet (*X*_36_), ergosterol associated with the complex sphingolipids (*X*_37_), plasma membrane ergosterol in inner leaflet (*X*_39_), ergosterol steryl-ester-2 (*X*_40_). (*A*) Wild-type condition. Dynamic simulation for steryl-esters pool and total sterols after pulse-chase bolus with labeled acetate. (*B*) Wild-type ergosterol and steryl-ester sub-populations. (*C*) Decrease to 1% in IPC synthase activity (*X*_133_). Dynamic simulation for the steryl-esters pool and total sterols after pulse-chase bolus with labeled acetate. (*D*) Decrease to 1% in IPC synthase activity (*X*_133_). Ergosterol and steryl-ester sub-populations. (*E*) Decrease to 1% in serine palmitoyl transferase activity (*X*_157_). Dynamic simulation for steryl-ester pool and total sterols after pulse-chase bolus with labeled acetate. (*F*) Decrease to 1% in serine palmitoyl transferase activity (*X*_157_). Ergosterol and steryl-ester sub-populations. (*P*) Pulse-chase bolus with labeled external acetate. Transported labeled acetate (*X*_125_) and cytoplasmic acetate (*X*_38_).
(TIF)

Table S1 **Condensed literature information for the SL-E metabolites. Included time dependent metabolites and time independent non-enzymatic parameters.**
(PDF)

Table S2 **Condensed literature information for the SL-E enzymes.**
(PDF)

Table S3 **Incoming and outgoing fluxes at model nodes.**
(PDF)

Table S4 **Eigenvalues for the flux balanced SL-E S-system model.**
(PDF)

Table S5 **Global sensitivity statistics for the SL-E model.**
(PDF)

Table S6 **SL-E logarithmic gains (of metabolites) with magnitudes greater than one.** First row and column correspond to dependent and time independent variables, respectively. The key for the variable names are given in *Tables S1 and S2*. Rows not presented have only values smaller than one.
(PDF)

Table S7 **SL-E rate constant sensitivities (of metabolites) with magnitudes greater than one.** First row and column correspond to dependent variables and rate constants respectively. The key for the variable names is given in *Tables S1 and S2*. Rows not present have only values smaller than one.
(PDF)

Table S8 **SL-E logarithmic gains (of fluxes) with magnitudes greater than one.** First row and column correspond to the fluxes and the independent variables respectively. The key for the variable names is given in *Tables S1 and S2*. Rows not presented have only values smaller than one.
(PDF)

Table S9 **SL-E rate constant sensitivities (of fluxes) with magnitudes greater than one.** First row and column correspond to dependent variables and rate constants, respectively. The key for the variable names is given in *Tables S1 and S2*. Rows not presented have only values smaller than one.
(PDF)

Table S10 **Simulation of the effect of statins in the SL-E model.** The total mass of ergosterol (Erg.) and complex sphingolipid (CS) sub-populations in the flux balanced SL-E model is presented under different experimental conditions, after 120 minutes. Wild type condition is represented by the basal values; the two experimental conditions correspond to: *A*) fold changes with respect to their corresponding basal values after decreasing the specific activity of Thiolase/Synthase and HMG Reductase to 10%, 1%, and 0.01%. *B*) Same conditions as in *A* but with an simultaneous decrease to 1% the activity of IPC Synthase (*X*_133_). Complex sphingolipids: IPC, MIPC, and M(IP)_2_C in Golgi compartment (Golgi CS), or plasma membrane (PM). Last row correspond at the result of divide the DIM ergosterol, and the PM CS's.
(PDF)

Table S11 **Variables equivalence from Plas** [**48**] **into Matlab®** [**49**] **.**
(PDF)

Equations S1 **GMA mass equations for pools of total masses.** In these equations, a superscript indicates fluxes for the complex sphingolipids that were not represented in Figs. 1 and 2 of the manuscript due to lack of space.
(PDF)

Equations S2 **Power-Law terms from** ***Equations S1*** **.** In these equations, a superscript indicates fluxes for the complex sphingolipids that were not represented in Figs. 1 and 2 of the manuscript due to lack of space.
(PDF)

Equations S3
**GMA mass balance equations for labeled pools.** The structures of the mass balance equations are similar to the Equations in S8, but for clarity we use 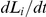 instead of 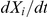 and 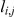 instead of 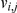.(PDF)

Equations S4 **Terms from** ***Equations S3*** **.** A superscript indicates deviations from the total pool; the method automatically computes equations for the labeled fractions.
(PDF)

Equations S5
**GMA mass balance equations for unlabeled pools.** The structures of the mass balance equations are similar to the Equations in S8, but for clarity we use 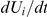 instead of 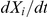 and 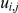 instead of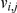.(PDF)

Equations S6 **Terms from** ***Equations S5*** **.**
(PDF)

Material S1 **Mathematical Framework.**
(DOC)

Materials S2 **Modifications of our former sphingolipid (SL) model.**
(DOC)

Material S3 **SL-E GMA model in Plas** [**48**] **format.** File used for the dynamic simulations.
(DOC)

Material S4 **SL-E flux balanced GMA and Flux aggregated model in Plas** [**48**] **format. **Files used for total mass experiments and sensitivity analysis.
(DOC)

Material S5 **SL-E GMA model in Matlab®** [**49**] **format. **Files used for the dynamic simulations.
(DOC)

Materials S6 **SL-E flux balanced GMA model in Matlab®** [**49**] **format. **Files used for total mass experiments.
(DOC)
