# Supplementary figures and images for: Mathematical Modeling and Validation of the Ergosterol Pathway in Saccharomyces cerevisiae
Source: PLoS One. 2011 Dec 14;6(12):e28344. doi: 10.1371/journal.pone.0028344 (PMC3237449; doi:10.1371/journal.pone.0028344)

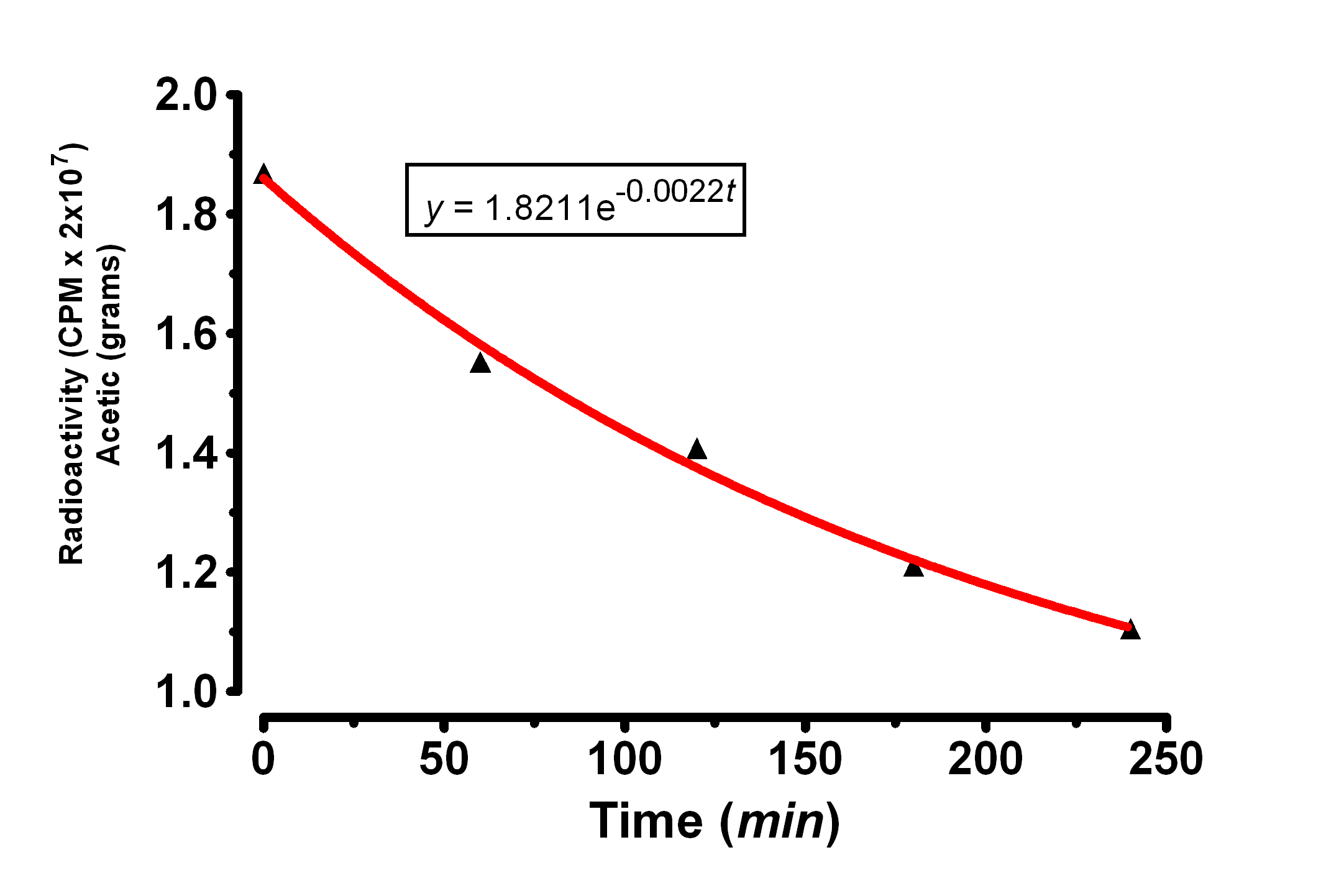

Supplement: Figure S1 — Experimentally observed exponential decay trend line for external radioactive acetate. Experimental points (triangles) data adapted from Wang et al. (see Fig. 2 in ref [121]). For all simulations presented in this manuscript, 100% of the acetate in the medium (X 125) was replaced with radioactive acetate (L 125). (TIF) [file pone.0028344.s001.tif]

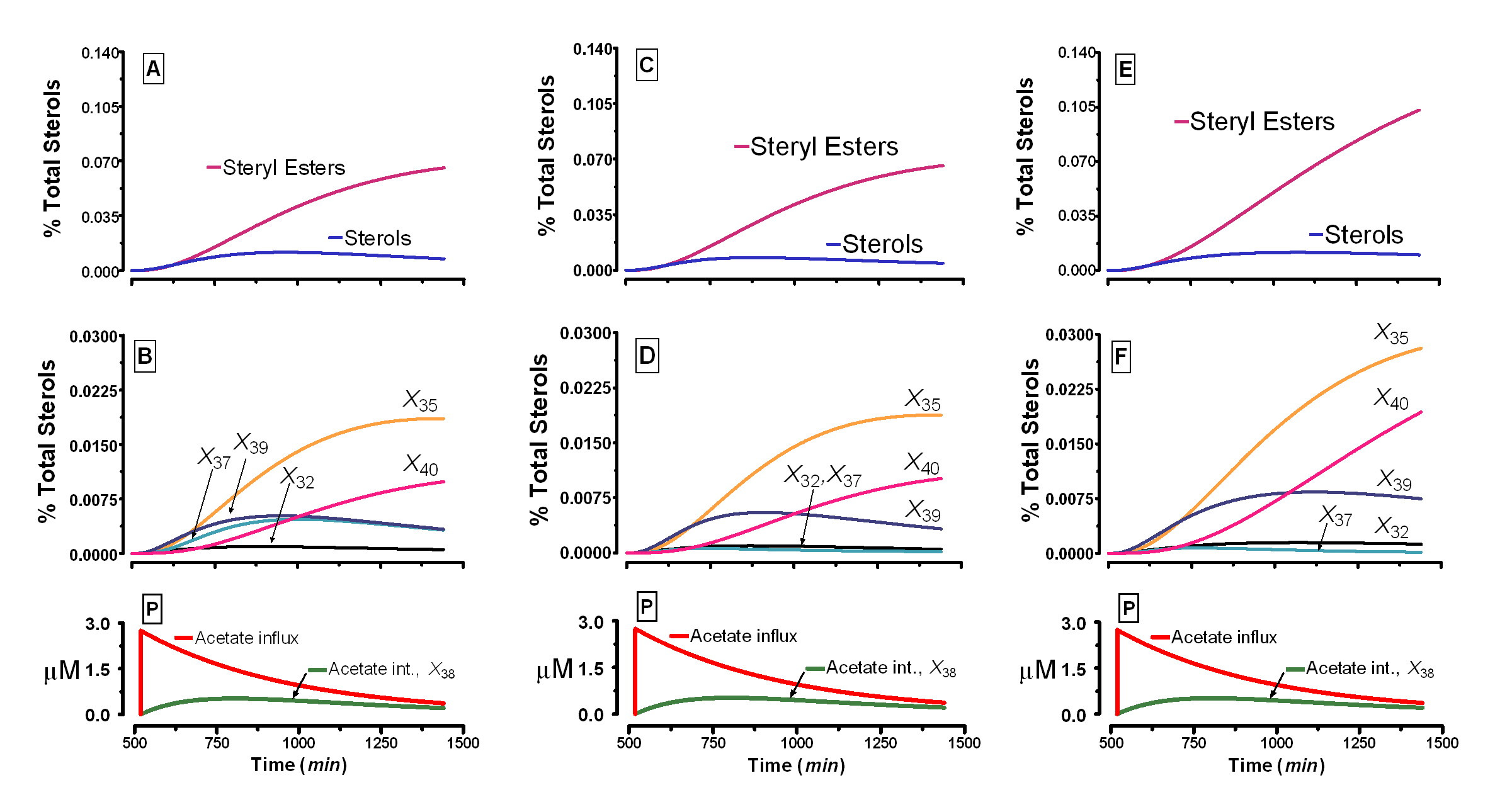

Supplement: Figure S2 — SL-E model dynamics after external radioactive acetate pulse bolus perturbation. Perturbation similar to the one in Fig. 3C. Pools: Steryl-esters (X 33+X 34+X 35+X 40), Sterol (X 30+X 31+X 32+X 36+X 37+X 39). Ergosterol sub-populations: ergosterol in endoplasmic reticulum (X 32), ergosterol steryl-ester-1 (X 35), plasma membrane ergosterol in outer leaflet (X 36), ergosterol associated with the complex sphingolipids (X 37), plasma membrane ergosterol in inner leaflet (X 39), ergosterol steryl-ester-2 (X 40). (A) Wild-type condition. Dynamic simulation for steryl-esters pool and total sterols after pulse-chase bolus with labeled acetate. (B) Wild-type ergosterol and steryl-ester sub-populations. (C) Decrease to 1% in IPC synthase activity (X 133). Dynamic simulation for the steryl-esters pool and total sterols after pulse-chase bolus with labeled acetate. (D) Decrease to 1% in IPC synthase activity (X 133). Ergosterol and steryl-ester sub-populations. (E) Decrease to 1% in serine palmitoyl transferase activity (X 157). Dynamic simulation for steryl-ester pool and total sterols after pulse-chase bolus with labeled acetate. (F) Decrease to 1% in serine palmitoyl transferase activity (X 157). Ergosterol and steryl-ester sub-populations. (P) Pulse-chase bolus with labeled external acetate. Transported labeled acetate (X 125) and cytoplasmic acetate (X 38). (TIF) [file pone.0028344.s002.tif]
