# Supplementary material for: Mathematical Modeling and Validation of the Ergosterol Pathway in Saccharomyces cerevisiae
Source: PLoS One. 2011 Dec 14;6(12):e28344. doi: 10.1371/journal.pone.0028344 (PMC3237449; doi:10.1371/journal.pone.0028344)
Supplement: Table S1 — Condensed literature information for the SL-E metabolites. Included time dependent metabolites and time independent non-enzymatic parameters. (PDF) [file pone.0028344.s003.pdf]

Table S1. *Condensed literature information for the SL-E metabolites.*

**Metabolite**	**Symbol in Model**	**Concentration** (*)	**Comments**	**References**
3-Keto-Dihydrosphingosine (KDHS)	X_1	0.0053 mol%	Low level expected because it is difficult to measure levels for this metabolite by thin layer chromatography	[1], ([2], p. 30693)
Dihydrosphingosine (DHS)	X_2	**0.01 mol%** (Φ)	Ten times the DHS-P concentration ([3]). Data obtained according to the procedures for mass and species measurements in [4]	[4]
		0.53 mol%	Exponential growth phase (1×10^7 cells/ml)	([5], Table II)
Dihydroceramide (Dihydro-C)	X_3	**0.036 mol%**		[6]
		0.16 mol%	Lag phase	[7]
Dihydrosphingosine -1P (DHS-P)	X_4	**0.001 mol%**		([8], Fig. 3C)
		0.00278 mol%	Single measure for both S-1-P species	([9], Table 2)
Phytosphingosine (PHS)	X_5	**0.05 mol%**	Ten times the PHS-P concentration ([3]). Data obtained according to the procedures for mass and species measurements in [4]	[4]
		0.16 mol%	Exponential growth phase (1×10^7 cells/ml)	([5], Table II)
Phytosphingosine-1P (PHS-P)	X_6	**0.005 mol%**		([8], Fig.3C)
		0.00278 mol%	Single measurement for both S-1-P species	([9], Table 2)
Phytoceramide (Phyto-C)	X_7	**0.052 mol%**		[6]
		0.086 mol%	Lag phase	[7]
		4.5 mol%	2.5 A_{600} that correspond to $2.5\text{-}5 \times 10^7$ cells	([10], Fig 8B)
Inositol Phosphorylceramide (IPC-g)	X_8	**0.102 mol%**	Value from [11] at 30°C and 2×10^7 cells/ml. Estimated as 10% of the non-plasma membrane concentration	([11], Fig 7A)
		8.4 mol%	Sum of IPC/C and IPC/D at 24°C	([10], Fig 8B)

CDP- Diacylglycerol (CDP-DAG)	X_9	5.4 mol%	Complete synthetic medium	([12], Table III)
Phosphatidylserine (PS)	X_{10}	**8.4 mol%**	Microsomes	([13], Table 2)
		9.8 mol%	Complete synthetic medium	([12], Table III)
Phosphatidic Acid (PA)	X_{11}	**3 mol%**	Microsomes	([13], Table 2)
		3.3 mol%	Harvested in the Late log phase	([14], Table 3)
		3.1 mol%	Complete synthetic medium	([12], Table III)
Palmitoyl-CoA (Pal-CoA)	X_{12}	**0.01 μM**	Low level for free long-chain acyl-CoA esters	([15], p. 100)
Serine	X_{13}	**2600 μM**		[12]
		2720 μM	Rabbit liver	([16], Table 1)
sn-1,2-Diacylglycerol (DAG)	X_{14}	**10.7 mol%**	Late exponential phase for DAG and for phospholipid concentrations	([17], Fig. 1)
		0.47 mol%	Rat kidney	[18]
Phosphatidylinositol (PI)	X_{15}	**16.7 mol%**	Microsomes	([13], Table 2)
		4.61 mol%	Exponential growth phase (2×10^7 cells/ml)	([11], Fig. 8)
		7.5 mol%	Complete synthetic medium	([12], Table III)
Inositol (I)	X_{16}	**24.1 μM**	Cytosolic concentration	([12], , Table V)
Cytidine diphosphate-Ethanolamine (CDP-Eth)	X_{17}	22 μM	Estimated using the K_M of DG-Ethanolamine phosphotransferase for CDP-Eth	
Mannosylinositol Phosphorylceramide (MIPC-g)	X_{18}	0.14 mol%	Value from [11] at 30°C and 2×10^7 cells/ml. Estimate 10% non-plasma membrane concentration	([11] Fig. 7A)
Mannosyldiinositol Phosphorylceramide ($M(IP)_2C$-g)	X_{19}	**0.0085 mol%**	Value from [11] at 30°C and 2×10^7 cells/ml. Estimate 10% non-plasma membrane concentration	([11], Fig. 7A)
		4.2 mol%	At 24°C, not all the species measured	([10], Fig.8B)
Plasma Membrane Inositol Phosphorylceramide (IPC-m)	X_{20}	0.918 mol%	Value from [11] at 30°C and 2×10^7 cells/ml. Estimated as 90% of the plasma membrane concentration from [19]	([11], Fig. 7A), [19]
Plasma Membrane Mannosylinositol Phosphorylceramide (MIPC-m)	X_{21}	1.26 mol%	Value from [11] at 30°C and 2×10^7 cells/ml. Estimated as 90% of the plasma membrane concentration from [19]	([11], Fig. 7A), [19]

Plasma Membrane Mannosyldinositol Phosphorylceramide ($M(IP)_2C$-m)	X_{22}	0.0765 mol%	Value from [11] at 30°C and 2 × 10^7 cells/ml. Estimated as 90% of the plasma membrane concentration from [19]	([11], Fig. 7A), [19]
Very Long Chain Fatty Acid (C_{26}-CoA)	X_{23}	0.5 mol%		([20], Fig. 2B)
Malonyl-CoA (Mal-CoA)	X_{24}	**182.7 μM**	Ac-CoA multiplied by the relationship between rat liver Mal-CoA and. Ac-CoA, which is 14.5/68.5 according to [21]	[21]
		7.73 mol%	Concentration with respect to long and very long species $C_{22:0}$, $C_{24:0}$ and, $C_{26:0}$	([22], Table VI)
		1740 μM	1:2 Acetyl-CoA: Malonyl-CoA *in vitro* relationship	[23]
Acetyl-CoA (Ac-CoA)	X_{25}	870 μM	Table 2 reported 2.5 mM/gr dw. Converted to μM using RS^d Y_{sx} from Table 4 and "O" from Fig 2	([24], Tables 2 & 4), ([25], Fig. 2)
3-hydroxy-3-methylglutaryl-coenzyme A (HMG-CoA)	X_{26}	0.1 μM	Below detection limit during growth in glucose medium in [26]	[26]
Mevalonate	X_{27}	0.1 μM	Low level; estimated	N/A
Farnesyl-PP	X_{28}	0.1 μM	Low level; estimated. No large changes were found under different experimental conditions in [27] for mouse and rat	[27]
Squalene	X_{29}	0.283 % total sterols	Wild-type Ergosterol / Squalene relationship was expressed as : ER ergosterol × 0.05 % (w/w) /1.67 % (w/w) = 23.7 × 0.05 / 1,67 = 0.712	([28], Fig. 2)
Lanosterol	X_{30}	**1.9 % total sterols** $^{(\Omega)}$	M30 microsomal fraction	([29], Table 3)
		4.0 % total sterols		([30], Table 2)
		3.8 % total sterols		[31]
Zymosterol	X_{31}	**6.4 % total sterols**	M30 microsomal fraction	([29], Table 3)
		12 % total sterols		([30], Table 2)
Ergosterol-ER	X_{32}	**9.51 % total sterols**	PM Ergosterol from [29]; average relationship of 10:1 between the PM and ER from [32] 95.1 / 10 = 9.51	([29], Table 3), ([32], Fig. 4)
		60.2 % total sterols		([29], Table 3)
		43 % total sterols		([30], Table 2)
		77 % total sterols		[31]

Steryl Lanosterol	X_{33}	3.4 % total sterols		([29],Table 3)
Steryl Zymosterol	X_{34}	13.1 % total sterols		([29],Table 3)
Steryl Ergosterol-1	X_{35}	41.13 % total sterols	Assumed as the biggest sub-population with 90% of the total Steryl Ergosterol pool, this yields 45.7 × 0.9 = 41.13 % total sterols	([29],Table 3)
Outer PM Ergosterol	X_{36}	4.755 % total sterols	PM Ergosterol from [29]. multiplied by the PM ergosterol non-DIG associated relationship from [32]. The value is split in half representing the PM outer ergosterol concentration 95.1 × 0.1 × 0.5 = 4.755 % total sterols	([29], Table 3), ([32], Fig. 4)
Outer PM Ergosterol DIM associated (Ergosterol-r)	X_{37}	42.795 % total sterols	PM Ergosterol from [29]. multiplied by the PM ergosterol DIG associated relationship from [32]. The value is split in half representing the PM outer ergosterol concentration: 95.1 × 0.9 × 0.5 = 42.795 % total sterols	([29], Table 3), ([32], Fig. 4)
Internal Acetate (Acetate Int.)	X_{38}	3086 μM	Value at 5 hrs during respiro-fermentative phase	([33], Fig. 2C)
Inner PM Ergosterol (Ergosterol-i)	X_{39}	47.55 % total sterols	PM Ergosterol from [29]. multiplied by 0.5 representing the PM outer ergosterol concentration: 95.1 × 0.5 = 47.55 % total sterols	([29], Table 3), ([32], Fig. 4)
Steryl Ergosterol-2	X_{40}	4.57 % total sterols	Assumed as the smallest sub-population with 10% of the total Steryl Ergosterol pool, this yields 45.7 × 0.1 = 4.57 % total sterols	([29],Table 3)
Pyruvate	X_{124}	227 μM		([34], Fig. 2)
External Acetate (Acetate Ext)	X_{125}	**1250 μM**	Assumed as the rich broth medium acetate concentration used in Taylor and Parks [35]	([33], Fig. 2C)
		0.01 μM	Calculated based on a low external acetate concentration of 1μM under aerobic exponential growth conditions: acetate pK of 4.75, internal pH of 6.75 and external pH of 4	([36], Fig. 6 & Eq. 1)
Adenosine-5’-Triphosphate (ATP)	X_{128}	**1100 μM**		([37], Table II)
		850 μM	Permeabilized yeast cells	([38], Fig. 4)
3-Phosphoserine (3-P-Serine)	X_{137}	**446 μM**	Rabbit liver	([16], Table 1)
Glucose-6-P (G6P)	X_{147}	**1176 μM**	Exponential growth	([34], Fig. 2)
		1000 μM	Permeabilized cells	([38], Fig. 4)
Palmitate	X_{158}	**0.05 μM**	Estimate	
CoA	X_{161}	**60 μM**	Physiological level in rat liver	[39]
		100 μM	*Dictyostelium discoideum*	[40]
Serine Ext.	X_{166}	**4000 μM**		[3]

(Φ) Where the literature reports more than one value reported for the same parameter, the value in bold type is used in the model.

(*) mol% = concentration of sphingoid base or phosphatidate / concentration of total phospholipid.

(†) U/mg = μmol/min/mg.

(Ω) Percent with respect the total sterol amount for the *S. cerevisiae* wild type strain.

A.3.- References.

1. Hannun Y: Unpublished data.
2. Beeler T, Bacikova D, Gable K, Hopkins L, Johnson C, et al. (1998) The Saccharomyces cerevisiae TSC10/YBR265w gene encoding 3-ketosphinganine reductase is identified in a screen for temperature-sensitive suppressors of the Ca2+-sensitive csg2Delta mutant. J Biol Chem 273: 30688-30694.
3. Cowart LA (2010): Personal communication.
4. Jenkins GM, Cowart LA, Signorelli P, Pettus BJ, Chalfant CE, et al. (2002) Acute activation of de novo sphingolipid biosynthesis upon heat shock causes an accumulation of ceramide and subsequent dephosphorylation of SR proteins. J Biol Chem 277: 42572-42578.
5. Wu WI, Lin YP, Wang E, Merrill AH, Jr., Carman GM (1993) Regulation of phosphatidate phosphatase activity from the yeast Saccharomyces cerevisiae by sphingoid bases. J Biol Chem 268: 13830-13837.
6. Mao C Personal communication. pp. Yeast cell labeled with ^{3}H-palmitic acid for 30 or 60 minutes. After labeling, total sphingolipids were extracted and analyzed by TLC, which was autoradiographed. The autoradiography image was scanned and lipids were quantitated by an ImageQuant software.
7. Okamoto Y Personal communication.
8. Skrzypek MS, Nagiec MM, Lester RL, Dickson RC (1999) Analysis of phosphorylated sphingolipid long-chain bases reveals potential roles in heat stress and growth control in Saccharomyces. J Bacteriol 181: 1134-1140.
9. Lanterman MM, Saba JD (1998) Characterization of sphingosine kinase (SK) activity in Saccharomyces cerevisiae and isolation of SK-deficient mutants. Biochem J 332 (Pt 2): 525-531.
10. Reggiori F, Conzelmann A (1998) Biosynthesis of inositol phosphoceramides and remodeling of glycosylphosphatidylinositol anchors in Saccharomyces cerevisiae are mediated by different enzymes. J Biol Chem 273: 30550-30559.
11. Wu WI, McDonough VM, Nickels JT, Jr., Ko J, Fischl AS, et al. (1995) Regulation of lipid biosynthesis in Saccharomyces cerevisiae by fumonisin B1. J Biol Chem 270: 13171-13178.
12. Kelley MJ, Bailis AM, Henry SA, Carman GM (1988) Regulation of phospholipid biosynthesis in Saccharomyces cerevisiae by inositol. Inositol is an inhibitor of phosphatidylserine synthase activity. J Biol Chem 263: 18078-18085.
13. Pichler H, Gaigg B, Hrastnik C, Achleitner G, Kohlwein SD, et al. (2001) A subfraction of the yeast endoplasmic reticulum associates with the plasma membrane and has a high capacity to synthesize lipids. Eur J Biochem 268: 2351-2361.
14. Summers EF, Letts VA, McGraw P, Henry SA (1988) Saccharomyces cerevisiae cho2 mutants are deficient in phospholipid methylation and cross-pathway regulation of inositol synthesis. Genetics 120: 909-922.
15. Knudsen J, Jensen MV, Hansen JK, Faergeman NJ, Neergaard TB, et al. (1999) Role of acylCoA binding protein in acylCoA transport, metabolism and cell signaling. Mol Cell Biochem 192: 95-103.
16. Snell K, Fell DA (1990) Metabolic control analysis of mammalian serine metabolism. Adv Enzyme Regul 30: 13-32.
17. Taylor FR, Parks LW (1979) Triaglycerol metabolism in Saccharomyces cerevisiae. Relation to phospholipid synthesis. Biochim Biophys Acta 575: 204-214.
18. Preiss J, Loomis CR, Bishop WR, Stein R, Niedel JE, et al. (1986) Quantitative measurement of sn-1,2-diacylglycerols present in platelets, hepatocytes, and ras- and sis-transformed normal rat kidney cells. J Biol Chem 261: 8597-8600.

19. Patton JL, Lester RL (1991) The phosphoinositol sphingolipids of Saccharomyces cerevisiae are highly localized in the plasma membrane. J Bacteriol 173: 3101-3108.
20. Gaigg B, Neergaard TB, Schneiter R, Hansen JK, Faergeman NJ, et al. (2001) Depletion of acyl-coenzyme A-binding protein affects sphingolipid synthesis and causes vesicle accumulation and membrane defects in Saccharomyces cerevisiae. Mol Biol Cell 12: 1147-1160.
21. Guynn RW, Veloso D, Veech RL (1972) The concentration of malonyl-coenzyme A and the control of fatty acid synthesis in vivo. J Biol Chem 247: 7325-7331.
22. Choi JY, Martin CE (1999) The Saccharomyces cerevisiae FAT1 gene encodes an acyl-CoA synthetase that is required for maintenance of very long chain fatty acid levels. J Biol Chem 274: 4671-4683.
23. Sumper M, Oesterhelt D, Riepertinger C, Lynen F (1969) [Synthesis of various carboxylic acids by the fatty acid synthetase multienzyme complex of yeast and the explanation for their structure]. Eur J Biochem 10: 377-387.
24. Gombert AK, Moreira dos Santos M, Christensen B, Nielsen J (2001) Network identification and flux quantification in the central metabolism of Saccharomyces cerevisiae under different conditions of glucose repression. J Bacteriol 183: 1441-1451.
25. Ertugay N, Hamamci H, Bayindirli A (1997) Fed-batch cultivation of bakers' yeast: effect of nutrient depletion and heat stress on cell composition. Folia Microbiol (Praha) 42: 214-218.
26. Seker T, Moller K, Nielsen J (2005) Analysis of acyl CoA ester intermediates of the mevalonate pathway in Saccharomyces cerevisiae. Appl Microbiol Biotechnol 67: 119-124.
27. Bruenger E, Rilling HC (1988) Determination of isopentenyl diphosphate and farnesyl diphosphate in tissue samples with a comment on secondary regulation of polyisoprenoid biosynthesis. Anal Biochem 173: 321-327.
28. Polakowski T, Stahl U, Lang C (1998) Overexpression of a cytosolic hydroxymethylglutaryl-CoA reductase leads to squalene accumulation in yeast. Appl Microbiol Biotechnol 49: 66-71.
29. Ott RG, Athenstaedt K, Hrastnik C, Leitner E, Bergler H, et al. (2005) Flux of sterol intermediates in a yeast strain deleted of the lanosterol C-14 demethylase Erg11p. Biochim Biophys Acta 1735: 111-118.
30. Marcireau C, Guilloton M, Karst F (1990) In vivo effects of fenpropimorph on the yeast Saccharomyces cerevisiae and determination of the molecular basis of the antifungal property. Antimicrob Agents Chemother 34: 989-993.
31. Heese-Peck A, Pichler H, Zanolari B, Watanabe R, Daum G, et al. (2002) Multiple functions of sterols in yeast endocytosis. Mol Biol Cell 13: 2664-2680.
32. Baumann NA, Sullivan DP, Ohvo-Rekila H, Simonot C, Pottekat A, et al. (2005) Transport of newly synthesized sterol to the sterol-enriched plasma membrane occurs via nonvesicular equilibration. Biochemistry 44: 5816-5826.
33. Xu Z, Tsurugi K (2006) A potential mechanism of energy-metabolism oscillation in an aerobic chemostat culture of the yeast Saccharomyces cerevisiae. Febs J 273: 1696-1709.
34. Lagunas R, Dominguez C, Busturia A, Saez MJ (1982) Mechanisms of appearance of the Pasteur effect in Saccharomyces cerevisiae: inactivation of sugar transport systems. J Bacteriol 152: 19-25.
35. Taylor FR, Parks LW (1978) Metabolic interconversion of free sterols and steryl esters in Saccharomyces cerevisiae. J Bacteriol 136: 531-537.
36. Keulers M, Satroutdinov AD, Suzuki T, Kuriyama H (1996) Synchronization affector of autonomous short-period-sustained oscillation of Saccharomyces cerevisiae. Yeast 12: 673-682.
37. Gancedo JM, Gancedo C (1973) Concentrations of intermediary metabolites in yeast. Biochimie 55: 205-211.

38. Banuelos M, Gancedo C (1978) In situ study of the glycolytic pathway in Saccharomyces cerevisiae. Arch Microbiol 117: 197-201.
39. Yeh LA, Lee KH, Kim KH (1980) Regulation of rat liver acetyl-CoA carboxylase. Regulation of phosphorylation and inactivation of acetyl-CoA carboxylase by the adenylate energy charge. J Biol Chem 255: 2308-2314.
40. Shiraishi F, Savageau MA (1992) The tricarboxylic acid cycle in Dictyostelium discoideum. I. Formulation of alternative kinetic representations. J Biol Chem 267: 22912-22918.
