# Supplementary material for: Mathematical Modeling and Validation of the Ergosterol Pathway in Saccharomyces cerevisiae
Source: PLoS One. 2011 Dec 14;6(12):e28344. doi: 10.1371/journal.pone.0028344 (PMC3237449; doi:10.1371/journal.pone.0028344)
Supplement: Table S2 — Condensed literature information for the SL-E enzymes. (PDF) [file pone.0028344.s004.pdf]

Table S2. *Condensed literature information for the SL-E enzymes*.

**Enzyme / Transporter**	**Symbol in Model**	**Gene(s)**	**Specific Activity** (†)	**Kinetics**	**Comments**	**Reference**
Pyruvate Decarboxylase (Pyr. Decarboxylase)	X_{122}	*PDC1, PDC5, PDC6*	45 U/mg		The Pyr decarboxylase produces acetaldehyde which is catalyzed by the acetaldehyde dehydrogenase to form Acetate. The Pyr. Decarboxylase is the rate limiting step of the two steps.	[1]
				K_M = 520 μM Pyruvate	*Z. mobilis*	[2]
Pyruvate Dehydrogenase (Pyr. Dehyd.)	X_{123}	*PDA1, PDB1*	0.12 U/mg		Range between 0.12 and 0.16 U/mg	[3]
				K_M = 650 μM Pyruvate		[3]
Phosphatidylinositol Synthase (PI Synthase)	X_{126}	*PIS1*	**2.66 e-3 U/mg** (Φ)		Microsomes.	([4], Table 3)
			8e-4 U/mg		Microsomes	([5], Table I)
				**K_M = 3.25 mol% CDP-DAG, K_M = 210 μM Inositol**	Rat liver CDP-DAG K_M 66 μM from [6]. Converted to mol% according the DHS K_M relationship 0.38 mol% = 7.7 μM from [7] and [8] respectively	([6], Table VI), ([7], Fig. 2), ([8], Fig. 2)
3-Ketodihydrosphingosine Reductase (KDHS reductase)	X_{127}	*TSC10, YBR265w*	**2.62 e-4 U/mg**			([9], Table 5)
				**K_M = 0.74 mol% 3-KDS-SPH**	From [10] K_M for beef liver microsomes is 15 μM. Converted to mol% according the DHS Km relationship 0.38 mol% = 7.7 μM from [7] and [8] respectively	([10], p.367), ([7], Fig. 2), ([8], Fig. 2)
Dihydroceramide Alkaline Ceramidase (Dihydro-CDase)	X_{129}	*YDC1*	**5.4 e-6 U/mg**			([11], Table II)
				**K_M = 0.036 mol% Dihydroceramide**	Estimated value, using dihydroceramide concentration	
				K_M = 3.84 mol% Dihydroceramide, V_{max} = 1.2 U/mg	Rat brain	([12], Table II & p. 27954)

Palmitoyl Transport & Palmitoyl-CoA Synthase (Transp./ Palmitoyl CoA Synthase)	X_{130}	*FAT1, FAA1, FAA4*	**5.08 e-2 U/mg**	Logarithmic phase	Mid-log phase	([13], Table 1)
			3.38 e-3 U/mg		Using oleate as the fatty acid substrate	([14], Table III)
				**K_M = 20 μM Palmitoyl-CoA**, V_{max} = 1.4 e-4 U/mg		([14], Table I)
Phosphoserine-Phosphatase (P-Serine-PPase)	X_{131}	*SER2*	**1.3 e-3 U/mg**			([15], Table 2)
			0.12 U/mg			([16], Fig. 3C)
			0.78 U/mg	K_M = 20 μM 3-Phosphoserine	Human recombinant enzyme	([17], Table I)
				**K_M = 89 μM 3-Phosphoserine**	Rabbit liver	([18], p 17)
Serine Hydroxymethyl Transferase (SHMT)	X_{132}	*SHM2*	**4.5 e-3 U/mg**		Cytosolic reversible enzyme	([19], Table 2)
			8.38e-5 U/mg			([20], Table II)
				**K_M = 650 μM L-Serine**	Cytosolic	([21], p. 11)
				K_M = 700 μM Serine		([22], p. 332)
Inositol Phosphorylceramide Synthase (IPC Synthase)	X_{133}	*AUR1*	**3.3 e-4 U/mg**	K_M = 1.35 mol% Ceramide, **K_M = 5 mol% PI**	Microsomes. PI interact with IPC synthase in a cooperative manner with a Hill constant of 3	([23], Table I)
			2.1e-4 U/mg		Microsomes in mid-exponential growth phase	([24], Table I)
				K_i = 2.89 mol% DHS K_i= 1.96 mol% PHS	Calculated based on IC50 data from [25] , *Vmax* from [24], and the MWC Dimeric model, interaction with effectors from [26]	([25], Fig. 10), [24], [26], [27]
				K_M = 0.5 mM PI, **V_{max} = 6.6e-6 U/mg**	IPC-II and IPC-III	([28], Fig. 4)
Ceramide Synthase (Cer Synthase)	X_{134}	*LAC1, LAG1*	1.65e-5 U/mg			([25], p. 13173)
				**$K_{0.5}$ = 0.27 mol% DHS, $K_{0.5}$ = 0.2 mol% PHS**, V_{max} = 135e-3 U/mg DHS, V_{max} = 105e-3 U/mg PHS		([29], p. 2659)
				K_M = 144 μM DHS, K_M = 299 μM Behenoyl-CoA (C22:0)	Bovine liver mitochondria. From [30] the relation between the DHS and C_{26}-CoA K_M's is approximately 2 and is used for the estimation of the C_{26}-CoA K_M using the DHS K_M from [29].	([30], Table 2), [29]
Mannosyl Inositol Phosphoceramide Synthase (MIPC Synthase)	X_{135}	*SUR1, CSG1*	1.65e-4 U/mg		It is assumed that the activity of MIPC synthase is about half the IPC synthase activity.	([28], Fig. 4)
				K_M = 0.102 mol% IPC	Estimates K_M using IPC concentration	

Sphingoid Base Kinase	X_{136}	*LCB 4/5*	**4e-6 U/mg**	**K_M = 0.38 mol% DHS, K_M = 1.2 mol% PHS, V_{max} = 4.25e-6 U/mg DHS, V_{max} = 6e-6 U/mg PHS**		([7], Figs. 2 & 5)
			0.0045 U/mg	**K_M = 25 μM ATP, K_M = 7.7 μM DHS, K_M = 10 μM PHS**	Pellet results for activity	[8]
Phosphatidylserine Synthase (PS Synthase)	X_{138}	*CHO1*	**3.32 e-3 U/mg**		Microsomes	([4], Table 3)
			4.2e-4 U/mg			([31], Table 3)
				K_M = 830 μM Serine, K_M = 83 μM CDP-DAG, K_i = 65 μM Inositol		([6], Table VI & p. 18084)
				K_i = 1.42 mol% DHS, K_i = 3.55 mol% PHS	Calculated based on IC50 data from [25]. and the MWC Dimeric model, interaction with effectors from [26]	([25], Fig. 11), [24], [27]
				K_i= 7 mol% DAG, K_a = 0.033 mol% PA, **K_a= 3.2 mol% PI**	Kinetic orders for PA and PI obtained from log-log plot (see [27])	[27], ([32], Table I & Fig. 3A)
Phosphatidate Phosphatase (PA-PPase)	X_{139}	*DPP1, LPP1*	**2.4e-3 U/mg**			([33], Table I)
			3 U/mg	K_M = 50 μM PA		([34], Table I)
				**K_M = 2.2 mol% PA, aK_i = 0.4 mol% PHS, aK_i = 0.2 mol% DHS, aK_i= 1.5 mol% Sphingosine**		([35], Table I)
				$A_{0.5}$ = 2.6 CDP-DAG, **$A_{0.5}$ = 5.5 PI**	Kinetic orders for PI and PA obtained from log-log plot (see [27])	[36], [27]
CDP-Diacylglycerol Synthase (CDP-DAG Synthase)	X_{140}	*CDS1*	6.1e-4 U/mg	K_M = 500 μM PA, K_M = 1 mM CTP, V_{max} = 4.7 U/mg		([37], Table 1)
Sphingoid-1-phosphate Phosphatase (SB-PPase)	X_{141}	*LCB3, YSR2*	8e-4 U/mg			([38], Fig. 3A)
				K_M = 3.11e-2 mol% (16 μM) DHS-P and PHS-P, V_{max}= 2.77e-5 μM	Rat liver microsomes. K_M and V_{max} converted to mol% with the relationship suggested by [7]: 3.5 mol% = 1.8 mM	[7], ([39], Fig. 10 and p. 382)
DG-Choline Phosphotransferase (ChoPT)	X_{142}	*CPT1*	6.6e-4 U/mg	K_M = 8 mol% Dioleoylglycerol		([40], Table I and Fig. 3)
GPI Remodelase (Remodeling)	X_{143}		1e-4 U/mg	K_M = 0.052 mol%	K_M estimation using phytoceramide concentration. Activity with order magnitude similar at IPC synthase	
Phosphoinositide Kinase (PI Kinase)	X_{144}	*VPS34*	1.72 e-3 U/mg	K_M = 70 μM PI, K_M = 300 μM MgATP, **V_{max} = 4.75 U/mg**	Microsomes	([41], Table I and p. 18902)
DG-Ethanolamine Phosphotransferase (EthPT)	X_{145}	*EPT1*	1 e-3 U/mg	K_M = 22 μM CDP-Eth, K_M = 3.3 mol% Dioleoylglycerol		([40], Table 1 and Fig. 2)

Inositol-1-P Synthase (I-1-P Synth.)	X_{146}	*INO1*	**8.33 e-4 U/mg**	K_M = 1180 µM Glucose-6-P, K_M = 8 µM NAD, V_{max} = 167 nmol/h		([42], Table 1 and p. 7082)
			3 e-4 U/mg		Two hr after inositol was added to the medium	([43], Fig. 8)
Acyl-CoA-Binding Protein (ACBP)	X_{148}	*ACB1*	20 µM	K_D = 5.5 e-5 µM	Within the range 10 to 50 µM	([44], Fig. 2)
Glycerol-3-Phosphate Acyltransferase (G3P Acyltranferase)	X_{149}	*GAT1, GAT2, SCT1*	**3.94 e-3 U/mg**		Microsomes 30,000 × *g*	([45], Table 1)
			1.04 e-3 U/mg		Late logarithmic phase	([46], Fig. 2)
				**K_M =15 µM Pal-CoA**, V_{max} =1. 53e-3 U/mg	K_M from curve S/Act vs. S	([47], Fig. 2B)
					PS activator effect calculated in [27] , based on data from [48]	[27], ([48], Fig. 5)
				K_M = 120 µM Glycerol-P, K_M = 4.6 µM Palmitoyl-CoA	*Escherichia coli*	([49], p 5)
Sphingosine-Phosphate Lyase (Lyase)	X_{150}	*DPL1*	3.67e-5 U/mg		Average from different organisms	([50], Table II)
				K_M = 0.024 mol% (12.5 µM) DHS-P and PHS-P	K_M from rat liver. Converted to mol% according to [7] 3.5 mol% = 1.8 mM	([51], p. 247), [7]
Inositol Phosphosphingolipid Phospholipase C (IPCase)	X_{151}, X_{164}	*ISC1*	1.5e-4 U/mg	K_M = 3.57 mol% IPC, K_M = 1.85 mol% MIPC, K_M = 1.92 mol% M(IP)$_2$C	X_{151} and X_{164} correspond to IPCase with Phyto-C and Dihydro-C as products respectively	([52], Tables I & II)
Fatty Acid Synthetase (FAS)	X_{152}	*FAS1, FAS2, FAS3*	**8.9 e-3 U/mg**	K_M = 8 µM Mal-CoA, **K_M = 28 µM Ac-CoA**		([53], Table I & p. 22)
			8.4e-3 U/mg			([54], Table 2)
			3.124 e-3 U/mg			([55], Table I)
			0.065 U/mg			([56], Table 1)
				K_M = 6 -19 µM Ac-CoA	*Cryptococcus neoformans*	([57], Table 2)
Phytoceramide Alkaline Ceramidase (Phyto-CDase)	X_{153}	*YPC1*	1.98e-5 U/mg			([11], Table II)
				**K_M = 0.052 mol% (*) Phytoceramide**	Assumed similar to phytoceramide concentration	
				K_M = 1.29 mol% Ceramide	Rat brain	([12], Fig. 7A)
4-Hydroxylase (Hydroxilase) (*SYR2p – SUR2p*)	X_{154}	*SYR2 SUR2*	1.7e-4 U/mg		Microsomes	([58] , Table II)
				K_M = 0.01 mol% DHS, K_M = 0.036 mol% Dihydro-C	Assumed similar to substrate concentration	
Mannosyldiinositol Phosphorylceramide Synthase (M(IP)$_2$C Synthase)	X_{155}	*IPT1*	**8.25e-5 U/mg**		Activity of M(IP)$_2$C synthase assumed to be about 1/4 the IPC synthase activity	([28], Fig. 4)
				K_M = 0.14 mol% MIPC, K_M = 5 mol% PI	Estimation of K_M using MIPC-g concentration. K_M of the M(IP)$_2$C Synthase for PI assumed equal than for IPC Synthase	([23], Table I)

Phosphatidylserine Decarboxilase (PS Decarboxylase)	X_{156}	*PSD2*	**1.06e-5 U/mg**		*PSD2p* value for non-mitochondrial enzyme	([59], Table I)
				**K_M = 8.4 mol%**	K_M assumed similar with the substrate concentration	
				K_M = 24 μM PS	*E. coli*	([60], Fig. 3 and p. 3081)
				K_M = 9.5 μM PS		([61], p. 6064)
Serine Palmitoyltransferase (SPT)	X_{157}	*LCB1*/2, *SCS1*	**1.06e -4 U/mg**	**K_M = 4000 μM Serine, K_M = 15 μM Palmitoyl-CoA,** V_{max} = 1.03 e-4 U/mg	Fig 3A K_M for Palmitoyl-CoA required correction; utilized the original data of velocity of Pal-CoA vs. SPT	([9], Table 3 and Fig. 3A)
			6.31e -5 U/mg	K_M = 670 μM Serine	Rat liver	([62], Table I)
Very Long Chain Fatty Acid Synthase (ELO1p)	X_{159}	*ELO1*	6e-4 U/mg	K_M = 130 μM Mal-CoA, K_M = 130 μM Pal-CoA		[63]
Acetyl-Coenzyme A Carboxylase (ACCp)	X_{160}	*ACC*	**2.2 e-2 U/mg**	**K_M = 16 μM Ac-CoA, K_M = 660 μM ATP**		([64], p. 7)
			0.009 U/mg	**K_i= 6.5e-3 μM Pal-CoA, $C_{16:}$0, Ki= 4.8e-2 μM** Arachidonoyl-CoA, $C_{20:0}$, K_M = 25 μM Ac-CoA, K_M = 15 μM ATP	Rat liver	([65], Tables I & II, and p. 12)
				K_i = 5.5e-3 μM Pal-CoA and C_{26}-CoA	Liver	([66], Table 2)
				K_M = 19 μM Ac-CoA, K_i = 7.2 μM Palmityl-CoA, K_i = 1.3 μM Oley-CoA	Rat liver	([67], Table 4)
			0.029 U/mg			([68], Table II)
			0.042 U/mg			([54], Table 2)
			2.5 e-3 U/mg			([56], Table 1)
				K_M = 260 μM Ac-CoA	*Candida lipolytica*	([69], p. 42)

Acetyl-Coenzyme A Synthetase (ACSp)	X_{163}	*ACS1, ACS2*	**0.73 U/mg**	**K_M = 208 µM Acetate, K_M = 238 µM CoA**	Activity for the microsomal fraction. Kinetic order for the Pal-CoA inhibition was obtained from ([70], Fig 1) log-log plot.	([70], Table 2 and Fig. 1)
			0.22 U/mg	**K_M = 1100 µM ATP,** K_M = 600 µM Acetate, V_{max} = 1.2		([71], Table I)
			0.66 U/mg	K_M = 35 µM CoA, K_M = 280 µM Acetate, K_M = 1200 µM ATP		([72], Table IV)
			0.025 U/mg			([73], Table II)
Inositol Phosphosphingolipid Phospholipase C (IPCase)	X_{164}	*ISC1*			See X_{151}	
Serine Transport	X_{165}		**4.8 e-2 U/mg**			([74], Table 3)
			0.011 U/mg		Yeast cells *rho*+ with functional mitochondria	([75], Table I)
				K_M = 166 µM Serine	Yeast cells *rho*+ with functional mitochondria	[75]
Phospholipase B	X_{168}	*PLB3*	5 e-5 U/mg		Estimated low value	
				K_M = 75 mol% PI		([76], Fig. 4B)
Acetoacetyl-CoA thiolase / HMG-CoA synthase (Thiolase/Synthase)	X_{171}	*ERG10, ERG13*	0.14 U/mg			([77], Table 3)
				K_M = 380 µM	*Rhizobium* sp.	[78]
HMG-CoA Reductase (Reductase)	X_{172}	*HMG1, HMG2*	5.5e-3 U/mg			([77], Table 3)
				K_M = 45 µM HMG-CoA	*Sulfolobus solfataricus*	([79], Table 3)
Mevalonate Kinase (Kinase)	X_{173}	*ERG12*	6 e-4 U/mg		From *ERG9* mutants. No activity detectable in wild type strains; estimated by dividing the value by 100	([80], Table 1)
				K_M = 41 µM Mevalonate	*Staphylococcus aureus*	[81]
Squalene Synthase	X_{174}	*ERG9*	**4.7e-4 U/mg**		Sum of radioactivity in squalene epoxidase and lanosterol fractions	[82]
			1.48e-5 U/mg		30 K *g* microsomes. Aerobic, late exp. growth.	([4], Table 4)
					Kinetic order for Farnesyl-PP obtained from log-log plot of concentration vs. enzymatic rate.	([83], Fig. 4a)
Squalene Epoxidase	X_{175}	*ERG1*	**1e-4 U/mg**		Sum of radioactivity in squalene epoxidase and lanosterol fractions	[82]
			5.8e-6 U/mg		30 K *g* microsomes. Aerobic, late exp. growth.	([4], Table 4)
				K_M = 28 µM Squalene	*Leptosphaeria nodorum*. Data not used because different units between substrate and K_M	[84]
					Kinetic order for Squalene estimated from log-log plot of concentration vs. enzymatic rate.	([85], Fig. 1A)

Lanosterol C-14 Demethylase	X_{176}	*ERG11*	2.65e-8 U/mg		525 pmol of 3-hydroxy-benzo[*a*]pyrene/ nmol of P-450/ hr from [86] transformed to U/mg based in the 3 pmol of cytocorme P-450/mg of yeast microsomal protein data from [87]. 525 × 3/ (1e9 × 60) = 2.65e-8	([86], p. 1035), [87]
				K_M = 13.5 μM Squalene	Data not used because different units between substrate and K_M	[88]
					Kinetic order for the Lanosterol estimated from log-log plot of concentration vs. enzymatic rate.	([89], Fig. 3)
delta 24-sterol methyltransferase	X_{177}	*ERG6*	4e-6 U/mg			([90], Table I)
			7.91e-4 U/mg			([91], Table I)
			**1 e-4 U/mg**		For microsomes (40K x *g*). General Steryl synthase activity. Mid-exponential growth.	([92], Table 3)
				K_M = 15 μM Zymosterol	Data not used because different units between substrate and K_M	[93]
				K_M = 6.2e-11 μM		[91]
					Kinetic order for Zymosterol estimated from log-log plot of concentration vs. enzymatic rate.	([91], Fig. 5)
Farnesyltransferase (GGPP)	X_{179}	*BTS1*	4 e-3 U/mg		Specific activity assumed similar to that of Farnesyl diphosphate synthetase.	([94], Table I)
				K_M = 43 μM isopentenyl pyrophosphate (IPP)	Condensation reaction of IPP with Farnesyl-PP	([95], Table 1)
				**K_M = 0.1 μM Farnesyl-PP**	Assumed equal to the farnesyl-PP concentration	
Steryl Ester Hydrolase	X_{180}	*YEH1*	**1.1 e-4 U/mg**		Microsomes 40k	([92], Table 3).
			9.975e-7 U/mg		Lipid particles. Tgl1p and Yeh1p are localized in Lipid Particles [96].	([97], Fig. 6B)
			1.56e-6 U/mg		Not for an specific protein	([98], Table I)
				K_M = 143 μM Cholesteryl oleate	Data not used because different units between substrate and K_M	[99]
					Kinetic order for Steryl Lanosterol, Steryl Zymosterol, and Steryl Ergosterol-1,2 estimated from log-log plot of concentration vs. enzymatic rate.	([100], Fig. 4)
Steryl Ester Synthase	X_{181}	*ARE1*	0.11 U/mg		Specifically for *ARE1, alias SAT2*. Mid-log phase.	([101], Figs. 2A,B)
			**2.54e-3 U/mg**		For microsomes (40K x *g*). General Steryl synthase activity. Mid-exponential growth.	([92], Table 3)
				K_M = 60 μM Cholesterol	Kinetic order for Lanosterol and Zymosterol estimated from log-log plot of Cholesterol concentration vs. enzymatic rate.	([102], Fig. 1a)

Steryl Ester Hydrolase	X_{182}	*YEH2*	**9.975e-7 U/mg**		Plasma membrane activity	([97], Fig. 6B)
			1.1e-7 U/mg		For microsomes (40K x *g*). General Steryl synthase activity. Mid-exponential growth.	([92], Table 3)
			1.8e-7 U/mg		30 K *g* microsomes. Aerobic, late exp. growth.	([4], Table 4)
				K_M = 0.121 μM/ml cholesteryl oleate	According to [96],Yeh2p appears to utilize SE's from LP as substrate despite the uncertain localization of the enzyme in the plasma membrane	[100], [96]
					Kinetic order for Steryl Ergosterol-2 estimated from log-log plot of concentration vs. enzymatic rate.	([100], Fig. 4)
Steryl Ester Synthase	X_{183}	*ARE2*	**2.54e-3 U/mg**		For microsomes (40K x *g*).	([92], Table 3)
			3.6e-2 U/mg		Specifically for *ARE2, alias SAT1*. Mid-log growing phase	([101], Figs. 2A,B)
			4.1e-7 U/mg		30 K *g* microsomes. Aerobic, late exp. growth.	([4], Table 4)
			1 U/mg		Not for an specific protein	([98], Table I)
				K_M = 60 μM Cholesterol	Kinetic order for Ergosterol estimated from log-log plot of Cholesterol concentration vs. enzymatic rate.	([102], Fig. 1a)
Proteins associated with the ergosterol flux from the ER to others organelles	X_{186}		1e-3 U/mg		Estimate	

(†) U/mg = μmol/min/mg.

(Φ) Parameter values in bold are used in the model.

(*) mol% = concentration of sphingoid base or phosphatidate / concentration of total phospholipid.

References.

1. Sergienko EA, Jordan F (2001) Catalytic acid-base groups in yeast pyruvate decarboxylase. 2. Insights into the specific roles of D28 and E477 from the rates and stereospecificity of formation of carboligase side products. Biochemistry 40: 7369-7381.
2. Candy JM, Duggleby RG (1998) Structure and properties of pyruvate decarboxylase and site-directed mutagenesis of the Zymomonas mobilis enzyme. Biochim Biophys Acta 1385: 323-338.
3. Kresze GB, Ronft H (1981) Pyruvate dehydrogenase complex from baker's yeast. 1. Purification and some kinetic and regulatory properties. Eur J Biochem 119: 573-579.
4. Pichler H, Gaigg B, Hrastnik C, Achleitner G, Kohlwein SD, et al. (2001) A subfraction of the yeast endoplasmic reticulum associates with the plasma membrane and has a high capacity to synthesize lipids. Eur J Biochem 268: 2351-2361.
5. Fischl AS, Carman GM (1983) Phosphatidylinositol biosynthesis in Saccharomyces cerevisiae: purification and properties of microsome-associated phosphatidylinositol synthase. J Bacteriol 154: 304-311.
6. Kelley MJ, Bailis AM, Henry SA, Carman GM (1988) Regulation of phospholipid biosynthesis in Saccharomyces cerevisiae by inositol. Inositol is an inhibitor of phosphatidylserine synthase activity. J Biol Chem 263: 18078-18085.
7. Lanterman MM, Saba JD (1998) Characterization of sphingosine kinase (SK) activity in Saccharomyces cerevisiae and isolation of SK-deficient mutants. Biochem J 332 (Pt 2): 525-531.
8. Nagiec MM, Skrzypek M, Nagiec EE, Lester RL, Dickson RC (1998) The LCB4 (YOR171c) and LCB5 (YLR260w) genes of Saccharomyces encode sphingoid long chain base kinases. J Biol Chem 273: 19437-19442.
9. Pinto WJ, Wells GW, Lester RL (1992) Characterization of enzymatic synthesis of sphingolipid long-chain bases in Saccharomyces cerevisiae: mutant strains exhibiting long-chain-base auxotrophy are deficient in serine palmitoyltransferase activity. J Bacteriol 174: 2575-2581.
10. Kishimoto Y (1983) In: Boyer. PD, editor. The Enzymes. 3 ed. New York,: Academic Press. pp. Ch 10.
11. Mao C, Xu R, Bielawska A, Szulc ZM, Obeid LM (2000) Cloning and characterization of a Saccharomyces cerevisiae alkaline ceramidase with specificity for dihydroceramide. J Biol Chem 275: 31369-31378.
12. El Bawab S, Bielawska A, Hannun YA (1999) Purification and characterization of a membrane-bound nonlysosomal ceramidase from rat brain. J Biol Chem 274: 27948-27955.
13. Kamiryo T, Parthasarathy S, Numa S (1976) Evidence that acyl coenzyme A synthetase activity is required for repression of yeast acetyl coenzyme A carboxylase by exogenous fatty acids. Proc Natl Acad Sci U S A 73: 386-390.
14. Knoll LJ, Johnson DR, Gordon JI (1994) Biochemical studies of three Saccharomyces cerevisiae acyl-CoA synthetases, Faa1p, Faa2p, and Faa3p. J Biol Chem 269: 16348-16356.
15. Melcher K, Entian KD (1992) Genetic analysis of serine biosynthesis and glucose repression in yeast. Curr Genet 21: 295-300.
16. Albers E, Laize V, Blomberg A, Hohmann S, Gustafsson L (2003) Ser3p (Yer081wp) and Ser33p (Yil074cp) are phosphoglycerate dehydrogenases in Saccharomyces cerevisiae. J Biol Chem 278: 10264-10272.
17. Collet JF, Stroobant V, Van Schaftingen E (1999) Mechanistic studies of phosphoserine phosphatase, an enzyme related to P-type ATPases. J Biol Chem 274: 33985-33990.
18. Snell K, Fell DA (1990) Metabolic control analysis of mammalian serine metabolism. Adv Enzyme Regul 30: 13-32.

19. Kastanos EK, Woldman YY, Appling DR (1997) Role of mitochondrial and cytoplasmic serine hydroxymethyltransferase isozymes in de novo purine synthesis in Saccharomyces cerevisiae. Biochemistry 36: 14956-14964.
20. McNeil JB, McIntosh EM, Taylor BV, Zhang FR, Tang S, et al. (1994) Cloning and molecular characterization of three genes, including two genes encoding serine hydroxymethyltransferases, whose inactivation is required to render yeast auxotrophic for glycine. J Biol Chem 269: 9155-9165.
21. Zelikson R, Luzzati M (1976) Two forms of serine transhydroxymethylase, one absent in a thymidylate-less mutant in Saccharomyces cerevisiae. Eur J Biochem 64: 7-13.
22. Nakamura KD, Trewyn RW, Parks LW (1973) Purification and characterization of serine transhydroxy-methylase from Saccharomyces cerevisiae. Biochim Biophys Acta 327: 328-335.
23. Fischl AS, Liu Y, Browdy A, Cremesti AE (2000) Inositolphosphoryl ceramide synthase from yeast. Methods Enzymol 311: 123-130.
24. Ko J, Cheah S, Fischl AS (1994) Regulation of phosphatidylinositol:ceramide phosphoinositol transferase in Saccharomyces cerevisiae. J Bacteriol 176: 5181-5183.
25. Wu WI, McDonough VM, Nickels JT, Jr., Ko J, Fischl AS, et al. (1995) Regulation of lipid biosynthesis in Saccharomyces cerevisiae by fumonisin B1. J Biol Chem 270: 13171-13178.
26. Hayashi K, Sakamoto N (1985) Dynamic Analysis of Enzyme Systems: An Introduction. Springer-Verlag, Japan Scientific Societies Press Tokyo.
27. Alvarez-Vasquez F, Sims KJ, Hannun YA, Voit EO (2004) Integration of kinetic information on yeast sphingolipid metabolism in dynamical pathway models. J Theor Biol 226: 265-291.
28. Becker GW, Lester RL (1980) Biosynthesis of phosphoinositol-containing sphingolipids from phosphatidylinositol by a membrane preparation from Saccharomyces cerevisiae. J Bacteriol 142: 747-754.
29. Guillas I, Kirchman PA, Chuard R, Pfefferli M, Jiang JC, et al. (2001) C26-CoA-dependent ceramide synthesis of Saccharomyces cerevisiae is operated by Lag1p and Lac1p. Embo J 20: 2655-2665.
30. Shimeno H, Soeda S, Sakamoto M, Kouchi T, Kowakame T, et al. (1998) Partial purification and characterization of sphingosine N-acyltransferase (ceramide synthase) from bovine liver mitochondrion-rich fraction. Lipids 33: 601-605.
31. Homann MJ, Bailis AM, Henry SA, Carman GM (1987) Coordinate regulation of phospholipid biosynthesis by serine in Saccharomyces cerevisiae. J Bacteriol 169: 3276-3280.
32. Bae-Lee M, Carman GM (1990) Regulation of yeast phosphatidylserine synthase and phosphatidylinositol synthase activities by phospholipids in Triton X-100/phospholipid mixed micelles. J Biol Chem 265: 7221-7226.
33. Hosaka K, Yamashita S (1984) Partial purification and properties of phosphatidate phosphatase in Saccharomyces cerevisiae. Biochim Biophys Acta 796: 102-109.
34. Lin YP, Carman GM (1989) Purification and characterization of phosphatidate phosphatase from Saccharomyces cerevisiae. J Biol Chem 264: 8641-8645.
35. Wu WI, Lin YP, Wang E, Merrill AH, Jr., Carman GM (1993) Regulation of phosphatidate phosphatase activity from the yeast Saccharomyces cerevisiae by sphingoid bases. J Biol Chem 268: 13830-13837.
36. Wu WI, Carman GM (1996) Regulation of phosphatidate phosphatase activity from the yeast Saccharomyces cerevisiae by phospholipids. Biochemistry 35: 3790-3796.
37. Kelley MJ, Carman GM (1987) Purification and characterization of CDP-diacylglycerol synthase from Saccharomyces cerevisiae. J Biol Chem 262: 14563-14570.
38. Mandala SM, Thornton R, Tu Z, Kurtz MB, Nickels J, et al. (1998) Sphingoid base 1-phosphate phosphatase: a key regulator of sphingolipid metabolism and stress response. Proc Natl Acad Sci U S A 95: 150-155.

39. Stoffel W, Grol M (1974) Chemistry and biochemistry of 1-desoxysphinganine 1-phosphonate (dihydrosphingosine-1-phosphonate). Chem Phys Lipids 13: 372-388.
40. Hjelmstad RH, Bell RM (1991) sn-1,2-diacylglycerol choline- and ethanolaminephosphotransferases in Saccharomyces cerevisiae. Mixed micellar analysis of the CPT1 and EPT1 gene products. J Biol Chem 266: 4357-4365.
41. Belunis CJ, Bae-Lee M, Kelley MJ, Carman GM (1988) Purification and characterization of phosphatidylinositol kinase from Saccharomyces cerevisiae. J Biol Chem 263: 18897-18903.
42. Donahue TF, Henry SA (1981) myo-Inositol-1-phosphate synthase. Characteristics of the enzyme and identification of its structural gene in yeast. J Biol Chem 256: 7077-7085.
43. Culbertson MR, Donahue TF, Henry SA (1976) Control of inositol biosynthesis in Saccharomyces cerevisiae: properties of a repressible enzyme system in extracts of wild-type (Ino+) cells. J Bacteriol 126: 232-242.
44. Knudsen J, Jensen MV, Hansen JK, Faergeman NJ, Neergaard TB, et al. (1999) Role of acylCoA binding protein in acylCoA transport, metabolism and cell signaling. Mol Cell Biochem 192: 95-103.
45. Athenstaedt K, Weys S, Paltauf F, Daum G (1999) Redundant systems of phosphatidic acid biosynthesis via acylation of glycerol-3-phosphate or dihydroxyacetone phosphate in the yeast Saccharomyces cerevisiae. J Bacteriol 181: 1458-1463.
46. Zheng Z, Zou J (2001) The initial step of the glycerolipid pathway: identification of glycerol 3-phosphate/dihydroxyacetone phosphate dual substrate acyltransferases in Saccharomyces cerevisiae. J Biol Chem 276: 41710-41716.
47. Schlossman DM, Bell RM (1978) Glycerolipid biosynthesis in Saccharomyces cerevisiae: sn-glycerol-3-phosphate and dihydroxyacetone phosphate acyltransferase activities. J Bacteriol 133: 1368-1376.
48. Mishra S, Kamisaka Y (2001) Purification and characterization of thiol-reagent-sensitive glycerol-3-phosphate acyltransferase from the membrane fraction of an oleaginous fungus. Biochem J 355: 315-322.
49. Wilkison WO, Bell RM (1997) sn-Glycerol-3-phosphate acyltransferase from Escherichia coli. Biochim Biophys Acta 1348: 3-9.
50. van Veldhoven PP, Mannaerts GP (1993) Sphingosine-phosphate lyase. Adv Lipid Res 26: 69-98.
51. Van Veldhoven PP (2000) Sphingosine-1-phosphate lyase. Methods Enzymol 311: 244-254.
52. Sawai H, Okamoto Y, Luberto C, Mao C, Bielawska A, et al. (2000) Identification of ISC1 (YER019w) as inositol phosphosphingolipid phospholipase C in Saccharomyces cerevisiae. J Biol Chem 275: 39793-39798.
53. Lynen F (1969) Yeast Fatty Acid Synthase. Methods Enzymology Vol XIV: 17-31.
54. Roggenkamp R, Numa S, Schweizer E (1980) Fatty acid-requiring mutant of Saccharomyces cerevisiae defective in acetyl-CoA carboxylase. Proc Natl Acad Sci U S A 77: 1814-1817.
55. Stoops JK, Wakil SJ (1981) The yeast fatty acid synthetase. Structure-function relationship and the role of the active cysteine-SH and pantetheine-SH. J Biol Chem 256: 8364-8370.
56. Chirala SS (1992) Coordinated regulation and inositol-mediated and fatty acid-mediated repression of fatty acid synthase genes in Saccharomyces cerevisiae. Proc Natl Acad Sci U S A 89: 10232-10236.
57. Mahmoud YA, Abu el Souod SM, Niehaus WG (1996) Purification and characterization of fatty acid synthetase from Cryptococcus neoformans. Mycopathologia 136: 75-84.
58. Grilley MM, Stock SD, Dickson RC, Lester RL, Takemoto JY (1998) Syringomycin action gene SYR2 is essential for sphingolipid 4-hydroxylation in Saccharomyces cerevisiae. J Biol Chem 273: 11062-11068.

59. Trotter PJ, Pedretti J, Yates R, Voelker DR (1995) Phosphatidylserine decarboxylase 2 of Saccharomyces cerevisiae. Cloning and mapping of the gene, heterologous expression, and creation of the null allele. J Biol Chem 270: 6071-6080.
60. Dowhan W, Wickner WT, Kennedy EP (1974) Purification and properties of phosphatidylserine decarboxylase from Escherichia coli. J Biol Chem 249: 3079-3084.
61. Trotter PJ, Voelker DR (1995) Identification of a non-mitochondrial phosphatidylserine decarboxylase activity (PSD2) in the yeast Saccharomyces cerevisiae. J Biol Chem 270: 6062-6070.
62. Williams RD, Wang E, Merrill AH, Jr. (1984) Enzymology of long-chain base synthesis by liver: characterization of serine palmitoyltransferase in rat liver microsomes. Arch Biochem Biophys 228: 282-291.
63. Dittrich F, Zajonc D, Huhne K, Hoja U, Ekici A, et al. (1998) Fatty acid elongation in yeast--biochemical characteristics of the enzyme system and isolation of elongation-defective mutants. Eur J Biochem 252: 477-485.
64. Matsuhashi M (1969) Acetyl-CoA Carboxilase from Yeast. Methods Enzymology XIV: C:3-8.
65. Tanabe T, Nakanishi S, Hashimoto T, Ogiwara H, Nikawa J, et al. (1981) Acetyl-CoA carboxylase from rat liver. Methods Enzymol 71 Pt C: 5-16.
66. Faergeman NJ, Knudsen J (1997) Role of long-chain fatty acyl-CoA esters in the regulation of metabolism and in cell signalling. Biochem J 323 (Pt 1): 1-12.
67. Lynen F, Matsuhashi M, Numa S, Schweizer E (1963) The cellular control of fatty acid synthesis at the enzymatic level. Biochem Soc Symp 24: 43-56.
68. Hasslacher M, Ivessa AS, Paltauf F, Kohlwein SD (1993) Acetyl-CoA carboxylase from yeast is an essential enzyme and is regulated by factors that control phospholipid metabolism. J Biol Chem 268: 10946-10952.
69. Mishina M, Kamiryo T, Tanaka A, Numa S (1981) Acetyl-coenzyme-A carboxylase from Candida lipolytica. Methods Enzymol 71: 37.
70. Satyanarayana T, Klein HP (1973) Studies on acetyl-coenzyme A synthetase of yeast: inhibition by long-chain acyl-coenzyme A esters. J Bacteriol 115: 600-606.
71. van den Berg MA, de Jong-Gubbels P, Kortland CJ, van Dijken JP, Pronk JT, et al. (1996) The two acetyl-coenzyme A synthetases of Saccharomyces cerevisiae differ with respect to kinetic properties and transcriptional regulation. J Biol Chem 271: 28953-28959.
72. Frenkel EP, Kitchens RL (1977) Purification and properties of acetyl coenzyme A synthetase from bakers' yeast. J Biol Chem 252: 504-507.
73. Kratzer S, Schuller HJ (1995) Carbon source-dependent regulation of the acetyl-coenzyme A synthetase-encoding gene ACS1 from Saccharomyces cerevisiae. Gene 161: 75-79.
74. Grenson M, Hennaut C (1971) Mutation affecting activity of several distinct amino acid transport systems in Saccharomyces cerevisiae. J Bacteriol 105: 477-482.
75. Verma RS, Rao TVG, Prasad R (1984) An inducible, specific and derepressible transport of L-serine in Saccharomyces cerevisiae. Biochim Biophys Acta 778: 289-297.
76. Merkel O, Fido M, Mayr JA, Pruger H, Raab F, et al. (1999) Characterization and function in vivo of two novel phospholipases B/lysophospholipases from Saccharomyces cerevisiae. J Biol Chem 274: 28121-28127.
77. Servouse M, Karst F (1986) Regulation of early enzymes of ergosterol biosynthesis in Saccharomyces cerevisiae. Biochem J 240: 541-547.
78. Kim SA, Copeland L (1997) Acetyl Coenzyme A Acetyltransferase of Rhizobium sp. (Cicer) Strain CC 1192. Appl Environ Microbiol 63: 3432-3437.
79. Kim DY, Stauffacher CV, Rodwell VW (2000) Engineering of Sulfolobus solfataricus HMG-CoA reductase to a form whose activity is regulated by phosphorylation and dephosphorylation. Biochemistry 39: 2269-2275.

80. Song L (2006) Reduction of background interference in the spectrophotometric assay of mevalonate kinase. Anal Bioanal Chem 384: 1444-1445.
81. Voynova NE, Rios SE, Miziorko HM (2004) Staphylococcus aureus mevalonate kinase: isolation and characterization of an enzyme of the isoprenoid biosynthetic pathway. J Bacteriol 186: 61-67.
82. M'Baya B, Fegueur M, Servouse M, Karst F (1989) Regulation of squalene synthetase and squalene epoxidase activities in Saccharomyces cerevisiae. Lipids 24: 1020-1023.
83. Mookhtiar KA, Kalinowski SS, Zhang D, Poulter CD (1994) Yeast squalene synthase. A mechanism for addition of substrates and activation by NADPH. J Biol Chem 269: 11201-11207.
84. Duff S.M.G. D-AJaTR (2005) The development of a medium throughput assay for lanosterol synthase from Leptosphaeria nodorum: Comparison of the enzyme from L. nodorum, Saccharomyces cerevisiae, and two species of Fusarium. Pesticide Biochemistry and Physiology 83: 97-106.
85. Satoh T, Horie M, Watanabe H, Tsuchiya Y, Kamei T (1993) Enzymatic properties of squalene epoxidase from Saccharomyces cerevisiae. Biol Pharm Bull 16: 349-352.
86. Kelly SL, Arnoldi A, Kelly DE (1993) Molecular genetic analysis of azole antifungal mode of action. Biochem Soc Trans 21: 1034-1038.
87. Ching MS, Lennard MS, Tucker GT, Woods HF, Kelly DE, et al. (1991) The expression of human cytochrome P450IA1 in the yeast Saccharomyces cerevisiae. Biochem Pharmacol 42: 753-758.
88. M'Baya B, Karst F (1987) In vitro assay of squalene epoxidase of Saccharomyces cerevisiae. Biochem Biophys Res Commun 147: 556-564.
89. Aoyama Y, Yoshida Y, Sato R (1984) Yeast cytochrome P-450 catalyzing lanosterol 14 alpha-demethylation. II. Lanosterol metabolism by purified P-450(14)DM and by intact microsomes. J Biol Chem 259: 1661-1666.
90. Nes WD, McCourt BS, Zhou WX, Ma J, Marshall JA, et al. (1998) Overexpression, purification, and stereochemical studies of the recombinant (S)-adenosyl-L-methionine: delta 24(25)- to delta 24(28)-sterol methyl transferase enzyme from Saccharomyces cerevisiae. Arch Biochem Biophys 353: 297-311.
91. Moore JT, Gaylor JL (1969) Isolation and purification of an S-adenosylmethionine: delta 24-sterol methyltransferase from yeast. J Biol Chem 244: 6334-6340.
92. Zinser E, Paltauf F, Daum G (1993) Sterol composition of yeast organelle membranes and subcellular distribution of enzymes involved in sterol metabolism. J Bacteriol 175: 2853-2858.
93. Nes WD, Marshall JA, Jia Z, Jaradat TT, Song Z, et al. (2002) Active site mapping and substrate channeling in the sterol methyltransferase pathway. J Biol Chem 277: 42549-42556.
94. Anderson MS, Yarger JG, Burck CL, Poulter CD (1989) Farnesyl diphosphate synthetase. Molecular cloning, sequence, and expression of an essential gene from Saccharomyces cerevisiae. J Biol Chem 264: 19176-19184.
95. Street IP, Coffman HR, Baker JA, Poulter CD (1994) Identification of Cys139 and Glu207 as catalytically important groups in the active site of isopentenyl diphosphate:dimethylallyl diphosphate isomerase. Biochemistry 33: 4212-4217.
96. Czabany T, Athenstaedt K, Daum G (2007) Synthesis, storage and degradation of neutral lipids in yeast. Biochim Biophys Acta 1771: 299-309.
97. Koffel R, Tiwari R, Falquet L, Schneiter R (2005) The Saccharomyces cerevisiae YLL012/YEH1, YLR020/YEH2, and TGL1 genes encode a novel family of membrane-anchored lipases that are required for steryl ester hydrolysis. Mol Cell Biol 25: 1655-1668.
98. Lewis TA, Rodriguez RJ, Parks LW (1987) Relationship between intracellular sterol content and sterol esterification and hydrolysis in Saccharomyces cerevisiae. Biochim Biophys Acta 921: 205-212.

99. Taketani S, Nishino T, Katsuki H (1981) Purification and properties of sterol-ester hydrolase from Saccharomyces cerevisiae. J Biochem 89: 1667-1673.
100. Mullner H, Deutsch G, Leitner E, Ingolic E, Daum G (2005) YEH2/YLR020c encodes a novel steryl ester hydrolase of the yeast Saccharomyces cerevisiae. J Biol Chem 280: 13321-13328.
101. Yu C, Kennedy NJ, Chang CC, Rothblatt JA (1996) Molecular cloning and characterization of two isoforms of Saccharomyces cerevisiae acyl-CoA:sterol acyltransferase. J Biol Chem 271: 24157-24163.
102. Taketani S, Nishino T, Katsuki H (1979) Characterization of sterol-ester synthetase in Saccharomyces cerevisiae. Biochim Biophys Acta 575: 148-155.
