# Supplementary material for: Mathematical Modeling and Validation of the Ergosterol Pathway in Saccharomyces cerevisiae
Source: PLoS One. 2011 Dec 14;6(12):e28344. doi: 10.1371/journal.pone.0028344 (PMC3237449; doi:10.1371/journal.pone.0028344)
Supplement: Table S3 — Incoming and outgoing fluxes at model nodes. (PDF) [file pone.0028344.s005.pdf]

TABLE S3. *Incoming and outgoing fluxes at model nodes*

	micromole /min/l
$V_1^+ = v_{12,1}$	0.882
$V_1^- = v_{1,2}$	0.882
$V_2^+ = v_{1,2} + v_{3,2} + v_{4,2}$	0.885
$V_2^- = v_{2,3} + v_{2,4} + v_{2,5}$	0.885
$V_3^+ = v_{2,3} + v_{8,3} + v_{18,3} + v_{19,3}$	0.798
$V_3^- = v_{3,2} + v_{3,7} + v_{3,8}$	0.798
$V_4^+ = v_{2,4}$	0.000119
$V_4^- = v_{4,2} + v_{4,17}$	0.000119
$V_5^+ = v_{2,5} + v_{6,5} + v_{7,5}$	0.112
$V_5^- = v_{5,6} + v_{5,7}$	0.112
$V_6^+ = v_{5,6}$	0.0181
$V_6^- = v_{6,5} + v_{6,17}$	0.0181
$V_7^+ = v_{5,7} + v_{8,7} + v_{3,7} + v_{18,7} + v_{19,7}$	0.896
$V_7^- = v_{7,5} + v_{7,8} + v_{7,143}$	0.896
$V_8^+ = v_{7,8} + v_{3,8} + v_{20,8}$	0.0618
$V_8^- = v_{8,3} + v_{8,7} + v_{8,18} + v_{8,20}$	0.0618
$V_9^+ = v_{11,9}$	4.568
$V_9^- = v_{9,10} + v_{9,15} + v_{9,169}$	4.568
$V_{10}^+ = v_{9,10}$	2.924
$V_{10}^- = v_{10,156}$	2.924
$V_{11}^+ = v_{12,11}$	6.177
$V_{11}^- = v_{11,14} + v_{11,9}$	6.177
$V_{12}^+ = v_{158,12} + v_{24,12} + v_{4,17} + v_{6,17} + v_{33,30} + v_{34,31} + v_{35,32} + v_{40,39}$	68.074
$V_{12}^- = v_{12,1} + v_{12,11} + v_{12,148} + v_{12,23} + v_{30,33} + v_{31,34} + v_{32,35}$	68.074
$V_{13}^+ = v_{137,13} + v_{166,165} + v_{166,167}$	54.812
$V_{13}^- = v_{12,1} + v_{13,132} + v_{9,10}$	54.812
$V_{14}^+ = v_{11,14} + v_{7,8} + v_{3,8} + v_{18,19}$	1.675
$V_{14}^- = v_{14,142} + v_{17,145}$	1.675
$V_{15}^+ = v_{9,15}$	1.643
$V_{15}^- = v_{7,8} + v_{3,8} + v_{15,144} + v_{18,19} + v_{15,168}$	1.643
$V_{16}^+ = v_{147,16}$	1.643
$V_{16}^- = v_{9,15}$	1.643
$V_{17}^+ = v_{4,17} + v_{6,17}$	0.0164
$V_{17}^- = v_{17,145}$	0.0164
$V_{18}^+ = v_{8,18} + v_{21,18}$	**0.0545**(*)
$V_{18}^- = v_{18,3} + v_{18,7} + v_{18,19} + v_{18,21}$	**0.0555**
$V_{19}^+ = v_{18,19} + v_{22,19}$	0.0283
$V_{19}^- = v_{19,3} + v_{19,7} + v_{19,22}$	0.0283
$V_{20}^+ = v_{8,20}$	**0.02**
$V_{20}^- = v_{20,8}$	**0.019**
$V_{21}^+ = v_{18,21}$	**0.02**
$V_{21}^- = v_{21,18}$	**0.019**

$V_{22}{}^{+} = v_{19,22}$	**0.01**
$V_{22}{}^{-} = v_{22,19}$	**0.005**
$V_{23}{}^{+} = v_{12,23}$	0.88
$V_{23}{}^{-} = v_{2,3} + v_{5,7}$	0.88
$V_{24}{}^{+} = v_{25,24}$	9.777
$V_{24}{}^{-} = v_{12,23} + v_{24,12}$	9.777
$V_{25}{}^{+} = v_{38,25} + v_{124,25}$	24.491
$V_{25}{}^{-} = v_{25,24} + v_{24,12} + v_{25,26}$	24.491
$V_{26}{}^{+} = v_{25,26}$	5.818
$V_{26}{}^{-} = v_{26,27}$	5.818
$V_{27}{}^{+} = v_{26,27}$	5.818
$V_{27}{}^{-} = v_{27,28}$	5.818
$V_{28}{}^{+} = v_{27,28}$	5.818
$V_{28}{}^{-} = v_{28,29} + v_{28,179}$	5.818
$V_{29}{}^{+} = v_{28,29}$	3.494
$V_{29}{}^{-} = v_{29,30}$	3.494
$V_{30}{}^{+} = v_{29,30} + v_{33,30}$	**3.495**
$V_{30}{}^{-} = v_{30,31} + v_{30,33}$	**3.647**
$V_{31}{}^{+} = v_{30,31} + v_{34,31}$	**3.5**
$V_{31}{}^{-} = v_{31,32} + v_{31,34}$	**3.974**
$V_{32}{}^{+} = v_{31,32} + v_{39,32} + v_{37,32} + v_{35,32}$	6.199
$V_{32}{}^{-} = v_{32,35} + v_{32,39} + v_{32,37} + v_{32,186}$	6.199
$V_{33}{}^{+} = v_{30,33}$	**0.153**
$V_{33}{}^{-} = v_{33,30}$	**0.0017**
$V_{34}{}^{+} = v_{31,34}$	**0.48**
$V_{34}{}^{-} = v_{34,31}$	**0.0063**
$V_{35}{}^{+} = v_{32,35} + v_{40,35}$	**1.365**
$V_{35}{}^{-} = v_{35,32} + v_{35,40}$	**0.699**
$V_{36}{}^{+} = v_{39,36} + v_{37,36}$	**2.988**
$V_{36}{}^{-} = v_{36,39} + v_{36,371} + v_{36,372} + v_{36,373}$	**5.836**
$V_{37}{}^{+} = v_{32,37} + v_{36,371} + v_{36,372} + v_{36,373}$	**2.968**
$V_{37}{}^{-} = v_{37,32} + v_{37,36} + v_{37,39}$	**1.537**
$V_{38}{}^{+} = v_{124,38} + v_{125,38}$	16.289
$V_{38}{}^{-} = v_{38,25}$	16.289
$V_{39}{}^{+} = v_{32,39} + v_{36,39} + v_{40,39} + v_{37,39}$	**5.836**
$V_{39}{}^{-} = v_{39,32} + v_{39,36}$	**4.139**
$V_{40}{}^{+} = v_{35,40}$	**0.682**
$V_{40}{}^{-} = v_{40,35} + v_{40,39}$	**0.789**

(*) Bold values indicate unbalanced incoming (V_i^+) and outgoing (V_i^-) fluxes**.**

Individual fluxes. Fluxes estimated (μM/l/min).

The vescicular traffic for the complex sphingolipids and ergosterol was collapsed into a single transport process (Figs 1 and 2). We estimated these fluxes based in experimental data generated by us [1]. The PM forward vescicular fluxes are:

$v_{8,20} = 0.02$

$v_{18,21} = 0.02$

$v_{19,22} = 0.01$

The vescicular PM to ER-Golgi fluxes were estimated as a percent from the corresponding three forward fluxes $v_{8,20}$, $v_{18,21}$, and $v_{19,22}$ as:

$v_{20,8} = v_{8,20} \times 0.95 = 0.019$

$v_{21,18} = v_{18,21} \times 0.95 = 0.019$

$v_{22,19} = v_{19,22} \times 0.5 = 0.005$

Fluxes estimated from kinetics equations.

Fluxes obtained based in the Michaelis-Menten (MM) kinetics:

$v_{2,4} = f(X_2, \mathrm{KM}_{2\_136}, Vmax_{2\_136}) = 0.00012$

$v_{2,5} = f(X_2, \mathrm{KM}_{2\_154}, Vmax_{2\_154}) = 0.0987$

$v_{3,2} = f(X_3, \mathrm{KM}_{3\_129}, Vmax_{3\_129}) = 0.0031$

$v_{7,5} = f(X_7, \mathrm{KM}_{7\_153}, Vmax_{7\_153}) = 0.0115$

$v_{8,7} = f(X_8, \mathrm{KM}_{8\_151}, Vmax_{8\_151}) = 0.00484$

$v_{9,10} = f(X_{13}, \mathrm{KM}_{13\_138}, Vmax_{13\_138}) = 2.924$

$v_{11,14} = f(X_{11}, \mathrm{KM}_{11\_139}, Vmax_{11\_139}) = 1.608$

$v_{15,144} = f(X_{128}, \mathrm{KM}_{128\_144}, Vmax_{128\_144}) = 1.57$

$v_{15,168} = f(X_{15}, \mathrm{KM}_{15\_168}, Vmax_{15\_168}) = 0.0074$

$v_{24,12} = f(X_{25}, X_{24}, \mathrm{KM}_{25\_152}, \mathrm{KM}_{24\_152}, Vmax_{25\_152}) = 8.896$

$v_{28,179} = f(X_{28}, KM_{28\_179}, Vmax_{28\_179}) = 2.324$

Fluxes related with serine (X_{13})

$v_{137,13} = f(X_{137}, KM_{137\_131}, Vmax_{37\_131}) =1.259$

$v_{166,165} = f(X_{166}, KM_{166\_165}, Vmax_{166\_165}) = 53.553$

Two fluxes were obtained with the bi-substrate MM with Hill order for one of the substrates:

$v_{3,8} = f(X_3, X_{15}, KM_{3\_133}, KM_{15\_133}, Vmax_{3\_133}, H_{\_133}) = 0.017$

$v_{7,8} = f(X_7, X_{15}, KM_{7\_133}, KM_{15\_133}, Vmax_{15\_133}, H_{133}) = 0.025$

When the corresponding V_{max} values are not available directly, they were based on the specific activity (see Alvarez-Vasquez *et al.* [2] Appendix A.3.3 section for details).

Fluxes obtained from kinetics equations and relationships.

The Pal-CoA (X_{12}) flux through the SPT (X_{157}) is smaller than through the G3P-acyltransferase (X_{149}). This is suggested by the greater concentration of phospholipids over the sphingolipids found in yeast cells [3,4].

$v_{12,1} = v_{12,11} \div 7 = 0.882$

The flux through the sphingoid base kinase (X_{136}) for PHS (X_5) is higher than for DHS (X_2). This is reflected in a greater PHS-P over DHS-P concentration observed for the lyase deletion strain Δ*dpl1* [5].

$v_{5,6} = v_{2,4} \times 150 = 0.018$

The flux through lyase (X_{150}) is responsible of the main sphingoid phosphate (X_4, X_6) degradation [6]. We set the diverting flux value through this enzyme as 90% of the sphingosine base kinase (X_{136}) incoming flux.

$v_{4,17} = v_{2,4} \times 0.90 = 0.0001$

$v_{6,17} = v_{5,6} \times 0.90 = 0.016$

The four fluxes below are involved in salvage ceramide formation and were obtained assuming that the phytoceramide formation represents 70% of the total flux through the IPCase (X_{151} plus X_{164}).

$v_{8,3} = f(X_8, \mathrm{KM}_{8\_151}, Vmax_{8\_151}) \times 0.3 = 0.00145$

$v_{18,3} = f(X_{18}, \mathrm{KM}_{18\_151}, Vmax_{8\_151}) \times 0.3 = 0.0036$

$v_{18,7} = f(X_{18}, \mathrm{KM}_{18\_151}, Vmax_{8\_151}) \times 0.7 = 0.0085$

$v_{19,3} = v_{18,19} \times 0.3 = 0.00699$

The fluxes $v^a_{36,37}$, $v^b_{36,37}$, and $v^c_{36,37}$ were obtained from the stoichiometry, multiplied by the complex sphingolipid relation proposed in Wu *et al.* ([4], Fig 7A), as follows:

$v^a_{36,37} = (v_{32,39} + v_{40,39}) \times (1.02 \div 2.505) = 1.188$

$v^b_{36,37} = (v_{32,39} + v_{40,39}) \times (1.4 \div 2.505) = 1.63$

$v^c_{36,37} = (v_{32,39} + v_{40,39}) \times (0.085 \div 2.505) = 0.099$

The $v_{124,25}$ flux, associated with pyruvate (X_{124}) degradation, through pyruvate dehydrogenase (X_{126}), was estimated from the stoichiometry, multiplied by the flux relationship during fermentative growth reported by Frick and Wittmann ([7], Fig 3):

$v_{124,25} = (v_{25,26} + v_{24,12} + v_{25,24} - v_{125,38}) \times (9.7 \div (16.1+9.7)) = 8.202$

The incoming flux $v_{124,38}$, for the internal acetate (X_{38}) flux coming from pyruvate decarboxylase (X_{122}), was obtained from the stoichiometry, multiplied by a relationship from Frick and Wittmann for the flux relationship of pyruvate to acetate during fermentative growth ([7], Fig 3) as:

$v_{124,38} = (v_{25,26} + v_{24,12} + v_{25,24} - v_{125,38}) \times (16.1 \div (16.1+9.7)) = 13.965$

The external acetate influx $v_{125,38}$ was estimated from Teusink *et al.* ([8], Table 1) as:

$v_{125,38} = 0.002\ \mu\mathrm{M/min/mg\ protein} \times 1162\ \mathrm{mg\ protein/l} = 2.324$

The incoming palmitate (X_{158}) influx was calculated based on the specific activity from palmitoyl-CoA synthase as:

$v_{158,12} = X_{130}$ µM/min/mg protein × 1162 mg protein/l = 0.0508 ×1162 = 59.029

Fluxes through the ergosterol pools.

We assume that the bidirectional non-vesicular ergosterol flux between PM and ER runs through the PM-associated membranes (PAM) structures. Although there is no direct experimental evidence to validate this assumption, there is indirect evidence that it is probably true (*e.g.* [9-12]).

The ergosterol flux moving forward-backward through the PM was estimated as 6×10^4 molecules/sec, according to Sullivan *et al.* ([13], Appendix 1). Sullivan and collaborators based their calculations on the assumption that 1.5×10^{-11} ergosterol g CDW/cell is equivalent to 1×10^8 ergosterol molecules/cell.

In order to transform Sullivan's ergosterol flux units from molecules/sec to µmol/l/min we proceeded as follows:

a - Calculate how many ergosterol molecules there are in gr CDW / cell

10^8 ergosterol molecules/cell ÷ 1.5×10^{-11} gr CDW/cell = 6.66×10^{18} ergosterol molecules / gr CDW / cell

b - Divide by the Avogadro number to obtain the moles per g CDW / cell

6.66×10^{18} ergosterol molecules / g CDW ÷ 6.022×10^{23} = 1.07×10^{-5} mol / gr CDW / cell

c - Obtain the flux per cell in moles / g CDW / second

6×10^4 ergosterol molecules/sec × 1.07×10^{-5} mol / g CDW ÷ 10^8 ergosterol molecules = 6.42×10^{-9} mol/gr CDW/cell/sec.

d - Calculate the moles/l/cell

Sullivan *et al.* assumed the average yeast cell volume as 70 µm^3 / cell

6.42×10^{-9} mol/gr CDW/sec ÷ 7×10^{-5} l/cell = 9.17×10^{-5} mol/l/sec which is equivalent to 5.5 μmol/l/min.

Computations of fluxes between compartments must take into account the volumes of the respective compartments [14]. For the present case, we can reasonably assume that the PAMs and the PM share similar areas of contact (PAM outer and PM inner), which are anchored by proteins such as oxysterol-binding protein homologues and presumably serve as the venues for non-vesicular flux [15]. We can furthermore assume that not just the areas but also the volumes occupied by these two inter-compartmental sub-domains of the ER and PM are similar, because the thicknesses of PAMs and PM are almost identical with between 6-7 nm for the former [11] and approximately 8 nm for the latter [16].

Pichler and collaborators [11] estimated that yeast contains on average 1,100 PAM's between the ER and PM, with a distance of 10-25 nm between the two lipid membranes, which implies that they are truly associated. One should note that the total ER area directly involved in the traffic of non-vesicular ergosterol with the PM is smaller than the total area of the ER, because the ER is closely associated with almost all cellular organelles. For example, the ER is in close contact with the mitochondria-associated membrane (MAM) and with the nucleus (ER-nucleus fractions) through highly specialized sub-fractions that are allegedly not directly involved in the non-vescicular ergosterol traffic between ER and PM.

e - To calculate the non-vesicular ergosterol flux moving from the ER to the PM and backwards, the 5.5 μmol/l/min total ergosterol flux needs to be corrected with respect to the different volumes of the ER and PM, and subtracted from the total vesicular-ergosterol and Yeh2p fluxes.

Bauman and collaborators estimated a ratio of 2.5:1 for the areas of ER and PM [17], and Sullivan *et al.* estimated a yeast PM surface and cross-sectional area of 8.2×10^{7} nm^2 and 0.6 nm^2, respectively [13], To estimate how much area is occupied by proteins, and assuming that these are all transmembranal proteins, we used ER and PM information from Zinser and colaborators [18]. These

authors calculated 0.068 and 0.63 mg lipid (ergosterol plus phospholipids) per mg protein, respectively.

With this information, we estimated the ER volume as

$A_{\mathrm{ER}} = 8.2\times10^7\ \mathrm{nm}^2 \times 0.6\ \mathrm{nm} \times 0.068\ \mathrm{mg/mg} \times 2.5 = 8.364\times10^6\ \mathrm{nm}^3$.

The PM volume was split in half in order to account for the volumes of the inner and outer bilayers, The outer bilayer was further split with respect the ergosterol associated with DIG. The inner ergosterol was not further compartmentalized into PAM and non-PAM areas because none of the inner ergosterol is associated with complex sphingolipid (called also chemically active ergosterol); it is therefore not restricted with respect to lateral movements. In other words, because the PAM-associated and the non-PAM areas share the same free sterol (which is not associated with CS), we collapsed these two volumes into one.

Based on the above assumptions and experimental data, the inner PM area (X_{39}) was calculated as $A_{39} = 8.2\times10^7\ \mathrm{nm}^2 \times 0.6\ \mathrm{nm} \times 0.63\ \mathrm{mg/mg} \times 0.5 = 1.549\times10^7\ \mathrm{nm}^3$.

For the calculation of the volume of RAF, we assumed that 80% of the outer PM proteins is raft associated; this assumption was based on the preferential association of Gas1p, Pma1p and at least seven other integral proteins with yeast microdomains [19]. Additionally there is experimental evidence that Pma1p is the most abundant protein in the PM [20,21]. We thus estimated, based on previous theoretical information, that 35% of the outer PM area is DIG associated [22]:

$A_{\mathrm{RAF}} = 8.2\times10^7\ \mathrm{nm}^2 \times 0.6\ \mathrm{nm} \times 0.5 \times 0.35 \times 0.63\ \mathrm{mg\ lipid/mg\ protein} \times (1 - 0.8) = 1.1158\times10^6\ \mathrm{nm}^3$.

Equivalently, we assumed that 65% of the PM outer area (X_{36}) is not associated with DIG [22] and that 20% of the total PM proteins is present in it:

$A_{36} = 8.2\times10^7\ \mathrm{nm}^2 \times 0.6\ \mathrm{nm} \times 0.5 \times (1 - 0.35) \times 0.63\ \mathrm{mg\ lipid/mg\ protein} \times (1 - 0.2) = 7.9349\times10^6\ \mathrm{nm}^3$.

Based on the above volume calculations, the bidirectional fluxes from the ER to the PM and inside the PM were calculated as:

$v_{32,39}$ = 5.5 $\div (A_{\text{ER}} / A_{\text{PAM}})$ - $v_{32,37}$ - $v_{40,39}$ = 2.75 - 0.05 - 0.235 = 2.811 μmol/l/min;

$v_{39,32}$ = 5.5 - $v_{32,39}$ – $v_{37,32}$ = 5.5 - 3.9402 - 0.05 - 0.0430 = 2.645 μmol/l/min;

$v_{39,,36}$ = $(v_{32,39} + v_{40,39}) \div (A_{39}/ A_{36})$ = 1.494 μmol/l/min;

$v_{36,37}$ = $v_{39,36} \div (A_{36} / A_{\text{RAF}})$ = 0.21 μmol/l/min;

$v_{36,39}$ = $v_{37,36} \div (A_{36} / A_{39})$ = 2.918 μmol/l/min;

$v_{37,36}$ = $v_{36,37} \div (A_{\text{RAF}} / A_{X36})$ = 1.494 μmol/l/min.

The forward-backward steryl-ester fluxes $v_{30,33}$, $v_{31,34}$, $v_{32,35}$, $v_{33,30}$, $v_{34,31}$, $v_{35,32}$, and $v_{40,39}$ were estimated assuming MM kinetics. Please note that the fluxes through the SE synthase (X_{181}, X_{183}) do not include the palmitoyl-CoA (X_{12}) into their kinetics. If the acylated fatty acid is taken into account, the fluxes are reduced significantly and do not represent properly the dynamics reported by Taylor and Parks [23]. This suggests that the rate limiting steps for these fluxes depend on their association with the sterols.

Because of difficulties obtaining a factor to convert % of total sterols into μM, the seven sterol-ester related fluxes below were calculated using % total sterols in the substrates' units. This causes a discrepancy in the flux estimations because the others parameters used for the flux calculation are based on μM units. This discrepancy mostly cancels out due to two facts: *a*) the % of total sterols for the substrates was utilized for the flux calculations in both, the steryl-ester synthesis and degradation. (*e*.g. [24]). *b*) the unknown substrate correction factor that is necessary to transform the substrate units from % of total sterols into μM is probably similar for the forward and backward fluxes.

For these reasons, the errors in the fluxes caused by different parameter units are ameliorated. Furthermore, the similarity between the dynamics of the SL-E model presented in this work and the

experimental time courses [23] lends additional support that the assumptions in the flux estimations are reasonable.

To estimate the back and forth fluxes between the ER and the LP's we calculated the LP volume based in the experimental LP diameter range of 0.3-0.4 μm obtained from electron microscopy of isolated yeast lipid particles from Leber and colaborators [25]. We used the value of 0.3 μm for our calculations. Exploratory SL-E dynamics (simulation results not shown) suggest *in vivo* LP diameter smaller than the reported by Leber [25]. During the Leber and collaborators LP isolation the LP size could be affected. Until our knowledge, *in vivo* or cryofracture microscopy measurements for the yeast LP diameters is not available in the literature.

$A_{LP} = 4/3 \times \pi \times (0.3\ \mu m \times 1000/2)^3 = 1.413\times10^7\ \ nm^3$

The bidirectional fluxes from the ER to the LP and viceversa were calculated as:

$v_{30,33} = f(X_{30}, \mathrm{KM}_{30\_181}, Vmax_{30\_181}) \div (A_{ER} / A_{LP}) = 0.153$ μmol/l/min;

$v_{31,34} = f(X_{31}, \mathrm{KM}_{31\_181}, Vmax_{31\_181}) \div (A_{ER} / A_{LP}) = 0.48$ μmol/l/min;

$v_{32,35} = f(X_{32}, \mathrm{KM}_{32\_183}, Vmax_{32\_183}) \div (A_{ER} / A_{LP}) = 0.682$ μmol/l/min.

$v_{33,30} = f(X_{33}, \mathrm{KM}_{33\_180}, Vmax_{33\_180}) \div (A_{LP} / A_{ER}) = 0.0017$ μmol/l/min.

$v_{34,31} = f(X_{34}, \mathrm{KM}_{34\_180}, Vmax_{34\_180}) \div (A_{LP} / A_{ER}) = 0.0063$ μmol/l/min.

$v_{35,32} = f(X_{35}, \mathrm{KM}_{35\_180}, Vmax_{35\_180}) \div (A_{LP} / A_{ER}) = 0.0168$ μmol/l/min.

The flux from the Steryl Ergosterol-2 (X_{40}) to the Ergosterol-I (X_{39}) was calculated as:

$v_{40,39} = f(X_{40}, \mathrm{KM}_{40\_182}, Vmax_{40\_182}) \div (A_{LP} / A_{39}) = 0.106$ μmol/l/min.

Fluxes obtained from the stoichiometry of the pathway system:

$v_{1,2} = v_{12,1}$

$v_{2,3} = v_{1,2} + v_{4,2} + v_{3,2} - v_{2,4} - v_{2,5}$

$v_{3,7} = v_{2,3} + v_{8,3} + v_{18,3} + v_{19,3} - v_{3,8} - v_{3,2}$

$v_{4,2} = v_{2,4} - v_{4,17}$

$v_{5,7} = v_{2,5} + v_{6,5} + v_{7,5} - v_{5,6}$

$v_{6,5} = v_{5,6} - v_{6,17}$

$v_{7,143} = v_{3,7} + v_{5,7} + v_{8,7} + v_{18,7} + v_{19,7} - v_{7,5} - v_{7,8}$

$v_{8,18} = v_{3,8} + v_{7,8} + v_{20,8} - v_{8,3} - v_{8,7} - v_{8,20}$

$v_{9,15} = v_{7,8} + v_{3,8} + v_{15,144} + v_{18,19} + v_{15,168}$

$v_{10,156} = v_{9,10}$

$v_{11,9} = v_{9,10} + v_{9,15} + v_{9,169}$

$v_{12,11} = v_{11,9} + v_{11,14}$

$v_{12,23} = v_{2,3} + v_{5,7}$

$v_{12,148} = v_{158,12} + v_{24,12} + v_{4,17} + v_{6,17} - v_{12,1} - v_{12,23} - v_{12,11} + v_{33,30} + v_{34,31} + v_{35,32} + v_{40,39} - v_{30,33} - v_{31,34} - v_{32,35}$

$v_{13,132} = v_{166,165} + v_{166,167} + v_{137,13} - v_{9,10} - v_{12,1}$

$v_{14,142} = v_{11,14} + v_{7,8} + v_{3,8} + v_{18,19} - v_{17,145}$

$v_{14,145} = v_{17,145}$

$v_{17,145} = v_{4,17} + v_{6,17}$

$v_{18,19} = v_{8,18} - v_{18,3} - v_{18,7}$

$v_{19,7} = v_{18,19} + v_{22,19} - v_{19,3} - v_{19,22}$

$v_{24,23} = v_{12,23}$

$v_{25,24} = v_{12,23} + v_{24,12}$

$v_{25,26} = v_{26,27} = v_{27,28} = v_{28,29} + v_{28,179}$

$v_{28,29} = v_{29,30} = v_{30,31} = v_{31,32}$

$v_{30,31} = v_{31,32}$

$v_{31,32} = v_{32,39} + v_{32,35}$

$v_{32,37} = v_{8,20} + v_{18,21} + v_{19,22}$

$v_{32,186} = v_{31,32} + v_{39,32} + v_{37,32} + v_{35,32} - v_{32,35} - v_{32,39} - v_{32,37}$

$v_{35,40} = v_{32,35}$

$v_{37,32} = v_{20,8} + v_{21,18} + v_{22,19}$

$v_{40,35} = v_{35,40}$

$v_{147,16} = v_{9,15}$

References.

1. Alvarez-Vasquez F, Sims KJ, Cowart LA, Okamoto Y, Voit EO, et al. (2005) Simulation and validation of modelled sphingolipid metabolism in Saccharomyces cerevisiae. Nature 433: 425-430.
2. Alvarez-Vasquez F, Sims KJ, Hannun YA, Voit EO (2004) Integration of kinetic information on yeast sphingolipid metabolism in dynamical pathway models. J Theor Biol 226: 265-291.
3. Wu WI, Lin YP, Wang E, Merrill AH, Jr., Carman GM (1993) Regulation of phosphatidate phosphatase activity from the yeast Saccharomyces cerevisiae by sphingoid bases. J Biol Chem 268: 13830-13837.
4. Wu WI, McDonough VM, Nickels JT, Jr., Ko J, Fischl AS, et al. (1995) Regulation of lipid biosynthesis in Saccharomyces cerevisiae by fumonisin B1. J Biol Chem 270: 13171-13178.
5. Skrzypek MS, Nagiec MM, Lester RL, Dickson RC (1999) Analysis of phosphorylated sphingolipid long-chain bases reveals potential roles in heat stress and growth control in Saccharomyces. J Bacteriol 181: 1134-1140.
6. Mandala SM, Thornton R, Tu Z, Kurtz MB, Nickels J, et al. (1998) Sphingoid base 1-phosphate phosphatase: a key regulator of sphingolipid metabolism and stress response. Proc Natl Acad Sci U S A 95: 150-155.
7. Frick O, Wittmann C (2005) Characterization of the metabolic shift between oxidative and fermentative growth in Saccharomyces cerevisiae by comparative 13C flux analysis. Microb Cell Fact 4: 30.
8. Teusink B, Passarge J, Reijenga CA, Esgalhado E, van der Weijden CC, et al. (2000) Can yeast glycolysis be understood in terms of in vitro kinetics of the constituent enzymes? Testing biochemistry. Eur J Biochem 267: 5313-5329.
9. Lebiedzinska M, Szabadkai G, Jones AW, Duszynski J, Wieckowski MR (2009) Interactions between the endoplasmic reticulum, mitochondria, plasma membrane and other subcellular organelles. Int J Biochem Cell Biol 41: 1805-1816.
10. Levine T (2004) Short-range intracellular trafficking of small molecules across endoplasmic reticulum junctions. Trends Cell Biol 14: 483-490.
11. Pichler H, Gaigg B, Hrastnik C, Achleitner G, Kohlwein SD, et al. (2001) A subfraction of the yeast endoplasmic reticulum associates with the plasma membrane and has a high capacity to synthesize lipids. Eur J Biochem 268: 2351-2361.
12. Prinz WA (2007) Non-vesicular sterol transport in cells. Prog Lipid Res 46: 297-314.
13. Sullivan DP, Ohvo-Rekila H, Baumann NA, Beh CT, Menon AK (2006) Sterol trafficking between the endoplasmic reticulum and plasma membrane in yeast. Biochem Soc Trans 34: 356-358.
14. Alves R, Antunes F, Salvador A (2006) Tools for kinetic modeling of biochemical networks. Nat Biotechnol 24: 667-672.
15. Schulz TA, Choi MG, Raychaudhuri S, Mears JA, Ghirlando R, et al. (2009) Lipid-regulated sterol transfer between closely apposed membranes by oxysterol-binding protein homologues. J Cell Biol 187: 889-903.
16. Schibeci A, Rattray JB, Kidby DK (1973) Electron microscope autoradiography of labelled yeast plasma membrane. Biochim Biophys Acta 323: 532-538.
17. Baumann NA, Sullivan DP, Ohvo-Rekila H, Simonot C, Pottekat A, et al. (2005) Transport of newly synthesized sterol to the sterol-enriched plasma membrane occurs via nonvesicular equilibration. Biochemistry 44: 5816-5826.
18. Zinser E, Sperka-Gottlieb CD, Fasch EV, Kohlwein SD, Paltauf F, et al. (1991) Phospholipid synthesis and lipid composition of subcellular membranes in the unicellular eukaryote Saccharomyces cerevisiae. J Bacteriol 173: 2026-2034.

19. Malinsky J, Opekarova M, Tanner W The lateral compartmentation of the yeast plasma membrane. Yeast 27: 473-478.
20. Bagnat M, Chang A, Simons K (2001) Plasma membrane proton ATPase Pma1p requires raft association for surface delivery in yeast. Mol Biol Cell 12: 4129-4138.
21. Bagnat M, Keranen S, Shevchenko A, Shevchenko A, Simons K (2000) Lipid rafts function in biosynthetic delivery of proteins to the cell surface in yeast. Proc Natl Acad Sci U S A 97: 3254-3259.
22. Simons K, Toomre D (2000) Lipid rafts and signal transduction. Nat Rev Mol Cell Biol 1: 31-39.
23. Taylor FR, Parks LW (1978) Metabolic interconversion of free sterols and steryl esters in Saccharomyces cerevisiae. J Bacteriol 136: 531-537.
24. Voit EO (2000) Computational analysis of biochemical systems : a practical guide for biochemists and molecular biologists. New York: Cambridge University Press. xii, 531 p. p.
25. Leber R, Zinser E, Zellnig G, Paltauf F, Daum G (1994) Characterization of lipid particles of the yeast, Saccharomyces cerevisiae. Yeast 10: 1421-1428.
