# Supplementary material for: Mathematical Modeling and Validation of the Ergosterol Pathway in Saccharomyces cerevisiae
Source: PLoS One. 2011 Dec 14;6(12):e28344. doi: 10.1371/journal.pone.0028344 (PMC3237449; doi:10.1371/journal.pone.0028344)
Supplement: Table S4 — Eigenvalues for the flux balanced SL-E S-system model. (PDF) [file pone.0028344.s006.pdf]

Table S4. *Eigenvalues for the flux balanced SL-E S-system model*

Real	*Imaginary*	*Real*	*Imaginary*
-6.93E+03	0	-1.33E-01	0
-1.75E+02	0	-1.15E-01	0
-8.17E+01	0	-9.26E-02	0
-5.91E+01	0	-8.21E-02	0
-5.91E+01	0	-7.00E-02	8.73E-03
-2.96E+01	0	-7.00E-02	-8.73E-03
-1.12E+01	0	-5.49E-02	0
-8.94E+00	0	-1.67E-02	7.54E-02
-7.55E+00	0	-1.67E-02	-7.54E-02
-3.45E+00	0	-1.66E-02	0
-3.12E+00	0	-1.29E-02	0
-1.86E+00	0	-1.02E-02	6.56E-03
-1.52E+00	0	-1.02E-02	-6.56E-03
-1.44E+00	0	-8.53E-03	0
-1.43E+00	0	-7.41E-03	0
-1.12E+00	0	-4.54E-03	0
-4.27E-01	2.57E-01	-4.13E-03	0
-4.27E-01	-2.57E-01	-1.13E-03	0
-3.57E-01	0	-3.72E-04	0
-2.20E-01	0	-3.34E-04	0

All eigenvalues have negative real parts, which indicates that the steady state is locally stable and that the system will return to this steady state following small perturbations. The eight non-zero imaginary parts indicate potential oscillatory dynamics in response to perturbations in the system. The wide range for the real parts suggest that the model potentially encompasses stiff and sloppy behaviors [1].

References.

1. Daniels BC, Chen YJ, Sethna JP, Gutenkunst RN, Myers CR (2008) Sloppiness, robustness, and evolvability in systems biology. Curr Opin Biotechnol 19: 389-395.
