# Supplementary material for: Mathematical Modeling and Validation of the Ergosterol Pathway in Saccharomyces cerevisiae
Source: PLoS One. 2011 Dec 14;6(12):e28344. doi: 10.1371/journal.pone.0028344 (PMC3237449; doi:10.1371/journal.pone.0028344)
Supplement: Table S5 — Global sensitivity statistics for the SL-E model. (PDF) [file pone.0028344.s007.pdf]

Table S5. *Global sensitivity statistics for the SL-E model.*

		% of Metabolites [(*)]				% of Fluxes [(*)]			
	**Total**	**< 1**	**< 2**	**< 5**	**< 10**	**< 1**	**< 2**	**< 5**	**< 10**
**Logarithmic Gains**	2280	85.00	91.14	97.46	99.30	91.36	95.70	99.47	99.96
**Rate Constants**	1600	76.69	85.75	95.31	98.88	83.72	92.41	98.59	99.81
**Kinetic Orders**	14320	79.58	85.25	91.13	94.90	83.25	88.42	93.95	97.60

[(*)] Percent of the S-system sensitivities magnitudes smaller than 1, 2, 5, and 10 with respect their respective total sensitivities.

Detailed mathematic descriptions of sensitivity equations and their significance in BST can be found elsewhere (*e.g.* [1-3]). The sensitivity output provides valuable information regarding the model quality. Sensitivities with respect to rate constants, kinetic orders, and independent variables (logarithmic gains) with magnitudes greater than 1 imply amplification of a signal produced after a perturbation in the corresponding model parameter. By contrast, an absolute value less than 1 indicates attenuation of the signal. A positive sensitivity indicates that the perturbation signal changes in the same direction, whereas a negative sensitivity indicates that the changes are in opposite directions.

Logarithmic gains characterize the propagation of biochemical signals throughout the system after a change in an independent variable, which causes a change in the dependent variables and fluxes of the system in steady state. The rate constant sensitivities are given by the ratio of the relative change in a time dependent metabolite (X_1 .. X_{40}) or flux (V_1..V_{40}) with respect to a 1% change in a rate constant parameter ($\alpha_1 .. \alpha_{40}$).

Table S5 shows the logarithmic gains, rate constant, and kinetic order sensitivities for the flux balanced S-system model. The vast majority of sensitivities are smaller than 1, indicating that most small perturbations in model parameters will be attenuated. This is a principal characteristic of a robust model. Table 5 results present a significant improvement in the model sensitivities with respect to a previous model for the sphingolipid-glycerolipid pathways alone [4-6]. As discussed

elsewhere [4], the few relatively high sensitivities are in most cases associated with metabolites that are not only involved in the SL-E pathway but in other pathways as well. An example is acetate (X_{38}), which takes part in numerous metabolic pathways, most of which are not represented in the model. The important consequence here is that these variables are significantly less buffered in the model than in reality, thereby leading to artificially high sensitivities and gains. In other cases, high sensitivity values may point to processes that were omitted or misrepresented and will require further attention in the future [4]. Finally, one should note that the sensitivities are unconstrained. Voit (2000) [3] discussed in detail that constraints between parameters, which are dictated by the pathway topology, tend to reduce sensitivities. Overall, the sensitivity profile seems reasonable, and perturbation simulations with high-gain variables have given us no cause for concern.

References.

1. Shiraishi F, Savageau MA (1992) The tricarboxylic acid cycle in Dictyostelium discoideum. II. Evaluation of model consistency and robustness. J Biol Chem 267: 22919-22925.
2. Shiraishi F, Savageau MA (1992) The tricarboxylic acid cycle in Dictyostelium discoideum. III. Analysis of steady state and dynamic behavior. J Biol Chem 267: 22926-22933.
3. Voit EO (2000) Computational analysis of biochemical systems : a practical guide for biochemists and molecular biologists. New York: Cambridge University Press. xii, 531 p. p.
4. Alvarez-Vasquez F, Sims KJ, Cowart LA, Okamoto Y, Voit EO, et al. (2005) Simulation and validation of modelled sphingolipid metabolism in Saccharomyces cerevisiae. Nature 433: 425-430.
5. Alvarez-Vasquez F, Sims KJ, Hannun YA, Voit EO (2004) Integration of kinetic information on yeast sphingolipid metabolism in dynamical pathway models. J Theor Biol 226: 265-291.
6. Alvarez-Vasquez F, Sims KJ, Voit EO, Hannun YA (2007) Coordination of the dynamics of yeast sphingolipid metabolism during the diauxic shift. Theor Biol Med Model 4: 42.
