# Supplementary material for: Mathematical Modeling and Validation of the Ergosterol Pathway in Saccharomyces cerevisiae
Source: PLoS One. 2011 Dec 14;6(12):e28344. doi: 10.1371/journal.pone.0028344 (PMC3237449; doi:10.1371/journal.pone.0028344)
Supplement: Table S6 — SL-E logarithmic gains (of metabolites) with magnitudes greater than one. First row and column correspond to dependent and time independent variables, respectively. The key for the variable names are given in Tables S1 and S2. Rows not presented have only values smaller than one. (PDF) [file pone.0028344.s008.pdf]

Table S6. *SL-E logarithmic gains (metabolites) with magnitudes greater than 1*

	X_1	X_2	X_3	X_4	X_5	X_6	X_7	X_8	X_9	X_{10}	X_{11}	X_{12}	X_{13}	X_{14}	X_{15}	X_{16}
X_{122}	---	-1.50	---	-1.52	-2.43	-2.81	---	---	---	---	---	---	---	---	---	---
X_{123}	---	---	---	---	-1.43	-1.65	---	---	---	---	---	---	---	---	---	---
X_{124}	---	-1.70	---	-1.72	-2.75	-3.17	---	---	---	---	---	---	---	---	---	---
X_{126}	---	---	---	---	---	---	---	---	---	---	---	---	---	---	---	---
X_{127}	---	---	---	---	---	---	---	---	-1.02	---	---	---	---	---	---	-3.50
X_{128}	-1.01	---	---	---	---	---	---	---	---	---	---	---	---	---	---	---
X_{130}	---	-1.13	---	-1.12	-1.82	-2.07	---	---	---	---	---	---	---	---	---	---
X_{132}	---	---	1.22	---	---	---	1.18	1.88	---	6.42	2.07	---	---	1.99	---	-14.29
X_{133}	---	-2.86	-1.79	-2.90	-3.60	-4.15	-1.82	-2.73	---	---	---	---	-4.84	---	---	---
X_{134}	---	---	---	---	---	---	---	1.64	---	---	---	---	---	---	---	---
X_{135}	---	---	---	---	-1.45	-1.68	---	---	---	---	---	---	---	---	---	---
X_{136}	---	---	---	---	---	---	---	-1.51	---	---	---	---	---	---	---	---
X_{138}	---	---	---	1.01	---	---	---	---	---	---	---	---	---	---	---	---
X_{139}	---	---	---	---	---	---	---	---	---	---	---	---	---	---	---	3.65
X_{142}	---	---	---	---	---	---	---	---	---	-1.87	---	---	---	1.66	---	---
X_{143}	---	---	---	---	---	---	---	---	---	---	---	---	---	-2.36	---	5.07
X_{144}	---	---	---	---	---	---	-1.99	-1.87	---	---	---	---	---	---	---	---
X_{146}	---	---	---	---	---	---	---	-2.93	---	---	---	---	---	---	---	---
X_{147}	---	---	---	---	---	---	---	3.41	1.00	-3.29	---	---	---	---	1.01	7.79
X_{148}	---	---	---	---	---	---	---	1.71	---	-1.65	---	---	---	---	---	3.90
X_{149}	---	---	-1.25	---	---	---	-1.20	-1.91	---	-6.52	-2.10	---	---	-2.02	---	14.52
X_{150}	---	---	---	---	---	-1.10	---	-1.07	---	6.49	2.09	---	---	2.18	---	-14.68
X_{152}	---	---	---	---	---	-1.11	---	---	---	---	---	---	---	---	---	---
X_{154}	---	4.75	---	4.81	7.57	8.73	---	---	---	1.72	---	---	---	---	---	-1.51
X_{156}	---	---	-2.17	---	---	1.09	---	-1.51	---	---	---	---	---	---	---	---
X_{157}	---	---	---	---	---	---	---	---	---	-3.94	---	---	---	---	---	4.38
X_{158}	---	2.89	1.81	2.93	3.64	4.19	1.84	2.75	---	---	---	---	---	---	---	---
X_{159}	---	---	1.22	---	---	---	1.17	1.88	---	6.40	2.06	---	---	1.99	---	-14.25
X_{160}	---	-1.50	---	-1.53	-2.40	-2.77	---	---	---	---	---	---	---	---	---	---
X_{165}	---	-3.01	---	-3.05	-4.84	-5.59	---	---	---	---	---	---	---	---	---	---

Table S6. **(cont...).**

	X_{17}	X_{18}	X_{19}	X_{20}	X_{21}	X_{22}	X_{23}	X_{24}	X_{25}	X_{26}	X_{27}	X_{28}	X_{29}	X_{30}	X_{31}	X_{32}
X_{122}	-4.81	---	---	---	---	---	2.97	---	13.15	2.00	2.00	4.00	3.35	3.02	3.59	4.00
X_{123}	-2.83	---	---	---	---	---	1.75	---	7.72	1.18	1.18	2.35	1.97	1.77	2.11	2.35
X_{124}	-5.44	---	---	---	---	---	3.36	---	14.88	2.27	2.27	4.52	3.79	3.42	4.06	4.52
X_{125}	---	---	---	---	---	---	---	---	2.27	---	---	---	---	---	---	---
X_{128}	-3.55	---	---	---	---	---	2.21	---	---	---	---	---	---	---	---	---
X_{130}	---	1.54	---	---	---	---	---	---	---	---	---	---	---	---	---	---
X_{132}	-7.05	-2.22	-1.01	-1.56	-1.05	---	3.53	-2.19	5.71	---	---	1.74	1.45	1.31	1.56	1.74
X_{133}	---	1.33	---	---	---	---	---	---	---	---	---	---	---	---	---	---
X_{134}	-2.86	---	---	---	---	---	---	---	---	---	---	---	---	---	---	---
X_{135}	---	---	---	-1.29	---	---	---	---	---	---	---	---	---	---	---	---
X_{136}	1.56	---	---	---	---	---	---	---	---	---	---	---	---	---	---	---
X_{142}	1.10	---	---	---	---	---	---	---	---	---	---	---	---	---	---	---
X_{143}	---	-1.52	---	---	---	---	---	---	---	---	---	---	---	---	---	---
X_{144}	---	---	-1.94	-2.04	---	-1.05	---	---	---	---	---	---	---	---	---	---
X_{145}	-2.00	---	---	---	---	---	---	---	---	---	---	---	---	---	---	---
X_{146}	---	---	2.16	2.32	---	1.07	---	---	---	---	---	---	---	---	---	---
X_{147}	---	---	1.08	1.16	---	---	---	---	---	---	---	---	---	---	---	---
X_{148}	---	-1.56	---	---	---	---	---	---	---	---	---	---	---	---	---	---
X_{149}	-2.84	---	---	---	---	---	---	---	1.20	---	---	---	---	---	---	---
X_{152}	14.79	---	---	---	---	---	-9.15	---	-15.99	-2.44	-2.44	-4.86	-4.07	-3.67	-4.36	-4.86
X_{154}	1.86	-1.23	---	---	---	---	---	---	---	---	---	---	---	---	---	---
X_{155}	---	-1.04	---	---	---	---	---	---	---	---	---	---	---	---	---	---
X_{157}	7.12	2.24	1.02	1.57	1.06	---	-3.56	2.21	-5.76	---	---	-1.75	-1.47	-1.32	-1.57	-1.75
X_{158}	---	1.53	---	---	---	---	---	---	---	---	---	---	---	---	---	---
X_{159}	-4.69	---	---	---	---	---	2.90	-2.26	5.07	---	---	1.54	1.29	1.16	1.38	1.54
X_{160}	-9.55	---	---	---	---	---	5.90	---	---	---	---	---	---	---	---	---
X_{165}	7.40	2.33	1.06	1.64	1.11	---	-3.70	2.30	-5.99	---	---	-1.82	-1.53	-1.38	-1.64	-1.82
X_{171}	1.02	---	---	---	---	---	---	---	-2.78	---	---	---	---	---	---	---
X_{172}	---	---	---	---	---	---	---	---	---	-1.00	---	---	---	---	---	---
X_{173}	---	---	---	---	---	---	---	---	---	---	-1.00	---	---	---	---	---
X_{174}	---	---	---	---	---	---	---	---	1.09	---	---	-1.28	---	---	---	---
X_{175}	---	---	---	---	---	---	---	---	---	---	---	---	-1.68	---	---	---
X_{176}	---	---	---	---	---	---	---	---	---	---	---	---	---	-1.51	---	---
X_{177}	---	---	---	---	---	---	---	---	---	---	---	---	---	---	-1.80	---
X_{179}	---	---	---	---	---	---	---	---	-1.09	---	---	---	---	---	---	---
X_{186}	1.02	---	---	---	---	---	---	---	-2.78	---	---	---	---	---	---	-1.84

Table S6. (cont...).

	X_{33}	X_{34}	X_{35}	X_{36}	X_{37}	X_{38}	X_{39}	X_{40}
X_{122}	6.53	7.75	8.63	4.02	4.61	13.62	4.01	17.26
X_{123}	3.84	4.55	5.07	2.36	2.71	---	2.35	10.14
X_{124}	7.39	8.77	9.77	4.55	5.22	9.49	4.54	19.53
X_{125}	1.12	1.34	1.49	---	---	2.35	---	2.97
X_{128}	---	---	---	---	---	-7.91	---	---
X_{130}	2.29	2.34	2.38	---	1.12	1.37	---	4.75
X_{132}	2.81	3.34	3.73	1.70	---	---	1.72	7.45
X_{148}	-2.33	-2.38	-2.41	---	-1.13	-1.39	---	-4.83
X_{152}	-7.66	-9.15	-10.21	-4.89	-5.79	---	-4.88	-20.43
X_{157}	-2.84	-3.38	-3.76	-1.71	---	---	-1.73	-7.52
X_{158}	2.28	2.33	2.37	---	1.11	1.37	---	4.74
X_{159}	2.43	2.90	3.24	1.55	1.84	---	1.55	6.48
X_{161}	---	---	---	---	---	-12.65	---	---
X_{163}	---	---	---	---	---	-15.84	---	---
X_{165}	-2.95	-3.51	-3.91	-1.78	---	---	-1.80	-7.82
X_{174}	1.18	1.40	1.56	---	---	---	---	3.11
X_{176}	-3.24	---	---	---	---	---	---	---
X_{177}	---	-3.86	---	---	---	---	---	---
X_{179}	-1.18	-1.40	-1.56	---	---	---	---	-3.11
X_{180}	-2.60	-2.60	-2.60	---	---	---	---	-5.19
X_{181}	2.60	2.60	---	---	---	---	---	---
X_{183}	---	---	2.60	---	---	---	---	5.19
X_{186}	---	---	-3.97	-1.85	-2.08	---	-1.85	-7.94
