# Supplementary material for: Mathematical Modeling and Validation of the Ergosterol Pathway in Saccharomyces cerevisiae
Source: PLoS One. 2011 Dec 14;6(12):e28344. doi: 10.1371/journal.pone.0028344 (PMC3237449; doi:10.1371/journal.pone.0028344)
Supplement: Table S7 — SL-E rate constant sensitivities (of metabolites) with magnitudes greater than one. First row and column correspond to dependent variables and rate constants respectively. The key for the variable names is given in Tables S1 and S2. Rows not present have only values smaller than one. (PDF) [file pone.0028344.s009.pdf]

Table S7. *SL-E rate constant sensitivities (metabolites) with magnitudes greater than 1.*

	X_1	X_2	X_3	X_4	X_5	X_6	X_7	X_8	X_9	X_{10}	X_{11}	X_{12}	X_{13}	X_{14}	X_{15}	X_{16}
α_1	1.00	2.95	1.86	2.99	3.71	4.27	1.89	2.83	---	---	---	---	---	---	---	---
α_2	---	2.96	1.86	3.00	3.72	4.29	1.90	2.84	---	---	---	---	---	---	---	---
α_3	---	---	2.02	---	---	---	1.84	3.04	---	---	---	---	---	---	---	---
α_4	---	---	---	1.04	---	---	---	---	---	---	---	---	---	---	---	---
α_5	---	---	---	---	1.55	1.79	---	---	---	---	---	---	---	---	---	---
α_6	---	---	---	---	---	1.23	---	---	---	---	---	---	---	---	---	---
α_7	---	---	---	---	---	---	2.08	1.95	---	---	---	---	---	---	---	---
α_8	---	---	---	---	---	---	---	2.70	---	---	---	---	---	---	---	---
α_9	---	---	---	---	---	---	---	---	---	6.09	---	---	---	1.06	---	-12.53
α_{10}	---	---	---	---	---	---	---	---	---	3.94	---	---	---	---	---	-4.38
α_{11}	---	---	---	---	---	-1.04	---	---	---	7.16	2.30	---	-1.00	2.39	---	-16.18
α_{12}	---	---	1.44	---	---	---	1.38	2.21	---	7.53	2.42	---	-1.14	2.34	---	-16.76
α_{13}	1.04	3.08	1.92	3.12	3.87	4.46	1.96	2.93	---	---	---	---	5.20	---	---	---
α_{14}	---	---	---	---	---	---	---	---	---	---	---	---	---	2.38	---	-5.12
α_{15}	---	---	---	---	---	---	---	3.07	---	---	---	---	---	---	1.02	---
α_{16}	---	---	---	---	---	---	---	3.41	1.00	-3.29	---	---	---	---	1.01	7.79
α_{23}	---	-1.86	---	-1.88	-2.98	-3.44	---	---	---	---	---	---	---	---	---	---
α_{24}	---	-4.05	---	-4.11	-6.54	-7.54	---	---	---	---	---	---	---	---	---	---
α_{25}	---	-2.64	---	-2.67	-4.27	-4.92	---	---	---	---	---	---	---	---	---	---
α_{32}	---	---	---	---	-1.03	-1.19	---	---	---	---	---	---	---	---	---	---
α_{38}	---	-1.75	---	-1.78	-2.84	-3.27	---	---	---	---	---	---	---	---	---	---

Table S7 (cont...).

	X_{17}	X_{18}	X_{19}	X_{20}	X_{21}	X_{22}	X_{23}	X_{24}	X_{25}	X_{26}	X_{27}	X_{28}	X_{29}	X_{30}	X_{31}	X_{32}
α_1	7.24	2.31	1.05	1.61	1.09	---	-3.63	2.24	-5.85	---	---	-1.78	-1.49	-1.34	-1.60	-1.78
α_2	7.26	2.31	1.05	1.62	1.10	---	-3.64	2.25	-5.88	---	---	-1.79	-1.50	-1.35	-1.60	-1.79
α_3	---	2.48	1.13	1.53	---	---	---	---	---	---	---	---	---	---	---	---
α_5	3.03	---	---	---	---	---	---	---	---	---	---	---	---	---	---	---
α_6	2.04	---	---	---	---	---	---	---	---	---	---	---	---	---	---	---
α_7	---	1.59	---	---	---	---	---	---	---	---	---	---	---	---	---	---
α_8	---	2.13	1.04	1.38	---	---	---	---	---	---	---	---	---	---	---	---
α_9	-1.96	---	---	---	---	---	---	---	1.07	---	---	---	---	---	---	---
α_{11}	-2.84	---	---	---	---	---	---	---	1.26	---	---	---	---	---	---	---
α_{12}	---	1.80	---	1.09	---	---	---	---	---	---	---	---	---	---	---	---
α_{13}	7.57	2.39	1.09	1.67	1.13	---	-3.79	2.36	-6.14	---	---	-1.87	-1.56	-1.41	-1.67	-1.87
α_{14}	-1.11	---	---	---	---	---	---	---	---	---	---	---	---	---	---	---
α_{15}	---	---	2.03	2.13	---	1.10	---	---	---	---	---	---	---	---	---	---
α_{16}	---	---	2.16	2.32	---	1.07	---	---	---	---	---	---	---	---	---	---
α_{17}	2.01	---	---	---	---	---	---	---	---	---	---	---	---	---	---	---
α_{18}	---	2.51	1.23	---	1.67	---	---	---	---	---	---	---	---	---	---	---
α_{19}	---	---	1.45	---	---	1.38	---	---	---	---	---	---	---	---	---	---
α_{20}	---	---	---	2.01	---	---	---	---	---	---	---	---	---	---	---	---

G.4.- Table S7 (cont...).

	X_{17}	X_{18}	X_{19}	X_{20}	X_{21}	X_{22}	X_{23}	X_{24}	X_{25}	X_{26}	X_{27}	X_{28}	X_{29}	X_{30}	X_{31}	X_{32}
α_{21}	---	---	---	---	1.98	---	---	---	---	---	---	---	---	---	---	---
α_{22}	---	---	---	---	---	2.39	---	---	---	---	---	---	---	---	---	---
α_{23}	-5.85	---	---	---	---	---	3.62	-2.24	5.85	---	---	1.78	1.49	1.34	1.60	1.78
α_{24}	-12.90	---	---	---	---	---	7.97	---	8.50	1.30	1.30	2.58	2.16	1.95	2.32	2.58
α_{25}	-8.44	---	---	---	---	---	5.21	---	23.05	3.51	3.51	7.01	5.87	5.29	6.29	7.01
α_{26}	-1.02	---	---	---	---	---	---	---	2.78	---	---	1.84	1.54	1.39	1.66	1.84
α_{27}	-1.02	---	---	---	---	---	---	---	2.78	---	---	1.84	1.54	1.39	1.66	1.84
α_{28}	-1.02	---	---	---	---	---	---	---	2.78	---	---	1.84	1.54	1.39	1.66	1.84
α_{29}	-1.02	---	---	---	---	---	---	---	2.78	---	---	---	1.54	1.39	1.66	1.84
α_{30}	-1.06	---	---	---	---	---	---	---	2.89	---	---	---	---	1.45	1.73	1.92
α_{31}	-1.15	---	---	---	---	---	---	---	3.15	---	---	---	---	---	1.88	2.09
α_{32}	-2.05	---	---	---	---	---	1.26	---	5.59	---	---	---	---	---	---	3.71
α_{35}	---	---	---	---	---	---	---	---	1.06	---	---	---	---	---	---	---
α_{36}	-1.65	---	---	-2.52	-2.54	-2.60	1.02	---	4.50	---	---	---	---	---	---	2.99
α_{37}	---	---	---	-1.75	-1.76	-1.79	---	---	2.29	---	---	---	---	---	---	1.52
α_{38}	-5.61	---	---	---	---	---	3.47	---	15.33	2.34	2.34	4.66	3.90	3.52	4.18	4.66
α_{39}	-1.65	---	---	-1.38	-1.41	-1.47	1.02	---	4.50	---	---	---	---	---	---	2.99

Table S7 **(cont...).**

	X_{33}	X_{34}	X_{35}	X_{36}	X_{37}	X_{38}	X_{39}	X_{40}
α_1	-2.85	-3.40	-3.79	-1.74	---	---	-1.76	-7.58
α_2	-2.86	-3.41	-3.80	-1.74	---	---	-1.77	-7.61
α_3	---	---	---	---	1.48	---	---	---
α_8	---	---	---	---	1.31	---	---	---
α_{11}	---	---	---	---	---	---	---	1.13
α_{12}	2.69	2.74	2.79	---	1.31	1.61	---	5.57
α_{13}	-3.02	-3.59	-4.00	-1.82	---	---	-1.84	-8.01
α_{23}	2.85	3.39	3.78	1.79	2.15	---	1.78	7.56
α_{24}	4.25	5.04	5.60	2.61	3.34	---	2.60	11.21
α_{25}	11.45	13.59	15.13	7.05	8.08	---	7.03	30.26
α_{26}	3.00	3.56	3.97	1.85	2.08	---	1.85	7.94
α_{27}	3.00	3.56	3.97	1.85	2.08	---	1.85	7.94
α_{28}	3.00	3.56	3.97	1.85	2.08	---	1.85	7.94
α_{29}	3.00	3.56	3.97	1.85	2.08	---	1.85	7.94
α_{30}	3.13	3.72	4.14	1.93	2.17	---	1.93	8.28
α_{31}	---	4.04	4.50	2.10	2.36	---	2.09	9.00
α_{32}	---	---	8.00	3.73	4.19	---	3.72	15.99
α_{34}	2.73	---	---	---	---	---	---	---
α_{35}	---	3.09	---	---	---	---	---	1.10
α_{36}	---	---	6.75	---	---	---	---	13.51
α_{37}	---	---	6.44	7.08	5.64	---	5.03	12.87
α_{38}	---	---	3.27	3.62	3.34	---	2.57	6.55
α_{39}	7.61	9.04	10.06	4.68	5.38	15.87	4.67	20.12
α_{40}	---	---	6.44	5.04	4.50	---	5.01	12.87
