# Supplementary material for: Mathematical Modeling and Validation of the Ergosterol Pathway in Saccharomyces cerevisiae
Source: PLoS One. 2011 Dec 14;6(12):e28344. doi: 10.1371/journal.pone.0028344 (PMC3237449; doi:10.1371/journal.pone.0028344)
Supplement: Table S8 — SL-E logarithmic gains (of fluxes) with magnitudes greater than one. First row and column correspond to the fluxes and the independent variables respectively. The key for the variable names is given in Tables S1 and S2. Rows not presented have only values smaller than one. (PDF) [file pone.0028344.s010.pdf]

Table S8. *SL-E logarithmic gains (fluxes) with magnitudes greater than 1***.**

	V_1	V_2	V_3	V_4	V_5	V_6	V_7	V_8	V_9	V_{10}	V_{11}	V_{12}	V_{13}	V_{14}	V_{15}	V_{16}
X_{122}	---	---	---	-1.47	---	-2.34	---	---	---	---	---	---	---	---	---	---
X_{123}	---	---	---	---	---	-1.37	---	---	---	---	---	---	---	---	---	---
X_{124}	---	---	---	-1.66	---	-2.64	---	---	---	---	---	---	---	---	---	---
X_{125}	---	---	---	-1.08	---	-1.73	---	---	---	---	---	---	---	---	---	---
X_{128}	---	---	---	---	---	---	---	1.06	2.05	3.21	1.74	---	---	---	---	---
X_{130}	---	---	---	-2.79	-1.41	-3.46	---	-1.22	---	---	---	---	---	---	---	---
X_{132}	---	---	---	---	---	-1.39	---	---	---	---	---	---	---	---	---	---
X_{134}	---	---	---	---	---	---	---	-1.02	---	---	---	---	---	---	---	---
X_{143}	---	---	---	---	---	---	---	-1.60	---	---	---	---	---	---	---	---
X_{144}	---	---	---	---	---	---	---	1.84	---	-1.64	---	---	---	---	1.00	1.00
X_{146}	---	---	---	---	---	---	---	-1.07	-2.09	-3.26	-1.77	---	---	---	---	---
X_{148}	---	---	---	---	---	---	---	---	2.08	3.24	1.79	---	---	---	---	---
X_{149}	---	---	---	4.62	2.20	7.27	---	-1.23	---	---	---	---	---	---	---	---
X_{152}	---	---	---	2.82	1.43	3.49	---	1.23	---	---	---	---	---	---	---	---
X_{157}	---	---	---	---	---	---	---	1.05	2.05	3.20	1.73	---	---	---	---	---
X_{158}	---	---	---	-1.47	---	-2.30	---	---	---	---	---	---	---	---	---	---
X_{159}	---	---	---	-2.93	-1.39	-4.65	---	---	---	---	---	---	---	---	---	---
X_{160}	**1.01**	**1.01**	**---**	**2.93**	**1.48**	**3.63**	**---**	**1.28**	---	---	---	---	---	---	---	---
X_{165}	---	---	---	-1.47	---	-2.34	---	---	---	---	---	---	---	---	---	---

Table S8 (cont...).

	V_{17}	V_{18}	V_{19}	V_{20}	V_{21}	V_{22}	V_{23}	V_{24}	V_{25}	V_{26}	V_{27}	V_{28}	V_{29}	V_{30}	V_{31}	V_{32}
X_{122}	-2.32	---	---	2.19	2.16	2.07	---	---	---	2.00	2.00	2.00	2.00	2.02	2.12	2.93
X_{123}	-1.36	---	---	1.29	1.27	1.22	---	---	---	1.17	1.17	1.17	1.17	1.19	1.24	1.72
X_{124}	-2.63	1.00	---	2.48	2.44	2.34	---	---	---	2.26	2.26	2.26	2.26	2.29	2.39	3.32
X_{128}	-1.72	---	---	---	---	---	---	---	---	---	---	---	---	---	---	---
X_{130}	---	---	---	1.02	---	---	---	---	---	---	---	---	---	---	---	---
X_{132}	-3.44	---	---	---	---	---	---	---	---	---	---	---	---	---	---	1.25
X_{134}	-1.39	---	---	---	---	---	---	---	---	---	---	---	---	---	---	---
X_{144}	---	-1.04	-1.65	-1.45	---	---	---	---	---	---	---	---	---	---	---	---
X_{146}	---	1.23	1.81	1.62	---	---	---	---	---	---	---	---	---	---	---	---
X_{148}	---	---	---	-1.04	---	---	---	---	---	---	---	---	---	---	---	---
X_{152}	7.22	-1.25	---	-2.85	-2.77	-2.59	---	---	---	-2.43	-2.43	-2.43	-2.43	-2.45	-2.56	-3.56
X_{157}	3.47	---	---	---	---	---	---	---	---	---	---	---	---	---	---	-1.26
X_{158}	---	---	---	1.02	---	---	---	---	---	---	---	---	---	---	---	---
X_{159}	-2.29	---	---	---	---	---	---	---	---	---	---	---	---	---	---	1.13
X_{160}	-4.62	---	---	---	---	---	---	---	---	---	---	---	---	---	---	---
X_{165}	3.61	1.02	---	---	---	---	---	---	---	---	---	---	---	---	---	-1.31

Table S8 **(cont...).**

	V_{33}	V_{34}	V_{35}	V_{36}	V_{37}	V_{38}	V_{39}	V_{40}
X_{122}	2.51	2.98	5.98	4.31	4.56	---	4.01	8.63
X_{123}	1.48	1.75	3.51	2.53	2.68	---	2.35	5.07
X_{124}	2.84	3.38	6.76	4.88	5.16	---	4.54	9.77
X_{125}	---	---	1.03	---	---	---	---	1.49
X_{130}	---	---	1.64	---	1.12	---	---	2.38
X_{132}	1.08	1.29	2.58	1.14	---	---	1.72	3.73
X_{148}	---	---	-1.67	---	-1.13	---	---	-2.41
X_{152}	-2.95	-3.52	-7.07	-5.34	-5.74	---	-4.88	-10.21
X_{157}	-1.09	-1.30	-2.60	-1.15	---	---	-1.73	-3.76
X_{158}	---	---	1.64	---	1.11	---	---	2.37
X_{159}	---	1.12	2.24	1.69	1.82	---	1.55	3.24
X_{165}	-1.14	-1.35	-2.71	-1.20	---	---	-1.80	-3.91
X_{174}	---	---	1.08	---	---	---	---	1.56
X_{176}	-1.25	---	---	---	---	---	---	---
X_{177}	---	-1.48	---	---	---	---	---	---
X_{179}	---	---	-1.08	---	---	---	---	-1.56
X_{180}	---	---	-1.30	---	---	---	---	-2.60
X_{183}	---	---	1.80	---	---	---	---	2.60
X_{186}	---	---	-2.75	-1.96	-2.06	---	-1.85	-3.97
