# Supplementary material for: Mathematical Modeling and Validation of the Ergosterol Pathway in Saccharomyces cerevisiae
Source: PLoS One. 2011 Dec 14;6(12):e28344. doi: 10.1371/journal.pone.0028344 (PMC3237449; doi:10.1371/journal.pone.0028344)
Supplement: Table S9 — SL-E rate constant sensitivities (of fluxes) with magnitudes greater than one. First row and column correspond to dependent variables and rate constants, respectively. The key for the variable names is given in Tables S1 and S2. Rows not presented have only values smaller than one. (PDF) [file pone.0028344.s011.pdf]

Table S9. *SL-E rate constant sensitivities (fluxes) with magnitudes greater than 1.*

	V_1	V_2	V_3	V_4	V_5	V_6	V_7	V_8	V_9	V_{10}	V_{11}	V_{12}	V_{13}	V_{14}	V_{15}	V_{16}
α_1	---	---	---	2.88	1.46	3.56	---	1.26	---	---	---	---	---	---	---	---
α_2	---	---	---	2.89	1.46	3.57	---	1.27	---	---	---	---	---	---	---	---
α_3	---	---	1.03	---	---	---	---	1.66	---	---	---	---	---	---	---	---
α_4	---	---	---	1.00	---	---	---	---	---	---	---	---	---	---	---	---
α_5	---	---	---	---	1.12	1.49	---	---	---	---	---	---	---	---	---	---
α_6	---	---	---	---	---	1.03	---	---	---	---	---	---	---	---	---	---
α_7	---	---	---	---	---	---	1.06	1.06	---	---	---	---	---	---	---	---
α_8	---	---	---	---	---	---	---	1.47	---	---	---	---	---	---	---	---
α_9	---	---	---	---	---	---	---	---	1.95	3.05	---	---	---	---	---	---
α_{10}	---	---	---	---	---	---	---	---	---	1.97	---	---	---	---	---	---
α_{11}	---	---	---	---	---	---	---	---	2.29	3.58	1.97	---	---	---	---	---
α_{12}	---	---	---	---	---	---	---	1.24	2.41	3.76	2.04	1.01	---	---	---	---
α_{13}	1.03	1.03	---	3.00	1.52	3.71	---	1.31	---	---	---	---	1.00	---	---	---
α_{14}	---	---	---	---	---	---	---	---	---	---	---	---	---	1.00	---	---
α_{15}	---	---	---	---	---	---	---	1.68	---	---	---	---	---	---	---	---
α_{16}	---	---	---	---	---	---	---	1.84	---	-1.64	---	---	---	---	1.00	1.00
α_{23}	---	---	---	-1.81	---	-2.86	---	---	---	---	---	---	---	---	---	---
α_{24}	---	---	---	-3.95	-1.87	-6.28	---	---	---	---	---	---	---	---	---	---
α_{25}	---	---	---	-2.57	-1.22	-4.10	---	1.48	---	---	---	---	---	---	---	---
α_{38}	---	---	---	-1.71	---	-2.72	---	---	---	---	---	---	---	---	---	---

Table S9 (cont...).

	V_{17}	V_{18}	V_{19}	V_{20}	V_{21}	V_{22}	V_{23}	V_{24}	V_{25}	V_{26}	V_{27}	V_{28}	V_{29}	V_{30}	V_{31}	V_{32}
α_1	3.54	1.01	---	---	---	---	---	---	---	---	---	---	---	---	---	-1.28
α_2	3.55	1.01	---	---	---	---	---	---	---	---	---	---	---	---	---	-1.28
α_3	---	1.42	---	1.51	1.23	---	---	---	---	---	---	---	---	---	---	---
α_5	1.47	---	---	---	---	---	---	---	---	---	---	---	---	---	---	---
α_6	1.01	---	---	---	---	---	---	---	---	---	---	---	---	---	---	---
α_8	---	1.25	---	1.35	1.06	---	---	---	---	---	---	---	---	---	---	---
α_{12}	---	1.07	---	1.20	---	---	---	---	---	---	---	---	---	---	---	---
α_{13}	3.70	1.04	---	---	---	---	---	---	---	---	---	---	---	---	---	-1.34
α_{15}	---	1.09	1.73	1.52	---	1.00	---	---	---	---	---	---	---	---	---	---
α_{16}	---	1.23	1.81	1.62	---	---	---	---	---	---	---	---	---	---	---	---
α_{18}	---	1.47	1.04	---	1.26	---	---	---	---	---	---	---	---	---	---	---
α_{19}	---	---	1.24	---	---	---	---	---	---	---	---	---	---	---	---	---
α_{20}	---	---	---	1.43	---	---	---	---	---	---	---	---	---	---	---	---
α_{21}	---	---	---	---	1.44	---	---	---	---	---	---	---	---	---	---	---
α_{22}	---	---	---	---	---	1.21	---	---	---	---	---	---	---	---	---	---
α_{23}	-2.84	---	---	1.08	1.04	---	---	---	---	---	---	---	---	---	---	1.30
α_{24}	-6.24	---	---	1.78	1.69	1.47	---	---	---	1.29	1.29	1.29	1.29	1.31	1.37	1.90
α_{25}	-4.07	1.55	1.23	3.84	3.78	3.63	---	---	1.00	3.50	3.50	3.50	3.50	3.54	3.71	5.14
α_{26}	---	---	---	---	---	---	---	---	---	---	---	---	---	---	---	1.35
α_{27}	---	---	---	---	---	---	---	---	---	---	---	---	---	---	---	1.35
α_{28}	---	---	---	---	---	---	---	---	---	---	---	---	---	---	---	1.35
α_{29}	---	---	---	---	---	---	---	---	---	---	---	---	---	---	---	1.35
α_{30}	---	---	---	1.00	---	---	---	---	---	---	---	---	---	---	1.02	1.41
α_{31}	---	---	---	1.09	1.08	1.06	---	---	---	---	---	---	---	---	1.11	1.53
α_{32}	---	---	---	1.94	1.92	1.89	---	---	---	---	---	---	---	---	---	2.72
α_{36}	---	---	---	1.56	1.55	1.52	---	---	---	---	---	---	---	---	---	2.19
α_{38}	---	---	---	---	---	---	---	---	---	---	---	---	---	---	---	1.11
α_{39}	-2.71	1.03	---	2.55	2.51	2.41	---	---	---	2.33	2.33	2.33	2.33	2.35	2.47	3.42

Table S9 (cont...).

	V_{33}	V_{34}	V_{35}	V_{36}	V_{37}	V_{38}	V_{39}	V_{40}
α_1	-1.10	-1.31	-2.62	-1.16	---	---	-1.76	-3.79
α_2	-1.10	-1.31	-2.63	-1.17	---	---	-1.77	-3.80
α_3	---	---	---	---	1.49	---	---	---
α_8	---	---	---	---	1.32	---	---	---
α_{12}	1.03	1.06	1.93	---	1.31	---	---	2.79
α_{13}	-1.16	-1.38	-2.77	-1.23	---	---	-1.84	-4.00
α_{23}	1.10	1.30	2.62	1.97	2.13	---	1.78	3.78
α_{24}	1.63	1.94	3.88	2.97	3.31	---	2.60	5.60
α_{25}	4.41	5.23	10.48	7.56	8.00	---	7.03	15.13
α_{26}	1.16	1.37	2.75	1.96	2.06	---	1.85	3.97
α_{27}	1.16	1.37	2.75	1.96	2.06	---	1.85	3.97
α_{28}	1.16	1.37	2.75	1.96	2.06	---	1.85	3.97
α_{29}	1.16	1.37	2.75	1.96	2.06	---	1.85	3.97
α_{30}	1.20	1.43	2.87	2.05	2.15	---	1.93	4.14
α_{31}	---	1.56	3.12	2.23	2.33	---	2.09	4.50
α_{32}	---	---	5.54	3.95	4.14	---	3.72	8.00
α_{33}	1.05	---	---	---	---	---	---	---
α_{34}	---	1.19	---	---	---	---	---	---
α_{35}	---	---	4.68	---	---	---	---	6.75
α_{36}	---	---	4.46	6.34	5.53	---	5.03	6.44
α_{37}	---	---	2.27	2.95	3.26	---	2.57	3.27
α_{38}	2.93	3.48	6.97	5.02	5.32	1.00	4.67	10.06
α_{39}	---	---	4.46	4.76	4.43	---	5.01	6.44
α_{40}	---	---	2.34	---	---	---	---	4.38
