# Supplementary material for: Mathematical Modeling and Validation of the Ergosterol Pathway in Saccharomyces cerevisiae
Source: PLoS One. 2011 Dec 14;6(12):e28344. doi: 10.1371/journal.pone.0028344 (PMC3237449; doi:10.1371/journal.pone.0028344)
Supplement: Table S10 — Simulation of the effect of statins in the SL-E model. The total mass of ergosterol (Erg.) and complex sphingolipid (CS) sub-populations in the flux balanced SL-E model is presented under different experimental conditions, after 120 minutes. Wild type condition is represented by the basal values; the two experimental conditions correspond to: A) fold changes with respect to their corresponding basal values after decreasing the specific activity of Thiolase/Synthase and HMG Reductase to 10%, 1%, and 0.01%. B) Same conditions as in A but with an simultaneous decrease to 1% the activity of IPC Synthase (X 133). Complex sphingolipids: IPC, MIPC, and M(IP)2C in Golgi compartment (Golgi CS), or plasma membrane (PM). Last row correspond at the result of divide the DIM ergosterol, and the PM CS's. (PDF) [file pone.0028344.s012.pdf]

Table. S10. *Statins SL-E model simulation.*

		WT		Thiolase/Synthase, X_{171}			HMG Reductase, X_{172}		
		Basal value*		Change after 2 hrs			Change after 2 hrs		
				10%	1%	0.02%	10%	1%	0.02%
**ER Erg.**	X_{32}	9.51	*A*	1.62	0.37	0.18	9.51	9.48	6.99
			B	1.62	0.35	0.16	10.08	10.04	7.26
**PM Outer Erg.**	X_{36}	4.76	*A*	1.46	0.86	0.77	4.75	4.72	3.19
			B	1.37	0.70	0.60	5.66	5.63	3.71
**DIM Erg.**	X_{37}	42.79	*A*	9.23	4.83	4.24	42.76	42.45	24.20
			B	3.71	2.07	1.86	17.18	17.08	9.91
**PM Inner Erg.**	X_{39}	47.55	*A*	12.24	6.24	5.36	47.53	47.30	32.86
			B	11.98	5.49	4.55	53.69	53.44	36.08
**Golgi CS**	$X_8+X_{18}+X_{19}$	0.25	*A*	0.26	0.28	0.29	0.25	0.25	0.22
			B	0.006	0.005	0.005	0.011	0.011	0.008
**PM CS**	$X_{20}+X_{21}+X_{22}$	2.25	*A*	1.74	1.41	1.33	2.25	2.26	2.17
			B	0.73	0.69	0.68	0.76	0.76	0.78
**DIM Erg. / PM CS**	X_{37} / $(X_{20}+ X_{21}+X_{22})$	18.98	*A*	5.31	3.42	3.19	18.97	18.81	11.14
			B	5.11	3.01	2.76	22.65	22.47	12.67

(*) For units see Table S1.

Under conditions *A* in Table S10 the DIM associated ergosterol and the complex sphingolipids are more sensitive to reductions in the specific activity of thiolase/synthase compared with equivalent reductions in the specific activity of HMG reductase. To observe equivalent changes in the ergosterol and complex sphingolipids under the HMG reductase condition it is necessary to reduce its activity to 0.02% of its basal value. This decrease in HMG reductase seems realistic, because it is in line with the nanomolar statins inhibition constants reported for this enzyme [1,2].

The last two rows in Table S10 show that synergistic effects with IPC synthase (condition *B*) influence the relationship between DIM Erg. And PM CS in a magnitude of less than 20% when compared with the corresponding experiment from condition *A*. This phenomenon occurs in spite of the notable reductions in DIM Ergosterol (X_{37}) and PM CS ($X_{20}+X_{21}+X_{22}$), which is observed for the synergistic condition *B*. This synergistic homeostatic effect of DIM Erg and PM CS is problaby due to the observed proportional decrease in both the DIM Erg and PM CS.Finally, the small increase in

complex sphingolipids observed in the Golgi (Table S10) in the case of reduced thiolase/synthase (between 3 to 8%) is not observed when the activity of the HMG Reductase is reduced.

References.

1. Holdgate GA, Ward WH, McTaggart F (2003) Molecular mechanism for inhibition of 3-hydroxy-3-methylglutaryl CoA (HMG-CoA) reductase by rosuvastatin. Biochem Soc Trans 31: 528-531.
2. Istvan ES, Deisenhofer J (2001) Structural mechanism for statin inhibition of HMG-CoA reductase. Science 292: 1160-1164.
