# Supplementary material for: Mathematical Modeling and Validation of the Ergosterol Pathway in Saccharomyces cerevisiae
Source: PLoS One. 2011 Dec 14;6(12):e28344. doi: 10.1371/journal.pone.0028344 (PMC3237449; doi:10.1371/journal.pone.0028344)
Supplement: Table S11 — Variables equivalence from Plas [48] into Matlab® [49] . (PDF) [file pone.0028344.s013.pdf]

Table S11. *Variables equivalence from Plas[1] into Matlab [2].*

Variables				
Total Mass	Labeled		Unlabeled	
Plas & Matlab	Matlab	Plas	Matlab	Plas
X_{1}	X_{41}	L_{1}	X_{81}	U_{1}
X_{2}	X_{42}	L_{2}	X_{82}	U_{2}
X_{3}	X_{43}	L_{3}	X_{83}	U_{3}
X_{4}	X_{44}	L_{4}	X_{84}	U_{4}
X_{5}	X_{45}	L_{5}	X_{85}	U_{5}
X_{6}	X_{46}	L_{6}	X_{86}	U_{6}
X_{7}	X_{47}	L_{7}	X_{87}	U_{7}
X_{8}	X_{48}	L_{8}	X_{88}	U_{8}
X_{9}	X_{49}	L_{9}	X_{89}	U_{9}
X_{10}	X_{50}	L_{10}	X_{90}	U_{10}
X_{11}	X_{51}	L_{11}	X_{91}	U_{11}
X_{12}	X_{52}	L_{12}	X_{92}	U_{12}
X_{13}	X_{53}	L_{13}	X_{93}	U_{13}
X_{14}	X_{54}	L_{14}	X_{94}	U_{14}
X_{15}	X_{55}	L_{15}	X_{95}	U_{15}
X_{16}	X_{56}	L_{16}	X_{96}	U_{16}
X_{17}	X_{57}	L_{17}	X_{97}	U_{17}
X_{18}	X_{58}	L_{18}	X_{98}	U_{18}
X_{19}	X_{59}	L_{19}	X_{99}	U_{19}
X_{20}	X_{60}	L_{20}	X_{100}	U_{20}
X_{21}	X_{61}	L_{21}	X_{101}	U_{21}
X_{22}	X_{62}	L_{22}	X_{102}	U_{22}
X_{23}	X_{63}	L_{23}	X_{103}	U_{23}
X_{24}	X_{64}	L_{24}	X_{104}	U_{24}
X_{25}	X_{65}	L_{25}	X_{105}	U_{25}
X_{26}	X_{66}	L_{26}	X_{106}	U_{26}
X_{27}	X_{67}	L_{27}	X_{107}	U_{27}
X_{28}	X_{68}	L_{28}	X_{108}	U_{28}
X_{29}	X_{69}	L_{29}	X_{109}	U_{29}
X_{30}	X_{70}	L_{30}	X_{110}	U_{30}
X_{31}	X_{71}	L_{31}	X_{111}	U_{31}
X_{32}	X_{72}	L_{32}	X_{112}	U_{32}
X_{33}	X_{73}	L_{33}	X_{113}	U_{33}
X_{34}	X_{74}	L_{34}	X_{114}	U_{34}
X_{35}	X_{75}	L_{35}	X_{115}	U_{35}
X_{36}	X_{76}	L_{36}	X_{116}	U_{36}
X_{37}	X_{77}	L_{37}	X_{117}	U_{37}
X_{38}	X_{78}	L_{38}	X_{118}	U_{38}
X_{39}	X_{79}	L_{39}	X_{119}	U_{39}
X_{40}	X_{80}	L_{40}	X_{120}	U_{40}
X_{124}	X_{162}	L_{124}	X_{185}	U_{124}
X_{125}	X_{187}	L_{125}	X_{188}	U_{125}
X_{158}	X_{169}	L_{158}	X_{184}	U_{158}

References

1. Ferreira AEN (2005) PLAS. 1.2.0.120. http://enzymology.fc.ul.pt/software.htm. Accessed 2011 Nov 15.
2. MATLAB (2010). 7.10.0.499 ed. Natick, Massachusetts: The MathWorks Inc.
