# Supplementary material for: Mathematical Modeling and Validation of the Ergosterol Pathway in Saccharomyces cerevisiae
Source: PLoS One. 2011 Dec 14;6(12):e28344. doi: 10.1371/journal.pone.0028344 (PMC3237449; doi:10.1371/journal.pone.0028344)
Supplement: Equations S1 — GMA mass equations for pools of total masses. In these equations, a superscript indicates fluxes for the complex sphingolipids that were not represented in Figs. 1 and 2 of the manuscript due to lack of space. (PDF) [file pone.0028344.s014.pdf]

Equations S1. *GMA mass balance equations for pools of total masses.*

$$dX_1/dt = v_{12,1} - v_{1,2}$$
$$dX_2/dt = (v_{1,2} + v_{3,2} + v_{4,2}) - (v_{2,3} + v_{2,4} + v_{2,5})$$
$$dX_3/dt = (v_{2,3} + v_{8,3} + v_{18,3} + v_{19,3}) - (v_{3,2} + v_{3,7} + v_{3,8})$$
$$dX_4/dt = v_{2,4} - (v_{4,2} + v_{4,17})$$
$$dX_5/dt = (v_{2,5} + v_{6,5} + v_{7,5}) - (v_{5,6} + v_{5,7})$$
$$dX_6/dt = v_{5,6} - (v_{6,5} + v_{6,17})$$
$$dX_7/dt = (v_{3,7} + v_{5,7} + v_{8,7} + v_{18,7} + v_{19,7}) - (v_{7,5} + v_{7,8} + v_{7,43})$$
$$dX_8/dt = (v_{3,8} + v_{7,8} + v_{20,8}) - (v_{8,3} + v_{8,7} + v_{8,18} + v_{8,20})$$
$$dX_9/dt = v_{11,9} - (v_{9,10} + v_{9,15})$$
$$dX_{10}/dt = v_{9,10} - v_{10,56}$$
$$dX_{11}/dt = v_{12,11} - (v_{11,9} + v_{11,14})$$
$$dX_{12}/dt = (v_{4,17} + v_{6,17} + v_{24,12} + v_{33,30} + v_{34,31} + v_{35,32} + v_{40,39} + v_{158,12}) \\ - (v_{12,1} + v_{12,11} + v_{12,23} + v_{12,148} + v_{30,33} + v_{31,34} + v_{32,35})$$
$$dX_{13}/dt = v_{37,13} - (v_{12,1} + v_{9,10} + v_{13,32})$$
$$dX_{14}/dt = (v_{3,8} + v_{7,8} + v_{11,14} + v_{18,19}) - (v_{14,142} + v_{14,145})$$
$$dX_{15}/dt = v_{9,15} - (v_{3,8} + v_{7,8} + v_{15,44} + v_{18,19})$$
$$dX_{16}/dt = v_{47,16} - v_{9,15}$$
$$dX_{17}/dt = (v_{4,17} + v_{6,17}) - v_{14,145}$$
$$dX_{18}/dt = (v_{8,18} + v_{21,18}) - (v_{18,3} + v_{18,7} + v_{18,19} + v_{18,21})$$
$$dX_{19}/dt = (v_{18,19} + v_{22,19}) - (v_{19,3} + v_{19,7} + v_{19,22})$$
$$dX_{20}/dt = v_{8,20} - v_{20,8}$$
$$dX_{21}/dt = v_{18,21} - v_{21,18}$$
$$dX_{22}/dt = v_{19,22} - v_{22,19}$$
$$dX_{23}/dt = v_{12,23} - (v_{2,3} + v_{5,7})$$
$$dX_{24}/dt = v_{25,24} - (v_{12,23} + v_{24,12})$$
$$dX_{25}/dt = (v_{38,25} + v_{124,25}) - (v_{24,12} + v_{25,24})$$
$$dX_{26}/dt = v_{25,26} - v_{26,27}$$
$$dX_{27}/dt = v_{26,27} - v_{27,28}$$
$$dX_{28}/dt = v_{27,28} - (v_{28,29} + v_{28,179})$$
$$dX_{29}/dt = v_{28,29} - v_{29,30}$$
$$dX_{30}/dt = (v_{29,30} + v_{33,30}) - (v_{30,31} + v_{30,33})$$
$$dX_{31}/dt = (v_{30,31} + v_{34,31}) - (v_{31,32} + v_{31,34})$$
$$dX_{32}/dt = (v_{31,32} + v_{35,32} + v_{37,32} + v_{41,32}) - (v_{32,35} + v_{32,37} + v_{32,41} + v_{32,186})$$
$$dX_{33}/dt = v_{30,33} - v_{33,30}$$

$$dX_{34}/dt = v_{31,34} - v_{34,31}$$
$$dX_{35}/dt = (v_{32,35} + v_{40,35}) - (v_{35,32} + v_{35,40})$$
$$dX_{36}/dt = (v_{37,36} + v_{39,36}) - (v^{a}_{36,37} + v^{b}_{36,37} + v^{c}_{36,37} + v_{36,39}) \; ^{*}$$
$$dX_{37}/dt = (v_{32,37} + v^{a}_{36,37} + v^{b}_{36,37} + v^{c}_{36,37}) - (v_{37,32} + v_{37,36})$$
$$dX_{38}/dt = (v_{124,38} + v_{125,38}) - v_{38,25}$$
$$dX_{39}/dt = (v_{42,39} + v_{36,39} + v_{40,39}) - (v_{39,42} + v_{39,36})$$
$$dX_{40}/dt = v_{35,40} - (v_{40,35} + v_{40,39})$$

(*) A superscript indicates fluxes for the complex sphingolipids that were not represented in Figs. 1 and 2 of the manuscript due to lack of space.
