# Supplementary material for: Mathematical Modeling and Validation of the Ergosterol Pathway in Saccharomyces cerevisiae
Source: PLoS One. 2011 Dec 14;6(12):e28344. doi: 10.1371/journal.pone.0028344 (PMC3237449; doi:10.1371/journal.pone.0028344)
Supplement: Equations S2 — Power-Law terms from Equations S1 . In these equations, a superscript indicates fluxes for the complex sphingolipids that were not represented in Figs. 1 and 2 of the manuscript due to lack of space. (PDF) [file pone.0028344.s015.pdf]

Equations S2. *Power-Law terms for Equations S1.*

$$v_{1,2} = 650105.24 X_1^{0.993} X_{127}$$
$$v_{2,3} = 5831662.55 X_2^{0.964} X_{23}^{0.527} X_{134}$$
$$v_{2,4} = 2266.06 X_2^{0.974} X_{128}^{0.02} X_{136}$$
$$v_{2,5} = 5810 X_2^{0.5} X_{154}$$
$$v_{3,2} = 3057.42 X_3^{0.5} X_{129}$$
$$v_{3,7} = 24083.44 X_3^{0.499} X_{154}$$
$$v_{3,8} = 10.85 X_2^{-0.0033} X_3^{0.973} X_5^{-0.0242} X_{15}^{1.685} X_{133}$$
$$v_{4,2} = 12.01 X_4^{0.968} X_{141}$$
$$v_{4,17} = 2224.47 X_4^{0.96} X_{150}$$
$$v_{5,6} = 69009.45 X_5^{0.96} X_{128}^{0.022} X_{136}$$
$$v_{5,7} = 90147.19 X_5^{0.8} X_{23}^{0.527} X_{134}$$
$$v_{6,5} = 218.22 X_6^{0.861} X_{141}$$
$$v_{6,17} = 36106.04 X_6^{0.829} X_{150}$$
$$v_{7,5} = 2547.85 X_7^{0.5} X_{153}$$
$$v_{7,8} = 10.49 X_2^{-0.0033} X_5^{-0.023} X_7^{0.962} X_{15}^{1.685} X_{133}$$
$$v_{7,143} = 37712.92 X_7^{0.499} X_{143}$$
$$v_{8,3} = 89.10 X_8^{0.972} X_{164}$$
$$v_{8,7} = 297.0 X_8^{0.972} X_{151}$$
$$v_{8,18} = 675.14 X_8^{0.5} X_{135}$$
$$v_{8,20} = 0.0203 X_8^{0.5} X_{32}^{0.5}$$
$$v_{9,10} = 893.15 X_2^{-0.0065} X_5^{-0.013} X_9^{0.993} X_{11}^{0.235} X_{13}^{0.000206} X_{14}^{-0.59} X_{15}^{0.088} X_{16}^{-0.27} X_{138}$$
$$v_{9,15} = 118.77 X_9^{0.971} X_{16}^{0.0033} X_{126}$$
$$v_{10,156} = 94651.3 X_{10}^{0.5} X_{156}$$
$$v_{11,9} = 2512.72 X_{11}^{0.994} X_{140}$$
$$v_{11,14} = 94.55 X_2^{-0.02} X_5^{-0.05} X_9^{0.326} X_{11}^{0.423} X_{15}^{0.248} X_{139}$$
$$v_{12,1} = 174268.87 X_{12}^{0.998} X_{13}^{0.198} X_{157}$$
$$v_{12,11} = 114103.68 X_{10}^{0.149} X_{12} X_{149}$$
$$v_{12,23} = 16834.72 X_{12} X_{24}^{0.415} X_{159}$$
$$v_{12,148} = 294.08 X_{12} X_{148}$$
$$v_{13,132} = 2351.877 X_{13}^{0.199} X_{132}$$
$$v_{14,142} = 912.65 X_{14}^{0.426} X_{142}$$
$$v_{14,145} = 2.01 X_{14}^{0.234} X_{17}^{0.5} X_{145}$$
$$v_{15,144} = 62.50 X_{15}^{0.944} X_{128}^{0.0033} X_{144}$$
$$v_{20,8} = 0.00303 X_{20}^{0.5} X_{37}^{0.5}$$
$$v_{21,18} = 0.00258 X_{21}^{0.5} X_{37}^{0.5}$$
$$v_{22,19} = 0.00276 X_{22}^{0.5} X_{37}^{0.5}$$
$$v_{24,12} = 476.86 X_{24}^{0.131} X_{25}^{0.0079} X_{152}$$
$$v_{25,24} = 17.25 X_{12}^{-0.045} X_{23}^{-0.158} X_{25}^{0.044} X_{128}^{0.375} X_{160}$$
$$v_{25,26} = 16.37 X_{25}^{0.304} X_{32}^{-0.5} X_{171}$$
$$v_{26,27} = 10524.59 X_{26}^{0.997} X_{172}$$
$$v_{27,28} = 96427.49 X_{27}^{0.997} X_{173}$$
$$v_{28,29} = 23509.54 X_{28}^{0.5} X_{174}$$
$$v_{28,179} = 1837.28 X_{28}^{0.5} X_{179}$$
$$v_{29,30} = 73968.17 X_{29}^{0.597} X_{175}$$
$$v_{30,31} = 8.703 \times 10^7 X_{30}^{0.662} X_{176}$$
$$v_{30,33} = 3443.22 X_{12}^{0.999} X_{30}^{0.827} X_{181}$$
$$v_{31,32} = 12425.09 X_{31}^{0.557} X_{177}$$
$$v_{31,34} = 4075.35 X_{12}^{0.999} X_{31}^{0.827} X_{181}$$
$$v_{32,35} = 4169.10 X_{12}^{0.999} X_{32}^{0.827} X_{183}$$
$$v_{32,37} = 1.471 X_8^{0.5} X_{18}^{0.5} X_{19}^{0.5} X_{32}^{0.5}$$
$$v_{32,39} = 0.295 X_{32}$$
$$v_{32,186} = 861.02 X_{32}^{0.5} X_{186}$$
$$v_{33,30} = 9.967 X_{33}^{0.385} X_{180}$$
$$v_{34,31} = 21.42 X_{34}^{0.385} X_{180}$$
$$v_{35,32} = 36.71 X_{35}^{0.385} X_{180}$$
$$v_{35,40} = 0.0165 X_{35}$$
$$v_{36,37}^{a} = 0.193 X_{20}^{1.23} X_{36}^{1.23} \; *$$
$$v_{36,37}^{b} = 0.1803 X_{21}^{1.23} X_{36}^{1.23}$$
$$v_{36,37}^{c} = 0.343 X_{22}^{1.23} X_{36}^{1.23}$$
$$v_{36,39} = 0.613 X_{36}$$
$$v_{37,36} = 0.0349 X_{37}$$
$$v_{38,25} = 0.00923 X_{12}^{-0.111} X_{38}^{0.063} X_{128}^{0.5} X_{161}^{0.798} X_{163}$$
$$v_{39,32} = 0.055 X_{39}$$
$$v_{39,36} = 0.0314 X_{39}$$
$$v_{40,35} = 0.319 X_{40}^{0.5}$$
$$v_{40,39} = 59552.08 X_{40}^{0.385} X_{182}$$

$$v_{18,3} = 152.55 X_{18}^{0.929} X_{164}$$
$$v_{18,7} = 355.94 X_{18}^{0.929} X_{151}$$
$$v_{18,19} = 6.57 X_{15}^{1.685} X_{18}^{0.5} X_{155}$$
$$v_{18,21} = 0.0173 X_{18}^{0.5} X_{32}^{0.5}$$
$$v_{19,3} = 5371.92 X_{19}^{0.995} X_{164}$$
$$v_{19,7} = 8694.45 X_{19}^{0.995} X_{151}$$
$$v_{19,22} = 0.0351 X_{19}^{0.5} X_{32}^{0.5}$$

$$v_{124,25} = 1.226 X_{123} X_{124}^{0.741}$$
$$v_{124,38} = 0.0072 X_{122} X_{124}^{0.696}$$
$$v_{125.,38} = 0.0013 X_{125}^{1.04}$$
$$v_{137,13} = 351.12 X_{131} X_{137}^{0.166}$$
$$v_{147,16} = 57.205 X_{146} X_{147}^{0.5}$$
$$v_{158,12} = 23067.03 X_{158}^{0.997} X_{130}$$
$$v_{166,13} = 801.71 X_{165} X_{166}^{0.0398}$$

(*) A superscript associated with v indicates fluxes for the complex sphingolipids that were not represented in Figs 1 and 2 of the manuscript due to lack of space.
