# Supplementary material for: Mathematical Modeling and Validation of the Ergosterol Pathway in Saccharomyces cerevisiae
Source: PLoS One. 2011 Dec 14;6(12):e28344. doi: 10.1371/journal.pone.0028344 (PMC3237449; doi:10.1371/journal.pone.0028344)
Supplement: Equations S3 — GMA mass balance equations for labeled pools. The structures of the mass balance equations are similar to the Equations in S8, but for clarity we use instead of and instead of . (PDF) [file pone.0028344.s016.pdf]

Equations S3. *GMA mass balance equations for labelad pools.*

$$dL_1/dt = l_{12,1} - l_{1,2}$$
$$dL_2/dt = \left(l_{1,2} + l_{3,2} + l_{4,2}\right) - \left(l_{2,3}^{a} + l_{2,4} + l_{2,5}\right)\ ^{*}$$
$$dL_3/dt = \left(l_{2,3}^{c} + l_{8,3} + l_{18,3} + l_{19,3}\right) - \left(l_{3,2} + l_{3,7} + l_{3,8}^{a}\right)$$
$$dL_4/dt = l_{2,4} - \left(l_{4,2} + l_{4,17}\right)$$
$$dL_5/dt = \left(l_{2,5} + l_{6,5} + l_{7,5}\right) - \left(l_{5,6} + l_{5,7}^{a}\right)$$
$$dL_6/dt = l_{5,6} - \left(l_{6,5} + l_{6,17}\right)$$
$$dL_7/dt = \left(l_{3,7} + l_{5,7}^{c} + l_{8,7} + l_{18,7} + l_{19,7}\right) - \left(l_{7,5} + l_{7,8}^{a} + l_{7,43}\right)$$
$$dL_8/dt = \left(l_{3,8}^{c} + l_{7,8}^{c} + l_{20,8}\right) - \left(l_{8,3} + l_{8,7} + l_{8,18} + l_{8,20}\right)$$
$$dL_9/dt = l_{11,9} - \left(l_{9,10} + l_{9,15}\right)$$
$$dL_{10}/dt = l_{9,10} - l_{10,56}$$
$$dL_{11}/dt = l_{12,11} - \left(l_{11,9} + l_{11,14}\right)$$
$$dL_{12}/dt = \left(l_{4,17} + l_{6,17} + l_{24,12}^{c}\right) - \left(l_{12,1} + l_{12,11} + l_{12,23}^{a} + l_{12,148}\right)$$
$$dL_{14}/dt = \left(l_{3,8}^{b} + l_{7,8}^{b} + l_{11,14} + l_{18,19}^{b}\right) - \left(l_{14,142} + l_{14,145}\right)$$
$$dL_{15}/dt = l_{9,15} - \left(l_{3,8}^{b} + l_{7,8}^{b} + l_{15,44} + l_{18,19}^{b}\right)$$
$$dL_{17}/dt = \left(l_{4,17} + l_{6,17}\right) - l_{14,145}$$
$$dL_{18}/dt = \left(l_{8,18} + l_{21,18}\right) - \left(l_{18,3} + l_{18,7} + l_{18,19}^{a} + l_{18,21}\right)$$
$$dL_{19}/dt = \left(l_{18,19}^{c} + l_{22,19}\right) - \left(l_{19,3} + l_{19,7} + l_{19,22}\right)$$
$$dL_{20}/dt = l_{8,20} - l_{20,8}$$
$$dL_{21}/dt = l_{18,21} - l_{21,18}$$
$$dL_{22}/dt = l_{19,22} - l_{22,19}$$
$$dL_{23}/dt = l_{12,23}^{c} - \left(l_{2,3}^{b} + l_{5,7}^{b}\right)$$
$$dL_{24}/dt = l_{25,24} - \left(l_{12,23}^{b} + l_{24,12}^{a}\right)$$
$$dL_{25}/dt = \left(l_{38,25} + l_{124,25}\right) - \left(l_{24,12}^{b} + l_{25,24}\right)$$
$$dL_{26}/dt = l_{25,26} - l_{26,27}$$
$$dL_{27}/dt = l_{26,27} - l_{27,28}$$
$$dL_{28}/dt = l_{27,28} - \left(l_{28,29} + l_{28,179}\right)$$
$$dL_{29}/dt = l_{28,29} - l_{29,30}$$
$$dL_{30}/dt = \left(l_{29,30} + l_{33,30}\right) - \left(l_{30,31} + l_{30,33}\right)$$
$$dL_{31}/dt = \left(l_{30,31} + l_{34,31}\right) - \left(l_{31,32} + l_{31,34}\right)$$
$$dL_{32}/dt = \left(l_{31,32} + l_{35,32} + l_{37,32} + l_{39,32}\right) - \left(l_{32,35} + l_{32,37} + l_{32,39} + l_{32,186}\right)$$
$$dL_{33}/dt = l_{30,33} - l_{33,30}$$
$$dL_{34}/dt = l_{31,34} - l_{34,31}$$
$$dL_{35}/dt = \left(l_{32,35} + l_{40,35}\right) - \left(l_{35,32} + l_{35,40}\right)$$
$$dL_{36}/dt = \left(l_{37,36} + l_{39,36}\right) - \left(l_{36,37}^{a} + l_{36,37}^{b} + l_{36,37}^{c} + l_{36,39}\right)$$

$$dL_{37}/dt = \left(l_{32,37} + l^{a}_{36,37} + l^{b}_{36,37} + l^{c}_{36,37}\right) - \left(l_{37,32} + l_{37,36}\right)$$
$$dL_{38}/dt = l_{125,38} - l_{38,25}$$
$$dL_{39}/dt = \left(l_{42,39} + l_{36,39} + l_{40,39}\right) - \left(l_{39,32} + l_{39,36}\right)$$
$$dL_{40}/dt = l_{35,40} - \left(l_{40,35} + l_{40,39}\right)$$

(*) A superscript indicates deviations from the total pool; the method automatically computes equations for the labeled fractions.

Tracer method: Mathematical approach to modeling tracer dynamics

To characterize the movement and distribution of a radioactive tracer throughout the sphingolipid pathway, simulations were performed using a method specifically developed for this purpose [1]. This method models the dynamics of total (labeled plus unlabeled) metabolites and in a second and third set of equations distributes material between labeled and unlabeled fractions (see [1] and main manuscript for details of the method).

Tracer equations

In Equations S3 and S4, the labeled metabolites serine (L_{13}) and inositol (L_{16}) are not included because they never receive label in radioactive acetate (L_{125}) experiments. For the same reason, the time independent variables palmitate (L_{158}) and pyruvate (L_{124}) are not included in Equations S3 and S4.

References.

1. Voit EO, Alvarez-Vasquez F, Sims KJ (2004) Analysis of dynamic labeling data. Math Biosci 191: 83-99.
