# Supplementary material for: Mathematical Modeling and Validation of the Ergosterol Pathway in Saccharomyces cerevisiae
Source: PLoS One. 2011 Dec 14;6(12):e28344. doi: 10.1371/journal.pone.0028344 (PMC3237449; doi:10.1371/journal.pone.0028344)
Supplement: Equations S4 — Terms from Equations S3 . A superscript indicates deviations from the total pool; the method automatically computes equations for the labeled fractions. (PDF) [file pone.0028344.s017.pdf]

Equations S4. *Power-Law terms in Equations S3*

$l_{1,2} = v_{1,2} \times L_1 / X_1$

$l^a_{2,3} = v_{2,3} \times L_2 / X_2$ *

$l^b_{2,3} = v_{2,3} \times L_{23} / X_{23}$

$l^c_{2,3} = v_{2,3} \times \left(L_2 U_{23} + U_2 L_{23} + L_2 L_{23}\right) / \left(X_2 X_{23}\right)$

$l_{2,4} = v_{2,4} \times L_2 / X_2$

$l_{2,5} = v_{2,5} \times L_2 / X_2$

$l_{3,2} = v_{3,2} \times L_3 / X_3$

$l_{3,7} = v_{3,7} \times L_3 / X_3$

$l^a_{3,8} = v_{3,8} \times L_3 / X_3$

$l^b_{3,8} = v_{3,8} \times L_{15} / X_{15}$

$l^c_{3,8} = v_{3,8} \times \left(L_3 U_{15} + U_3 L_{15} + L_3 L_{15}\right) / \left(X_3 X_{15}\right)$

$l_{4,2} = v_{4,2} \times L_4 / X_4$

$l_{4,17} = v_{4,17} \times L_4 / X_4$

$l_{5,6} = v_{5,6} \times L_5 / X_5$

$l^a_{5,7} = v_{5,7} \times L_5 / X_5$

$l^b_{5,7} = v_{5,7} \times L_{23} / X_{23}$

$l^c_{5,7} = v_{5,7} \times \left(L_5 U_{23} + U_5 L_{23} + L_5 L_{23}\right) / \left(X_5 X_{23}\right)$

$l_{6,5} = v_{6,5} \times L_6 / X_6$

$l_{6,17} = v_{6,17} \times L_6 / X_6$

$l_{7,5} = v_{7,5} \times L_7 / X_7$

$l^a_{7,8} = v_{7,8} \times L_7 / X_7$

$l^b_{7,8} = v_{7,8} \times L_{15} / X_{15}$

$l^c_{7,8} = v_{7,8} \times \left(L_7 U_{15} + U_7 L_{15} + L_7 L_{15}\right) / \left(X_7 X_{15}\right)$

$l_{7,143} = v_{7,143} \times L_7 / X_7$

$l_{8,3} = v_{8,3} \times L_8 / X_8$

$l_{8,7} = v_{8,7} \times L_8 / X_8$

$l_{8,18} = v_{8,18} \times L_8 / X_8$

$l_{8,20} = v_{8,20} \times L_8 / X_8$

$l_{9,10} = v_{9,10} \times L_9 / X_9$

$l_{9,15} = v_{9,15} \times L_9 / X_9$

$l_{10,156} = v_{10,156} \times L_{10} / X_{10}$

$l_{11,9} = v_{11,9} \times L_{11} / X_{11}$

$l_{18,3} = v_{18,3} \times L_{18} / X_{18}$

$l_{18,7} = v_{18,7} \times L_{18} / X_{18}$

$l^a_{18,19} = v_{18,19} \times L_{18} / X_{18}$

$l^b_{18,19} = v_{18,19} \times L_{15} / X_{15}$

$l^c_{18,19} = v_{18,19} \times \left(L_{18} U_{15} + U_{18} L_{15} + L_{18} L_{15}\right) / \left(X_{18} X_{15}\right)$

$l_{18,21} = v_{18,21} \times L_{18} / X_{18}$

$l_{19,3} = v_{19,3} \times L_{19} / X_{19}$

$l_{19,7} = v_{19,7} \times L_{19} / X_{19}$

$l_{19,22} = v_{19,22} \times L_{19} / X_{19}$

$l_{20,8} = v_{20,8} \times L_{20} / X_{20}$

$l_{21,18} = v_{21,18} \times L_{21} / X_{21}$

$l_{22,19} = v_{22,19} \times L_{22} / X_{22}$

$l^a_{24,12} = v_{24,12} \times L_{24} / X_{24}$

$l^b_{24,12} = v_{24,12} \times L_{25} / X_{25}$

$l^c_{24,12} = v_{24,12} \times \left(L_{24} U_{25} + U_{24} L_{25} + L_{24} L_{25}\right) / \left(X_{24} X_{25}\right)$

$l_{25,24} = v_{25,24} \times L_{25} / X_{25}$

$l_{25,26} = v_{25,26} \times L_{25} / X_{25}$

$l_{26,27} = v_{26,27} \times L_{26} / X_{26}$

$l_{27,28} = v_{27,28} \times L_{27} / X_{27}$

$l_{28,29} = v_{28,29} \times L_{28} / X_{28}$

$l_{28,179} = v_{28,179} \times L_{28} / X_{28}$

$l_{29,30} = v_{29,30} \times L_{29} / X_{29}$

$l_{30,31} = v_{30,31} \times L_{30} / X_{30}$

$l_{30,33} = v_{30,33} \times L_{30} / X_{30}$ †

$l_{31,32} = v_{31,32} \times L_{31} / X_{31}$

$l_{31,34} = v_{31,34} \times L_{31} / X_{31}$ †

$l_{32,35} = v_{32,35} \times L_{32} / X_{32}$ †

$l_{32,37} = v_{32,37} \times L_{32} / X_{32}$

$l_{32,39} = v_{32,39} \times L_{32} / X_{32}$

$l_{32,186} = v_{32,186} \times L_{32} / X_{32}$

$l_{33,30} = v_{33,30} \times L_{33} / X_{33}$

$l_{34,31} = v_{34,31} \times L_{34} / X_{34}$

$$l_{11,14} = v_{11,14} \times L_{11} / X_{11}$$
$$l_{12,1} = v_{12,1} \times L_{12} / X_{12}$$
$$l_{12,11} = v_{12,11} \times L_{12} / X_{12}$$
$$l^{a}_{12,23} = v_{12,23} \times L_{12} / X_{12}$$
$$l^{b}_{12,23} = v_{12,23} \times \left(L_{24} / X_{24} \right)$$
$$l^{c}_{12,23} = v_{12,23} \times \left(L_{12} U_{24} + U_{12} L_{24} + L_{12} L_{24} \right) / \left(X_{12} X_{24} \right)$$
$$l_{12,148} = v_{12,148} \times L_{12} / X_{12}$$
$$l_{14,142} = v_{14,142} \times L_{14} / X_{14}$$
$$l_{14,145} = v_{14,145} \times L_{14} / X_{14}$$
$$l_{15,144} = v_{15,144} \times L_{15} / X_{15}$$
$$l_{35,32} = v_{35,32} \times L_{35} / X_{35}$$
$$l_{35,40} = v_{35,40} \times L_{35} / X_{35}$$
$$l^{a}_{36,37} = v^{a}_{36,37} \times L_{36} / X_{36}$$
$$l^{b}_{36,37} = v^{b}_{36,37} \times L_{36} / X_{36}$$
$$l^{c}_{36,37} = v^{c}_{36,37} \times L_{36} / X_{36}$$
$$l_{36,39} = v_{36,39} \times L_{36} / X_{36}$$
$$l_{38,25} = v_{38,25} \times L_{38} / X_{38}$$
$$l_{40,35} = v_{40,35} \times L_{40} / X_{40}$$
$$l_{40,39} = v_{40,39} \times L_{40} / X_{40}$$
$$l_{125,38} = v_{125.38} \times L_{125} / X_{125}$$

(†) The contribution of labeled palmitoyl-CoA (X_{12}) was not included. See text for explanations.

(*) A superscript indicates deviations from the total pool; the method automatically computes equations for the labeled fractions.
