# Supplementary material for: Mathematical Modeling and Validation of the Ergosterol Pathway in Saccharomyces cerevisiae
Source: PLoS One. 2011 Dec 14;6(12):e28344. doi: 10.1371/journal.pone.0028344 (PMC3237449; doi:10.1371/journal.pone.0028344)
Supplement: Equations S5 — GMA mass balance equations for unlabeled pools. The structures of the mass balance equations are similar to the Equations in S8, but for clarity we use instead of and instead of. (PDF) [file pone.0028344.s018.pdf]

Equations S5. *GMA mass balance equations for unlabeled pools.*

$$dU_1/dt = u_{12,1} - u_{1,2}$$
$$dU_2/dt = (u_{1,2} + u_{3,2} + u_{4,2}) - (u^a_{2,3} + u_{2,4} + u_{2,5})\ ^*$$
$$dU_3/dt = (u^c_{2,3} + u_{8,3} + u_{18,3} + u_{19,3}) - (u_{3,2} + u_{3,7} + u^a_{3,8})$$
$$dU_4/dt = u_{2,4} - (u_{4,2} + u_{4,17})$$
$$dU_5/dt = (u_{2,5} + u_{6,5} + u_{7,5}) - (u_{5,6} + u^a_{5,7})$$
$$dU_6/dt = u_{5,6} - (u_{6,5} + u_{6,17})$$
$$dU_7/dt = (u_{3,7} + u^c_{5,7} + u_{8,7} + u_{18,7} + u_{19,7}) - (u_{7,5} + u^a_{7,8} + u_{7,43})$$
$$dU_8/dt = (u^c_{3,8} + u^c_{7,8} + u_{20,8}) - (u_{8,3} + u_{8,7} + u_{8,18} + u_{8,20})$$
$$dU_9/dt = u_{11,9} - (u_{9,10} + u_{9,15})$$
$$dU_{10}/dt = u_{9,10} - u_{10,56}$$
$$dU_{11}/dt = u_{12,11} - (u_{11,9} + u_{11,14})$$
$$dU_{12}/dt = (u_{4,17} + u_{6,17} + u_{24,12} + u_{33,30} + u_{34,31} + u_{35,32} + u_{40,39} + u_{158,12})$$
$$- (u_{12,1} + u_{12,11} + u^a_{12,23} + u_{12,148} + u_{30,33} + u_{31,34} + u_{32,35})$$
$$dU_{13}/dt = u_{37,13} - (u_{12,1} + u_{9,10} + u_{13,32})$$
$$dU_{14}/dt = (u^b_{3,8} + u^b_{7,8} + u_{11,14} + u^b_{18,19}) - (u_{14,142} + u_{14,145})$$
$$dU_{15}/dt = u_{9,15} - (u^b_{3,8} + u^b_{7,8} + u_{15,44} + u^b_{18,19})$$
$$dU_{16}/dt = u_{47,16} - u_{9,15}$$
$$dU_{17}/dt = (u_{4,17} + u_{6,17}) - u_{14,145}$$
$$dU_{18}/dt = (u_{8,18} + u_{21,18}) - (u_{18,3} + u_{18,7} + u^a_{18,19} + u_{18,21})$$
$$dU_{19}/dt = (u^c_{18,19} + u_{22,19}) - (u_{19,3} + u_{18,7} + u_{19,22})$$
$$dU_{20}/dt = u_{8,20} - u_{20,8}$$
$$dU_{21}/dt = u_{18,21} - u_{21,18}$$
$$dU_{22}/dt = u_{19,22} - u_{22,19}$$
$$dU_{23}/dt = u^c_{12,23} - (u^b_{2,3} + u^b_{5,7})$$
$$dU_{24}/dt = u_{25,24} - (u^b_{12,23} + u^a_{24,12})$$
$$dU_{25}/dt = u_{62,25} - (u^b_{24,12} + u_{25,24})$$
$$dU_{26}/dt = u_{25,26} - u_{26,27}$$
$$dU_{27}/dt = u_{26,27} - u_{27,28}$$
$$dU_{28}/dt = u_{27,28} - (u_{28,29} + u_{28,179})$$
$$dU_{29}/dt = u_{28,29} - u_{29,30}$$
$$dU_{30}/dt = (u_{29,30} + u_{33,30}) - (u_{30,31} + u_{30,33})$$
$$dU_{31}/dt = (u_{30,31} + u_{34,31}) - (u_{31,32} + u_{31,34})$$
$$dU_{32}/dt = (u_{31,32} + u_{35,32} + u_{37,32} + u_{39,32}) - (u_{32,35} + u_{32,37} + u_{32,41} + u_{32,186})$$

$$dU_{33}/dt = u_{30,33} - u_{33,30}$$
$$dU_{34}/dt = u_{31,34} - u_{34,31}$$
$$dU_{35}/dt = (u_{32,35} + u_{40,35}) - (u_{35,32} + u_{35,40})$$
$$dU_{36}/dt = (u_{37,36} + u_{39,36}) - (u^{a}_{36,37} + u^{b}_{36,37} + u^{c}_{36,37} + u_{36,39})$$
$$dU_{37}/dt = (u_{32,37} + u^{a}_{36,37} + u^{b}_{36,37} + u^{c}_{36,37}) - (u_{37,32} + u_{37,36})$$
$$dU_{38}/dt = (u_{124,38} + u_{125,38}) - u_{38,25}$$
$$dU_{39}/dt = (u_{42,39} + u_{36,39} + u_{40,39}) - (u_{39,32} + u_{39,36})$$
$$dU_{40}/dt = u_{35,40} - (u_{40,35} + u_{40,39})$$

(*) A superscript indicates deviations from the total pools.
