# Supplementary material for: Mathematical Modeling and Validation of the Ergosterol Pathway in Saccharomyces cerevisiae
Source: PLoS One. 2011 Dec 14;6(12):e28344. doi: 10.1371/journal.pone.0028344 (PMC3237449; doi:10.1371/journal.pone.0028344)
Supplement: Equations S6 — Terms from Equations S5 . (PDF) [file pone.0028344.s019.pdf]

Equations S6. *Power-Law terms in Equations S5.*

$u_{1,2} = v_{1,2} \times U_1 / X_1$

$u_{2,3}^a = v_{2,3} \times U_2 / X_2$ *

$u_{2,3}^b = v_{2,3} \times U_{23} / X_{23}$

$u_{2,3}^c = v_{2,3} \times (U_2 U_{23}) / (X_2 X_{23})$

$u_{2,4} = v_{2,4} \times U_2 / X_2$

$u_{2,5} = v_{2,5} \times U_2 / X_2$

$u_{3,2} = v_{3,2} \times U_3 / X_3$

$u_{3,7} = v_{3,7} \times U_3 / X_3$

$u_{3,8}^a = v_{3,8} \times U_3 / X_3$

$u_{3,8}^b = v_{3,8} \times U_{15} / X_{15}$

$u_{3,8}^c = v_{3,8} \times (U_3 U_{15}) / (X_3 X_{15})$

$u_{4,2} = v_{4,2} \times U_4 / X_4$

$u_{4,17} = v_{4,17} \times U_4 / X_4$

$u_{5,6} = v_{5,6} \times U_5 / X_5$

$u_{5,7}^a = v_{5,7} \times U_5 / X_5$

$u_{5,7}^b = v_{5,7} \times U_{23} / X_{23}$

$u_{5,7}^c = v_{5,7} \times (U_5 U_{23}) / (X_5 X_{23})$

$u_{6,5} = v_{6,5} \times U_6 / X_6$

$u_{6,17} = v_{6,17} \times U_6 / X_6$

$u_{7,5} = v_{7,5} \times U_7 / X_7$

$u_{7,8}^a = v_{7,8} \times U_7 / X_7$

$u_{7,8}^b = v_{7,8} \times U_{15} / X_{15}$

$u_{7,8}^c = v_{7,8} \times (U_7 U_{15}) / (X_7 X_{15})$

$u_{7,143} = v_{7,143} \times U_7 / X_7$

$u_{8,3} = v_{8,3} \times U_8 / X_8$

$u_{8,7} = v_{8,7} \times U_8 / X_8$

$u_{8,18} = v_{8,18} \times U_8 / X_8$

$u_{8,20} = v_{8,20} \times U_8 / X_8$

$u_{9,10} = v_{9,10} \times U_9 / X_9$

$u_{9,15} = v_{9,15} \times U_9 / X_9$

$u_{10,156} = v_{10,156} \times U_{10} / X_{10}$

$u_{11,9} = v_{11,9} \times U_{11} / X_{11}$

$u_{11,14} = v_{11,14} \times U_{11} / X_{11}$

$u_{18,19}^a = v_{18,19} \times U_{18} / X_{18}$

$u_{18,19}^b = v_{18,19} \times U_{15} / X_{15}$

$u_{18,19}^c = v_{18,19} \times (U_{18} U_{15}) / (X_{18} X_{15})$

$u_{18,21} = v_{18,21} \times U_{18} / X_{18}$

$u_{19,3} = v_{19,3} \times U_{19} / X_{19}$

$u_{19,7} = v_{19,7} \times U_{19} / X_{19}$

$u_{19,22} = v_{19,22} \times U_{19} / X_{19}$

$u_{20,8} = v_{20,8} \times U_{20} / X_{20}$

$u_{21,18} = v_{21,18} \times U_{21} / X_{21}$

$u_{22,19} = v_{22,19} \times U_{22} / X_{22}$

$u_{24,12}^a = v_{24,12} \times U_{24} / X_{24}$

$u_{24,12}^b = v_{24,12} \times U_{25} / X_{25}$

$u_{24,12}^c = v_{24,12} \times (U_{24} U_{25}) / (X_{24} X_{25})$

$u_{25,24} = v_{25,24} \times U_{25} / X_{25}$

$u_{25,26} = v_{25,26} \times U_{25} / X_{25}$

$u_{26,27} = v_{26,27} \times U_{26} / X_{26}$

$u_{27,28} = v_{27,28} \times U_{27} / X_{27}$

$u_{28,29} = v_{28,29} \times U_{28} / X_{28}$

$u_{28,179} = v_{28,179} \times U_{28} / X_{28}$

$u_{29,30} = v_{29,30} \times U_{29} / X_{29}$

$u_{30,31} = v_{30,31} \times U_{30} / X_{30}$

$u_{30,33} = v_{30,33} \times U_{30} / X_{30}$

$u_{31,32} = v_{31,32} \times U_{31} / X_{31}$

$u_{31,34} = v_{31,34} \times U_{31} / X_{31}$

$u_{32,35} = v_{32,35} \times U_{32} / X_{32}$

$u_{32,37} = v_{32,37} \times U_{32} / X_{32}$

$u_{32,39} = v_{32,39} \times U_{32} / X_{32}$

$u_{32,186} = v_{32,186} \times U_{32} / X_{32}$

$u_{33,30} = v_{33,30} \times U_{33} / X_{33}$

$u_{34,31} = v_{34,31} \times U_{34} / X_{34}$

$u_{35,32} = v_{35,32} \times U_{35} / X_{35}$

$u_{35,40} = v_{35,40} \times U_{35} / X_{35}$

$u_{36,37}^a = v_{36,37}^a \times U_{36} / X_{36}$

$$u_{12,1} = v_{12,1} \times U_{12} / X_{12}$$
$$u_{12,11} = v_{12,11} \times U_{12} / X_{12}$$
$$u^{a}_{12,23} = v_{12,23} \times U_{12} / X_{12}$$
$$u^{b}_{12,23} = v_{12,23} \times (U_{24} / X_{24})$$
$$u^{c}_{12,23} = v_{12,23} \times (U_{12} U_{24}) / (X_{12} X_{24})$$
$$u_{12,148} = v_{12,148} \times U_{12} / X_{12}$$
$$u_{14,142} = v_{14,142} \times U_{14} / X_{14}$$
$$u_{14,145} = v_{14,145} \times U_{14} / X_{14}$$
$$u_{15,144} = v_{15,144} \times U_{15} / X_{15}$$
$$u_{18,3} = v_{18,3} \times U_{18} / X_{18}$$
$$u_{18,7} = v_{18,7} \times U_{18} / X_{18}$$
$$u^{b}_{36,37} = v^{b}_{36,37} \times U_{36} / X_{36}$$
$$u^{c}_{36,37} = v^{c}_{36,37} \times U_{36} / X_{36}$$
$$u_{36,39} = v_{36,39} \times U_{36} / X_{36}$$
$$u_{38,25} = v_{38,25} \times U_{38} / X_{38}$$
$$u_{40,35} = v_{40,35} \times U_{40} / X_{40}$$
$$u_{40,39} = v_{40,39} \times U_{40} / X_{40}$$
$$u_{125.,38} = v_{125.38} \times U_{125} / X_{125}$$
$$u_{124,.25} = v_{124,.25} \times U_{124} / X_{124}$$
$$u_{137,13} = v_{137,13} \times U_{137} / X_{137}$$
$$u_{147,16} = v_{147,16} \times U_{147} / X_{147}$$
$$v_{158,12} = v_{158,12} \times U_{158} / X_{158}$$

(*) A superscript indicates deviations from the total pools.
