# Supplementary material for: Mathematical Modeling and Validation of the Ergosterol Pathway in Saccharomyces cerevisiae
Source: PLoS One. 2011 Dec 14;6(12):e28344. doi: 10.1371/journal.pone.0028344 (PMC3237449; doi:10.1371/journal.pone.0028344)
Supplement: Material S1 — Mathematical Framework. (DOC) [file pone.0028344.s020.doc]

**Supplements**

**Mathematical Modeling and Validation of the Ergosterol Pathway in *Saccharomyces cerevisiae*.**

Fernando Alvarez-Vasquez^*, 1, 4,5^, Howard Riezman^2^, Yusuf A. Hannun^1^, Eberhard O. Voit^3^

***Material S1*.- *Mathematical Framework***

It is straightforward to design a GMA model from a metabolic diagram. In fact, this step can be accomplished with computer software [1]. Each metabolite of interest, *X_i_* (*i* = 1,…*n*), is represented by a differential equation that contains the difference between all influxes and effluxes. The key is that each flux is formulated as a product of power-law functions that contain exclusively those variables that have a direct effect on this flux. Each variable is raised to a power $f_{kij}$, called a kinetic order, that quantifies the strength and direction of the effect, and the product of variables is multiplied with a rate constant $\gamma_{ik}$that quantifies the turn-over rate of the process [2,3]. Thus, each GMA equation has the generic form

$$\frac{dX_{i}}{dt}=\pm\gamma_{i1}\prod_{j=1}^{n+m} X_{j}^{f_{1ij}}\pm\gamma_{i2}\prod_{j=1}^{n+m} X_{j}^{f_{2ij}}\pm...\pm\gamma_{ik}\prod_{j=1}^{n+m} X_{j}^{f_{kij}}$$

[Eq. S1]

While there are *n* equations, one for each *dependent* metabolite variable, the products may include up to *m* further *independent* variables whose concentrations remain constant over time. Typical examples are constant substrates and enzyme activities.

In order to compare the SL-E model results with measured [^14^*C*]-acetate labeling experiments [4], we used a method specifically developed for this purpose [5] that separates labeled from unlabeled portions of each metabolite pool. For example, in the simplest case of a reaction with substrate $T_{i}$ and product $T_{j}$ that is catalyzed by $T_{k}$, the dynamics of labeled substrate *L*_i_ over time are described as

$\frac{dL_{i}}{dt}=\frac{L_{i}}{L_{i}+U_{i}}\cdot\frac{dT_{i}}{dt}$, [Eq. S2],

while the unlabeled material is represented as

$\frac{dU_{i}}{dt}=\frac{U_{i}}{L_{i}+U_{i}}\cdot\frac{dT_{i}}{dt}$. [Eq. S3],

The dynamics of the total pool $T_{i}$ is simply

$\frac{dT_{i}}{dt}=\frac{dL_{i}}{dt}+\frac{dU_{i}}{dt}$. [Eq. S4].

Equations for the labeled and unlabeled products $L_{j}$ and $U_{j}$do not require additional parameters, because they depend directly on the equations for the total substrates as

$$\frac{dL_{j}}{dt}=\frac{L_{i}}{L_{i}+U_{i}}\cdot\frac{dT_{j}}{dt}$$

$\frac{dU_{j}}{dt}=\frac{U_{i}}{L_{i}+U_{i}}\cdot\frac{dT_{j}}{dt}$ . [Eqs. S5],

For multi-substrate reactions, the fractions of labeled (or unlabeled) materials are determined probabilistically by the combinations of the labeled and unlabeled portions of the two substrates. For example, in a bi-substrate reaction with $L_{1}$ and $L_{2}$ as labeled substrates, the dynamics of the labeled product $L_{j}$ is expressed as

$\frac{dL_{j}}{dt}=\frac{\left(L_{1}\cdot U_{2}+U_{1}\cdot L_{2}+L_{1}\cdot L_{2}\right)}{X_{1}\cdot X_{2}}\cdot\frac{dT_{j}}{dt}$ [Eq. S6],

where $X_{1}=L_{1}+U_{1}$ and $X_{2}=L_{2}+U_{2}$, and the unlabeled fraction $U_{j}$ follows the differential equation

$\frac{dU_{j}}{dt}=\frac{U_{1}\cdot U_{2}}{X_{1}\cdot X_{2}}\cdot\frac{dT_{j}}{dt}$. [Eq. S8]

***Fluxes general considerations.***

The GMA Equations S1 to S6 were used for the dynamic simulations.

For total mass steady-state experiments (Tables 1 to 3) the GMA equations were flux balanced. Specifically, the balanced fluxes were directly associated with the complex sphingolipids ( *v*_20,8_ = *v*_8,20_; *v*_21,18_ = *v*_18,21_; *v*_22,19_ = *v*_19,22_ ), with the steryl-ester biosynthesis ( *v*_33,30_ = *v*_30,33_; *v*_34,31_ = *v*_31,34_; *v*_35,32_ = *v*_32,35_; *v*_40,39_ = 0 ), with equal steady-state ergosterol fluxes between ER and PM (*v*_32,39_ = *v*_39,32_) and inside the PM ( *v*_39,36_ = *v*_36,39_; *v*_36,37_ = *v*_37,36_). The flux balance at steady-state implies changes in the earlier determined volumes and/or areas of contact between organelles. However, these adjustments do not change the dynamics of metabolite levels in any significant manner.

Using standard methods of algebra (*e.g.*, [3]), the flux balanced GMA’s equations were aggregated into S-system equations (*Supplement Equations S3 and S5*) for ease of eigenvalue (*Supplement* *Table* *S4*) and sensitivity analysis (*Supplement* *Tables* *S5-9*). This S-system flux aggregation procedure significantly reduces the number of sensitivities to be analyzed, while describing the sphingolipid pathway dynamics as well as the GMA or Michaelis-Menten representations [6].

**References.**

1. Goel G, Chou IC, Voit EO (2006) Biological systems modeling and analysis: a biomolecular technique of the twenty-first century. J Biomol Tech 17: 252-269.

2. Sims KJ, Alvarez-Vasquez F, Voit EO, Hannun YA (2007) A guide to biochemical systems modeling of sphingolipids for the biochemist. Methods Enzymol 432: 319-350.

3. Voit EO (2000) Computational analysis of biochemical systems : a practical guide for biochemists and molecular biologists. New York: Cambridge University Press. xii, 531 p. p.

4. Taylor FR, Parks LW (1978) Metabolic interconversion of free sterols and steryl esters in Saccharomyces cerevisiae. J Bacteriol 136: 531-537.

5. Voit EO, Alvarez-Vasquez F, Sims KJ (2004) Analysis of dynamic labeling data. Math Biosci 191: 83-99.

6. Alvarez-Vasquez F, Sims KJ, Hannun YA, Voit EO (2004) Integration of kinetic information on yeast sphingolipid metabolism in dynamical pathway models. J Theor Biol 226: 265-291.
