# Supplementary material for: Mathematical Modeling and Validation of the Ergosterol Pathway in Saccharomyces cerevisiae
Source: PLoS One. 2011 Dec 14;6(12):e28344. doi: 10.1371/journal.pone.0028344 (PMC3237449; doi:10.1371/journal.pone.0028344)
Supplement: Materials S2 — Modifications of our former sphingolipid (SL) model. (DOC) [file pone.0028344.s021.doc]

***Materials S2*.-** *Modifications of our former sphingolipid (SL) model.*

The previous versions of the sphingolipid model [1-3] were modified as follows:

All recalculated kinetic orders (*a*-*d*) are close to the values presented in Alvarez-Vasquez *et al*. [2] and do not affect the previous model dynamics or conclusions, but can affect numerical values of model sensitivities; the corrections (*a*-*d*) are reflected in Tables S1 and S2.

The SL GMA kinetic orders for *v*_1,2_, *v*_9,15_, *v*_10,56_, *v*_2,4_, and *v*_5,6_ where recalculated as follows:

*a.*- *v*_1,2_ : Kishimoro *et al.* [4] present the K_M_ of KDHS reductase for KDHS in μM. For the kinetic order calculation, this value must first be converted to mol%. For this propose, we used a μM-mol% relationship based on data in Lanterman & Saba, ([5], Fig 2) and Nagiec *et al*. ([6], Fig 2) as follows:

15 μM × 0.38 mol% ÷ 7.7 μM = 0.74 mol%

The kinetic order is then

*f*(1,2,127) = 0.74 ÷ (0.74 + 0.0053) = 0.993

This value is not very different from the published data of 0.999 [2].

*b.*- *v*_10,156_ : The K_M_ of the PS decarboxylase for PS is 9.5 μM [7].

Implementing the μM-mol% relationship from above one obtains:

9.5 μM × 0.38 mol% / 7.7 μM = 0.468 mol%

However, this recalculated K_M_ value led to instability in the SL-E model. Instead we used a K_M_ similar at the substrate concentration something, which is justified by the fact that in many cases the K_m_ is close to the cellular substrate concentration.

As commented by Cleland, the K_M_ of the enzymes is often useful to develop an idea of the physiological substrate concentration because: “While it is not an independent constant, it is a very useful one for the biochemist, since it is also a clue to the physiological level of the substrate. (A substrate concentration around K_M_ utilizes most of the catalytic potential of the enzyme, while still maintaining proportional control; at high substrate levels the rate does not vary with substrate concentration, and one has no control)” [8].

*f*(10,0,120) = 8.4 ÷ (8.4 + 8.4) = 0.5

*c.*- *v*_9,15_: The K_M_ of the PI synthase for CDP-DAG is 66 μM according to Kelley *et al*. [9] . Implementing the same μM-mol% relationship from point *a* one obtains:

66 μM × 0.38 mol% ÷ 7.7 μM = 3.25 mol%

The kinetic order is then

*f*(9,15,126) = 0.976

which is not very different from the published data of 0.912 [2].

*d.*- *v*_2,4_ and *v*_5,6_: The kinetic orders value for *f*(2,4,28) and *f*(5,6,2) is 0.022, which was derived in Alvarez-Vasquez *et al*. [3]. It was obtained using a ATP concentration of 1100 μM and a K_M_ from the sphingoid base kinase for the ATP of 25 μM.

**References**

1. Alvarez-Vasquez F, Hannun YA, Voit EO (2008) Dynamics of Positional Enrichment: Theoretical Development and Application to Carbon Labeling in Zymomonas mobilis. Biochem Eng J 40: 157-174.

2. Alvarez-Vasquez F, Sims KJ, Cowart LA, Okamoto Y, Voit EO, et al. (2005) Simulation and validation of modelled sphingolipid metabolism in Saccharomyces cerevisiae. Nature 433: 425-430.

3. Alvarez-Vasquez F, Sims KJ, Hannun YA, Voit EO (2004) Integration of kinetic information on yeast sphingolipid metabolism in dynamical pathway models. J Theor Biol 226: 265-291.

4. Kishimoto Y (1983) In: Boyer. PD, editor. The Enzymes. 3 ed. New York,: Academic Press. pp. Ch 10.

5. Lanterman MM, Saba JD (1998) Characterization of sphingosine kinase (SK) activity in Saccharomyces cerevisiae and isolation of SK-deficient mutants. Biochem J 332 ( Pt 2): 525-531.

6. Nagiec MM, Skrzypek M, Nagiec EE, Lester RL, Dickson RC (1998) The LCB4 (YOR171c) and LCB5 (YLR260w) genes of Saccharomyces encode sphingoid long chain base kinases. J Biol Chem 273: 19437-19442.

7. Trotter PJ, Voelker DR (1995) Identification of a non-mitochondrial phosphatidylserine decarboxylase activity (PSD2) in the yeast Saccharomyces cerevisiae. J Biol Chem 270: 6062-6070.

8. Cleland WW (1970) The Enzymes. 3 ed: Academic Press, Inc., New York and London. pp. 1-65.

9. Kelley MJ, Bailis AM, Henry SA, Carman GM (1988) Regulation of phospholipid biosynthesis in Saccharomyces cerevisiae by inositol. Inositol is an inhibitor of phosphatidylserine synthase activity. J Biol Chem 263: 18078-18085.
