# Supplementary material for: Mathematical Modeling and Validation of the Ergosterol Pathway in Saccharomyces cerevisiae
Source: PLoS One. 2011 Dec 14;6(12):e28344. doi: 10.1371/journal.pone.0028344 (PMC3237449; doi:10.1371/journal.pone.0028344)
Supplement: Material S3 — SL-E GMA model in Plas [48] format. File used for the dynamic simulations. (DOC) [file pone.0028344.s022.doc]

**Material S3. *SL-E GMA model in Plas format****. File used for the dynamic simulations. It can be run into PLAS [1].*

| /GMA_(FLUXES Unbalanced):  X1' = 174268.8761*X12^.9986438167*X13^.1980849053*X157^1.-650105.2401*X1^.9932909297*X127^1.  X2' = 650105.2401*X1^.9932909297*X127^1.+3057.424256*X3^.5000000001*X129^1.+12.01395274*X4^.9688581315*X141^1.-5831662.556*X2^.9642857143*X23^.5278118802*X134^1.-2266.068147*X2^.9743589746*X128^0.2222222230e-1*X136^1.-5810.000000*X2^.5000000000*X154^1.  X3' = 5831662.556*X2^.9642857143*X23^.5278118802*X134^1.+89.10166634*X8^.9722222223*X164^1.+152.5467333*X18^.9296482412*X164^1.+5371.916275*X19^.9955924294*X164^1.-3057.424256*X3^.5000000001*X129^1.-24083.44972*X3^.4999999998*X154^1.-10.85002492*X3^.9739478958*X15^1.685*X133^1./(X2^0.3358742751e-2*X5^0.2424327076e-1)  X4' = 2266.068147*X2^.9743589746*X128^0.2222222230e-1*X136^1.-12.01395274*X4^.9688581315*X141^1.-2224.471846*X4^.9604829853*X150^1.  X5' = 5810.000000*X2^.5000000000*X154^1.+218.2238068*X6^.8615384613*X141^1.+2547.853547*X7^.5000000002*X153^1.-90147.19843*X5^.8000000000*X23^.5278118802*X134^1.-69009.45118*X5^.9600000000*X128^0.2222222230e-1*X136^1.  X6' = 69009.45118*X5^.9600000000*X128^0.2222222230e-1*X136^1.-218.2238068*X6^.8615384613*X141^1.-36106.04490*X6^.8293838859*X150^1.  X7' = 90147.19843*X5^.8000000000*X23^.5278118802*X134^1.+297.0055545*X8^.9722222223*X151^1.+24083.44972*X3^.4999999998*X154^1.+355.9423777*X18^.9296482412*X151^1.+8694.450414*X19^.9955924294*X151^1.-2547.853547*X7^.5000000002*X153^1.-10.49201131*X7^.9629101285*X15^1.685*X133^1./(X2^0.3320804470e-2*X5^0.2397510852e-1)-37712.92981*X7^.4999999997*X143^1.  X8' = 10.49201131*X7^.9629101285*X15^1.685*X133^1./(X2^0.3320804470e-2*X5^0.2397510852e-1)+10.85002492*X3^.9739478958*X15^1.685*X133^1./(X2^0.3358742751e-2*X5^0.2424327076e-1)+0.3031347641e-2*X20^.5*X37^.5-297.0055545*X8^.9722222223*X151^1.-675.1417734*X8^.5000000000*X135^1.-89.10166634*X8^.9722222223*X164^1.-0.2030671395e-1*X8^.5*X32^.5  X9' = 2512.729767*X11^.9940357859*X140^1.-893.1564995*X9^.9933310569*X11^.235*X13^0.2063074440e-3*X15^0.88e-1*X138^1./(X2^0.6568638313e-2*X5^0.1305154574e-1*X14^.5910831328*X16^.2704826039)-118.7734899*X9^.9716382669*X16^0.3376749592e-2*X126^1.  X10' = 893.1564995*X9^.9933310569*X11^.235*X13^0.2063074440e-3*X15^0.88e-1*X138^1./(X2^0.6568638313e-2*X5^0.1305154574e-1*X14^.5910831328*X16^.2704826039)-94651.30187*X10^.5000000001*X156^1.  X11' = 114103.6810*X10^.1493*X12^1.*X149^1.-94.55645805*X9^.326*X11^.4230769231*X15^.248*X139^1./(X2^0.2071563088e-1*X5^0.5022831050e-1)-2512.729767*X11^.9940357859*X140^1.  X12' = 23067.02893*X130^1.*X158^.9975062347+476.8620195*X24^.1318391563*X25^0.7910349154e-2*X152^1.+2224.471846*X4^.9604829853*X150^1.+36106.04490*X6^.8293838859*X150^1.+9.967272082*X33^.385*X180^1.+21.42776130*X34^.385*X180^1.+36.71477823*X35^.385*X180^1.+59552.08746*X40^.385*X182^1.-174268.8761*X12^.9986438167*X13^.1980849053*X157^1.-114103.6810*X10^.1493*X12^1.*X149^1.-294.0873226*X12^1.*X148^1.-16834.72807*X12^.9999230829*X24^.4157339305*X159^1.-3543.221570*X12^.9998550936*X30^.827*X181^1.-4075.356854*X12^.9998550936*X31^.827*X181^1.-4169.102075*X12^.9998550936*X32^.827*X183^1.  X13' = 351.1209420*X131^1.*X137^.1663551401+801.7109569*X165^1.*X166^0.3984637534e-1-893.1564995*X9^.9933310569*X11^.235*X13^0.2063074440e-3*X15^0.88e-1*X138^1./(X2^0.6568638313e-2*X5^0.1305154574e-1*X14^.5910831328*X16^.2704826039)-174268.8761*X12^.9986438167*X13^.1980849053*X157^1.-2351.877266*X13^.1999999997*X132^1.  X14' = 94.55645805*X9^.326*X11^.4230769231*X15^.248*X139^1./(X2^0.2071563088e-1*X5^0.5022831050e-1)+10.49201131*X7^.9629101285*X15^1.685*X133^1./(X2^0.3320804470e-2*X5^0.2397510852e-1)+10.85002492*X3^.9739478958*X15^1.685*X133^1./(X2^0.3358742751e-2*X5^0.2424327076e-1)+6.574294837*X15^1.685*X18^.5000000001*X155^1.-2.010896717*X14^.2344299609*X17^.5000000003*X145^1.-912.6577402*X14^.4260599794*X142^1.  X15' = 118.7734899*X9^.9716382669*X16^0.3376749592e-2*X126^1.-10.49201131*X7^.9629101285*X15^1.685*X133^1./(X2^0.3320804470e-2*X5^0.2397510852e-1)-10.85002492*X3^.9739478958*X15^1.685*X133^1./(X2^0.3358742751e-2*X5^0.2424327076e-1)-62.50743872*X15^.9441006585*X128^0.3344404739e-2*X144^1.-6.574294837*X15^1.685*X18^.5000000001*X155^1.-14.81189310*X15^.8178844057*X168^1.  X16' = 57.20507542*X146^1.*X147^.5008488966-118.7734899*X9^.9716382669*X16^0.3376749592e-2*X126^1.  X17' = 2224.471846*X4^.9604829853*X150^1.+36106.04490*X6^.8293838859*X150^1.-2.010896717*X14^.2344299609*X17^.5000000003*X145^1.  X18' = 675.1417734*X8^.5000000000*X135^1.+0.2587449087e-2*X21^.5*X37^.5-152.5467333*X18^.9296482412*X164^1.-355.9423777*X18^.9296482412*X151^1.-6.574294837*X15^1.685*X18^.5000000001*X155^1.-0.1733307912e-1*X18^.5*X32^.5  X19' = 6.574294837*X15^1.685*X18^.5000000001*X155^1.+0.2763393752e-2*X22^.5*X37^.5-5371.916275*X19^.9955924294*X164^1.-8694.450414*X19^.9955924294*X151^1.-0.3517226030e-1*X19^.5*X32^.5  X20' = 0.2030671395e-1*X8^.5*X32^.5-0.3031347641e-2*X20^.5*X37^.5  X21' = 0.1733307912e-1*X18^.5*X32^.5-0.2587449087e-2*X21^.5*X37^.5  X22' = 0.3517226030e-1*X19^.5*X32^.5-0.2763393752e-2*X22^.5*X37^.5  X23' = 16834.72807*X12^.9999230829*X24^.4157339305*X159^1.-5831662.556*X2^.9642857143*X23^.5278118802*X134^1.-90147.19843*X5^.8000000000*X23^.5278118802*X134^1.  X24' = 17.25637757*X25^0.4460212912e-1*X128^.3750000000*X160^1./(X12^0.4577407228e-1*X23^.1583280558)-16834.72807*X12^.9999230829*X24^.4157339305*X159^1.-476.8620195*X24^.1318391563*X25^0.7910349154e-2*X152^1.  X25' = 1.226153687*X123^1.*X124^.7411630560+0.9234698783e-2*X38^0.6314511210e-1*X128^.5000000001*X161^.7986577182*X163^1./X12^.111-17.25637757*X25^0.4460212912e-1*X128^.3750000000*X160^1./(X12^0.4577407228e-1*X23^.1583280558)-476.8620195*X24^.1318391563*X25^0.7910349154e-2*X152^1.-16.37319996*X25^.3039999999*X171^1./X32^.5000000000  X26' = 16.37319996*X25^.3039999999*X171^1./X32^.5000000000-10524.59060*X26^.9977827049*X172^1.  X27' = 10524.59060*X26^.9977827049*X172^1.-96427.48842*X27^.9975669099*X173^1.  X28' = 96427.48842*X27^.9975669099*X173^1.-23509.54134*X28^.5*X174^1.-1837.283321*X28^.5000000000*X179^1.  X29' = 23509.54134*X28^.5*X174^1.-73968.17205*X29^.597*X175^1.  X30' = 73968.17205*X29^.597*X175^1.+9.967272082*X33^.385*X180^1.-87031771.36*X30^.662*X176^1.-3543.221570*X12^.9998550936*X30^.827*X181^1.  X31' = 87031771.36*X30^.662*X176^1.+21.42776130*X34^.385*X180^1.-12425.09645*X31^.557*X177^1.-4075.356854*X12^.9998550936*X31^.827*X181^1.  X32' = 12425.09645*X31^.557*X177^1.+0.5563355615e-1*X39+0.2209706584e-1*X20^.5*X21^.5*X22^.5*X37^.5+36.71477823*X35^.385*X180^1.-.2956492539*X32-4169.102075*X12^.9998550936*X32^.827*X183^1.-1.471655572*X8^.5*X18^.5*X19^.5*X32^.5-861.0295570*X32^.5000000000*X186^1.  X33' = 3543.221570*X12^.9998550936*X30^.827*X181^1.-9.967272082*X33^.385*X180^1.  X34' = 4075.356854*X12^.9998550936*X31^.827*X181^1.-21.42776130*X34^.385*X180^1.  X35' = 4169.102075*X12^.9998550936*X32^.827*X183^1.+.3192737733*X40^.5-36.71477823*X35^.385*X180^1.-0.1659444132e-1*X35  X36' = 0.3142262948e-1*X39+0.3491403276e-1*X37-.6137232322*X36-.1939652307*X20^1.23*X36^1.23-.1803401715*X21^1.23*X36^1.23-.3435069860*X22^1.23*X36^1.23  X37' = .1939652307*X20^1.23*X36^1.23+.1803401715*X21^1.23*X36^1.23+.3435069860*X22^1.23*X36^1.23+1.471655572*X8^.5*X18^.5*X19^.5*X32^.5-0.2209706584e-1*X20^.5*X21^.5*X22^.5*X37^.5-0.3491403276e-1*X37  X38' = 0.1397815044e-2*X125^1.04+0.7108537236e-2*X122^1.*X124^.6961178048-0.9234698783e-2*X38^0.6314511210e-1*X128^.5000000001*X161^.7986577182*X163^1./X12^.111  X39' = .2956492539*X32+.6137232322*X36+59552.08746*X40^.385*X182^1.-0.8705618563e-1*X39  X40' = 0.1659444132e-1*X35-.3192737733*X40^.5-59552.08746*X40^.385*X182^1.  L1' = 174268.8761*(L12*U13+U12*L13+L12*L13)*X157^1./(X12^0.13561833e-2*X13^.8019150947)-650105.2401*L1*X127^1./X1^0.67090703e-2  L2' = 650105.2401*L1*X127^1./X1^0.67090703e-2+3057.424256*L3*X129^1./X3^.4999999999+12.01395274*L4*X141^1./X4^0.311418685e-1-5831662.556*L2*X23^.5278118802*X134^1./X2^0.357142857e-1-2266.068147*L2*X128^0.2222222230e-1*X136^1./X2^0.256410254e-1-5810.000000*L2*X154^1./X2^.5000000000  L3' = 5831662.556*(L2*U23+U2*L23+L2*L23)*X134^1./(X2^0.357142857e-1*X23^.4721881198)+89.10166634*L8*X164^1./X8^0.277777777e-1+152.5467333*L18*X164^1./X18^0.703517588e-1+5371.916275*L19*X164^1./X19^0.44075706e-2-3057.424256*L3*X129^1./X3^.4999999999-24083.44972*L3*X154^1./X3^.5000000002-10.85002492*L3*X15^1.685*X133^1./(X3^0.260521042e-1*X2^0.3358742751e-2*X5^0.2424327076e-1)  L4' = 2266.068147*L2*X128^0.2222222230e-1*X136^1./X2^0.256410254e-1-12.01395274*L4*X141^1./X4^0.311418685e-1-2224.471846*L4*X150^1./X4^0.395170147e-1  L5' = 5810.000000*L2*X154^1./X2^.5000000000+218.2238068*L6*X141^1./X6^.1384615387+2547.853547*L7*X153^1./X7^.4999999998-90147.19843*L5*X23^.5278118802*X134^1./X5^.2000000000-69009.45118*L5*X128^0.2222222230e-1*X136^1./X5^0.400000000e-1  L6' = 69009.45118*L5*X128^0.2222222230e-1*X136^1./X5^0.400000000e-1-218.2238068*L6*X141^1./X6^.1384615387-36106.04490*L6*X150^1./X6^.1706161141  L7' = 90147.19843*(L5*U23+U5*L23+L5*L23)*X134^1./(X5^.2000000000*X23^.4721881198)+297.0055545*L8*X151^1./X8^0.277777777e-1+24083.44972*L3*X154^1./X3^.5000000002+355.9423777*L18*X151^1./X18^0.703517588e-1+8694.450414*L19*X151^1./X19^0.44075706e-2-2547.853547*L7*X153^1./X7^.4999999998-10.49201131*L7*X15^1.685*X133^1./(X7^0.370898715e-1*X2^0.3320804470e-2*X5^0.2397510852e-1)-37712.92981*L7*X143^1./X7^.5000000003  L8' = 10.49201131*(L7*U15+U7*L15+L7*L15)*X15^.685*X133^1./(X7^0.370898715e-1*X2^0.3320804470e-2*X5^0.2397510852e-1)+10.85002492*(L3*U15+U3*L15+L3*L15)*X15^.685*X133^1./(X3^0.260521042e-1*X2^0.3358742751e-2*X5^0.2424327076e-1)+0.3031347641e-2*L20*X37^.5/X20^.5-297.0055545*L8*X151^1./X8^0.277777777e-1-675.1417734*L8*X135^1./X8^.5000000000-89.10166634*L8*X164^1./X8^0.277777777e-1-0.2030671395e-1*L8*X32^.5/X8^.5  L9' = 2512.729767*L11*X140^1./X11^0.59642141e-2-893.1564995*L9*X11^.235*X13^0.2063074440e-3*X15^0.88e-1*X138^1./(X9^0.66689431e-2*X2^0.6568638313e-2*X5^0.1305154574e-1*X14^.5910831328*X16^.2704826039)-118.7734899*L9*X16^0.3376749592e-2*X126^1./X9^0.283617331e-1  L10' = 893.1564995*(L9*U13+U9*L13+L9*L13)*X11^.235*X15^0.88e-1*X138^1./(X9^0.66689431e-2*X13^.9997936926*X2^0.6568638313e-2*X5^0.1305154574e-1*X14^.5910831328*X16^.2704826039)-94651.30187*L10*X156^1./X10^.4999999999  L11' = 114103.6810*L12*X10^.1493*X149^1.-94.55645805*L11*X9^.326*X15^.248*X139^1./(X11^.5769230769*X2^0.2071563088e-1*X5^0.5022831050e-1)-2512.729767*L11*X140^1./X11^0.59642141e-2  L12' = 23067.02893*L158*X130^1./X158^0.24937653e-2+476.8620195*(L24*U25+U24*L25+L24*L25)*X152^1./(X24^.8681608437*X25^.9920896508)+2224.471846*L4*X150^1./X4^0.395170147e-1+36106.04490*L6*X150^1./X6^.1706161141+9.967272082*L33*X180^1./X33^.615+21.42776130*L34*X180^1./X34^.615+36.71477823*L35*X180^1./X35^.615+59552.08746*L40*X182^1./X40^.615-174268.8761*L12*X13^.1980849053*X157^1./X12^0.13561833e-2-114103.6810*L12*X10^.1493*X149^1.-294.0873226*L12*X148^1.-16834.72807*L12*X24^.4157339305*X159^1./X12^0.769171e-4-3543.221570*L30*X12^.9998550936*X181^1./X30^.173-4075.356854*L31*X12^.9998550936*X181^1./X31^.173-4169.102075*L32*X12^.9998550936*X183^1./X32^.173  L14' = 94.55645805*L11*X9^.326*X15^.248*X139^1./(X11^.5769230769*X2^0.2071563088e-1*X5^0.5022831050e-1)+10.49201131*(L7*U15+U7*L15+L7*L15)*X15^.685*X133^1./(X7^0.370898715e-1*X2^0.3320804470e-2*X5^0.2397510852e-1)+10.85002492*(L3*U15+U3*L15+L3*L15)*X15^.685*X133^1./(X3^0.260521042e-1*X2^0.3358742751e-2*X5^0.2424327076e-1)+6.574294837*(L18*U15+U18*L15+L18*L15)*X15^.685*X155^1./X18^.4999999999-2.010896717*L14*X17^.5000000003*X145^1./X14^.7655700391-912.6577402*L14*X142^1./X14^.5739400206  L15' = 118.7734899*(L9*U16+U9*L16+L9*L16)*X126^1./(X9^0.283617331e-1*X16^.9966232504)-10.49201131*L15*X15^.685*X7^.9629101285*X133^1./(X2^0.3320804470e-2*X5^0.2397510852e-1)-10.85002492*L15*X15^.685*X3^.9739478958*X133^1./(X2^0.3358742751e-2*X5^0.2424327076e-1)-62.50743872*L15*X128^0.3344404739e-2*X144^1./X15^0.558993415e-1-6.574294837*L15*X15^.685*X18^.5000000001*X155^1.-14.81189310*L15*X168^1./X15^.1821155943  L17' = 2224.471846*L4*X150^1./X4^0.395170147e-1+36106.04490*L6*X150^1./X6^.1706161141-2.010896717*L17*X14^.2344299609*X145^1./X17^.4999999997  L18' = 675.1417734*L8*X135^1./X8^.5000000000+0.2587449087e-2*L21*X37^.5/X21^.5-152.5467333*L18*X164^1./X18^0.703517588e-1-355.9423777*L18*X151^1./X18^0.703517588e-1-6.574294837*L18*X15^1.685*X155^1./X18^.4999999999-0.1733307912e-1*L18*X32^.5/X18^.5  L19' = 6.574294837*(L18*U15+U18*L15+L18*L15)*X15^.685*X155^1./X18^.4999999999+0.2763393752e-2*L22*X37^.5/X22^.5-5371.916275*L19*X164^1./X19^0.44075706e-2-8694.450414*L19*X151^1./X19^0.44075706e-2-0.3517226030e-1*L19*X32^.5/X19^.5  L20' = 0.2030671395e-1*L8*X32^.5/X8^.5-0.3031347641e-2*L20*X37^.5/X20^.5  L21' = 0.1733307912e-1*L18*X32^.5/X18^.5-0.2587449087e-2*L21*X37^.5/X21^.5  L22' = 0.3517226030e-1*L19*X32^.5/X19^.5-0.2763393752e-2*L22*X37^.5/X22^.5  L23' = 16834.72807*(L12*U24+U12*L24+L12*L24)*X159^1./(X12^0.769171e-4*X24^.5842660695)-5831662.556*L23*X2^.9642857143*X134^1./X23^.4721881198-90147.19843*L23*X5^.8000000000*X134^1./X23^.4721881198  L24' = 17.25637757*L25*X128^.3750000000*X160^1./(X25^.9553978709*X12^0.4577407228e-1*X23^.1583280558)-16834.72807*L24*X12^.9999230829*X159^1./X24^.5842660695-476.8620195*L24*X25^0.7910349154e-2*X152^1./X24^.8681608437  L25' = 1.226153687*L124*X123^1./X124^.2588369440+0.9234698783e-2*L38*X128^.5000000001*X161^.7986577182*X163^1./(X38^.9368548879*X12^.111)-17.25637757*L25*X128^.3750000000*X160^1./(X25^.9553978709*X12^0.4577407228e-1*X23^.1583280558)-476.8620195*L25*X24^.1318391563*X152^1./X25^.9920896508-16.37319996*L25*X171^1./(X25^.6960000001*X32^.5000000000)  L26' = 16.37319996*L25*X171^1./(X25^.6960000001*X32^.5000000000)-10524.59060*L26*X172^1./X26^0.22172951e-2  L27' = 10524.59060*L26*X172^1./X26^0.22172951e-2-96427.48842*L27*X173^1./X27^0.24330901e-2  L28' = 96427.48842*L27*X173^1./X27^0.24330901e-2-23509.54134*L28*X174^1./X28^.5-1837.283321*L28*X179^1./X28^.5000000000  L29' = 23509.54134*L28*X174^1./X28^.5-73968.17205*L29*X175^1./X29^.403  L30' = 73968.17205*L29*X175^1./X29^.403+9.967272082*L33*X180^1./X33^.615-87031771.36*L30*X176^1./X30^.338-3543.221570*L30*X12^.9998550936*X181^1./X30^.173  L31' = 87031771.36*L30*X176^1./X30^.338+21.42776130*L34*X180^1./X34^.615-12425.09645*L31*X177^1./X31^.443-4075.356854*L31*X12^.9998550936*X181^1./X31^.173  L32' = 12425.09645*L31*X177^1./X31^.443+0.5563355615e-1*L39+0.2209706584e-1*L37*X20^.5*X21^.5*X22^.5/X37^.5+36.71477823*L35*X180^1./X35^.615-.2956492539*L32-4169.102075*L32*X12^.9998550936*X183^1./X32^.173-1.471655572*L32*X8^.5*X18^.5*X19^.5/X32^.5-861.0295570*L32*X186^1./X32^.5000000000  L33' = 3543.221570*L30*X12^.9998550936*X181^1./X30^.173-9.967272082*L33*X180^1./X33^.615  L34' = 4075.356854*L31*X12^.9998550936*X181^1./X31^.173-21.42776130*L34*X180^1./X34^.615  L35' = 4169.102075*L32*X12^.9998550936*X183^1./X32^.173+.3192737733*L40/X40^.5-36.71477823*L35*X180^1./X35^.615-0.1659444132e-1*L35  L36' = 0.3142262948e-1*L39+0.3491403276e-1*L37-.6137232322*L36-.1939652307*L36*X36^.23*X20^1.23-.1803401715*L36*X36^.23*X21^1.23-.3435069860*L36*X36^.23*X22^1.23  L37' = .1939652307*L36*X36^.23*X20^1.23+.1803401715*L36*X36^.23*X21^1.23+.3435069860*L36*X36^.23*X22^1.23+1.471655572*L32*X8^.5*X18^.5*X19^.5/X32^.5-0.2209706584e-1*L37*X20^.5*X21^.5*X22^.5/X37^.5-0.3491403276e-1*L37  L38' = 0.1397815044e-2*L125*X125^0.4e-1+0.7108537236e-2*L124*X122^1./X124^.3038821952-0.9234698783e-2*L38*X128^.5000000001*X161^.7986577182*X163^1./(X38^.9368548879*X12^.111)  L39' = .2956492539*L32+.6137232322*L36+59552.08746*L40*X182^1./X40^.615-0.8705618563e-1*L39  L40' = 0.1659444132e-1*L35-.3192737733*L40/X40^.5-59552.08746*L40*X182^1./X40^.615  U1' = 174268.8761*U12*U13*X157^1./(X12^0.13561833e-2*X13^.8019150947)-650105.2401*U1*X127^1./X1^0.67090703e-2  U2' = 650105.2401*U1*X127^1./X1^0.67090703e-2+3057.424256*U3*X129^1./X3^.4999999999+12.01395274*U4*X141^1./X4^0.311418685e-1-5831662.556*U2*X23^.5278118802*X134^1./X2^0.357142857e-1-2266.068147*U2*X128^0.2222222230e-1*X136^1./X2^0.256410254e-1-5810.000000*U2*X154^1./X2^.5000000000  U3' = 5831662.556*U2*U23*X134^1./(X2^0.357142857e-1*X23^.4721881198)+89.10166634*U8*X164^1./X8^0.277777777e-1+152.5467333*U18*X164^1./X18^0.703517588e-1+5371.916275*U19*X164^1./X19^0.44075706e-2-3057.424256*U3*X129^1./X3^.4999999999-24083.44972*U3*X154^1./X3^.5000000002-10.85002492*U3*X15^1.685*X133^1./(X3^0.260521042e-1*X2^0.3358742751e-2*X5^0.2424327076e-1)  U4' = 2266.068147*U2*X128^0.2222222230e-1*X136^1./X2^0.256410254e-1-12.01395274*U4*X141^1./X4^0.311418685e-1-2224.471846*U4*X150^1./X4^0.395170147e-1  U5' = 5810.000000*U2*X154^1./X2^.5000000000+218.2238068*U6*X141^1./X6^.1384615387+2547.853547*U7*X153^1./X7^.4999999998-90147.19843*U5*X23^.5278118802*X134^1./X5^.2000000000-69009.45118*U5*X128^0.2222222230e-1*X136^1./X5^0.400000000e-1  U6' = 69009.45118*U5*X128^0.2222222230e-1*X136^1./X5^0.400000000e-1-218.2238068*U6*X141^1./X6^.1384615387-36106.04490*U6*X150^1./X6^.1706161141  U7' = 90147.19843*U5*U23*X134^1./(X5^.2000000000*X23^.4721881198)+297.0055545*U8*X151^1./X8^0.277777777e-1+24083.44972*U3*X154^1./X3^.5000000002+355.9423777*U18*X151^1./X18^0.703517588e-1+8694.450414*U19*X151^1./X19^0.44075706e-2-2547.853547*U7*X153^1./X7^.4999999998-10.49201131*U7*X15^1.685*X133^1./(X7^0.370898715e-1*X2^0.3320804470e-2*X5^0.2397510852e-1)-37712.92981*U7*X143^1./X7^.5000000003  U8' = 10.49201131*U7*U15*X15^.685*X133^1./(X7^0.370898715e-1*X2^0.3320804470e-2*X5^0.2397510852e-1)+10.85002492*U3*U15*X15^.685*X133^1./(X3^0.260521042e-1*X2^0.3358742751e-2*X5^0.2424327076e-1)+0.3031347641e-2*U20*X37^.5/X20^.5-297.0055545*U8*X151^1./X8^0.277777777e-1-675.1417734*U8*X135^1./X8^.5000000000-89.10166634*U8*X164^1./X8^0.277777777e-1-0.2030671395e-1*U8*X32^.5/X8^.5  U9' = 2512.729767*U11*X140^1./X11^0.59642141e-2-893.1564995*U9*X11^.235*X13^0.2063074440e-3*X15^0.88e-1*X138^1./(X9^0.66689431e-2*X2^0.6568638313e-2*X5^0.1305154574e-1*X14^.5910831328*X16^.2704826039)-118.7734899*U9*X16^0.3376749592e-2*X126^1./X9^0.283617331e-1  U10' = 893.1564995*U9*U13*X11^.235*X15^0.88e-1*X138^1./(X9^0.66689431e-2*X13^.9997936926*X2^0.6568638313e-2*X5^0.1305154574e-1*X14^.5910831328*X16^.2704826039)-94651.30187*U10*X156^1./X10^.4999999999  U11' = 114103.6810*U12*X10^.1493*X149^1.-94.55645805*U11*X9^.326*X15^.248*X139^1./(X11^.5769230769*X2^0.2071563088e-1*X5^0.5022831050e-1)-2512.729767*U11*X140^1./X11^0.59642141e-2  U12' = 23067.02893*U158*X130^1./X158^0.24937653e-2+476.8620195*U24*U25*X152^1./(X24^.8681608437*X25^.9920896508)+2224.471846*U4*X150^1./X4^0.395170147e-1+36106.04490*U6*X150^1./X6^.1706161141+9.967272082*U33*X180^1./X33^.615+21.42776130*U34*X180^1./X34^.615+36.71477823*U35*X180^1./X35^.615+59552.08746*U40*X182^1./X40^.615-174268.8761*U12*X13^.1980849053*X157^1./X12^0.13561833e-2-114103.6810*U12*X10^.1493*X149^1.-294.0873226*U12*X148^1.-16834.72807*U12*X24^.4157339305*X159^1./X12^0.769171e-4-3543.221570*U30*X12^.9998550936*X181^1./X30^.173-4075.356854*U31*X12^.9998550936*X181^1./X31^.173-4169.102075*U32*X12^.9998550936*X183^1./X32^.173  U13' = 351.1209420*X131^1.*X137^.1663551401+801.7109569*X165^1.*X166^0.3984637534e-1-893.1564995*U13*X9^.9933310569*X11^.235*X15^0.88e-1*X138^1./(X13^.9997936926*X2^0.6568638313e-2*X5^0.1305154574e-1*X14^.5910831328*X16^.2704826039)-174268.8761*U13*X12^.9986438167*X157^1./X13^.8019150947-2351.877266*U13*X132^1./X13^.8000000003  U14' = 94.55645805*U11*X9^.326*X15^.248*X139^1./(X11^.5769230769*X2^0.2071563088e-1*X5^0.5022831050e-1)+10.49201131*U7*U15*X15^.685*X133^1./(X7^0.370898715e-1*X2^0.3320804470e-2*X5^0.2397510852e-1)+10.85002492*U3*U15*X15^.685*X133^1./(X3^0.260521042e-1*X2^0.3358742751e-2*X5^0.2424327076e-1)+6.574294837*U18*U15*X15^.685*X155^1./X18^.4999999999-2.010896717*U14*X17^.5000000003*X145^1./X14^.7655700391-912.6577402*U14*X142^1./X14^.5739400206  U15' = 118.7734899*U9*U16*X126^1./(X9^0.283617331e-1*X16^.9966232504)-10.49201131*U15*X15^.685*X7^.9629101285*X133^1./(X2^0.3320804470e-2*X5^0.2397510852e-1)-10.85002492*U15*X15^.685*X3^.9739478958*X133^1./(X2^0.3358742751e-2*X5^0.2424327076e-1)-62.50743872*U15*X128^0.3344404739e-2*X144^1./X15^0.558993415e-1-6.574294837*U15*X15^.685*X18^.5000000001*X155^1.-14.81189310*U15*X168^1./X15^.1821155943  U16' = 57.20507542*X146^1.*X147^.5008488966-118.7734899*U16*X9^.9716382669*X126^1./X16^.9966232504  U17' = 2224.471846*U4*X150^1./X4^0.395170147e-1+36106.04490*U6*X150^1./X6^.1706161141-2.010896717*U17*X14^.2344299609*X145^1./X17^.4999999997  U18' = 675.1417734*U8*X135^1./X8^.5000000000+0.2587449087e-2*U21*X37^.5/X21^.5-152.5467333*U18*X164^1./X18^0.703517588e-1-355.9423777*U18*X151^1./X18^0.703517588e-1-6.574294837*U18*X15^1.685*X155^1./X18^.4999999999-0.1733307912e-1*U18*X32^.5/X18^.5  U19' = 6.574294837*U18*U15*X15^.685*X155^1./X18^.4999999999+0.2763393752e-2*U22*X37^.5/X22^.5-5371.916275*U19*X164^1./X19^0.44075706e-2-8694.450414*U19*X151^1./X19^0.44075706e-2-0.3517226030e-1*U19*X32^.5/X19^.5  U20' = 0.2030671395e-1*U8*X32^.5/X8^.5-0.3031347641e-2*U20*X37^.5/X20^.5  U21' = 0.1733307912e-1*U18*X32^.5/X18^.5-0.2587449087e-2*U21*X37^.5/X21^.5  U22' = 0.3517226030e-1*U19*X32^.5/X19^.5-0.2763393752e-2*U22*X37^.5/X22^.5  U23' = 16834.72807*U12*U24*X159^1./(X12^0.769171e-4*X24^.5842660695)-5831662.556*U23*X2^.9642857143*X134^1./X23^.4721881198-90147.19843*U23*X5^.8000000000*X134^1./X23^.4721881198  U24' = 17.25637757*U25*X128^.3750000000*X160^1./(X25^.9553978709*X12^0.4577407228e-1*X23^.1583280558)-16834.72807*U24*X12^.9999230829*X159^1./X24^.5842660695-476.8620195*U24*X25^0.7910349154e-2*X152^1./X24^.8681608437  U25' = 1.226153687*U124*X123^1./X124^.2588369440+0.9234698783e-2*U38*X128^.5000000001*X161^.7986577182*X163^1./(X38^.9368548879*X12^.111)-17.25637757*U25*X128^.3750000000*X160^1./(X25^.9553978709*X12^0.4577407228e-1*X23^.1583280558)-476.8620195*U25*X24^.1318391563*X152^1./X25^.9920896508-16.37319996*U25*X171^1./(X25^.6960000001*X32^.5000000000)  U26' = 16.37319996*U25*X171^1./(X25^.6960000001*X32^.5000000000)-10524.59060*U26*X172^1./X26^0.22172951e-2  U27' = 10524.59060*U26*X172^1./X26^0.22172951e-2-96427.48842*U27*X173^1./X27^0.24330901e-2  U28' = 96427.48842*U27*X173^1./X27^0.24330901e-2-23509.54134*U28*X174^1./X28^.5-1837.283321*U28*X179^1./X28^.5000000000  U29' = 23509.54134*U28*X174^1./X28^.5-73968.17205*U29*X175^1./X29^.403  U30' = 73968.17205*U29*X175^1./X29^.403+9.967272082*U33*X180^1./X33^.615-87031771.36*U30*X176^1./X30^.338-3543.221570*U30*X12^.9998550936*X181^1./X30^.173  U31' = 87031771.36*U30*X176^1./X30^.338+21.42776130*U34*X180^1./X34^.615-12425.09645*U31*X177^1./X31^.443-4075.356854*U31*X12^.9998550936*X181^1./X31^.173  U32' = 12425.09645*U31*X177^1./X31^.443+0.5563355615e-1*U39+0.2209706584e-1*U37*X20^.5*X21^.5*X22^.5/X37^.5+36.71477823*U35*X180^1./X35^.615-.2956492539*U32-4169.102075*U32*X12^.9998550936*X183^1./X32^.173-1.471655572*U32*X8^.5*X18^.5*X19^.5/X32^.5-861.0295570*U32*X186^1./X32^.5000000000  U33' = 3543.221570*U30*X12^.9998550936*X181^1./X30^.173-9.967272082*U33*X180^1./X33^.615  U34' = 4075.356854*U31*X12^.9998550936*X181^1./X31^.173-21.42776130*U34*X180^1./X34^.615  U35' = 4169.102075*U32*X12^.9998550936*X183^1./X32^.173+.3192737733*U40/X40^.5-36.71477823*U35*X180^1./X35^.615-0.1659444132e-1*U35  U36' = 0.3142262948e-1*U39+0.3491403276e-1*U37-.6137232322*U36-.1939652307*U36*X36^.23*X20^1.23-.1803401715*U36*X36^.23*X21^1.23-.3435069860*U36*X36^.23*X22^1.23  U37' = .1939652307*U36*X36^.23*X20^1.23+.1803401715*U36*X36^.23*X21^1.23+.3435069860*U36*X36^.23*X22^1.23+1.471655572*U32*X8^.5*X18^.5*X19^.5/X32^.5-0.2209706584e-1*U37*X20^.5*X21^.5*X22^.5/X37^.5-0.3491403276e-1*U37  U38' = 0.1397815044e-2*U125*X125^0.4e-1+0.7108537236e-2*U124*X122^1./X124^.3038821952-0.9234698783e-2*U38*X128^.5000000001*X161^.7986577182*X163^1./(X38^.9368548879*X12^.111)  U39' = .2956492539*U32+.6137232322*U36+59552.08746*U40*X182^1./X40^.615-0.8705618563e-1*U39  U40' = 0.1659444132e-1*U35-.3192737733*U40/X40^.5-59552.08746*U40*X182^1./X40^.615  !! L1 L2 L3 L4 L5 L6 L7 L8 L9 L10 L11 L12 L13 L14 L15 L18 L19 L20 L21 L22 L23 L24 L25 L26 L27 L28 L29 L30 L31 L32 L33 L34 L35 L36 L37 L38 L39 L40 L124 L125 IPC_t MIPC_t MIP2C_t L_E_Ester L_Esterol  MI = 0  Hot = 1250  S_L125 = MI  S_U125 = MI  SW = 0  rate1 = .0022  S_L125' = -rate1*S_L125*SW  S_U125' = -rate1*S_U125*SW  L125 = rate1*S_L125 * SW  U125 = X125 - L125  / Pulse:  / @ 501 S_L125 = Hot  / @ 501 S_U125 = X125-Hot  / @ 501 SW = 1  / Pulse Chase 30 min:  @ 501 S_L125 = Hot  @ 501 S_U125 = X125-Hot  @ 501 SW = 1  @ 531 rate1 = 0  X1 = 0.5e-2  X2 = 0.10e-1  X3 = 0.3611111111e-1  X4 = 0.1e-2  X5 = 0.50e-1  X6 = 0.5e-2  X7 = 0.52e-1  X8 = .102  X9 = 5.4  X10 = 8.4  X11 = 3  X12 = 0.1e-1  X13 = 2600  X14 = 10.77669903  X15 = 16.7  X16 = 24.1  X17 = 22  X18 = .14  X19 = 0.85e-2  X20 = .918  X21 = 1.26  X22 = 0.765e-1  X23 = .5  X24 = 182.70  X25 = 870  X26 = .1  X27 = .1  X28 = .1  X29 = .2847305389  X30 = 1.9  X31 = 6.4  X32 = 9.51  X33 = 3.4  X34 = 13.1  X35 = 41.13  X36 = 4.755  X37 = 42.795  X38 = 3086  X39 = 47.55  X40 = 4.57  U1 = 0.5e-2  U2 = 0.10e-1  U3 = 0.3611111111e-1  U4 = 0.1e-2  U5 = 0.50e-1  U6 = 0.5e-2  U7 = 0.52e-1  U8 = .102  U9 = 5.4  U10 = 8.4  U11 = 3  U12 = 0.1e-1  U13 = 2600  U14 = 10.77669903  U15 = 16.7  U16 = 24.1  U17 = 22  U18 = .14  U19 = 0.85e-2  U20 = .918  U21 = 1.26  U22 = 0.765e-1  U23 = .5  U24 = 182.70  U25 = 870  U26 = .1  U27 = .1  U28 = .1  U29 = .2847305389  U30 = 1.9  U31 = 6.4  U32 = 9.51  U33 = 3.4  U34 = 13.1  U35 = 41.13  U36 = 4.755  U37 = 42.795  U38 = 3086  U39 = 47.55  U40 = 4.57  U124 = 227.000  U158 = .5e-1  L1 = 0  L2 = 0  L3 = 0  L4 = 0  L5 = 0  L6 = 0  L7 = 0  L8 = 0  L9 = 0  L10 = 0  L11 = 0  L12 = 0  L13 = 0  L14 = 0  L15 = 0  L16 = 0  L17 = 0  L18 = 0  L19 = 0  L20 = 0  L21 = 0  L22 = 0  L23 = 0  L24 = 0  L25 = 0  L26 = 0  L27 = 0  L28 = 0  L29 = 0  L30 = 0  L31 = 0  L32 = 0  L33 = 0  L34 = 0  L35 = 0  L36 = 0  L37 = 0  L38 = 0  L39 = 0  L40 = 0  L124 = 0  L158 = 0  X122 = 45  X123 = .12  X124 = 227.000  X125 = 1250  X126 = 0.266e-2  X127 = 0.262e-3  X128 = 1100  X129 = 0.54e-5  X130 = 0.508e-1  X131 = 0.13e-2  X132 = 0.45e-2  X133 = 0.33e-3  X134 = 0.165e-4  X135 = 0.1650000000e-3  X136 = 0.4e-5  X137 = 446  X138 = 0.332e-2  X139 = 0.24e-2  X140 = 0.61e-3  X141 = 0.8e-3  X142 = 0.66e-3  X143 = 0.1e-3  X144 = 0.172e-2  X145 = 0.1e-2  X146 = 0.833e-3  X147 = 1176  X148 = 20  X149 = 0.394e-2  X150 = 0.367e-4  X151 = 0.15e-3  X152 = 0.89e-2  X153 = 0.198e-4  X154 = 0.17e-3  X155 = 0.8250000000e-4  X156 = 0.1066e-4  X157 = 0.106e-3  X158 = 0.5e-1  X159 = 0.6000000000e-3  X160 = 0.22e-1  X161 = 60  X163 = .73  X164 = 0.15e-3  X165 = 6/125  X166 = 4000  X168 = 0.5e-4  X171 = .1400000000  X172 = 0.5500000000e-2  X173 = 0.6000000000e-3  X174 = 0.4700000000e-3  X175 = 0.1000000000e-3  X176 = 0.2625e-7  X177 = 0.1000000000e-3  X179 = 0.4e-2  X180 = 0.1100000000e-3  X181 = 0.2540000000e-2  X182 = 0.9975e-6  X183 = 0.2540000000e-2  X186 = 0.1000000000e-2  X_E_Ester = X33+X34+X35+X40  X_Esterol = X30+X31+X32+X36+X37+X39  U_E_Ester = U33+U34+U35+U40  U_Esterol = U30+U31+U32+U36+U37+U39  L_E_Ester = L33+L34+L35+L40  L_Esterol = L30+L31+L32+L36+L37+L39  t0 = 500  hr = .25  tf = 1400 |
| --- |

**Reference.**
