# Supplementary material for: Mathematical Modeling and Validation of the Ergosterol Pathway in Saccharomyces cerevisiae
Source: PLoS One. 2011 Dec 14;6(12):e28344. doi: 10.1371/journal.pone.0028344 (PMC3237449; doi:10.1371/journal.pone.0028344)
Supplement: Material S4 — SL-E flux balanced GMA and Flux aggregated model in Plas [48] format. Files used for total mass experiments and sensitivity analysis. (DOC) [file pone.0028344.s023.doc]

**Material S4. *SL-E flux balanced GMA model in Plas format.*** *File used for sensitivity analysis and total mass experiments, respectively. It can be run into PLAS* [1].

| / GMA_(FLUXES Balanced):  X1' = 174297.0878*X12^.9986438167*X13^.1980849053*X157^1.-650210.4832*X1^.9932909297*X127^1.  X2' = 650210.4832*X1^.9932909297*X127^1.+3057.424256*X3^.5000000001*X129^1.+12.01395274*X4^.9688581315*X141^1.-5832721.509*X2^.9642857143*X23^.5278118802*X134^1.-2266.068147*X2^.9743589746*X128^0.2222222230e-1*X136^1.-5810.000000*X2^.5000000000*X154^1.  X3' = 5832721.509*X2^.9642857143*X23^.5278118802*X134^1.+89.10166634*X8^.9722222223*X164^1.+152.5467333*X18^.9296482412*X164^1.+5602.317528*X19^.9955924294*X164^1.-3057.424256*X3^.5000000001*X129^1.-24097.15836*X3^.4999999998*X154^1.-10.85002492*X3^.9739478958*X15^1.685*X133^1./(X2^0.3358742751e-2*X5^0.2424327076e-1)  X4' = 2266.068147*X2^.9743589746*X128^0.2222222230e-1*X136^1.-12.01395274*X4^.9688581315*X141^1.-2224.471846*X4^.9604829853*X150^1.  X5' = 5810.000000*X2^.5000000000*X154^1.+218.2238068*X6^.8615384613*X141^1.+2547.853547*X7^.5000000002*X153^1.-90147.19843*X5^.8000000000*X23^.5278118802*X134^1.-69009.45118*X5^.9600000000*X128^0.2222222230e-1*X136^1.  X6' = 69009.45118*X5^.9600000000*X128^0.2222222230e-1*X136^1.-218.2238068*X6^.8615384613*X141^1.-36106.04490*X6^.8293838859*X150^1.  X7' = 90147.19843*X5^.8000000000*X23^.5278118802*X134^1.+297.0055545*X8^.9722222223*X151^1.+24097.15836*X3^.4999999998*X154^1.+355.9423777*X18^.9296482412*X151^1.+13072.07423*X19^.9955924294*X151^1.-2547.853547*X7^.5000000002*X153^1.-10.49201131*X7^.9629101285*X15^1.685*X133^1./(X2^0.3320804470e-2*X5^0.2397510852e-1)-37982.31192*X7^.4999999997*X143^1.  X8' = 10.49201131*X7^.9629101285*X15^1.685*X133^1./(X2^0.3320804470e-2*X5^0.2397510852e-1)+10.85002492*X3^.9739478958*X15^1.685*X133^1./(X2^0.3358742751e-2*X5^0.2424327076e-1)+0.3190892254e-2*X20^.5*X37^.5-297.0055545*X8^.9722222223*X151^1.-694.1182673*X8^.5000000000*X135^1.-89.10166634*X8^.9722222223*X164^1.-0.2030671395e-1*X8^.5*X32^.5  X9' = 2513.279808*X11^.9940357859*X140^1.-893.1564995*X9^.9933310569*X11^.235*X13^0.2063074440e-3*X15^0.88e-1*X138^1./(X2^0.6568638313e-2*X5^0.1305154574e-1*X14^.5910831328*X16^.2704826039)-118.8457386*X9^.9716382669*X16^0.3376749592e-2*X126^1.  X10' = 893.1564995*X9^.9933310569*X11^.235*X13^0.2063074440e-3*X15^0.88e-1*X138^1./(X2^0.6568638313e-2*X5^0.1305154574e-1*X14^.5910831328*X16^.2704826039)-94651.30187*X10^.5000000001*X156^1.  X11' = 114122.1528*X10^.1493*X12^1.*X149^1.-94.55645805*X9^.326*X11^.4230769231*X15^.248*X139^1./(X2^0.2071563088e-1*X5^0.5022831050e-1)-2513.279808*X11^.9940357859*X140^1.  X12' = 23067.02893*X130^1.*X158^.9975062347+476.8620195*X24^.1318391563*X25^0.7910349154e-2*X152^1.+2224.471846*X4^.9604829853*X150^1.+36106.04490*X6^.8293838859*X150^1.+869.0384668*X33^.385*X180^1.+1623.520875*X34^.385*X180^1.+1483.465863*X35^.385*X180^1.-174297.0878*X12^.9986438167*X13^.1980849053*X157^1.-114122.1528*X10^.1493*X12^1.*X149^1.-300.0052503*X12^1.*X148^1.-16837.45902*X12^.9999230829*X24^.4157339305*X159^1.-3543.221570*X12^.9998550936*X30^.827*X181^1.-4075.356854*X12^.9998550936*X31^.827*X181^1.-4169.102075*X12^.9998550936*X32^.827*X183^1.  X13' = 351.1209420*X131^1.*X137^.1663551401+801.7109569*X165^1.*X166^0.3984637534e-1-893.1564995*X9^.9933310569*X11^.235*X13^0.2063074440e-3*X15^0.88e-1*X138^1./(X2^0.6568638313e-2*X5^0.1305154574e-1*X14^.5910831328*X16^.2704826039)-174297.0878*X12^.9986438167*X13^.1980849053*X157^1.-2351.870680*X13^.1999999997*X132^1.  X14' = 94.55645805*X9^.326*X11^.4230769231*X15^.248*X139^1./(X2^0.2071563088e-1*X5^0.5022831050e-1)+10.49201131*X7^.9629101285*X15^1.685*X133^1./(X2^0.3320804470e-2*X5^0.2397510852e-1)+10.85002492*X3^.9739478958*X15^1.685*X133^1./(X2^0.3358742751e-2*X5^0.2424327076e-1)+6.856266054*X15^1.685*X18^.5000000001*X155^1.-2.010896717*X14^.2344299609*X17^.5000000003*X145^1.-913.2079842*X14^.4260599794*X142^1.  X15' = 118.8457386*X9^.9716382669*X16^0.3376749592e-2*X126^1.-10.49201131*X7^.9629101285*X15^1.685*X133^1./(X2^0.3320804470e-2*X5^0.2397510852e-1)-10.85002492*X3^.9739478958*X15^1.685*X133^1./(X2^0.3358742751e-2*X5^0.2424327076e-1)-62.50743872*X15^.9441006585*X128^0.3344404739e-2*X144^1.-6.856266054*X15^1.685*X18^.5000000001*X155^1.-14.81189310*X15^.8178844057*X168^1.  X16' = 57.23987266*X146^1.*X147^.5008488966-118.8457386*X9^.9716382669*X16^0.3376749592e-2*X126^1.  X17' = 2224.471846*X4^.9604829853*X150^1.+36106.04490*X6^.8293838859*X150^1.-2.010896717*X14^.2344299609*X17^.5000000003*X145^1.  X18' = 694.1182673*X8^.5000000000*X135^1.+0.2723630619e-2*X21^.5*X37^.5-152.5467333*X18^.9296482412*X164^1.-355.9423777*X18^.9296482412*X151^1.-6.856266054*X15^1.685*X18^.5000000001*X155^1.-0.1733307912e-1*X18^.5*X32^.5  X19' = 6.856266054*X15^1.685*X18^.5000000001*X155^1.+0.5526787506e-2*X22^.5*X37^.5-5602.317528*X19^.9955924294*X164^1.-13072.07423*X19^.9955924294*X151^1.-0.3517226030e-1*X19^.5*X32^.5  X20' = 0.2030671395e-1*X8^.5*X32^.5-0.3190892254e-2*X20^.5*X37^.5  X21' = 0.1733307912e-1*X18^.5*X32^.5-0.2723630619e-2*X21^.5*X37^.5  X22' = 0.3517226030e-1*X19^.5*X32^.5-0.5526787506e-2*X22^.5*X37^.5  X23' = 16837.45902*X12^.9999230829*X24^.4157339305*X159^1.-5832721.509*X2^.9642857143*X23^.5278118802*X134^1.-90147.19843*X5^.8000000000*X23^.5278118802*X134^1.  X24' = 17.25662971*X25^0.4460212912e-1*X128^.3750000000*X160^1./(X12^0.4577407228e-1*X23^.1583280558)-16837.45902*X12^.9999230829*X24^.4157339305*X159^1.-476.8620195*X24^.1318391563*X25^0.7910349154e-2*X152^1.  X25' = 1.232059507*X123^1.*X124^.7411630560+0.9272832387e-2*X38^0.6314511210e-1*X128^.5000000001*X161^.7986577182*X163^1./X12^.111-17.25662971*X25^0.4460212912e-1*X128^.3750000000*X160^1./(X12^0.4577407228e-1*X23^.1583280558)-476.8620195*X24^.1318391563*X25^0.7910349154e-2*X152^1.-16.67327233*X25^.3039999999*X171^1./X32^.5000000000  X26' = 16.67327233*X25^.3039999999*X171^1./X32^.5000000000-10717.47524*X26^.9977827049*X172^1.  X27' = 10717.47524*X26^.9977827049*X172^1.-98194.71929*X27^.9975669099*X173^1.  X28' = 98194.71929*X27^.9975669099*X173^1.-24226.97172*X28^.5*X174^1.-1837.283321*X28^.5000000000*X179^1.  X29' = 24226.97172*X28^.5*X174^1.-76225.42641*X29^.597*X175^1.  X30' = 76225.42641*X29^.597*X175^1.+869.0384668*X33^.385*X180^1.-89687681.84*X30^.662*X176^1.-3543.221570*X12^.9998550936*X30^.827*X181^1.  X31' = 89687681.84*X30^.662*X176^1.+1623.520875*X34^.385*X180^1.-12804.26769*X31^.557*X177^1.-4075.356854*X12^.9998550936*X31^.827*X181^1.  X32' = 12804.26769*X31^.557*X177^1.+0.6137232320e-1*X39+0.2569426260e-1*X20^.5*X21^.5*X22^.5*X37^.5+1483.465863*X35^.385*X180^1.-.3068616160*X32-4169.102075*X12^.9998550936*X32^.827*X183^1.-1.471655572*X8^.5*X18^.5*X19^.5*X32^.5-1167.633998*X32^.5000000000*X186^1.  X33' = 3543.221570*X12^.9998550936*X30^.827*X181^1.-869.0384668*X33^.385*X180^1.  X34' = 4075.356854*X12^.9998550936*X31^.827*X181^1.-1623.520875*X34^.385*X180^1.  X35' = 4169.102075*X12^.9998550936*X32^.827*X183^1.+.3192737733*X40^.5-1483.465863*X35^.385*X180^1.-0.1659444132e-1*X35  X36' = 0.6137232320e-1*X39+0.6819147022e-1*X37-.6137232320*X36-.1939652307*X20^1.23*X36^1.23-.1803401715*X21^1.23*X36^1.23-.3435069860*X22^1.23*X36^1.23  X37' = .1939652307*X20^1.23*X36^1.23+.1803401715*X21^1.23*X36^1.23+.3435069860*X22^1.23*X36^1.23+1.471655572*X8^.5*X18^.5*X19^.5*X32^.5-0.2569426260e-1*X20^.5*X21^.5*X22^.5*X37^.5-0.6819147022e-1*X37  X38' = 0.1397815044e-2*X125^1.04+0.7142775800e-2*X122^1.*X124^.6961178048-0.9272832387e-2*X38^0.6314511210e-1*X128^.5000000001*X161^.7986577182*X163^1./X12^.111  X39' = .3068616160*X32+.6137232320*X36-.1227446464*X39  X40' = 0.1659444132e-1*X35-.3192737733*X40^.5  / S-system (FLUXES Balanced):  / X1' = 174297.0878*X12^.9986438167*X13^.1980849053*X157^1.-650210.4832*X1^.9932909297*X127^1.  / X2' = 653180.2624*X1^.9897592360*X127^.9964444519*X3^0.1771046463e-2*X129^0.3542092927e-2*X4^0.1303622234e-4*X141^0.1345524377e-4-3829311.911*X2^.9125144151*X23^.4688843834*X134^.8883551147*X128^0.2990054186e-5*X136^0.1345524379e-3*X154^.1115103329  / X3' = 5617459.784*X2^.9492947279*X23^.5196064069*X134^.9844537918*X8^0.1766771855e-2*X164^0.1554620810e-1*X18^0.4278692153e-2*X19^0.9086246408e-2-22994.47239*X3^.5104888376*X129^0.3925262269e-2*X154^.9739439554*X15^0.3729036829e-1*X133^0.2213078237e-1/(X2^0.7433160486e-4*X5^0.5365225491e-3)  / X4' = 2266.068147*X2^.9743589746*X128^0.2222222230e-1*X136^1.-1826.646699*X4^.9613204999*X141^0.9999999983e-1*X150^.9000000002  / X5' = 7642.582854*X2^.4405768391*X154^.8811536782*X6^0.1397228859e-1*X141^0.1621783498e-1*X7^0.5131424363e-1*X153^.1026284872-134474.4534*X5^.8259485360*X23^.4422122202*X134^.8378216499*X128^0.3603963349e-2*X136^.1621783501  / X6' = 69009.45118*X5^.9600000000*X128^0.2222222230e-1*X136^1.-29984.29767*X6^.8325993433*X141^0.9999999978e-1*X150^.9000000002  / X7' = 42714.18630*X5^0.8321761351e-1*X23^0.5490405631e-1*X134^.1040220169*X8^0.5213876334e-2*X151^0.3372347203e-1*X3^.4311272553*X154^.8622545111*X18^0.8838719152e-2*X19^0.1876993653e-1-35493.70723*X7^.5129124149*X153^0.1274211788e-1*X15^0.4700139028e-1*X133^0.2789400016e-1*X143^.9593638820/(X2^0.9263052042e-4*X5^0.6687616809e-3)  / X8' = 2.390774391*X7^.3856907801*X15^1.148989990*X133^.6818931692*X3^.2740164924*X20^.1590534154*X37^.1590534154/(X2^0.2275107674e-2*X5^0.1642390914e-1)-58.72968738*X8^.5472745777*X151^0.7700836195e-1*X135^.5817822986*X164^0.2310250858e-1*X32^.1590534154  / X9' = 2513.279808*X11^.9940357859*X140^1.-830.5409732*X9^.9855215626*X11^.1503990259*X13^0.1320359090e-3*X15^0.5631963525e-1*X138^.6399958550*X126^.3600041450/(X2^0.4203901294e-2*X5^0.8352935176e-2*X14^.3782907550*X16^.1718921014)  / X10' = 893.1564995*X9^.9933310569*X11^.235*X13^0.2063074440e-3*X15^0.88e-1*X138^1./(X2^0.6568638313e-2*X5^0.1305154574e-1*X14^.5910831328*X16^.2704826039)-94651.30187*X10^.5000000001*X156^1.  / X11' = 114122.1528*X10^.1493*X12^1.*X149^1.-1898.129087*X9^0.8489689487e-1*X11^.8453467230*X15^0.6458414088e-1*X139^.2604199229*X140^.7395800771/(X2^0.5394762997e-2*X5^0.1308045275e-1)  / X12' = 21821.50936*X130^.8523009695*X158^.8501755308*X24^0.1693513343e-1*X25^0.1016107977e-2*X152^.1284529870*X4^0.1487499539e-5*X150^0.2377769997e-3*X6^0.1959239455e-3*X33^0.8512065171e-3*X180^0.1900826665e-1*X34^0.2672907212e-2*X35^0.3794068930e-2-1053.812859*X12^.9999789851*X13^0.2524280191e-2*X157^0.1274342528e-1*X10^0.1331815376e-1*X149^0.8920397697e-1*X148^.8663272854*X24^0.5286907422e-2*X159^0.1271704577e-1*X30^0.1828435816e-2*X181^0.9153542153e-2*X31^0.5741543544e-2*X32^0.8149857154e-2*X183^0.9854724490e-2  / X13' = 877.5905845*X131^0.2297461868e-1*X137^0.3821945910e-2*X165^.9770253814*X166^0.3893092007e-1-3198.603074*X9^0.5299503810e-1*X11^0.1253744546e-1*X13^.1893100031*X15^0.4694873195e-2*X138^0.5335083175e-1*X12^0.1608019856e-1*X157^0.1610203587e-1*X132^.9305471324/(X2^0.3504423176e-3*X5^0.6963108210e-3*X14^0.3153477678e-1*X16^0.1443047189e-1)  / X14' = 106.3819822*X9^.3129321827*X11^.4061177453*X15^.3056026183*X139^.9599146708*X7^0.1446750384e-1*X133^0.2557824183e-1*X3^0.1027853104e-1*X18^0.7253543750e-2*X155^0.1450708750e-1/(X2^0.1997057872e-1*X5^0.4883096331e-1)-908.7281507*X14^.4241771646*X17^0.4912629974e-2*X145^0.9825259942e-2*X142^.9901747398  / X15' = 118.8457386*X9^.9716382669*X16^0.3376749592e-2*X126^1.-73.23179473*X7^0.1474152721e-1*X15^.9737940669*X133^0.2606270937e-1*X3^0.1047321270e-1*X128^0.3192745305e-2*X144^.9546527871*X18^0.7390930304e-2*X155^0.1478186061e-1*X168^0.4502643142e-2/(X2^0.8695712578e-4*X5^0.6277399306e-3)  / X16' = 57.23987266*X146^1.*X147^.5008488966-118.8457386*X9^.9716382669*X16^0.3376749592e-2*X126^1.  / X17' = 36876.97572*X4^0.6255859654e-2*X150^.9999999999*X6^.8239819064-2.010896717*X14^.2344299609*X17^.5000000003*X145^1.  / X18' = 16.30970666*X8^.3232522094*X135^.6465044189*X21^.1767477906*X37^.1767477906-6.088553476*X18^.5931192306*X164^0.6502009427e-1*X151^.1517135533*X15^.7241637496*X155^.4297707713*X32^.1767477906  / X19' = 1.572623650*X15^1.193967992*X18^.3542931728*X155^.7085863456*X22^.1457068272*X37^.1457068272-732.7378009*X19^.8511700286*X164^.2125759037*X151^.4960104420*X32^.1457068272  / X20' = 0.2030671395e-1*X8^.5*X32^.5-0.3190892254e-2*X20^.5*X37^.5  / X21' = 0.1733307912e-1*X18^.5*X32^.5-0.2723630619e-2*X21^.5*X37^.5  / X22' = 0.3517226030e-1*X19^.5*X32^.5-0.5526787506e-2*X22^.5*X37^.5  / X23' = 16837.45902*X12^.9999230829*X24^.4157339305*X159^1.-5250263.451*X2^.8614680782*X23^.5278118802*X134^1.000000000*X5^0.8530055734e-1  / X24' = 17.25662971*X25^0.4460212912e-1*X128^.3750000000*X160^1./(X12^0.4577407228e-1*X23^.1583280558)-889.8260236*X12^0.9007625317e-1*X24^.1574133010*X159^0.9008318211e-1*X25^0.7197759730e-2*X152^.9099168178  / X25' = 0.9028113010e-1*X123^.3350435840*X124^.2483219266*X38^0.4198874743e-1*X128^.3324782080*X161^.5310725739*X163^.6649564158/X12^0.7381016215e-1-166.9543092*X25^0.9381000233e-1*X128^.1490526467*X160^.3974737245*X24^0.4768200749e-1*X152^.3616680266*X171^.2408582488/(X12^0.1819399100e-1*X23^0.6293124202e-1*X32^.1204291244)  / X26' = 16.67327233*X25^.3039999999*X171^1./X32^.5000000000-10717.47524*X26^.9977827049*X172^1.  / X27' = 10717.47524*X26^.9977827049*X172^1.-98194.71929*X27^.9975669099*X173^1.  / X28' = 98194.71929*X27^.9975669099*X173^1.-17210.61472*X28^.5000000001*X174^.6077493696*X179^.3922506304  / X29' = 24226.97172*X28^.5*X174^1.-76225.42641*X29^.597*X175^1.  / X30' = 75312.27415*X29^.5726476131*X175^.9592087324*X33^0.1570463804e-1*X180^0.4079126765e-1-70330064.92*X30^.6687305590*X176^.9592087324*X12^0.4078535674e-1*X181^0.4079126765e-1  / X31' = 35604894.56*X30^.5840124738*X176^.8821940693*X34^0.4535528330e-1*X180^.1178059307-16077.84867*X31^.5888076013*X177^.8821940693*X12^.1177888599*X181^.1178059307  / X32' = 182.3209797*X31^.2765797253*X177^.4965524691*X39^.4024308260*X20^0.3447530873e-2*X21^0.3447530873e-2*X22^0.3447530873e-2*X37^0.3447530873e-2*X35^0.3623683263e-1*X180^0.9412164317e-1-120.2389353*X32^.7319931904*X12^0.9410800434e-1*X183^0.9412164317e-1*X8^0.3447530873e-2*X18^0.3447530873e-2*X19^0.3447530873e-2*X186^.4965524691  / X33' = 3543.221570*X12^.9998550936*X30^.827*X181^1.-869.0384668*X33^.385*X180^1.  / X34' = 4075.356854*X12^.9998550936*X31^.827*X181^1.-1623.520875*X34^.385*X180^1.  / X35' = 72.96807376*X12^.4999275466*X32^.4134999999*X183^.4999999999*X40^.2499999999-9.923162237*X35^.6924999999*X180^.4999999999  / X36' = .1293842177*X39^.5000000000*X37^.5000000000-1.021486386*X36^1.115000000*X20^.2504191618*X21^.3437125747*X22^0.2086826348e-1  / X37' = .4727668252*X20^.4924017422*X36^1.209280749*X21^.6758455279*X22^0.4103347851e-1*X8^0.8422459894e-2*X18^0.8422459894e-2*X19^0.8422459894e-2*X32^0.8422459894e-2-0.7306649498e-1*X20^0.8422459894e-2*X21^0.8422459894e-2*X22^0.8422459894e-2*X37^.9915775401  / X38' = 0.8525512814e-2*X125^.1477628753*X122^.8579203122*X124^.5972136044-0.9272832387e-2*X38^0.6314511210e-1*X128^.5000000001*X161^.7986577182*X163^1./X12^.111  / X39' = .8679357180*X32^.5000000000*X36^.5000000000-.1227446464*X39^1.000000000  / X40' = 0.1659444132e-1*X35-.3192737735*X40^.4999999999  && X122 X123 X124 X125 X126 X127 X128 X129 X130 X131 X132 X133 X134 X135 X136 X137 X138 X139 X140 X141 X142 X143 X144 X145 X146 X147 X148 X149 X150 X151 X152 X153 X154 X155 X156 X157 X158 X159 X160 X161 X163 X164 X165 X166 X168 X171 X172 X173 X174 X175 X176 X177 X179 X180 X181 X183 X186  !! X1 X2 X3 X4 X5 X6 X7 X8 X9 X10 X11 X12 X13 X14 X15 X16 X17 X18 X19 X20 X21 X22 X23 X24 X25 X26 X27 X28 X29 X30 X31 X32 X33 X34 X35 X36 X37 X38 X39 X40  X1 = 0.5e-2  X2 = 0.10e-1  X3 = 0.3611111111e-1  X4 = 0.1e-2  X5 = 0.50e-1  X6 = 0.5e-2  X7 = 0.52e-1  X8 = .102  X9 = 5.4  X10 = 8.4  X11 = 3  X12 = 0.1e-1  X13 = 2600  X14 = 10.77669903  X15 = 16.7  X16 = 24.1  X17 = 22  X18 = .14  X19 = 0.85e-2  X20 = .918  X21 = 1.26  X22 = 0.765e-1  X23 = .5  X24 = 182.70  X25 = 870  X26 = .1  X27 = .1  X28 = .1  X29 = .2847305389  X30 = 1.9  X31 = 6.4  X32 = 9.51  X33 = 3.4  X34 = 13.1  X35 = 41.13  X36 = 4.755  X37 = 42.795  X38 = 3086  X39 = 47.55  X40 = 4.57  X122 = 45  X123 = .12  X124 = 227.000  X125 = 1250  X126 = 0.266e-2  X127 = 0.262e-3  X128 = 1100  X129 = 0.54e-5  X130 = 0.508e-1  X131 = 0.13e-2  X132 = 0.45e-2  X133 = 0.33e-3  X134 = 0.165e-4  X135 = 0.1650000000e-3  X136 = 0.4e-5  X137 = 446  X138 = 0.332e-2  X139 = 0.24e-2  X140 = 0.61e-3  X141 = 0.8e-3  X142 = 0.66e-3  X143 = 0.1e-3  X144 = 0.172e-2  X145 = 0.1e-2  X146 = 0.833e-3  X147 = 1176  X148 = 20  X149 = 0.394e-2  X150 = 0.367e-4  X151 = 0.15e-3  X152 = 0.89e-2  X153 = 0.198e-4  X154 = 0.17e-3  X155 = 0.8250000000e-4  X156 = 0.1066e-4  X157 = 0.106e-3  X158 = 0.5e-1  X159 = 0.6000000000e-3  X160 = 0.22e-1  X161 = 60  X163 = .73  X164 = 0.15e-3  X165 = 6/125  X166 = 4000  X168 = 0.5e-4  X171 = .1400000000  X172 = 0.5500000000e-2  X173 = 0.6000000000e-3  X174 = 0.4700000000e-3  X175 = 0.1000000000e-3  X176 = 0.2625e-7  X177 = 0.1000000000e-3  X179 = 0.4e-2  X180 = 0.1100000000e-3  X181 = 0.2540000000e-2  X182 = 0.9975e-6  X183 = 0.2540000000e-2  X186 = 0.1000000000e-2  X_E_Ester = X33+X34+X35+X40  X_Esterol = X30+X31+X32+X36+X37+X39  t0 = 0  hr = .25  tf = 60 |
| --- |

**Reference.**
