# Supplementary material for: Mathematical Modeling and Validation of the Ergosterol Pathway in Saccharomyces cerevisiae
Source: PLoS One. 2011 Dec 14;6(12):e28344. doi: 10.1371/journal.pone.0028344 (PMC3237449; doi:10.1371/journal.pone.0028344)
Supplement: Material S5 — SL-E GMA model in Matlab® [49] format. Files used for the dynamic simulations. (DOC) [file pone.0028344.s024.doc]

**Material S5. - SL-E GMA model in Matlab format.** Files used for the dynamic simulations. This code was written for MATLAB® [1].

| function Ergo_Unbalanced_main % Unbalanced flux model  global MM SMM P PP    % Independent variables (MM) perturbed in the work: X(133), X(134), X(157), X(171), and X(172)  % Independent variables (MM) percent’s tested in the work: 0.01, 1, 10, and 12.5    % Enter the following data:    s = [121]; % ODE to be plotted. Single value must be between 1 and 121.  TT = ('no'); % To plot total labeled Sterols and Steryl Esters.  % no = no plot, yes = plot. No spaces inside the characters ''.    MM = []; % Variable(s) to be perturbed  P = []; % Percent of change for the MM variable(s) to be perturbed.  % Empty bracket in MM or P means no change,  % otherwise number of elements in MM = P    time_start = 500; % Starting time for numeric integration  time_end = 1400; % Final time for numeric integration    pert_start = 501; % pert_start > time_start.  pert_end = 531; % pert_end < time_end.  % time_start < pert_start < pert_end < time_end    acetate = 1250; % Perturbation initial value    SW1 = 0; % First perturbation change.  SW2 = 1; % Second perturbation change.  SW3 = 0; % Third perturbation change.  % 0 = no perturbation, 1 = yes perturbation:  % Acetate Pulse experiment: SW1=0, SW2=1, SW3=1.  % Acetate Chase experiment: SW1=0, SW2=1, SW3=0.    %---------------------------------------------------------    if pert_start <= time_start || pert_end >= time_end...  || pert_start == pert_end || time_start == time_end...  || pert_start > pert_end...  error(['Sequence must be: time_start < pert_start < pert_end < time_end']);  end    tspan = [time_start, time_end];  tspan1 = [tspan(1), pert_start];  tspan2 = [pert_start, pert_end];  tspan3 = [pert_end, tspan(2)];    inix =[0.5e-2, 0.10e-1, 0.3611111111e-1, 0.1e-2, 0.50e-1, 0.5e-2, 0.52e-1, .102,...  5.4, 8.4, 3, 0.1e-1, 2600, 10.77669903, 16.7, 24.1,...  22, .14, 0.85e-2, .918, 1.26, 0.765e-1, .5, 182.70,...  870, .1, .1, .1, .2847305389, 1.9, 6.4, 9.51,...  3.4, 13.1, 41.13, 4.755, 42.795, 3086, 47.55, 4.57];    inil = [0 0 0 0 0 0 0 0 ,...  0 0 0 0 0 0 0 0 ,...  0 0 0 0 0 0 0 0 ,...  0 0 0 0 0 0 0 0 ,...  0 0 0 0 0 0 0 0 ];    inip = [acetate*SW1];    indp = [122:166,168,169,171:177,179:186];    ini = [inix, inil, inix, inip];  inif = ini;  depl = [numel(inix)+1 : numel(inix)+numel(inil)];  depx = [1:numel(ini)];  depx1 = [ 1:numel(inix)];  depx2 = [ numel(inix)+numel(inil)+1 : 2*numel(inix)+numel(inil) ];  depx3 = [ 2*numel(inix)+numel(inil)+1 : numel(ini) ];    if isempty(s)  error('Introduce a time dependent variable');  elseif ~ismember(s,depx)  error('Not a valid time dependent variable');  elseif ismember(s,depx)  % (1) ----------------------------------------------------  [t,x] = ode23tb('Ergo_Unbalanced', tspan1, ini);  acu1 = [t,x];  sa = length(acu1(:,1));  st = length(acu1(sa,:));  % (2) ----------------------------------------------------  if acu1(sa,st)~=0  acu1(sa,st) = acu1(sa,st)*SW2;  else  acu1(sa,st) = acetate*SW2;  end  ini2 = [acu1(sa,2:st)];  [t,x] = ode23tb('Ergo_Unbalanced', tspan2, ini2);  acu2 = [t,x];  acu2 = vertcat(acu1,acu2);  sa = length(acu2(:,1));  st = length(acu2(sa,:));  % (3) ----------------------------------------------------  if acu2(sa,st)~=0  acu2(sa,st) = acu2(sa,st)*SW3;  else  acu2(sa,st) = acetate*SW3;  end  acu2(sa,st)=acu2(sa,st)*SW3;  ini3 = [acu2(sa,2:st)];  [t,x] = ode23tb('Ergo_Unbalanced', tspan3, ini3);  acu3 = [t,x];  acu3 = vertcat(acu2,acu3);  sa = length(acu3(:,1));  st = length(acu3(sa,:));  % -------------------------------------------------------  if ismember(s,depx1) && strcmp('no',TT)  x= acu3(:,s+1)/inif(s);  g = plot(acu3(:,1),x,'-');  title ('Yeast Sphingolipid-Ergosterol model-Total mass');  xlabel('time (min)'); ylabel(['Normalized X(',num2str(s),')']);  set(g, 'Color', 'black', 'LineWidth',3);  elseif ismember(s,depl) && strcmp('no',TT)  x= acu3(:,s+1);  g = plot(acu3(:,1),acu3(:,s+1),'r-');  title ('Yeast Sphingolipid-Ergosterol model-Labeled material');  xlabel('time (min)'); ylabel(['X(',num2str(s),')']);  set(g,'LineWidth',3);  elseif strcmp(TT,'yes')  Sterols = acu3(:,70+1)+acu3(:,71+1)+acu3(:,72+1)+acu3(:,76+1)...  +acu3(:,77+1)+acu3(:,79+1);  Steryl_esters = acu3(:,73+1)+acu3(:,74+1)+acu3(:,75+1)+acu3(:,80+1);  g = plot(acu3(:,1),Sterols,'b-',acu3(:,1),Steryl_esters,'r-.');  title ('Yeast Sphingolipid-Ergosterol model-acetate material');  legend('Sterols','Steryl esters');  xlabel('time (min)'); ylabel(['% Total Sterols']);  set(g,'LineWidth',3);  elseif ismember(s,depx2) && strcmp('no',TT)  x= acu3(:,s+1)/inif(s);  g = plot(acu3(:,1),x,'-');  title ('Yeast Sphingolipid-Ergosterol model-Unlabeled material');  xlabel('time (min)'); ylabel(['Normalized X(',num2str(s),')']);  set(g, 'Color', 'blue', 'LineWidth',3);  elseif ismember(s,depx3) && strcmp('no',TT)  x= acu3(:,s+1);  g = plot(acu3(:,1),x,'-');  title ('Yeast Sphingolipid-Ergosterol model - Acetate perturbation');  xlabel('time (min)'); ylabel(['X(',num2str(s),')']);  set(g, 'Color', 'red', 'LineWidth',3);  elseif ismember(s,indp)  error(['Not a time dependent variable']);  end  if ~isempty(MM)  ['Time interval = [', num2str(time_start), ',',num2str(time_end),']']  ['Variable(s) perturbed : ',num2str(MM), ' . Percent(s) of change: ',num2str(P)]  if ismember(MM,depx1)  XX= real(acu3(sa,MM+1));  XXX= real(acu3(sa,MM+1)/inif(MM));  ['Initial X(',num2str(MM), ') = ',num2str(ini(MM)) ,...  ', Final X(',num2str(MM), ') = ',num2str(real(XX)),...  ', Normalized X(',num2str(MM), ') = ',num2str(XXX)]  else  XX= real(acu3(sa,s+1));  XXX= real(acu3(sa,s+1)/inif(s));  ['Initial X(',num2str(s), ') = ',num2str(ini(s)) ,...  ', Final X(',num2str(s), ') = ',num2str(real(XX)),...  ', Normalized X(',num2str(s), ') = ',num2str(XXX)]  end  end  end  end  %----------------------------------------------------------------------  function [ERG] = Ergo_Unbalanced(t,X)    global MM SMM P PP  SMM = size(MM);  PP = length(P);    X(122) = 45;  X(123) = 0.12e0;  X(124) = 0.227000e3;  X(125) = 1250;  X(126) = 0.266e-2;  X(127) = 0.262e-3;  X(128) = 1100;  X(129) = 0.54e-5;  X(130) = 0.508e-1;  X(131) = 0.13e-2;  X(132) = 0.45e-2;  X(133) = 0.33e-3;  X(134) = 0.165e-4;  X(135) = 0.1650000000e-3;  X(136) = 0.4e-5;  X(137) = 446;  X(138) = 0.332e-2;  X(139) = 0.24e-2;  X(140) = 0.61e-3;  X(141) = 0.8e-3;  X(142) = 0.66e-3;  X(143) = 0.1e-3;  X(144) = 0.172e-2;  X(145) = 0.1e-2;  X(146) = 0.833e-3;  X(147) = 1176;  X(148) = 20;  X(149) = 0.394e-2;  X(150) = 0.367e-4;  X(151) = 0.15e-3;  X(152) = 0.89e-2;  X(153) = 0.198e-4;  X(154) = 0.17e-3;  X(155) = 0.8250000000e-4;  X(156) = 0.1066e-4;  X(157) = 0.106e-3;  X(158) = 0.5e-1;  X(159) = 0.6000000000e-3;  X(160) = 0.22e-1;  X(161) = 60;  X(162) = 0;  X(163) = 0.73e0;  X(164) = 0.15e-3;  X(165) = 0.6e1 / 0.125e3;  X(166) = 4000;  X(168) = 0.5e-4;  X(169) = 0;  X(171) = 0.1400000000e0;  X(172) = 0.5500000000e-2;  X(173) = 0.6000000000e-3;  X(174) = 0.4700000000e-3;  X(175) = 0.1000000000e-3;  X(176) = 0.2625e-7;  X(177) = 0.1000000000e-3;  X(179) = 0.4e-2;  X(180) = 0.1100000000e-3;  X(181) = 0.2540000000e-2;  X(182) = 0.9975e-6;  X(183) = 0.2540000000e-2;  X(184) = 0.5e-1;  X(185) = 0.227000e3;  X(186) = 0.1000000000e-2;    rate1 = 0.0022;  X(187) = rate1 * X(121);  X(188) = X(125) - X(187);    for m= 1:PP  if isempty(MM) || isempty(P)  X(MM) = X(MM);  elseif PP == SMM(2)  X(MM(m)) = X(MM(m))*P(m)/100;  elseif PP ~= SMM(2)  error('Not the same number of variables and perturbations (MM ~= P)');  ['Time interval = [', num2str(Time_start), ',',num2str(Time_end),']']  end  end    % Total mass ODE's:    ERG(1,1) = 0.1742688761e6 * X(12) ^ 0.9986438167e0 * X(13) ^ 0.1980849053e0 * X(157) ^ 0.1e1 - 0.6501052401e6 * X(1) ^ 0.9932909297e0 * X(127) ^ 0.1e1;  ERG(2,1) = 0.6501052401e6 * X(1) ^ 0.9932909297e0 * X(127) ^ 0.1e1 + 0.3057424256e4 * X(3) ^ 0.5000000001e0 * X(129) ^ 0.1e1 + 0.1201395274e2 * X(4) ^ 0.9688581315e0 * X(141) ^ 0.1e1 - 0.5831662556e7 * X(2) ^ 0.9642857143e0 * X(23) ^ 0.5278118802e0 * X(134) ^ 0.1e1 - 0.2266068147e4 * X(2) ^ 0.9743589746e0 * X(128) ^ 0.2222222230e-1 * X(136) ^ 0.1e1 - 0.5810000000e4 * X(2) ^ 0.5000000000e0 * X(154) ^ 0.1e1;  ERG(3,1) = 0.5831662556e7 * X(2) ^ 0.9642857143e0 * X(23) ^ 0.5278118802e0 * X(134) ^ 0.1e1 + 0.8910166634e2 * X(8) ^ 0.9722222223e0 * X(164) ^ 0.1e1 + 0.1525467333e3 * X(18) ^ 0.9296482412e0 * X(164) ^ 0.1e1 + 0.5371916275e4 * X(19) ^ 0.9955924294e0 * X(164) ^ 0.1e1 - 0.3057424256e4 * X(3) ^ 0.5000000001e0 * X(129) ^ 0.1e1 - 0.2408344972e5 * X(3) ^ 0.4999999998e0 * X(154) ^ 0.1e1 - 0.1085002492e2 * X(2) ^ (-0.3358742751e-2) * X(3) ^ 0.9739478958e0 * X(5) ^ (-0.2424327076e-1) * X(15) ^ 0.1685e1 * X(133) ^ 0.1e1;  ERG(4,1) = 0.2266068147e4 * X(2) ^ 0.9743589746e0 * X(128) ^ 0.2222222230e-1 * X(136) ^ 0.1e1 - 0.1201395274e2 * X(4) ^ 0.9688581315e0 * X(141) ^ 0.1e1 - 0.2224471846e4 * X(4) ^ 0.9604829853e0 * X(150) ^ 0.1e1;  ERG(5,1) = 0.5810000000e4 * X(2) ^ 0.5000000000e0 * X(154) ^ 0.1e1 + 0.2182238068e3 * X(6) ^ 0.8615384613e0 * X(141) ^ 0.1e1 + 0.2547853547e4 * X(7) ^ 0.5000000002e0 * X(153) ^ 0.1e1 - 0.9014719843e5 * X(5) ^ 0.8000000000e0 * X(23) ^ 0.5278118802e0 * X(134) ^ 0.1e1 - 0.6900945118e5 * X(5) ^ 0.9600000000e0 * X(128) ^ 0.2222222230e-1 * X(136) ^ 0.1e1;  ERG(6,1) = 0.6900945118e5 * X(5) ^ 0.9600000000e0 * X(128) ^ 0.2222222230e-1 * X(136) ^ 0.1e1 - 0.2182238068e3 * X(6) ^ 0.8615384613e0 * X(141) ^ 0.1e1 - 0.3610604490e5 * X(6) ^ 0.8293838859e0 * X(150) ^ 0.1e1;  ERG(7,1) = 0.9014719843e5 * X(5) ^ 0.8000000000e0 * X(23) ^ 0.5278118802e0 * X(134) ^ 0.1e1 + 0.2970055545e3 * X(8) ^ 0.9722222223e0 * X(151) ^ 0.1e1 + 0.2408344972e5 * X(3) ^ 0.4999999998e0 * X(154) ^ 0.1e1 + 0.3559423777e3 * X(18) ^ 0.9296482412e0 * X(151) ^ 0.1e1 + 0.8694450414e4 * X(19) ^ 0.9955924294e0 * X(151) ^ 0.1e1 - 0.2547853547e4 * X(7) ^ 0.5000000002e0 * X(153) ^ 0.1e1 - 0.1049201131e2 * X(2) ^ (-0.3320804470e-2) * X(5) ^ (-0.2397510852e-1) * X(7) ^ 0.9629101285e0 * X(15) ^ 0.1685e1 * X(133) ^ 0.1e1 - 0.3771292981e5 * X(7) ^ 0.4999999997e0 * X(143) ^ 0.1e1;  ERG(8,1) = 0.1049201131e2 * X(2) ^ (-0.3320804470e-2) * X(5) ^ (-0.2397510852e-1) * X(7) ^ 0.9629101285e0 * X(15) ^ 0.1685e1 * X(133) ^ 0.1e1 + 0.1085002492e2 * X(2) ^ (-0.3358742751e-2) * X(3) ^ 0.9739478958e0 * X(5) ^ (-0.2424327076e-1) * X(15) ^ 0.1685e1 * X(133) ^ 0.1e1 + 0.3031347641e-2 * X(20) ^ 0.5e0 * X(37) ^ 0.5e0 - 0.2970055545e3 * X(8) ^ 0.9722222223e0 * X(151) ^ 0.1e1 - 0.6751417734e3 * X(8) ^ 0.5000000000e0 * X(135) ^ 0.1e1 - 0.8910166634e2 * X(8) ^ 0.9722222223e0 * X(164) ^ 0.1e1 - 0.2030671395e-1 * X(8) ^ 0.5e0 * X(32) ^ 0.5e0;  ERG(9,1) = 0.2512729767e4 * X(11) ^ 0.9940357859e0 * X(140) ^ 0.1e1 - 0.8931564995e3 * X(2) ^ (-0.6568638313e-2) * X(5) ^ (-0.1305154574e-1) * X(9) ^ 0.9933310569e0 * X(11) ^ 0.235e0 * X(13) ^ 0.2063074440e-3 * X(14) ^ (-0.5910831328e0) * X(15) ^ 0.88e-1 * X(16) ^ (-0.2704826039e0) * X(138) ^ 0.1e1 - 0.1187734899e3 * X(9) ^ 0.9716382669e0 * X(16) ^ 0.3376749592e-2 * X(126) ^ 0.1e1;  ERG(10,1) = 0.8931564995e3 * X(2) ^ (-0.6568638313e-2) * X(5) ^ (-0.1305154574e-1) * X(9) ^ 0.9933310569e0 * X(11) ^ 0.235e0 * X(13) ^ 0.2063074440e-3 * X(14) ^ (-0.5910831328e0) * X(15) ^ 0.88e-1 * X(16) ^ (-0.2704826039e0) * X(138) ^ 0.1e1 - 0.9465130187e5 * X(10) ^ 0.5000000001e0 * X(156) ^ 0.1e1;  ERG(11,1) = 0.1141036810e6 * X(10) ^ 0.1493e0 * X(12) ^ 0.1e1 * X(149) ^ 0.1e1 - 0.9455645805e2 * X(2) ^ (-0.2071563088e-1) * X(5) ^ (-0.5022831050e-1) * X(9) ^ 0.326e0 * X(11) ^ 0.4230769231e0 * X(15) ^ 0.248e0 * X(139) ^ 0.1e1 - 0.2512729767e4 * X(11) ^ 0.9940357859e0 * X(140) ^ 0.1e1;  ERG(12,1) = 0.2306702893e5 * X(130) ^ 0.1e1 * X(158) ^ 0.9975062347e0 + 0.4768620195e3 * X(24) ^ 0.1318391563e0 * X(25) ^ 0.7910349154e-2 * X(152) ^ 0.1e1 + 0.2224471846e4 * X(4) ^ 0.9604829853e0 * X(150) ^ 0.1e1 + 0.3610604490e5 * X(6) ^ 0.8293838859e0 * X(150) ^ 0.1e1 + 0.9967272082e1 * X(33) ^ 0.385e0 * X(180) ^ 0.1e1 + 0.2142776130e2 * X(34) ^ 0.385e0 * X(180) ^ 0.1e1 + 0.3671477823e2 * X(35) ^ 0.385e0 * X(180) ^ 0.1e1 + 0.5955208746e5 * X(40) ^ 0.385e0 * X(182) ^ 0.1e1 - 0.1742688761e6 * X(12) ^ 0.9986438167e0 * X(13) ^ 0.1980849053e0 * X(157) ^ 0.1e1 - 0.1141036810e6 * X(10) ^ 0.1493e0 * X(12) ^ 0.1e1 * X(149) ^ 0.1e1 - 0.2940873226e3 * X(12) ^ 0.1e1 * X(148) ^ 0.1e1 - 0.1683472807e5 * X(12) ^ 0.9999230829e0 * X(24) ^ 0.4157339305e0 * X(159) ^ 0.1e1 - 0.3543221570e4 * X(12) ^ 0.9998550936e0 * X(30) ^ 0.827e0 * X(181) ^ 0.1e1 - 0.4075356854e4 * X(12) ^ 0.9998550936e0 * X(31) ^ 0.827e0 * X(181) ^ 0.1e1 - 0.4169102075e4 * X(12) ^ 0.9998550936e0 * X(32) ^ 0.827e0 * X(183) ^ 0.1e1;  ERG(13,1) = 0.3511209420e3 * X(131) ^ 0.1e1 * X(137) ^ 0.1663551401e0 + 0.8017109569e3 * X(165) ^ 0.1e1 * X(166) ^ 0.3984637534e-1 - 0.8931564995e3 * X(2) ^ (-0.6568638313e-2) * X(5) ^ (-0.1305154574e-1) * X(9) ^ 0.9933310569e0 * X(11) ^ 0.235e0 * X(13) ^ 0.2063074440e-3 * X(14) ^ (-0.5910831328e0) * X(15) ^ 0.88e-1 * X(16) ^ (-0.2704826039e0) * X(138) ^ 0.1e1 - 0.1742688761e6 * X(12) ^ 0.9986438167e0 * X(13) ^ 0.1980849053e0 * X(157) ^ 0.1e1 - 0.2351877266e4 * X(13) ^ 0.1999999997e0 * X(132) ^ 0.1e1;  ERG(14,1) = 0.9455645805e2 * X(2) ^ (-0.2071563088e-1) * X(5) ^ (-0.5022831050e-1) * X(9) ^ 0.326e0 * X(11) ^ 0.4230769231e0 * X(15) ^ 0.248e0 * X(139) ^ 0.1e1 + 0.1049201131e2 * X(2) ^ (-0.3320804470e-2) * X(5) ^ (-0.2397510852e-1) * X(7) ^ 0.9629101285e0 * X(15) ^ 0.1685e1 * X(133) ^ 0.1e1 + 0.1085002492e2 * X(2) ^ (-0.3358742751e-2) * X(3) ^ 0.9739478958e0 * X(5) ^ (-0.2424327076e-1) * X(15) ^ 0.1685e1 * X(133) ^ 0.1e1 + 0.6574294837e1 * X(15) ^ 0.1685e1 * X(18) ^ 0.5000000001e0 * X(155) ^ 0.1e1 - 0.2010896717e1 * X(14) ^ 0.2344299609e0 * X(17) ^ 0.5000000003e0 * X(145) ^ 0.1e1 - 0.9126577402e3 * X(14) ^ 0.4260599794e0 * X(142) ^ 0.1e1;  ERG(15,1) = 0.1187734899e3 * X(9) ^ 0.9716382669e0 * X(16) ^ 0.3376749592e-2 * X(126) ^ 0.1e1 - 0.1049201131e2 * X(2) ^ (-0.3320804470e-2) * X(5) ^ (-0.2397510852e-1) * X(7) ^ 0.9629101285e0 * X(15) ^ 0.1685e1 * X(133) ^ 0.1e1 - 0.1085002492e2 * X(2) ^ (-0.3358742751e-2) * X(3) ^ 0.9739478958e0 * X(5) ^ (-0.2424327076e-1) * X(15) ^ 0.1685e1 * X(133) ^ 0.1e1 - 0.6250743872e2 * X(15) ^ 0.9441006585e0 * X(128) ^ 0.3344404739e-2 * X(144) ^ 0.1e1 - 0.6574294837e1 * X(15) ^ 0.1685e1 * X(18) ^ 0.5000000001e0 * X(155) ^ 0.1e1 - 0.1481189310e2 * X(15) ^ 0.8178844057e0 * X(168) ^ 0.1e1;  ERG(16,1) = 0.5720507542e2 * X(146) ^ 0.1e1 * X(147) ^ 0.5008488966e0 - 0.1187734899e3 * X(9) ^ 0.9716382669e0 * X(16) ^ 0.3376749592e-2 * X(126) ^ 0.1e1;  ERG(17,1) = 0.2224471846e4 * X(4) ^ 0.9604829853e0 * X(150) ^ 0.1e1 + 0.3610604490e5 * X(6) ^ 0.8293838859e0 * X(150) ^ 0.1e1 - 0.2010896717e1 * X(14) ^ 0.2344299609e0 * X(17) ^ 0.5000000003e0 * X(145) ^ 0.1e1;  ERG(18,1) = 0.6751417734e3 * X(8) ^ 0.5000000000e0 * X(135) ^ 0.1e1 + 0.2587449087e-2 * X(21) ^ 0.5e0 * X(37) ^ 0.5e0 - 0.1525467333e3 * X(18) ^ 0.9296482412e0 * X(164) ^ 0.1e1 - 0.3559423777e3 * X(18) ^ 0.9296482412e0 * X(151) ^ 0.1e1 - 0.6574294837e1 * X(15) ^ 0.1685e1 * X(18) ^ 0.5000000001e0 * X(155) ^ 0.1e1 - 0.1733307912e-1 * X(18) ^ 0.5e0 * X(32) ^ 0.5e0;  ERG(19,1) = 0.6574294837e1 * X(15) ^ 0.1685e1 * X(18) ^ 0.5000000001e0 * X(155) ^ 0.1e1 + 0.2763393752e-2 * X(22) ^ 0.5e0 * X(37) ^ 0.5e0 - 0.5371916275e4 * X(19) ^ 0.9955924294e0 * X(164) ^ 0.1e1 - 0.8694450414e4 * X(19) ^ 0.9955924294e0 * X(151) ^ 0.1e1 - 0.3517226030e-1 * X(19) ^ 0.5e0 * X(32) ^ 0.5e0;  ERG(20,1) = 0.2030671395e-1 * X(8) ^ 0.5e0 * X(32) ^ 0.5e0 - 0.3031347641e-2 * X(20) ^ 0.5e0 * X(37) ^ 0.5e0;  ERG(21,1) = 0.1733307912e-1 * X(18) ^ 0.5e0 * X(32) ^ 0.5e0 - 0.2587449087e-2 * X(21) ^ 0.5e0 * X(37) ^ 0.5e0;  ERG(22,1) = 0.3517226030e-1 * X(19) ^ 0.5e0 * X(32) ^ 0.5e0 - 0.2763393752e-2 * X(22) ^ 0.5e0 * X(37) ^ 0.5e0;  ERG(23,1) = 0.1683472807e5 * X(12) ^ 0.9999230829e0 * X(24) ^ 0.4157339305e0 * X(159) ^ 0.1e1 - 0.5831662556e7 * X(2) ^ 0.9642857143e0 * X(23) ^ 0.5278118802e0 * X(134) ^ 0.1e1 - 0.9014719843e5 * X(5) ^ 0.8000000000e0 * X(23) ^ 0.5278118802e0 * X(134) ^ 0.1e1;  ERG(24,1) = 0.1725637757e2 * X(12) ^ (-0.4577407228e-1) * X(23) ^ (-0.1583280558e0) * X(25) ^ 0.4460212912e-1 * X(128) ^ 0.3750000000e0 * X(160) ^ 0.1e1 - 0.1683472807e5 * X(12) ^ 0.9999230829e0 * X(24) ^ 0.4157339305e0 * X(159) ^ 0.1e1 - 0.4768620195e3 * X(24) ^ 0.1318391563e0 * X(25) ^ 0.7910349154e-2 * X(152) ^ 0.1e1;  ERG(25,1) = 0.1226153687e1 * X(123) ^ 0.1e1 * X(124) ^ 0.7411630560e0 + 0.9234698783e-2 * X(12) ^ (-0.111e0) * X(38) ^ 0.6314511210e-1 * X(128) ^ 0.5000000001e0 * X(161) ^ 0.7986577182e0 * X(163) ^ 0.1e1 - 0.1725637757e2 * X(12) ^ (-0.4577407228e-1) * X(23) ^ (-0.1583280558e0) * X(25) ^ 0.4460212912e-1 * X(128) ^ 0.3750000000e0 * X(160) ^ 0.1e1 - 0.4768620195e3 * X(24) ^ 0.1318391563e0 * X(25) ^ 0.7910349154e-2 * X(152) ^ 0.1e1 - 0.1637319996e2 * X(25) ^ 0.3039999999e0 * X(32) ^ (-0.5000000000e0) * X(171) ^ 0.1e1;  ERG(26,1) = 0.1637319996e2 * X(25) ^ 0.3039999999e0 * X(32) ^ (-0.5000000000e0) * X(171) ^ 0.1e1 - 0.1052459060e5 * X(26) ^ 0.9977827049e0 * X(172) ^ 0.1e1;  ERG(27,1) = 0.1052459060e5 * X(26) ^ 0.9977827049e0 * X(172) ^ 0.1e1 - 0.9642748842e5 * X(27) ^ 0.9975669099e0 * X(173) ^ 0.1e1;  ERG(28,1) = 0.9642748842e5 * X(27) ^ 0.9975669099e0 * X(173) ^ 0.1e1 - 0.2350954134e5 * X(28) ^ 0.5e0 * X(174) ^ 0.1e1 - 0.1837283321e4 * X(28) ^ 0.5000000000e0 * X(179) ^ 0.1e1;  ERG(29,1) = 0.2350954134e5 * X(28) ^ 0.5e0 * X(174) ^ 0.1e1 - 0.7396817205e5 * X(29) ^ 0.597e0 * X(175) ^ 0.1e1;  ERG(30,1) = 0.7396817205e5 * X(29) ^ 0.597e0 * X(175) ^ 0.1e1 + 0.9967272082e1 * X(33) ^ 0.385e0 * X(180) ^ 0.1e1 - 0.8703177136e8 * X(30) ^ 0.662e0 * X(176) ^ 0.1e1 - 0.3543221570e4 * X(12) ^ 0.9998550936e0 * X(30) ^ 0.827e0 * X(181) ^ 0.1e1;  ERG(31,1) = 0.8703177136e8 * X(30) ^ 0.662e0 * X(176) ^ 0.1e1 + 0.2142776130e2 * X(34) ^ 0.385e0 * X(180) ^ 0.1e1 - 0.1242509645e5 * X(31) ^ 0.557e0 * X(177) ^ 0.1e1 - 0.4075356854e4 * X(12) ^ 0.9998550936e0 * X(31) ^ 0.827e0 * X(181) ^ 0.1e1;  ERG(32,1) = 0.1242509645e5 * X(31) ^ 0.557e0 * X(177) ^ 0.1e1 + 0.5563355615e-1 * X(39) + 0.2209706584e-1 * X(20) ^ 0.5e0 * X(21) ^ 0.5e0 * X(22) ^ 0.5e0 * X(37) ^ 0.5e0 + 0.3671477823e2 * X(35) ^ 0.385e0 * X(180) ^ 0.1e1 - 0.2956492539e0 * X(32) - 0.4169102075e4 * X(12) ^ 0.9998550936e0 * X(32) ^ 0.827e0 * X(183) ^ 0.1e1 - 0.1471655572e1 * X(8) ^ 0.5e0 * X(18) ^ 0.5e0 * X(19) ^ 0.5e0 * X(32) ^ 0.5e0 - 0.8610295570e3 * X(32) ^ 0.5000000000e0 * X(186) ^ 0.1e1;  ERG(33,1) = 0.3543221570e4 * X(12) ^ 0.9998550936e0 * X(30) ^ 0.827e0 * X(181) ^ 0.1e1 - 0.9967272082e1 * X(33) ^ 0.385e0 * X(180) ^ 0.1e1;  ERG(34,1) = 0.4075356854e4 * X(12) ^ 0.9998550936e0 * X(31) ^ 0.827e0 * X(181) ^ 0.1e1 - 0.2142776130e2 * X(34) ^ 0.385e0 * X(180) ^ 0.1e1;  ERG(35,1) = 0.4169102075e4 * X(12) ^ 0.9998550936e0 * X(32) ^ 0.827e0 * X(183) ^ 0.1e1 + 0.3192737733e0 * X(40) ^ 0.5e0 - 0.3671477823e2 * X(35) ^ 0.385e0 * X(180) ^ 0.1e1 - 0.1659444132e-1 * X(35);  ERG(36,1) = 0.3142262948e-1 * X(39) + 0.3491403276e-1 * X(37) - 0.6137232322e0 * X(36) - 0.1939652307e0 * X(20) ^ 0.123e1 * X(36) ^ 0.123e1 - 0.1803401715e0 * X(21) ^ 0.123e1 * X(36) ^ 0.123e1 - 0.3435069860e0 * X(22) ^ 0.123e1 * X(36) ^ 0.123e1;  ERG(37,1) = 0.1939652307e0 * X(20) ^ 0.123e1 * X(36) ^ 0.123e1 + 0.1803401715e0 * X(21) ^ 0.123e1 * X(36) ^ 0.123e1 + 0.3435069860e0 * X(22) ^ 0.123e1 * X(36) ^ 0.123e1 + 0.1471655572e1 * X(8) ^ 0.5e0 * X(18) ^ 0.5e0 * X(19) ^ 0.5e0 * X(32) ^ 0.5e0 - 0.2209706584e-1 * X(20) ^ 0.5e0 * X(21) ^ 0.5e0 * X(22) ^ 0.5e0 * X(37) ^ 0.5e0 - 0.3491403276e-1 * X(37);  ERG(38,1) = 0.1397815044e-2 * X(125) ^ 0.104e1 + 0.7108537236e-2 * X(122) ^ 0.1e1 * X(124) ^ 0.6961178048e0 - 0.9234698783e-2 * X(12) ^ (-0.111e0) * X(38) ^ 0.6314511210e-1 * X(128) ^ 0.5000000001e0 * X(161) ^ 0.7986577182e0 * X(163) ^ 0.1e1;  ERG(39,1) = 0.2956492539e0 * X(32) + 0.6137232322e0 * X(36) + 0.5955208746e5 * X(40) ^ 0.385e0 * X(182) ^ 0.1e1 - 0.8705618563e-1 * X(39);  ERG(40,1) = 0.1659444132e-1 * X(35) - 0.3192737733e0 * X(40) ^ 0.5e0 - 0.5955208746e5 * X(40) ^ 0.385e0 * X(182) ^ 0.1e1;    %Labeled ODE's:    ERG(41,1) = 0.1742688761e6 * (X(52) * X(93) + X(92) * X(53) + X(52) * X(53)) * X(12) ^ (-0.13561833e-2) * X(13) ^ (-0.8019150947e0) * X(157) ^ 0.1e1 - 0.6501052401e6 * X(41) * X(1) ^ (-0.67090703e-2) * X(127) ^ 0.1e1;  ERG(42,1) = 0.6501052401e6 * X(41) * X(1) ^ (-0.67090703e-2) * X(127) ^ 0.1e1 + 0.3057424256e4 * X(43) * X(3) ^ (-0.4999999999e0) * X(129) ^ 0.1e1 + 0.1201395274e2 * X(44) * X(4) ^ (-0.311418685e-1) * X(141) ^ 0.1e1 - 0.5831662556e7 * X(42) * X(2) ^ (-0.357142857e-1) * X(23) ^ 0.5278118802e0 * X(134) ^ 0.1e1 - 0.2266068147e4 * X(42) * X(2) ^ (-0.256410254e-1) * X(128) ^ 0.2222222230e-1 * X(136) ^ 0.1e1 - 0.5810000000e4 * X(42) * X(2) ^ (-0.5000000000e0) * X(154) ^ 0.1e1;  ERG(43,1) = 0.5831662556e7 * (X(42) * X(103) + X(82) * X(63) + X(42) * X(63)) * X(2) ^ (-0.357142857e-1) * X(23) ^ (-0.4721881198e0) * X(134) ^ 0.1e1 + 0.8910166634e2 * X(48) * X(8) ^ (-0.277777777e-1) * X(164) ^ 0.1e1 + 0.1525467333e3 * X(58) * X(18) ^ (-0.703517588e-1) * X(164) ^ 0.1e1 + 0.5371916275e4 * X(59) * X(19) ^ (-0.44075706e-2) * X(164) ^ 0.1e1 - 0.3057424256e4 * X(43) * X(3) ^ (-0.4999999999e0) * X(129) ^ 0.1e1 - 0.2408344972e5 * X(43) * X(3) ^ (-0.5000000002e0) * X(154) ^ 0.1e1 - 0.1085002492e2 * X(43) * X(3) ^ (-0.260521042e-1) * X(2) ^ (-0.3358742751e-2) * X(5) ^ (-0.2424327076e-1) * X(15) ^ 0.1685e1 * X(133) ^ 0.1e1;  ERG(44,1) = 0.2266068147e4 * X(42) * X(2) ^ (-0.256410254e-1) * X(128) ^ 0.2222222230e-1 * X(136) ^ 0.1e1 - 0.1201395274e2 * X(44) * X(4) ^ (-0.311418685e-1) * X(141) ^ 0.1e1 - 0.2224471846e4 * X(44) * X(4) ^ (-0.395170147e-1) * X(150) ^ 0.1e1;  ERG(45,1) = 0.5810000000e4 * X(42) * X(2) ^ (-0.5000000000e0) * X(154) ^ 0.1e1 + 0.2182238068e3 * X(46) * X(6) ^ (-0.1384615387e0) * X(141) ^ 0.1e1 + 0.2547853547e4 * X(47) * X(7) ^ (-0.4999999998e0) * X(153) ^ 0.1e1 - 0.9014719843e5 * X(45) * X(5) ^ (-0.2000000000e0) * X(23) ^ 0.5278118802e0 * X(134) ^ 0.1e1 - 0.6900945118e5 * X(45) * X(5) ^ (-0.400000000e-1) * X(128) ^ 0.2222222230e-1 * X(136) ^ 0.1e1;  ERG(46,1) = 0.6900945118e5 * X(45) * X(5) ^ (-0.400000000e-1) * X(128) ^ 0.2222222230e-1 * X(136) ^ 0.1e1 - 0.2182238068e3 * X(46) * X(6) ^ (-0.1384615387e0) * X(141) ^ 0.1e1 - 0.3610604490e5 * X(46) * X(6) ^ (-0.1706161141e0) * X(150) ^ 0.1e1;  ERG(47,1) = 0.9014719843e5 * (X(45) * X(103) + X(85) * X(63) + X(45) * X(63)) * X(5) ^ (-0.2000000000e0) * X(23) ^ (-0.4721881198e0) * X(134) ^ 0.1e1 + 0.2970055545e3 * X(48) * X(8) ^ (-0.277777777e-1) * X(151) ^ 0.1e1 + 0.2408344972e5 * X(43) * X(3) ^ (-0.5000000002e0) * X(154) ^ 0.1e1 + 0.3559423777e3 * X(58) * X(18) ^ (-0.703517588e-1) * X(151) ^ 0.1e1 + 0.8694450414e4 * X(59) * X(19) ^ (-0.44075706e-2) * X(151) ^ 0.1e1 - 0.2547853547e4 * X(47) * X(7) ^ (-0.4999999998e0) * X(153) ^ 0.1e1 - 0.1049201131e2 * X(47) * X(7) ^ (-0.370898715e-1) * X(2) ^ (-0.3320804470e-2) * X(5) ^ (-0.2397510852e-1) * X(15) ^ 0.1685e1 * X(133) ^ 0.1e1 - 0.3771292981e5 * X(47) * X(7) ^ (-0.5000000003e0) * X(143) ^ 0.1e1;  ERG(48,1) = 0.1049201131e2 * (X(47) * X(95) + X(87) * X(55) + X(47) * X(55)) * X(7) ^ (-0.370898715e-1) * X(15) ^ 0.685e0 * X(2) ^ (-0.3320804470e-2) * X(5) ^ (-0.2397510852e-1) * X(133) ^ 0.1e1 + 0.1085002492e2 * (X(43) * X(95) + X(83) * X(55) + X(43) * X(55)) * X(3) ^ (-0.260521042e-1) * X(15) ^ 0.685e0 * X(2) ^ (-0.3358742751e-2) * X(5) ^ (-0.2424327076e-1) * X(133) ^ 0.1e1 + 0.3031347641e-2 * X(60) * X(20) ^ (-0.5e0) * X(37) ^ 0.5e0 - 0.2970055545e3 * X(48) * X(8) ^ (-0.277777777e-1) * X(151) ^ 0.1e1 - 0.6751417734e3 * X(48) * X(8) ^ (-0.5000000000e0) * X(135) ^ 0.1e1 - 0.8910166634e2 * X(48) * X(8) ^ (-0.277777777e-1) * X(164) ^ 0.1e1 - 0.2030671395e-1 * X(48) * X(8) ^ (-0.5e0) * X(32) ^ 0.5e0;  ERG(49,1) = 0.2512729767e4 * X(51) * X(11) ^ (-0.59642141e-2) * X(140) ^ 0.1e1 - 0.8931564995e3 * X(49) * X(9) ^ (-0.66689431e-2) * X(2) ^ (-0.6568638313e-2) * X(5) ^ (-0.1305154574e-1) * X(11) ^ 0.235e0 * X(13) ^ 0.2063074440e-3 * X(14) ^ (-0.5910831328e0) * X(15) ^ 0.88e-1 * X(16) ^ (-0.2704826039e0) * X(138) ^ 0.1e1 - 0.1187734899e3 * X(49) * X(9) ^ (-0.283617331e-1) * X(16) ^ 0.3376749592e-2 * X(126) ^ 0.1e1;  ERG(50,1) = 0.8931564995e3 * (X(49) * X(93) + X(89) * X(53) + X(49) * X(53)) * X(9) ^ (-0.66689431e-2) * X(13) ^ (-0.9997936926e0) * X(2) ^ (-0.6568638313e-2) * X(5) ^ (-0.1305154574e-1) * X(11) ^ 0.235e0 * X(14) ^ (-0.5910831328e0) * X(15) ^ 0.88e-1 * X(16) ^ (-0.2704826039e0) * X(138) ^ 0.1e1 - 0.9465130187e5 * X(50) * X(10) ^ (-0.4999999999e0) * X(156) ^ 0.1e1;  ERG(51,1) = 0.1141036810e6 * X(52) * X(10) ^ 0.1493e0 * X(149) ^ 0.1e1 - 0.9455645805e2 * X(51) * X(11) ^ (-0.5769230769e0) * X(2) ^ (-0.2071563088e-1) * X(5) ^ (-0.5022831050e-1) * X(9) ^ 0.326e0 * X(15) ^ 0.248e0 * X(139) ^ 0.1e1 - 0.2512729767e4 * X(51) * X(11) ^ (-0.59642141e-2) * X(140) ^ 0.1e1;  ERG(52,1) = 0.2306702893e5 * X(169) / X(158) * X(130) ^ 0.1e1 * X(158) ^ 0.9975062347e0 + 0.4768620195e3 * (X(64) * X(105) + X(104) * X(65) + X(64) * X(65)) * X(24) ^ (-0.8681608437e0) * X(25) ^ (-0.9920896508e0) * X(152) ^ 0.1e1 + 0.2224471846e4 * X(44) * X(4) ^ (-0.395170147e-1) * X(150) ^ 0.1e1 + 0.3610604490e5 * X(46) * X(6) ^ (-0.1706161141e0) * X(150) ^ 0.1e1 + 0.9967272082e1 * X(73) * X(33) ^ (-0.615e0) * X(180) ^ 0.1e1 + 0.2142776130e2 * X(74) * X(34) ^ (-0.615e0) * X(180) ^ 0.1e1 + 0.3671477823e2 * X(75) * X(35) ^ (-0.615e0) * X(180) ^ 0.1e1 + 0.5955208746e5 * X(80) * X(40) ^ (-0.615e0) * X(182) ^ 0.1e1 - 0.1742688761e6 * X(52) * X(12) ^ (-0.13561833e-2) * X(13) ^ 0.1980849053e0 * X(157) ^ 0.1e1 - 0.1141036810e6 * X(52) * X(10) ^ 0.1493e0 * X(149) ^ 0.1e1 - 0.2940873226e3 * X(52) * X(148) ^ 0.1e1 - 0.1683472807e5 * X(52) * X(12) ^ (-0.769171e-4) * X(24) ^ 0.4157339305e0 * X(159) ^ 0.1e1 - 0.3543221570e4 * X(70) * X(30) ^ (-0.173e0) * X(12) ^ 0.9998550936e0 * X(181) ^ 0.1e1 - 0.4075356854e4 * X(71) * X(31) ^ (-0.173e0) * X(12) ^ 0.9998550936e0 * X(181) ^ 0.1e1 - 0.4169102075e4 * X(72) * X(32) ^ (-0.173e0) * X(12) ^ 0.9998550936e0 * X(183) ^ 0.1e1;  ERG(54,1) = 0.9455645805e2 * X(51) * X(11) ^ (-0.5769230769e0) * X(2) ^ (-0.2071563088e-1) * X(5) ^ (-0.5022831050e-1) * X(9) ^ 0.326e0 * X(15) ^ 0.248e0 * X(139) ^ 0.1e1 + 0.1049201131e2 * (X(47) * X(95) + X(87) * X(55) + X(47) * X(55)) * X(7) ^ (-0.370898715e-1) * X(15) ^ 0.685e0 * X(2) ^ (-0.3320804470e-2) * X(5) ^ (-0.2397510852e-1) * X(133) ^ 0.1e1 + 0.1085002492e2 * (X(43) * X(95) + X(83) * X(55) + X(43) * X(55)) * X(3) ^ (-0.260521042e-1) * X(15) ^ 0.685e0 * X(2) ^ (-0.3358742751e-2) * X(5) ^ (-0.2424327076e-1) * X(133) ^ 0.1e1 + 0.6574294837e1 * (X(58) * X(95) + X(98) * X(55) + X(58) * X(55)) * X(18) ^ (-0.4999999999e0) * X(15) ^ 0.685e0 * X(155) ^ 0.1e1 - 0.2010896717e1 * X(54) * X(14) ^ (-0.7655700391e0) * X(17) ^ 0.5000000003e0 * X(145) ^ 0.1e1 - 0.9126577402e3 * X(54) * X(14) ^ (-0.5739400206e0) * X(142) ^ 0.1e1;  ERG(55,1) = 0.1187734899e3 * (X(49) * X(96) + X(89) * X(56) + X(49) * X(56)) * X(9) ^ (-0.283617331e-1) * X(16) ^ (-0.9966232504e0) * X(126) ^ 0.1e1 - 0.1049201131e2 * X(55) * X(15) ^ 0.685e0 * X(2) ^ (-0.3320804470e-2) * X(5) ^ (-0.2397510852e-1) * X(7) ^ 0.9629101285e0 * X(133) ^ 0.1e1 - 0.1085002492e2 * X(55) * X(15) ^ 0.685e0 * X(2) ^ (-0.3358742751e-2) * X(3) ^ 0.9739478958e0 * X(5) ^ (-0.2424327076e-1) * X(133) ^ 0.1e1 - 0.6250743872e2 * X(55) * X(15) ^ (-0.558993415e-1) * X(128) ^ 0.3344404739e-2 * X(144) ^ 0.1e1 - 0.6574294837e1 * X(55) * X(15) ^ 0.685e0 * X(18) ^ 0.5000000001e0 * X(155) ^ 0.1e1 - 0.1481189310e2 * X(55) * X(15) ^ (-0.1821155943e0) * X(168) ^ 0.1e1;  ERG(57,1) = 0.2224471846e4 * X(44) * X(4) ^ (-0.395170147e-1) * X(150) ^ 0.1e1 + 0.3610604490e5 * X(46) * X(6) ^ (-0.1706161141e0) * X(150) ^ 0.1e1 - 0.2010896717e1 * X(57) * X(17) ^ (-0.4999999997e0) * X(14) ^ 0.2344299609e0 * X(145) ^ 0.1e1;  ERG(58,1) = 0.6751417734e3 * X(48) * X(8) ^ (-0.5000000000e0) * X(135) ^ 0.1e1 + 0.2587449087e-2 * X(61) * X(21) ^ (-0.5e0) * X(37) ^ 0.5e0 - 0.1525467333e3 * X(58) * X(18) ^ (-0.703517588e-1) * X(164) ^ 0.1e1 - 0.3559423777e3 * X(58) * X(18) ^ (-0.703517588e-1) * X(151) ^ 0.1e1 - 0.6574294837e1 * X(58) * X(18) ^ (-0.4999999999e0) * X(15) ^ 0.1685e1 * X(155) ^ 0.1e1 - 0.1733307912e-1 * X(58) * X(18) ^ (-0.5e0) * X(32) ^ 0.5e0;  ERG(59,1) = 0.6574294837e1 * (X(58) * X(95) + X(98) * X(55) + X(58) * X(55)) * X(18) ^ (-0.4999999999e0) * X(15) ^ 0.685e0 * X(155) ^ 0.1e1 + 0.2763393752e-2 * X(62) * X(22) ^ (-0.5e0) * X(37) ^ 0.5e0 - 0.5371916275e4 * X(59) * X(19) ^ (-0.44075706e-2) * X(164) ^ 0.1e1 - 0.8694450414e4 * X(59) * X(19) ^ (-0.44075706e-2) * X(151) ^ 0.1e1 - 0.3517226030e-1 * X(59) * X(19) ^ (-0.5e0) * X(32) ^ 0.5e0;  ERG(60,1) = 0.2030671395e-1 * X(48) * X(8) ^ (-0.5e0) * X(32) ^ 0.5e0 - 0.3031347641e-2 * X(60) * X(20) ^ (-0.5e0) * X(37) ^ 0.5e0;  ERG(61,1) = 0.1733307912e-1 * X(58) * X(18) ^ (-0.5e0) * X(32) ^ 0.5e0 - 0.2587449087e-2 * X(61) * X(21) ^ (-0.5e0) * X(37) ^ 0.5e0;  ERG(62,1) = 0.3517226030e-1 * X(59) * X(19) ^ (-0.5e0) * X(32) ^ 0.5e0 - 0.2763393752e-2 * X(62) * X(22) ^ (-0.5e0) * X(37) ^ 0.5e0;  ERG(63,1) = 0.1683472807e5 * (X(52) * X(104) + X(92) * X(64) + X(52) * X(64)) * X(12) ^ (-0.769171e-4) * X(24) ^ (-0.5842660695e0) * X(159) ^ 0.1e1 - 0.5831662556e7 * X(63) * X(23) ^ (-0.4721881198e0) * X(2) ^ 0.9642857143e0 * X(134) ^ 0.1e1 - 0.9014719843e5 * X(63) * X(23) ^ (-0.4721881198e0) * X(5) ^ 0.8000000000e0 * X(134) ^ 0.1e1;  ERG(64,1) = 0.1725637757e2 * X(65) * X(25) ^ (-0.9553978709e0) * X(12) ^ (-0.4577407228e-1) * X(23) ^ (-0.1583280558e0) * X(128) ^ 0.3750000000e0 * X(160) ^ 0.1e1 - 0.1683472807e5 * X(64) * X(24) ^ (-0.5842660695e0) * X(12) ^ 0.9999230829e0 * X(159) ^ 0.1e1 - 0.4768620195e3 * X(64) * X(24) ^ (-0.8681608437e0) * X(25) ^ 0.7910349154e-2 * X(152) ^ 0.1e1;  ERG(65,1) = 0.1226153687e1 * X(162) / X(124) * X(123) ^ 0.1e1 * X(124) ^ 0.7411630560e0 + 0.9234698783e-2 * X(78) * X(38) ^ (-0.9368548879e0) * X(12) ^ (-0.111e0) * X(128) ^ 0.5000000001e0 * X(161) ^ 0.7986577182e0 * X(163) ^ 0.1e1 - 0.1725637757e2 * X(65) * X(25) ^ (-0.9553978709e0) * X(12) ^ (-0.4577407228e-1) * X(23) ^ (-0.1583280558e0) * X(128) ^ 0.3750000000e0 * X(160) ^ 0.1e1 - 0.4768620195e3 * X(65) * X(25) ^ (-0.9920896508e0) * X(24) ^ 0.1318391563e0 * X(152) ^ 0.1e1 - 0.1637319996e2 * X(65) * X(25) ^ (-0.6960000001e0) * X(32) ^ (-0.5000000000e0) * X(171) ^ 0.1e1;  ERG(66,1) = 0.1637319996e2 * X(65) * X(25) ^ (-0.6960000001e0) * X(32) ^ (-0.5000000000e0) * X(171) ^ 0.1e1 - 0.1052459060e5 * X(66) * X(26) ^ (-0.22172951e-2) * X(172) ^ 0.1e1;  ERG(67,1) = 0.1052459060e5 * X(66) * X(26) ^ (-0.22172951e-2) * X(172) ^ 0.1e1 - 0.9642748842e5 * X(67) * X(27) ^ (-0.24330901e-2) * X(173) ^ 0.1e1;  ERG(68,1) = 0.9642748842e5 * X(67) * X(27) ^ (-0.24330901e-2) * X(173) ^ 0.1e1 - 0.2350954134e5 * X(68) * X(28) ^ (-0.5e0) * X(174) ^ 0.1e1 - 0.1837283321e4 * X(68) * X(28) ^ (-0.5000000000e0) * X(179) ^ 0.1e1;  ERG(69,1) = 0.2350954134e5 * X(68) * X(28) ^ (-0.5e0) * X(174) ^ 0.1e1 - 0.7396817205e5 * X(69) * X(29) ^ (-0.403e0) * X(175) ^ 0.1e1;  ERG(70,1) = 0.7396817205e5 * X(69) * X(29) ^ (-0.403e0) * X(175) ^ 0.1e1 + 0.9967272082e1 * X(73) * X(33) ^ (-0.615e0) * X(180) ^ 0.1e1 - 0.8703177136e8 * X(70) * X(30) ^ (-0.338e0) * X(176) ^ 0.1e1 - 0.3543221570e4 * X(70) * X(30) ^ (-0.173e0) * X(12) ^ 0.9998550936e0 * X(181) ^ 0.1e1;  ERG(71,1) = 0.8703177136e8 * X(70) * X(30) ^ (-0.338e0) * X(176) ^ 0.1e1 + 0.2142776130e2 * X(74) * X(34) ^ (-0.615e0) * X(180) ^ 0.1e1 - 0.1242509645e5 * X(71) * X(31) ^ (-0.443e0) * X(177) ^ 0.1e1 - 0.4075356854e4 * X(71) * X(31) ^ (-0.173e0) * X(12) ^ 0.9998550936e0 * X(181) ^ 0.1e1;  ERG(72,1) = 0.1242509645e5 * X(71) * X(31) ^ (-0.443e0) * X(177) ^ 0.1e1 + 0.5563355615e-1 * X(79) + 0.2209706584e-1 * X(77) * X(37) ^ (-0.5e0) * X(20) ^ 0.5e0 * X(21) ^ 0.5e0 * X(22) ^ 0.5e0 + 0.3671477823e2 * X(75) * X(35) ^ (-0.615e0) * X(180) ^ 0.1e1 - 0.2956492539e0 * X(72) - 0.4169102075e4 * X(72) * X(32) ^ (-0.173e0) * X(12) ^ 0.9998550936e0 * X(183) ^ 0.1e1 - 0.1471655572e1 * X(72) * X(32) ^ (-0.5e0) * X(8) ^ 0.5e0 * X(18) ^ 0.5e0 * X(19) ^ 0.5e0 - 0.8610295570e3 * X(72) * X(32) ^ (-0.5000000000e0) * X(186) ^ 0.1e1;  ERG(73,1) = 0.3543221570e4 * X(70) * X(30) ^ (-0.173e0) * X(12) ^ 0.9998550936e0 * X(181) ^ 0.1e1 - 0.9967272082e1 * X(73) * X(33) ^ (-0.615e0) * X(180) ^ 0.1e1;  ERG(74,1) = 0.4075356854e4 * X(71) * X(31) ^ (-0.173e0) * X(12) ^ 0.9998550936e0 * X(181) ^ 0.1e1 - 0.2142776130e2 * X(74) * X(34) ^ (-0.615e0) * X(180) ^ 0.1e1;  ERG(75,1) = 0.4169102075e4 * X(72) * X(32) ^ (-0.173e0) * X(12) ^ 0.9998550936e0 * X(183) ^ 0.1e1 + 0.3192737733e0 * X(80) * X(40) ^ (-0.5e0) - 0.3671477823e2 * X(75) * X(35) ^ (-0.615e0) * X(180) ^ 0.1e1 - 0.1659444132e-1 * X(75);  ERG(76,1) = 0.3142262948e-1 * X(79) + 0.3491403276e-1 * X(77) - 0.6137232322e0 * X(76) - 0.1939652307e0 * X(76) * X(36) ^ 0.23e0 * X(20) ^ 0.123e1 - 0.1803401715e0 * X(76) * X(36) ^ 0.23e0 * X(21) ^ 0.123e1 - 0.3435069860e0 * X(76) * X(36) ^ 0.23e0 * X(22) ^ 0.123e1;  ERG(77,1) = 0.1939652307e0 * X(76) * X(36) ^ 0.23e0 * X(20) ^ 0.123e1 + 0.1803401715e0 * X(76) * X(36) ^ 0.23e0 * X(21) ^ 0.123e1 + 0.3435069860e0 * X(76) * X(36) ^ 0.23e0 * X(22) ^ 0.123e1 + 0.1471655572e1 * X(72) * X(32) ^ (-0.5e0) * X(8) ^ 0.5e0 * X(18) ^ 0.5e0 * X(19) ^ 0.5e0 - 0.2209706584e-1 * X(77) * X(37) ^ (-0.5e0) * X(20) ^ 0.5e0 * X(21) ^ 0.5e0 * X(22) ^ 0.5e0 - 0.3491403276e-1 * X(77);  ERG(78,1) = 0.1397815044e-2 * X(187) / X(125) * X(125) ^ 0.104e1 + 0.7108537236e-2 * X(162) / X(124) * X(122) ^ 0.1e1 * X(124) ^ 0.6961178048e0 - 0.9234698783e-2 * X(78) * X(38) ^ (-0.9368548879e0) * X(12) ^ (-0.111e0) * X(128) ^ 0.5000000001e0 * X(161) ^ 0.7986577182e0 * X(163) ^ 0.1e1;  ERG(79,1) = 0.2956492539e0 * X(72) + 0.6137232322e0 * X(76) + 0.5955208746e5 * X(80) * X(40) ^ (-0.615e0) * X(182) ^ 0.1e1 - 0.8705618563e-1 * X(79);  ERG(80,1) = 0.1659444132e-1 * X(75) - 0.3192737733e0 * X(80) * X(40) ^ (-0.5e0) - 0.5955208746e5 * X(80) * X(40) ^ (-0.615e0) * X(182) ^ 0.1e1;    % Unlabeled ODE's    ERG(81,1) = 0.1742688761e6 * X(92) * X(93) * X(12) ^ (-0.13561833e-2) * X(13) ^ (-0.8019150947e0) * X(157) ^ 0.1e1 - 0.6501052401e6 * X(81) * X(1) ^ (-0.67090703e-2) * X(127) ^ 0.1e1;  ERG(82,1) = 0.6501052401e6 * X(81) * X(1) ^ (-0.67090703e-2) * X(127) ^ 0.1e1 + 0.3057424256e4 * X(83) * X(3) ^ (-0.4999999999e0) * X(129) ^ 0.1e1 + 0.1201395274e2 * X(84) * X(4) ^ (-0.311418685e-1) * X(141) ^ 0.1e1 - 0.5831662556e7 * X(82) * X(2) ^ (-0.357142857e-1) * X(23) ^ 0.5278118802e0 * X(134) ^ 0.1e1 - 0.2266068147e4 * X(82) * X(2) ^ (-0.256410254e-1) * X(128) ^ 0.2222222230e-1 * X(136) ^ 0.1e1 - 0.5810000000e4 * X(82) * X(2) ^ (-0.5000000000e0) * X(154) ^ 0.1e1;  ERG(83,1) = 0.5831662556e7 * X(82) * X(103) * X(2) ^ (-0.357142857e-1) * X(23) ^ (-0.4721881198e0) * X(134) ^ 0.1e1 + 0.8910166634e2 * X(88) * X(8) ^ (-0.277777777e-1) * X(164) ^ 0.1e1 + 0.1525467333e3 * X(98) * X(18) ^ (-0.703517588e-1) * X(164) ^ 0.1e1 + 0.5371916275e4 * X(99) * X(19) ^ (-0.44075706e-2) * X(164) ^ 0.1e1 - 0.3057424256e4 * X(83) * X(3) ^ (-0.4999999999e0) * X(129) ^ 0.1e1 - 0.2408344972e5 * X(83) * X(3) ^ (-0.5000000002e0) * X(154) ^ 0.1e1 - 0.1085002492e2 * X(83) * X(3) ^ (-0.260521042e-1) * X(2) ^ (-0.3358742751e-2) * X(5) ^ (-0.2424327076e-1) * X(15) ^ 0.1685e1 * X(133) ^ 0.1e1;  ERG(84,1) = 0.2266068147e4 * X(82) * X(2) ^ (-0.256410254e-1) * X(128) ^ 0.2222222230e-1 * X(136) ^ 0.1e1 - 0.1201395274e2 * X(84) * X(4) ^ (-0.311418685e-1) * X(141) ^ 0.1e1 - 0.2224471846e4 * X(84) * X(4) ^ (-0.395170147e-1) * X(150) ^ 0.1e1;  ERG(85,1) = 0.5810000000e4 * X(82) * X(2) ^ (-0.5000000000e0) * X(154) ^ 0.1e1 + 0.2182238068e3 * X(86) * X(6) ^ (-0.1384615387e0) * X(141) ^ 0.1e1 + 0.2547853547e4 * X(87) * X(7) ^ (-0.4999999998e0) * X(153) ^ 0.1e1 - 0.9014719843e5 * X(85) * X(5) ^ (-0.2000000000e0) * X(23) ^ 0.5278118802e0 * X(134) ^ 0.1e1 - 0.6900945118e5 * X(85) * X(5) ^ (-0.400000000e-1) * X(128) ^ 0.2222222230e-1 * X(136) ^ 0.1e1;  ERG(86,1) = 0.6900945118e5 * X(85) * X(5) ^ (-0.400000000e-1) * X(128) ^ 0.2222222230e-1 * X(136) ^ 0.1e1 - 0.2182238068e3 * X(86) * X(6) ^ (-0.1384615387e0) * X(141) ^ 0.1e1 - 0.3610604490e5 * X(86) * X(6) ^ (-0.1706161141e0) * X(150) ^ 0.1e1;  ERG(87,1) = 0.9014719843e5 * X(85) * X(103) * X(5) ^ (-0.2000000000e0) * X(23) ^ (-0.4721881198e0) * X(134) ^ 0.1e1 + 0.2970055545e3 * X(88) * X(8) ^ (-0.277777777e-1) * X(151) ^ 0.1e1 + 0.2408344972e5 * X(83) * X(3) ^ (-0.5000000002e0) * X(154) ^ 0.1e1 + 0.3559423777e3 * X(98) * X(18) ^ (-0.703517588e-1) * X(151) ^ 0.1e1 + 0.8694450414e4 * X(99) * X(19) ^ (-0.44075706e-2) * X(151) ^ 0.1e1 - 0.2547853547e4 * X(87) * X(7) ^ (-0.4999999998e0) * X(153) ^ 0.1e1 - 0.1049201131e2 * X(87) * X(7) ^ (-0.370898715e-1) * X(2) ^ (-0.3320804470e-2) * X(5) ^ (-0.2397510852e-1) * X(15) ^ 0.1685e1 * X(133) ^ 0.1e1 - 0.3771292981e5 * X(87) * X(7) ^ (-0.5000000003e0) * X(143) ^ 0.1e1;  ERG(88,1) = 0.1049201131e2 * X(87) * X(95) * X(7) ^ (-0.370898715e-1) * X(15) ^ 0.685e0 * X(2) ^ (-0.3320804470e-2) * X(5) ^ (-0.2397510852e-1) * X(133) ^ 0.1e1 + 0.1085002492e2 * X(83) * X(95) * X(3) ^ (-0.260521042e-1) * X(15) ^ 0.685e0 * X(2) ^ (-0.3358742751e-2) * X(5) ^ (-0.2424327076e-1) * X(133) ^ 0.1e1 + 0.3031347641e-2 * X(100) * X(20) ^ (-0.5e0) * X(37) ^ 0.5e0 - 0.2970055545e3 * X(88) * X(8) ^ (-0.277777777e-1) * X(151) ^ 0.1e1 - 0.6751417734e3 * X(88) * X(8) ^ (-0.5000000000e0) * X(135) ^ 0.1e1 - 0.8910166634e2 * X(88) * X(8) ^ (-0.277777777e-1) * X(164) ^ 0.1e1 - 0.2030671395e-1 * X(88) * X(8) ^ (-0.5e0) * X(32) ^ 0.5e0;  ERG(89,1) = 0.2512729767e4 * X(91) * X(11) ^ (-0.59642141e-2) * X(140) ^ 0.1e1 - 0.8931564995e3 * X(89) * X(9) ^ (-0.66689431e-2) * X(2) ^ (-0.6568638313e-2) * X(5) ^ (-0.1305154574e-1) * X(11) ^ 0.235e0 * X(13) ^ 0.2063074440e-3 * X(14) ^ (-0.5910831328e0) * X(15) ^ 0.88e-1 * X(16) ^ (-0.2704826039e0) * X(138) ^ 0.1e1 - 0.1187734899e3 * X(89) * X(9) ^ (-0.283617331e-1) * X(16) ^ 0.3376749592e-2 * X(126) ^ 0.1e1;  ERG(90,1) = 0.8931564995e3 * X(89) * X(93) * X(9) ^ (-0.66689431e-2) * X(13) ^ (-0.9997936926e0) * X(2) ^ (-0.6568638313e-2) * X(5) ^ (-0.1305154574e-1) * X(11) ^ 0.235e0 * X(14) ^ (-0.5910831328e0) * X(15) ^ 0.88e-1 * X(16) ^ (-0.2704826039e0) * X(138) ^ 0.1e1 - 0.9465130187e5 * X(90) * X(10) ^ (-0.4999999999e0) * X(156) ^ 0.1e1;  ERG(91,1) = 0.1141036810e6 * X(92) * X(10) ^ 0.1493e0 * X(149) ^ 0.1e1 - 0.9455645805e2 * X(91) * X(11) ^ (-0.5769230769e0) * X(2) ^ (-0.2071563088e-1) * X(5) ^ (-0.5022831050e-1) * X(9) ^ 0.326e0 * X(15) ^ 0.248e0 * X(139) ^ 0.1e1 - 0.2512729767e4 * X(91) * X(11) ^ (-0.59642141e-2) * X(140) ^ 0.1e1;  ERG(92,1) = 0.2306702893e5 * X(184) / X(158) * X(130) ^ 0.1e1 * X(158) ^ 0.9975062347e0 + 0.4768620195e3 * X(104) * X(105) * X(24) ^ (-0.8681608437e0) * X(25) ^ (-0.9920896508e0) * X(152) ^ 0.1e1 + 0.2224471846e4 * X(84) * X(4) ^ (-0.395170147e-1) * X(150) ^ 0.1e1 + 0.3610604490e5 * X(86) * X(6) ^ (-0.1706161141e0) * X(150) ^ 0.1e1 + 0.9967272082e1 * X(113) * X(33) ^ (-0.615e0) * X(180) ^ 0.1e1 + 0.2142776130e2 * X(114) * X(34) ^ (-0.615e0) * X(180) ^ 0.1e1 + 0.3671477823e2 * X(115) * X(35) ^ (-0.615e0) * X(180) ^ 0.1e1 + 0.5955208746e5 * X(120) * X(40) ^ (-0.615e0) * X(182) ^ 0.1e1 - 0.1742688761e6 * X(92) * X(12) ^ (-0.13561833e-2) * X(13) ^ 0.1980849053e0 * X(157) ^ 0.1e1 - 0.1141036810e6 * X(92) * X(10) ^ 0.1493e0 * X(149) ^ 0.1e1 - 0.2940873226e3 * X(92) * X(148) ^ 0.1e1 - 0.1683472807e5 * X(92) * X(12) ^ (-0.769171e-4) * X(24) ^ 0.4157339305e0 * X(159) ^ 0.1e1 - 0.3543221570e4 * X(110) * X(30) ^ (-0.173e0) * X(12) ^ 0.9998550936e0 * X(181) ^ 0.1e1 - 0.4075356854e4 * X(111) * X(31) ^ (-0.173e0) * X(12) ^ 0.9998550936e0 * X(181) ^ 0.1e1 - 0.4169102075e4 * X(112) * X(32) ^ (-0.173e0) * X(12) ^ 0.9998550936e0 * X(183) ^ 0.1e1;  ERG(93,1) = 0.3511209420e3 * X(131) ^ 0.1e1 * X(137) ^ 0.1663551401e0 + 0.8017109569e3 * X(165) ^ 0.1e1 * X(166) ^ 0.3984637534e-1 - 0.8931564995e3 * X(93) * X(13) ^ (-0.9997936926e0) * X(2) ^ (-0.6568638313e-2) * X(5) ^ (-0.1305154574e-1) * X(9) ^ 0.9933310569e0 * X(11) ^ 0.235e0 * X(14) ^ (-0.5910831328e0) * X(15) ^ 0.88e-1 * X(16) ^ (-0.2704826039e0) * X(138) ^ 0.1e1 - 0.1742688761e6 * X(93) * X(13) ^ (-0.8019150947e0) * X(12) ^ 0.9986438167e0 * X(157) ^ 0.1e1 - 0.2351877266e4 * X(93) * X(13) ^ (-0.8000000003e0) * X(132) ^ 0.1e1;  ERG(94,1) = 0.9455645805e2 * X(91) * X(11) ^ (-0.5769230769e0) * X(2) ^ (-0.2071563088e-1) * X(5) ^ (-0.5022831050e-1) * X(9) ^ 0.326e0 * X(15) ^ 0.248e0 * X(139) ^ 0.1e1 + 0.1049201131e2 * X(87) * X(95) * X(7) ^ (-0.370898715e-1) * X(15) ^ 0.685e0 * X(2) ^ (-0.3320804470e-2) * X(5) ^ (-0.2397510852e-1) * X(133) ^ 0.1e1 + 0.1085002492e2 * X(83) * X(95) * X(3) ^ (-0.260521042e-1) * X(15) ^ 0.685e0 * X(2) ^ (-0.3358742751e-2) * X(5) ^ (-0.2424327076e-1) * X(133) ^ 0.1e1 + 0.6574294837e1 * X(98) * X(95) * X(18) ^ (-0.4999999999e0) * X(15) ^ 0.685e0 * X(155) ^ 0.1e1 - 0.2010896717e1 * X(94) * X(14) ^ (-0.7655700391e0) * X(17) ^ 0.5000000003e0 * X(145) ^ 0.1e1 - 0.9126577402e3 * X(94) * X(14) ^ (-0.5739400206e0) * X(142) ^ 0.1e1;  ERG(95,1) = 0.1187734899e3 * X(89) * X(96) * X(9) ^ (-0.283617331e-1) * X(16) ^ (-0.9966232504e0) * X(126) ^ 0.1e1 - 0.1049201131e2 * X(95) * X(15) ^ 0.685e0 * X(2) ^ (-0.3320804470e-2) * X(5) ^ (-0.2397510852e-1) * X(7) ^ 0.9629101285e0 * X(133) ^ 0.1e1 - 0.1085002492e2 * X(95) * X(15) ^ 0.685e0 * X(2) ^ (-0.3358742751e-2) * X(3) ^ 0.9739478958e0 * X(5) ^ (-0.2424327076e-1) * X(133) ^ 0.1e1 - 0.6250743872e2 * X(95) * X(15) ^ (-0.558993415e-1) * X(128) ^ 0.3344404739e-2 * X(144) ^ 0.1e1 - 0.6574294837e1 * X(95) * X(15) ^ 0.685e0 * X(18) ^ 0.5000000001e0 * X(155) ^ 0.1e1 - 0.1481189310e2 * X(95) * X(15) ^ (-0.1821155943e0) * X(168) ^ 0.1e1;  ERG(96,1) = 0.5720507542e2 * X(146) ^ 0.1e1 * X(147) ^ 0.5008488966e0 - 0.1187734899e3 * X(96) * X(16) ^ (-0.9966232504e0) * X(9) ^ 0.9716382669e0 * X(126) ^ 0.1e1;  ERG(97,1) = 0.2224471846e4 * X(84) * X(4) ^ (-0.395170147e-1) * X(150) ^ 0.1e1 + 0.3610604490e5 * X(86) * X(6) ^ (-0.1706161141e0) * X(150) ^ 0.1e1 - 0.2010896717e1 * X(97) * X(17) ^ (-0.4999999997e0) * X(14) ^ 0.2344299609e0 * X(145) ^ 0.1e1;  ERG(98,1) = 0.6751417734e3 * X(88) * X(8) ^ (-0.5000000000e0) * X(135) ^ 0.1e1 + 0.2587449087e-2 * X(101) * X(21) ^ (-0.5e0) * X(37) ^ 0.5e0 - 0.1525467333e3 * X(98) * X(18) ^ (-0.703517588e-1) * X(164) ^ 0.1e1 - 0.3559423777e3 * X(98) * X(18) ^ (-0.703517588e-1) * X(151) ^ 0.1e1 - 0.6574294837e1 * X(98) * X(18) ^ (-0.4999999999e0) * X(15) ^ 0.1685e1 * X(155) ^ 0.1e1 - 0.1733307912e-1 * X(98) * X(18) ^ (-0.5e0) * X(32) ^ 0.5e0;  ERG(99,1) = 0.6574294837e1 * X(98) * X(95) * X(18) ^ (-0.4999999999e0) * X(15) ^ 0.685e0 * X(155) ^ 0.1e1 + 0.2763393752e-2 * X(102) * X(22) ^ (-0.5e0) * X(37) ^ 0.5e0 - 0.5371916275e4 * X(99) * X(19) ^ (-0.44075706e-2) * X(164) ^ 0.1e1 - 0.8694450414e4 * X(99) * X(19) ^ (-0.44075706e-2) * X(151) ^ 0.1e1 - 0.3517226030e-1 * X(99) * X(19) ^ (-0.5e0) * X(32) ^ 0.5e0;  ERG(100,1) = 0.2030671395e-1 * X(88) * X(8) ^ (-0.5e0) * X(32) ^ 0.5e0 - 0.3031347641e-2 * X(100) * X(20) ^ (-0.5e0) * X(37) ^ 0.5e0;  ERG(101,1) = 0.1733307912e-1 * X(98) * X(18) ^ (-0.5e0) * X(32) ^ 0.5e0 - 0.2587449087e-2 * X(101) * X(21) ^ (-0.5e0) * X(37) ^ 0.5e0;  ERG(102,1) = 0.3517226030e-1 * X(99) * X(19) ^ (-0.5e0) * X(32) ^ 0.5e0 - 0.2763393752e-2 * X(102) * X(22) ^ (-0.5e0) * X(37) ^ 0.5e0;  ERG(103,1) = 0.1683472807e5 * X(92) * X(104) * X(12) ^ (-0.769171e-4) * X(24) ^ (-0.5842660695e0) * X(159) ^ 0.1e1 - 0.5831662556e7 * X(103) * X(23) ^ (-0.4721881198e0) * X(2) ^ 0.9642857143e0 * X(134) ^ 0.1e1 - 0.9014719843e5 * X(103) * X(23) ^ (-0.4721881198e0) * X(5) ^ 0.8000000000e0 * X(134) ^ 0.1e1;  ERG(104,1) = 0.1725637757e2 * X(105) * X(25) ^ (-0.9553978709e0) * X(12) ^ (-0.4577407228e-1) * X(23) ^ (-0.1583280558e0) * X(128) ^ 0.3750000000e0 * X(160) ^ 0.1e1 - 0.1683472807e5 * X(104) * X(24) ^ (-0.5842660695e0) * X(12) ^ 0.9999230829e0 * X(159) ^ 0.1e1 - 0.4768620195e3 * X(104) * X(24) ^ (-0.8681608437e0) * X(25) ^ 0.7910349154e-2 * X(152) ^ 0.1e1;  ERG(105,1) = 0.1226153687e1 * X(185) / X(124) * X(123) ^ 0.1e1 * X(124) ^ 0.7411630560e0 + 0.9234698783e-2 * X(118) * X(38) ^ (-0.9368548879e0) * X(12) ^ (-0.111e0) * X(128) ^ 0.5000000001e0 * X(161) ^ 0.7986577182e0 * X(163) ^ 0.1e1 - 0.1725637757e2 * X(105) * X(25) ^ (-0.9553978709e0) * X(12) ^ (-0.4577407228e-1) * X(23) ^ (-0.1583280558e0) * X(128) ^ 0.3750000000e0 * X(160) ^ 0.1e1 - 0.4768620195e3 * X(105) * X(25) ^ (-0.9920896508e0) * X(24) ^ 0.1318391563e0 * X(152) ^ 0.1e1 - 0.1637319996e2 * X(105) * X(25) ^ (-0.6960000001e0) * X(32) ^ (-0.5000000000e0) * X(171) ^ 0.1e1;  ERG(106,1) = 0.1637319996e2 * X(105) * X(25) ^ (-0.6960000001e0) * X(32) ^ (-0.5000000000e0) * X(171) ^ 0.1e1 - 0.1052459060e5 * X(106) * X(26) ^ (-0.22172951e-2) * X(172) ^ 0.1e1;  ERG(107,1) = 0.1052459060e5 * X(106) * X(26) ^ (-0.22172951e-2) * X(172) ^ 0.1e1 - 0.9642748842e5 * X(107) * X(27) ^ (-0.24330901e-2) * X(173) ^ 0.1e1;  ERG(108,1) = 0.9642748842e5 * X(107) * X(27) ^ (-0.24330901e-2) * X(173) ^ 0.1e1 - 0.2350954134e5 * X(108) * X(28) ^ (-0.5e0) * X(174) ^ 0.1e1 - 0.1837283321e4 * X(108) * X(28) ^ (-0.5000000000e0) * X(179) ^ 0.1e1;  ERG(109,1) = 0.2350954134e5 * X(108) * X(28) ^ (-0.5e0) * X(174) ^ 0.1e1 - 0.7396817205e5 * X(109) * X(29) ^ (-0.403e0) * X(175) ^ 0.1e1;  ERG(110,1) = 0.7396817205e5 * X(109) * X(29) ^ (-0.403e0) * X(175) ^ 0.1e1 + 0.9967272082e1 * X(113) * X(33) ^ (-0.615e0) * X(180) ^ 0.1e1 - 0.8703177136e8 * X(110) * X(30) ^ (-0.338e0) * X(176) ^ 0.1e1 - 0.3543221570e4 * X(110) * X(30) ^ (-0.173e0) * X(12) ^ 0.9998550936e0 * X(181) ^ 0.1e1;  ERG(111,1) = 0.8703177136e8 * X(110) * X(30) ^ (-0.338e0) * X(176) ^ 0.1e1 + 0.2142776130e2 * X(114) * X(34) ^ (-0.615e0) * X(180) ^ 0.1e1 - 0.1242509645e5 * X(111) * X(31) ^ (-0.443e0) * X(177) ^ 0.1e1 - 0.4075356854e4 * X(111) * X(31) ^ (-0.173e0) * X(12) ^ 0.9998550936e0 * X(181) ^ 0.1e1;  ERG(112,1) = 0.1242509645e5 * X(111) * X(31) ^ (-0.443e0) * X(177) ^ 0.1e1 + 0.5563355615e-1 * X(119) + 0.2209706584e-1 * X(117) * X(37) ^ (-0.5e0) * X(20) ^ 0.5e0 * X(21) ^ 0.5e0 * X(22) ^ 0.5e0 + 0.3671477823e2 * X(115) * X(35) ^ (-0.615e0) * X(180) ^ 0.1e1 - 0.2956492539e0 * X(112) - 0.4169102075e4 * X(112) * X(32) ^ (-0.173e0) * X(12) ^ 0.9998550936e0 * X(183) ^ 0.1e1 - 0.1471655572e1 * X(112) * X(32) ^ (-0.5e0) * X(8) ^ 0.5e0 * X(18) ^ 0.5e0 * X(19) ^ 0.5e0 - 0.8610295570e3 * X(112) * X(32) ^ (-0.5000000000e0) * X(186) ^ 0.1e1;  ERG(113,1) = 0.3543221570e4 * X(110) * X(30) ^ (-0.173e0) * X(12) ^ 0.9998550936e0 * X(181) ^ 0.1e1 - 0.9967272082e1 * X(113) * X(33) ^ (-0.615e0) * X(180) ^ 0.1e1;  ERG(114,1) = 0.4075356854e4 * X(111) * X(31) ^ (-0.173e0) * X(12) ^ 0.9998550936e0 * X(181) ^ 0.1e1 - 0.2142776130e2 * X(114) * X(34) ^ (-0.615e0) * X(180) ^ 0.1e1;  ERG(115,1) = 0.4169102075e4 * X(112) * X(32) ^ (-0.173e0) * X(12) ^ 0.9998550936e0 * X(183) ^ 0.1e1 + 0.3192737733e0 * X(120) * X(40) ^ (-0.5e0) - 0.3671477823e2 * X(115) * X(35) ^ (-0.615e0) * X(180) ^ 0.1e1 - 0.1659444132e-1 * X(115);  ERG(116,1) = 0.3142262948e-1 * X(119) + 0.3491403276e-1 * X(117) - 0.6137232322e0 * X(116) - 0.1939652307e0 * X(116) * X(36) ^ 0.23e0 * X(20) ^ 0.123e1 - 0.1803401715e0 * X(116) * X(36) ^ 0.23e0 * X(21) ^ 0.123e1 - 0.3435069860e0 * X(116) * X(36) ^ 0.23e0 * X(22) ^ 0.123e1;  ERG(117,1) = 0.1939652307e0 * X(116) * X(36) ^ 0.23e0 * X(20) ^ 0.123e1 + 0.1803401715e0 * X(116) * X(36) ^ 0.23e0 * X(21) ^ 0.123e1 + 0.3435069860e0 * X(116) * X(36) ^ 0.23e0 * X(22) ^ 0.123e1 + 0.1471655572e1 * X(112) * X(32) ^ (-0.5e0) * X(8) ^ 0.5e0 * X(18) ^ 0.5e0 * X(19) ^ 0.5e0 - 0.2209706584e-1 * X(117) * X(37) ^ (-0.5e0) * X(20) ^ 0.5e0 * X(21) ^ 0.5e0 * X(22) ^ 0.5e0 - 0.3491403276e-1 * X(117);  ERG(118,1) = 0.1397815044e-2 * X(188) / X(125) * X(125) ^ 0.104e1 + 0.7108537236e-2 * (X(185) / X(124)) * X(122) ^ 0.1e1 * X(124) ^ 0.6961178048e0 - 0.9234698783e-2 * X(118) * X(38) ^ (-0.9368548879e0) * X(12) ^ (-0.111e0) * X(128) ^ 0.5000000001e0 * X(161) ^ 0.7986577182e0 * X(163) ^ 0.1e1;  ERG(119,1) = 0.2956492539e0 * X(112) + 0.6137232322e0 * X(116) + 0.5955208746e5 * X(120) * X(40) ^ (-0.615e0) * X(182) ^ 0.1e1 - 0.8705618563e-1 * X(119);  ERG(120,1) = 0.1659444132e-1 * X(115) - 0.3192737733e0 * X(120) * X(40) ^ (-0.5e0) - 0.5955208746e5 * X(120) * X(40) ^ (-0.615e0) * X(182) ^ 0.1e1;    % acetate perturbation ODE:  ERG(121,1) = -rate1*X(121); |
| --- |

**Reference.**

1. MATLAB (2010). 7.10.0.499 ed. Natick, Massachusetts: The MathWorks Inc.
