# Supplementary material for: Mathematical Modeling and Validation of the Ergosterol Pathway in Saccharomyces cerevisiae
Source: PLoS One. 2011 Dec 14;6(12):e28344. doi: 10.1371/journal.pone.0028344 (PMC3237449; doi:10.1371/journal.pone.0028344)
Supplement: Materials S6 — SL-E flux balanced GMA model in Matlab® [49] format. Files used for total mass experiments. (DOC) [file pone.0028344.s025.doc]

**Materials S6. SL-E flux balanced GMA model in Matlab format.** Files were used for total mass experiments. This code was written for MATLAB® [1].

| function Ergo_Balanced_main % Balanced flux model  global SW SWW P PP    s = [32]; % ODE to be presented. Single value must be between 1 and 40 inclusive.  SW = [157]; % Variable(s) to be perturbed.  P = [1]; % Percent of change for each variable(s) to be perturbed.  Time_start = 0; % Starting time  Time_end = 60; % Final time    % SW's tested in the manuscript: X(133), X(134), X(157), X(171), and X(172)  % Percents (P)tested in the work: 0.01, 1, 10, and 12.5  % Empty brackets in SW or P means no change  % Manuscript interval times: [0,60] and [0,120]    ini =[0.5e-2, 0.10e-1, 0.3611111111e-1, 0.1e-2, 0.50e-1, 0.5e-2, 0.52e-1, .102,...  5.4, 8.4, 3, 0.1e-1, 2600, 10.77669903, 16.7, 24.1,...  22, .14, 0.85e-2, .918, 1.26, 0.765e-1, .5, 182.70,...  870, .1, .1, .1, .2847305389, 1.9, 6.4, 9.51,...  3.4, 13.1, 41.13, 4.755, 42.795, 3086, 47.55, 4.57];  depx1 = [1:numel(ini)];  indp = [122:166,168,169,171:177,179:186];    if isempty(s) error('Introduce a time dependent variable'); end    if ismember(s,depx1) || ismember(s,indp)  tspan = [Time_start, Time_end];  [t,x] = ode23tb('Ergo_Balanced', tspan, ini);  cc=[t,x];  dd=[t];  sa= length(dd);  XX = real(cc(sa,s+1)/ini(s));    ['Time interval = [', num2str(Time_start), ',',num2str(Time_end),']']  ['Variable(s) perturbed : ',num2str(SW), ' . Percent(s) of change: ',num2str(P)]  ['Initial X(',num2str(s), ') = ',num2str(ini(s)) ,...  ', Final X(',num2str(s), ') = ',num2str(real(cc(sa,s+1))),...  ', Normalized X(',num2str(s), ') = ',num2str(XX)]    else  error('Not a valid time dependent variable');  end  end  %--------------------------------------------------------------------------    function [ERG] = Ergo_Balanced(t,X)    global SW SWW P PP  SWW = size(SW);  PP = length(P);    X(122) = 45;  X(123) = 0.12e0;  X(124) = 0.227000e3;  X(125) = 1250;  X(126) = 0.266e-2;  X(127) = 0.262e-3;  X(128) = 1100;  X(129) = 0.54e-5;  X(130) = 0.508e-1;  X(131) = 0.13e-2;  X(132) = 0.45e-2;  X(133) = 0.33e-3;  X(134) = 0.165e-4;  X(135) = 0.1650000000e-3;  X(136) = 0.4e-5;  X(137) = 446;  X(138) = 0.332e-2;  X(139) = 0.24e-2;  X(140) = 0.61e-3;  X(141) = 0.8e-3;  X(142) = 0.66e-3;  X(143) = 0.1e-3;  X(144) = 0.172e-2;  X(145) = 0.1e-2;  X(146) = 0.833e-3;  X(147) = 1176;  X(148) = 20;  X(149) = 0.394e-2;  X(150) = 0.367e-4;  X(151) = 0.15e-3;  X(152) = 0.89e-2;  X(153) = 0.198e-4;  X(154) = 0.17e-3;  X(155) = 0.8250000000e-4;  X(156) = 0.1066e-4;  X(157) = 0.106e-3;  X(158) = 0.5e-1;  X(159) = 0.6000000000e-3;  X(160) = 0.22e-1;  X(161) = 60;  X(162) = 0;  X(163) = 0.73e0;  X(164) = 0.15e-3;  X(165) = 0.6e1 / 0.125e3;  X(166) = 4000;  X(168) = 0.5e-4;  X(169) = 0;  X(171) = 0.1400000000e0;  X(172) = 0.5500000000e-2;  X(173) = 0.6000000000e-3;  X(174) = 0.4700000000e-3;  X(175) = 0.1000000000e-3;  X(176) = 0.2625e-7;  X(177) = 0.1000000000e-3;  X(179) = 0.4e-2;  X(180) = 0.1100000000e-3;  X(181) = 0.2540000000e-2;  X(182) = 0.9975e-6;  X(183) = 0.2540000000e-2;  X(184) = 0.5e-1;  X(185) = 0.227000e3;  X(186) = 0.1000000000e-2;    for m= 1:PP  if isempty(SW) || isempty(P)  X(SW) = X(SW);  elseif PP == SWW(2)  X(SW(m)) = X(SW(m))*P(m)/100;  elseif PP ~= SWW(2)  error('Not the same number of variables and perturbations');  end  end    % Flux balance ODE's:    ERG(1,1) = 0.1742970878e6 * X(12) ^ 0.9986438167e0 * X(13) ^ 0.1980849053e0 * X(157) ^ 0.1e1 - 0.6502104832e6 * X(1) ^ 0.9932909297e0 * X(127) ^ 0.1e1;  ERG(2,1) = 0.6502104832e6 * X(1) ^ 0.9932909297e0 * X(127) ^ 0.1e1 + 0.3057424256e4 * X(3) ^ 0.5000000001e0 * X(129) ^ 0.1e1 + 0.1201395274e2 * X(4) ^ 0.9688581315e0 * X(141) ^ 0.1e1 - 0.5832721509e7 * X(2) ^ 0.9642857143e0 * X(23) ^ 0.5278118802e0 * X(134) ^ 0.1e1 - 0.2266068147e4 * X(2) ^ 0.9743589746e0 * X(128) ^ 0.2222222230e-1 * X(136) ^ 0.1e1 - 0.5810000000e4 * X(2) ^ 0.5000000000e0 * X(154) ^ 0.1e1;  ERG(3,1) = 0.5832721509e7 * X(2) ^ 0.9642857143e0 * X(23) ^ 0.5278118802e0 * X(134) ^ 0.1e1 + 0.8910166634e2 * X(8) ^ 0.9722222223e0 * X(164) ^ 0.1e1 + 0.1525467333e3 * X(18) ^ 0.9296482412e0 * X(164) ^ 0.1e1 + 0.5602317528e4 * X(19) ^ 0.9955924294e0 * X(164) ^ 0.1e1 - 0.3057424256e4 * X(3) ^ 0.5000000001e0 * X(129) ^ 0.1e1 - 0.2409715836e5 * X(3) ^ 0.4999999998e0 * X(154) ^ 0.1e1 - 0.1085002492e2 * X(2) ^ (-0.3358742751e-2) * X(3) ^ 0.9739478958e0 * X(5) ^ (-0.2424327076e-1) * X(15) ^ 0.1685e1 * X(133) ^ 0.1e1;  ERG(4,1) = 0.2266068147e4 * X(2) ^ 0.9743589746e0 * X(128) ^ 0.2222222230e-1 * X(136) ^ 0.1e1 - 0.1201395274e2 * X(4) ^ 0.9688581315e0 * X(141) ^ 0.1e1 - 0.2224471846e4 * X(4) ^ 0.9604829853e0 * X(150) ^ 0.1e1;  ERG(5,1) = 0.5810000000e4 * X(2) ^ 0.5000000000e0 * X(154) ^ 0.1e1 + 0.2182238068e3 * X(6) ^ 0.8615384613e0 * X(141) ^ 0.1e1 + 0.2547853547e4 * X(7) ^ 0.5000000002e0 * X(153) ^ 0.1e1 - 0.9014719843e5 * X(5) ^ 0.8000000000e0 * X(23) ^ 0.5278118802e0 * X(134) ^ 0.1e1 - 0.6900945118e5 * X(5) ^ 0.9600000000e0 * X(128) ^ 0.2222222230e-1 * X(136) ^ 0.1e1;  ERG(6,1) = 0.6900945118e5 * X(5) ^ 0.9600000000e0 * X(128) ^ 0.2222222230e-1 * X(136) ^ 0.1e1 - 0.2182238068e3 * X(6) ^ 0.8615384613e0 * X(141) ^ 0.1e1 - 0.3610604490e5 * X(6) ^ 0.8293838859e0 * X(150) ^ 0.1e1;  ERG(7,1) = 0.9014719843e5 * X(5) ^ 0.8000000000e0 * X(23) ^ 0.5278118802e0 * X(134) ^ 0.1e1 + 0.2970055545e3 * X(8) ^ 0.9722222223e0 * X(151) ^ 0.1e1 + 0.2409715836e5 * X(3) ^ 0.4999999998e0 * X(154) ^ 0.1e1 + 0.3559423777e3 * X(18) ^ 0.9296482412e0 * X(151) ^ 0.1e1 + 0.1307207423e5 * X(19) ^ 0.9955924294e0 * X(151) ^ 0.1e1 - 0.2547853547e4 * X(7) ^ 0.5000000002e0 * X(153) ^ 0.1e1 - 0.1049201131e2 * X(2) ^ (-0.3320804470e-2) * X(5) ^ (-0.2397510852e-1) * X(7) ^ 0.9629101285e0 * X(15) ^ 0.1685e1 * X(133) ^ 0.1e1 - 0.3798231192e5 * X(7) ^ 0.4999999997e0 * X(143) ^ 0.1e1;  ERG(8,1) = 0.1049201131e2 * X(2) ^ (-0.3320804470e-2) * X(5) ^ (-0.2397510852e-1) * X(7) ^ 0.9629101285e0 * X(15) ^ 0.1685e1 * X(133) ^ 0.1e1 + 0.1085002492e2 * X(2) ^ (-0.3358742751e-2) * X(3) ^ 0.9739478958e0 * X(5) ^ (-0.2424327076e-1) * X(15) ^ 0.1685e1 * X(133) ^ 0.1e1 + 0.3190892254e-2 * X(20) ^ 0.5e0 * X(37) ^ 0.5e0 - 0.2970055545e3 * X(8) ^ 0.9722222223e0 * X(151) ^ 0.1e1 - 0.6941182673e3 * X(8) ^ 0.5000000000e0 * X(135) ^ 0.1e1 - 0.8910166634e2 * X(8) ^ 0.9722222223e0 * X(164) ^ 0.1e1 - 0.2030671395e-1 * X(8) ^ 0.5e0 * X(32) ^ 0.5e0;  ERG(9,1) = 0.2513279808e4 * X(11) ^ 0.9940357859e0 * X(140) ^ 0.1e1 - 0.8931564995e3 * X(2) ^ (-0.6568638313e-2) * X(5) ^ (-0.1305154574e-1) * X(9) ^ 0.9933310569e0 * X(11) ^ 0.235e0 * X(13) ^ 0.2063074440e-3 * X(14) ^ (-0.5910831328e0) * X(15) ^ 0.88e-1 * X(16) ^ (-0.2704826039e0) * X(138) ^ 0.1e1 - 0.1188457386e3 * X(9) ^ 0.9716382669e0 * X(16) ^ 0.3376749592e-2 * X(126) ^ 0.1e1;  ERG(10,1) = 0.8931564995e3 * X(2) ^ (-0.6568638313e-2) * X(5) ^ (-0.1305154574e-1) * X(9) ^ 0.9933310569e0 * X(11) ^ 0.235e0 * X(13) ^ 0.2063074440e-3 * X(14) ^ (-0.5910831328e0) * X(15) ^ 0.88e-1 * X(16) ^ (-0.2704826039e0) * X(138) ^ 0.1e1 - 0.9465130187e5 * X(10) ^ 0.5000000001e0 * X(156) ^ 0.1e1;  ERG(11,1) = 0.1141221528e6 * X(10) ^ 0.1493e0 * X(12) ^ 0.1e1 * X(149) ^ 0.1e1 - 0.9455645805e2 * X(2) ^ (-0.2071563088e-1) * X(5) ^ (-0.5022831050e-1) * X(9) ^ 0.326e0 * X(11) ^ 0.4230769231e0 * X(15) ^ 0.248e0 * X(139) ^ 0.1e1 - 0.2513279808e4 * X(11) ^ 0.9940357859e0 * X(140) ^ 0.1e1;  ERG(12,1) = 0.2306702893e5 * X(130) ^ 0.1e1 * X(158) ^ 0.9975062347e0 + 0.4768620195e3 * X(24) ^ 0.1318391563e0 * X(25) ^ 0.7910349154e-2 * X(152) ^ 0.1e1 + 0.2224471846e4 * X(4) ^ 0.9604829853e0 * X(150) ^ 0.1e1 + 0.3610604490e5 * X(6) ^ 0.8293838859e0 * X(150) ^ 0.1e1 + 0.8690384668e3 * X(33) ^ 0.385e0 * X(180) ^ 0.1e1 + 0.1623520875e4 * X(34) ^ 0.385e0 * X(180) ^ 0.1e1 + 0.1483465863e4 * X(35) ^ 0.385e0 * X(180) ^ 0.1e1 - 0.1742970878e6 * X(12) ^ 0.9986438167e0 * X(13) ^ 0.1980849053e0 * X(157) ^ 0.1e1 - 0.1141221528e6 * X(10) ^ 0.1493e0 * X(12) ^ 0.1e1 * X(149) ^ 0.1e1 - 0.3000052502e3 * X(12) ^ 0.1e1 * X(148) ^ 0.1e1 - 0.1683745902e5 * X(12) ^ 0.9999230829e0 * X(24) ^ 0.4157339305e0 * X(159) ^ 0.1e1 - 0.3543221570e4 * X(12) ^ 0.9998550936e0 * X(30) ^ 0.827e0 * X(181) ^ 0.1e1 - 0.4075356854e4 * X(12) ^ 0.9998550936e0 * X(31) ^ 0.827e0 * X(181) ^ 0.1e1 - 0.4169102075e4 * X(12) ^ 0.9998550936e0 * X(32) ^ 0.827e0 * X(183) ^ 0.1e1;  ERG(13,1) = 0.3511209420e3 * X(131) ^ 0.1e1 * X(137) ^ 0.1663551401e0 + 0.8017109569e3 * X(165) ^ 0.1e1 * X(166) ^ 0.3984637534e-1 - 0.8931564995e3 * X(2) ^ (-0.6568638313e-2) * X(5) ^ (-0.1305154574e-1) * X(9) ^ 0.9933310569e0 * X(11) ^ 0.235e0 * X(13) ^ 0.2063074440e-3 * X(14) ^ (-0.5910831328e0) * X(15) ^ 0.88e-1 * X(16) ^ (-0.2704826039e0) * X(138) ^ 0.1e1 - 0.1742970878e6 * X(12) ^ 0.9986438167e0 * X(13) ^ 0.1980849053e0 * X(157) ^ 0.1e1 - 0.2351870680e4 * X(13) ^ 0.1999999997e0 * X(132) ^ 0.1e1;  ERG(14,1) = 0.9455645805e2 * X(2) ^ (-0.2071563088e-1) * X(5) ^ (-0.5022831050e-1) * X(9) ^ 0.326e0 * X(11) ^ 0.4230769231e0 * X(15) ^ 0.248e0 * X(139) ^ 0.1e1 + 0.1049201131e2 * X(2) ^ (-0.3320804470e-2) * X(5) ^ (-0.2397510852e-1) * X(7) ^ 0.9629101285e0 * X(15) ^ 0.1685e1 * X(133) ^ 0.1e1 + 0.1085002492e2 * X(2) ^ (-0.3358742751e-2) * X(3) ^ 0.9739478958e0 * X(5) ^ (-0.2424327076e-1) * X(15) ^ 0.1685e1 * X(133) ^ 0.1e1 + 0.6856266054e1 * X(15) ^ 0.1685e1 * X(18) ^ 0.5000000001e0 * X(155) ^ 0.1e1 - 0.2010896717e1 * X(14) ^ 0.2344299609e0 * X(17) ^ 0.5000000003e0 * X(145) ^ 0.1e1 - 0.9132079842e3 * X(14) ^ 0.4260599794e0 * X(142) ^ 0.1e1;  ERG(15,1) = 0.1188457386e3 * X(9) ^ 0.9716382669e0 * X(16) ^ 0.3376749592e-2 * X(126) ^ 0.1e1 - 0.1049201131e2 * X(2) ^ (-0.3320804470e-2) * X(5) ^ (-0.2397510852e-1) * X(7) ^ 0.9629101285e0 * X(15) ^ 0.1685e1 * X(133) ^ 0.1e1 - 0.1085002492e2 * X(2) ^ (-0.3358742751e-2) * X(3) ^ 0.9739478958e0 * X(5) ^ (-0.2424327076e-1) * X(15) ^ 0.1685e1 * X(133) ^ 0.1e1 - 0.6250743872e2 * X(15) ^ 0.9441006585e0 * X(128) ^ 0.3344404739e-2 * X(144) ^ 0.1e1 - 0.6856266054e1 * X(15) ^ 0.1685e1 * X(18) ^ 0.5000000001e0 * X(155) ^ 0.1e1 - 0.1481189310e2 * X(15) ^ 0.8178844057e0 * X(168) ^ 0.1e1;  ERG(16,1) = 0.5723987266e2 * X(146) ^ 0.1e1 * X(147) ^ 0.5008488966e0 - 0.1188457386e3 * X(9) ^ 0.9716382669e0 * X(16) ^ 0.3376749592e-2 * X(126) ^ 0.1e1;  ERG(17,1) = 0.2224471846e4 * X(4) ^ 0.9604829853e0 * X(150) ^ 0.1e1 + 0.3610604490e5 * X(6) ^ 0.8293838859e0 * X(150) ^ 0.1e1 - 0.2010896717e1 * X(14) ^ 0.2344299609e0 * X(17) ^ 0.5000000003e0 * X(145) ^ 0.1e1;  ERG(18,1) = 0.6941182673e3 * X(8) ^ 0.5000000000e0 * X(135) ^ 0.1e1 + 0.2723630619e-2 * X(21) ^ 0.5e0 * X(37) ^ 0.5e0 - 0.1525467333e3 * X(18) ^ 0.9296482412e0 * X(164) ^ 0.1e1 - 0.3559423777e3 * X(18) ^ 0.9296482412e0 * X(151) ^ 0.1e1 - 0.6856266054e1 * X(15) ^ 0.1685e1 * X(18) ^ 0.5000000001e0 * X(155) ^ 0.1e1 - 0.1733307912e-1 * X(18) ^ 0.5e0 * X(32) ^ 0.5e0;  ERG(19,1) = 0.6856266054e1 * X(15) ^ 0.1685e1 * X(18) ^ 0.5000000001e0 * X(155) ^ 0.1e1 + 0.5526787506e-2 * X(22) ^ 0.5e0 * X(37) ^ 0.5e0 - 0.5602317528e4 * X(19) ^ 0.9955924294e0 * X(164) ^ 0.1e1 - 0.1307207423e5 * X(19) ^ 0.9955924294e0 * X(151) ^ 0.1e1 - 0.3517226030e-1 * X(19) ^ 0.5e0 * X(32) ^ 0.5e0;  ERG(20,1) = 0.2030671395e-1 * X(8) ^ 0.5e0 * X(32) ^ 0.5e0 - 0.3190892254e-2 * X(20) ^ 0.5e0 * X(37) ^ 0.5e0;  ERG(21,1) = 0.1733307912e-1 * X(18) ^ 0.5e0 * X(32) ^ 0.5e0 - 0.2723630619e-2 * X(21) ^ 0.5e0 * X(37) ^ 0.5e0;  ERG(22,1) = 0.3517226030e-1 * X(19) ^ 0.5e0 * X(32) ^ 0.5e0 - 0.5526787506e-2 * X(22) ^ 0.5e0 * X(37) ^ 0.5e0;  ERG(23,1) = 0.1683745902e5 * X(12) ^ 0.9999230829e0 * X(24) ^ 0.4157339305e0 * X(159) ^ 0.1e1 - 0.5832721509e7 * X(2) ^ 0.9642857143e0 * X(23) ^ 0.5278118802e0 * X(134) ^ 0.1e1 - 0.9014719843e5 * X(5) ^ 0.8000000000e0 * X(23) ^ 0.5278118802e0 * X(134) ^ 0.1e1;  ERG(24,1) = 0.1725662971e2 * X(12) ^ (-0.4577407228e-1) * X(23) ^ (-0.1583280558e0) * X(25) ^ 0.4460212912e-1 * X(128) ^ 0.3750000000e0 * X(160) ^ 0.1e1 - 0.1683745902e5 * X(12) ^ 0.9999230829e0 * X(24) ^ 0.4157339305e0 * X(159) ^ 0.1e1 - 0.4768620195e3 * X(24) ^ 0.1318391563e0 * X(25) ^ 0.7910349154e-2 * X(152) ^ 0.1e1;  ERG(25,1) = 0.1232059507e1 * X(123) ^ 0.1e1 * X(124) ^ 0.7411630560e0 + 0.9272832387e-2 * X(12) ^ (-0.111e0) * X(38) ^ 0.6314511210e-1 * X(128) ^ 0.5000000001e0 * X(161) ^ 0.7986577182e0 * X(163) ^ 0.1e1 - 0.1725662971e2 * X(12) ^ (-0.4577407228e-1) * X(23) ^ (-0.1583280558e0) * X(25) ^ 0.4460212912e-1 * X(128) ^ 0.3750000000e0 * X(160) ^ 0.1e1 - 0.4768620195e3 * X(24) ^ 0.1318391563e0 * X(25) ^ 0.7910349154e-2 * X(152) ^ 0.1e1 - 0.1667327233e2 * X(25) ^ 0.3039999999e0 * X(32) ^ (-0.5000000000e0) * X(171) ^ 0.1e1;  ERG(26,1) = 0.1667327233e2 * X(25) ^ 0.3039999999e0 * X(32) ^ (-0.5000000000e0) * X(171) ^ 0.1e1 - 0.1071747524e5 * X(26) ^ 0.9977827049e0 * X(172) ^ 0.1e1;  ERG(27,1) = 0.1071747524e5 * X(26) ^ 0.9977827049e0 * X(172) ^ 0.1e1 - 0.9819471929e5 * X(27) ^ 0.9975669099e0 * X(173) ^ 0.1e1;  ERG(28,1) = 0.9819471929e5 * X(27) ^ 0.9975669099e0 * X(173) ^ 0.1e1 - 0.2422697172e5 * X(28) ^ 0.5e0 * X(174) ^ 0.1e1 - 0.1837283321e4 * X(28) ^ 0.5000000000e0 * X(179) ^ 0.1e1;  ERG(29,1) = 0.2422697172e5 * X(28) ^ 0.5e0 * X(174) ^ 0.1e1 - 0.7622542641e5 * X(29) ^ 0.597e0 * X(175) ^ 0.1e1;  ERG(30,1) = 0.7622542641e5 * X(29) ^ 0.597e0 * X(175) ^ 0.1e1 + 0.8690384668e3 * X(33) ^ 0.385e0 * X(180) ^ 0.1e1 - 0.8968768184e8 * X(30) ^ 0.662e0 * X(176) ^ 0.1e1 - 0.3543221570e4 * X(12) ^ 0.9998550936e0 * X(30) ^ 0.827e0 * X(181) ^ 0.1e1;  ERG(31,1) = 0.8968768184e8 * X(30) ^ 0.662e0 * X(176) ^ 0.1e1 + 0.1623520875e4 * X(34) ^ 0.385e0 * X(180) ^ 0.1e1 - 0.1280426769e5 * X(31) ^ 0.557e0 * X(177) ^ 0.1e1 - 0.4075356854e4 * X(12) ^ 0.9998550936e0 * X(31) ^ 0.827e0 * X(181) ^ 0.1e1;  ERG(32,1) = 0.1280426769e5 * X(31) ^ 0.557e0 * X(177) ^ 0.1e1 + 0.6137232320e-1 * X(39) + 0.2569426260e-1 * X(20) ^ 0.5e0 * X(21) ^ 0.5e0 * X(22) ^ 0.5e0 * X(37) ^ 0.5e0 + 0.1483465863e4 * X(35) ^ 0.385e0 * X(180) ^ 0.1e1 - 0.3068616160e0 * X(32) - 0.4169102075e4 * X(12) ^ 0.9998550936e0 * X(32) ^ 0.827e0 * X(183) ^ 0.1e1 - 0.1471655572e1 * X(8) ^ 0.5e0 * X(18) ^ 0.5e0 * X(19) ^ 0.5e0 * X(32) ^ 0.5e0 - 0.1167633998e4 * X(32) ^ 0.5000000000e0 * X(186) ^ 0.1e1;  ERG(33,1) = 0.3543221570e4 * X(12) ^ 0.9998550936e0 * X(30) ^ 0.827e0 * X(181) ^ 0.1e1 - 0.8690384668e3 * X(33) ^ 0.385e0 * X(180) ^ 0.1e1;  ERG(34,1) = 0.4075356854e4 * X(12) ^ 0.9998550936e0 * X(31) ^ 0.827e0 * X(181) ^ 0.1e1 - 0.1623520875e4 * X(34) ^ 0.385e0 * X(180) ^ 0.1e1;  ERG(35,1) = 0.4169102075e4 * X(12) ^ 0.9998550936e0 * X(32) ^ 0.827e0 * X(183) ^ 0.1e1 + 0.3192737733e0 * X(40) ^ 0.5e0 - 0.1483465863e4 * X(35) ^ 0.385e0 * X(180) ^ 0.1e1 - 0.1659444132e-1 * X(35);  ERG(36,1) = 0.6137232320e-1 * X(39) + 0.6819147022e-1 * X(37) - 0.6137232320e0 * X(36) - 0.1939652307e0 * X(20) ^ 0.123e1 * X(36) ^ 0.123e1 - 0.1803401715e0 * X(21) ^ 0.123e1 * X(36) ^ 0.123e1 - 0.3435069860e0 * X(22) ^ 0.123e1 * X(36) ^ 0.123e1;  ERG(37,1) = 0.1939652307e0 * X(20) ^ 0.123e1 * X(36) ^ 0.123e1 + 0.1803401715e0 * X(21) ^ 0.123e1 * X(36) ^ 0.123e1 + 0.3435069860e0 * X(22) ^ 0.123e1 * X(36) ^ 0.123e1 + 0.1471655572e1 * X(8) ^ 0.5e0 * X(18) ^ 0.5e0 * X(19) ^ 0.5e0 * X(32) ^ 0.5e0 - 0.2569426260e-1 * X(20) ^ 0.5e0 * X(21) ^ 0.5e0 * X(22) ^ 0.5e0 * X(37) ^ 0.5e0 - 0.6819147022e-1 * X(37);  ERG(38,1) = 0.1397815044e-2 * X(125) ^ 0.104e1 + 0.7142775800e-2 * X(122) ^ 0.1e1 * X(124) ^ 0.6961178048e0 - 0.9272832387e-2 * X(12) ^ (-0.111e0) * X(38) ^ 0.6314511210e-1 * X(128) ^ 0.5000000001e0 * X(161) ^ 0.7986577182e0 * X(163) ^ 0.1e1;  ERG(39,1) = 0.3068616160e0 * X(32) + 0.6137232320e0 * X(36) - 0.1227446464e0 * X(39);  ERG(40,1) = 0.1659444132e-1 * X(35) - 0.3192737733e0 * X(40) ^ 0.5e0;    %% Flux balance, flux agregation system:    % ERG(1,1) = 0.1742970878e6 * X(12) ^ 0.9986438167e0 * X(13) ^ 0.1980849053e0 * X(157) ^ 0.1e1 - 0.6502104832e6 * X(1) ^ 0.9932909297e0 * X(127) ^ 0.1e1;  % ERG(2,1) = 0.6531802624e6 * X(1) ^ 0.9897592360e0 * X(127) ^ 0.9964444519e0 * X(3) ^ 0.1771046463e-2 * X(129) ^ 0.3542092927e-2 * X(4) ^ 0.1303622234e-4 * X(141) ^ 0.1345524377e-4 - 0.3829311911e7 * X(2) ^ 0.9125144151e0 * X(23) ^ 0.4688843834e0 * X(134) ^ 0.8883551147e0 * X(128) ^ 0.2990054186e-5 * X(136) ^ 0.1345524379e-3 * X(154) ^ 0.1115103329e0;  % ERG(3,1) = 0.5617459784e7 * X(2) ^ 0.9492947279e0 * X(23) ^ 0.5196064069e0 * X(134) ^ 0.9844537918e0 * X(8) ^ 0.1766771855e-2 * X(164) ^ 0.1554620810e-1 * X(18) ^ 0.4278692153e-2 * X(19) ^ 0.9086246408e-2 - 0.2299447239e5 * X(3) ^ 0.5104888376e0 * X(129) ^ 0.3925262269e-2 * X(154) ^ 0.9739439554e0 * X(2) ^ (-0.7433160486e-4) * X(5) ^ (-0.5365225491e-3) * X(15) ^ 0.3729036829e-1 * X(133) ^ 0.2213078237e-1;  % ERG(4,1) = 0.2266068147e4 * X(2) ^ 0.9743589746e0 * X(128) ^ 0.2222222230e-1 * X(136) ^ 0.1e1 - 0.1826646699e4 * X(4) ^ 0.9613204999e0 * X(141) ^ 0.9999999983e-1 * X(150) ^ 0.9000000002e0;  % ERG(5,1) = 0.7642582854e4 * X(2) ^ 0.4405768391e0 * X(154) ^ 0.8811536782e0 * X(6) ^ 0.1397228859e-1 * X(141) ^ 0.1621783498e-1 * X(7) ^ 0.5131424363e-1 * X(153) ^ 0.1026284872e0 - 0.1344744534e6 * X(5) ^ 0.8259485360e0 * X(23) ^ 0.4422122202e0 * X(134) ^ 0.8378216499e0 * X(128) ^ 0.3603963349e-2 * X(136) ^ 0.1621783501e0;  % ERG(6,1) = 0.6900945118e5 * X(5) ^ 0.9600000000e0 * X(128) ^ 0.2222222230e-1 * X(136) ^ 0.1e1 - 0.2998429767e5 * X(6) ^ 0.8325993433e0 * X(141) ^ 0.9999999978e-1 * X(150) ^ 0.9000000002e0;  % ERG(7,1) = 0.4271418630e5 * X(5) ^ 0.8321761351e-1 * X(23) ^ 0.5490405631e-1 * X(134) ^ 0.1040220169e0 * X(8) ^ 0.5213876334e-2 * X(151) ^ 0.3372347203e-1 * X(3) ^ 0.4311272553e0 * X(154) ^ 0.8622545111e0 * X(18) ^ 0.8838719152e-2 * X(19) ^ 0.1876993653e-1 - 0.3549370723e5 * X(7) ^ 0.5129124149e0 * X(153) ^ 0.1274211788e-1 * X(2) ^ (-0.9263052042e-4) * X(5) ^ (-0.6687616809e-3) * X(15) ^ 0.4700139028e-1 * X(133) ^ 0.2789400016e-1 * X(143) ^ 0.9593638820e0;  % ERG(8,1) = 0.2390774391e1 * X(2) ^ (-0.2275107674e-2) * X(5) ^ (-0.1642390914e-1) * X(7) ^ 0.3856907801e0 * X(15) ^ 0.1148989990e1 * X(133) ^ 0.6818931692e0 * X(3) ^ 0.2740164924e0 * X(20) ^ 0.1590534154e0 * X(37) ^ 0.1590534154e0 - 0.5872968738e2 * X(8) ^ 0.5472745777e0 * X(151) ^ 0.7700836195e-1 * X(135) ^ 0.5817822986e0 * X(164) ^ 0.2310250858e-1 * X(32) ^ 0.1590534154e0;  % ERG(9,1) = 0.2513279808e4 * X(11) ^ 0.9940357859e0 * X(140) ^ 0.1e1 - 0.8305409732e3 * X(2) ^ (-0.4203901294e-2) * X(5) ^ (-0.8352935176e-2) * X(9) ^ 0.9855215626e0 * X(11) ^ 0.1503990259e0 * X(13) ^ 0.1320359090e-3 * X(14) ^ (-0.3782907550e0) * X(15) ^ 0.5631963525e-1 * X(16) ^ (-0.1718921014e0) * X(138) ^ 0.6399958550e0 * X(126) ^ 0.3600041450e0;  % ERG(10,1) = 0.8931564995e3 * X(2) ^ (-0.6568638313e-2) * X(5) ^ (-0.1305154574e-1) * X(9) ^ 0.9933310569e0 * X(11) ^ 0.235e0 * X(13) ^ 0.2063074440e-3 * X(14) ^ (-0.5910831328e0) * X(15) ^ 0.88e-1 * X(16) ^ (-0.2704826039e0) * X(138) ^ 0.1e1 - 0.9465130187e5 * X(10) ^ 0.5000000001e0 * X(156) ^ 0.1e1;  % ERG(11,1) = 0.1141221528e6 * X(10) ^ 0.1493e0 * X(12) ^ 0.1e1 * X(149) ^ 0.1e1 - 0.1898129087e4 * X(2) ^ (-0.5394762997e-2) * X(5) ^ (-0.1308045275e-1) * X(9) ^ 0.8489689487e-1 * X(11) ^ 0.8453467230e0 * X(15) ^ 0.6458414088e-1 * X(139) ^ 0.2604199229e0 * X(140) ^ 0.7395800771e0;  % ERG(12,1) = 0.2182150936e5 * X(130) ^ 0.8523009695e0 * X(158) ^ 0.8501755308e0 * X(24) ^ 0.1693513343e-1 * X(25) ^ 0.1016107977e-2 * X(152) ^ 0.1284529870e0 * X(4) ^ 0.1487499539e-5 * X(150) ^ 0.2377769997e-3 * X(6) ^ 0.1959239455e-3 * X(33) ^ 0.8512065171e-3 * X(180) ^ 0.1900826665e-1 * X(34) ^ 0.2672907212e-2 * X(35) ^ 0.3794068930e-2 - 0.1053812860e4 * X(12) ^ 0.9999789851e0 * X(13) ^ 0.2524280191e-2 * X(157) ^ 0.1274342528e-1 * X(10) ^ 0.1331815376e-1 * X(149) ^ 0.8920397698e-1 * X(148) ^ 0.8663272854e0 * X(24) ^ 0.5286907422e-2 * X(159) ^ 0.1271704577e-1 * X(30) ^ 0.1828435817e-2 * X(181) ^ 0.9153542154e-2 * X(31) ^ 0.5741543545e-2 * X(32) ^ 0.8149857155e-2 * X(183) ^ 0.9854724491e-2;  % ERG(13,1) = 0.8775905845e3 * X(131) ^ 0.2297461868e-1 * X(137) ^ 0.3821945910e-2 * X(165) ^ 0.9770253814e0 * X(166) ^ 0.3893092007e-1 - 0.3198603074e4 * X(2) ^ (-0.3504423176e-3) * X(5) ^ (-0.6963108210e-3) * X(9) ^ 0.5299503810e-1 * X(11) ^ 0.1253744546e-1 * X(13) ^ 0.1893100031e0 * X(14) ^ (-0.3153477678e-1) * X(15) ^ 0.4694873195e-2 * X(16) ^ (-0.1443047189e-1) * X(138) ^ 0.5335083175e-1 * X(12) ^ 0.1608019856e-1 * X(157) ^ 0.1610203587e-1 * X(132) ^ 0.9305471324e0;  % ERG(14,1) = 0.1063819822e3 * X(2) ^ (-0.1997057872e-1) * X(5) ^ (-0.4883096331e-1) * X(9) ^ 0.3129321827e0 * X(11) ^ 0.4061177453e0 * X(15) ^ 0.3056026183e0 * X(139) ^ 0.9599146708e0 * X(7) ^ 0.1446750384e-1 * X(133) ^ 0.2557824183e-1 * X(3) ^ 0.1027853104e-1 * X(18) ^ 0.7253543750e-2 * X(155) ^ 0.1450708750e-1 - 0.9087281507e3 * X(14) ^ 0.4241771646e0 * X(17) ^ 0.4912629974e-2 * X(145) ^ 0.9825259942e-2 * X(142) ^ 0.9901747398e0;  % ERG(15,1) = 0.1188457386e3 * X(9) ^ 0.9716382669e0 * X(16) ^ 0.3376749592e-2 * X(126) ^ 0.1e1 - 0.7323179473e2 * X(2) ^ (-0.8695712578e-4) * X(5) ^ (-0.6277399306e-3) * X(7) ^ 0.1474152721e-1 * X(15) ^ 0.9737940669e0 * X(133) ^ 0.2606270937e-1 * X(3) ^ 0.1047321270e-1 * X(128) ^ 0.3192745305e-2 * X(144) ^ 0.9546527871e0 * X(18) ^ 0.7390930304e-2 * X(155) ^ 0.1478186061e-1 * X(168) ^ 0.4502643142e-2;  % ERG(16,1) = 0.5723987266e2 * X(146) ^ 0.1e1 * X(147) ^ 0.5008488966e0 - 0.1188457386e3 * X(9) ^ 0.9716382669e0 * X(16) ^ 0.3376749592e-2 * X(126) ^ 0.1e1;  % ERG(17,1) = 0.3687697572e5 * X(4) ^ 0.6255859654e-2 * X(150) ^ 0.9999999999e0 * X(6) ^ 0.8239819064e0 - 0.2010896717e1 * X(14) ^ 0.2344299609e0 * X(17) ^ 0.5000000003e0 * X(145) ^ 0.1e1;  % ERG(18,1) = 0.1630970666e2 * X(8) ^ 0.3232522094e0 * X(135) ^ 0.6465044189e0 * X(21) ^ 0.1767477906e0 * X(37) ^ 0.1767477906e0 - 0.6088553476e1 * X(18) ^ 0.5931192306e0 * X(164) ^ 0.6502009427e-1 * X(151) ^ 0.1517135533e0 * X(15) ^ 0.7241637496e0 * X(155) ^ 0.4297707713e0 * X(32) ^ 0.1767477906e0;  % ERG(19,1) = 0.1572623650e1 * X(15) ^ 0.1193967992e1 * X(18) ^ 0.3542931728e0 * X(155) ^ 0.7085863456e0 * X(22) ^ 0.1457068272e0 * X(37) ^ 0.1457068272e0 - 0.7327378009e3 * X(19) ^ 0.8511700286e0 * X(164) ^ 0.2125759037e0 * X(151) ^ 0.4960104420e0 * X(32) ^ 0.1457068272e0;  % ERG(20,1) = 0.2030671395e-1 * X(8) ^ 0.5e0 * X(32) ^ 0.5e0 - 0.3190892254e-2 * X(20) ^ 0.5e0 * X(37) ^ 0.5e0;  % ERG(21,1) = 0.1733307912e-1 * X(18) ^ 0.5e0 * X(32) ^ 0.5e0 - 0.2723630619e-2 * X(21) ^ 0.5e0 * X(37) ^ 0.5e0;  % ERG(22,1) = 0.3517226030e-1 * X(19) ^ 0.5e0 * X(32) ^ 0.5e0 - 0.5526787506e-2 * X(22) ^ 0.5e0 * X(37) ^ 0.5e0;  % ERG(23,1) = 0.1683745902e5 * X(12) ^ 0.9999230829e0 * X(24) ^ 0.4157339305e0 * X(159) ^ 0.1e1 - 0.5250263451e7 * X(2) ^ 0.8614680782e0 * X(23) ^ 0.5278118802e0 * X(134) ^ 0.1000000000e1 * X(5) ^ 0.8530055734e-1;  % ERG(24,1) = 0.1725662971e2 * X(12) ^ (-0.4577407228e-1) * X(23) ^ (-0.1583280558e0) * X(25) ^ 0.4460212912e-1 * X(128) ^ 0.3750000000e0 * X(160) ^ 0.1e1 - 0.8898260236e3 * X(12) ^ 0.9007625317e-1 * X(24) ^ 0.1574133010e0 * X(159) ^ 0.9008318211e-1 * X(25) ^ 0.7197759730e-2 * X(152) ^ 0.9099168178e0;  % ERG(25,1) = 0.9028113010e-1 * X(123) ^ 0.3350435840e0 * X(124) ^ 0.2483219266e0 * X(12) ^ (-0.7381016215e-1) * X(38) ^ 0.4198874743e-1 * X(128) ^ 0.3324782080e0 * X(161) ^ 0.5310725739e0 * X(163) ^ 0.6649564158e0 - 0.1669543092e3 * X(12) ^ (-0.1819399100e-1) * X(23) ^ (-0.6293124202e-1) * X(25) ^ 0.9381000233e-1 * X(128) ^ 0.1490526467e0 * X(160) ^ 0.3974737245e0 * X(24) ^ 0.4768200749e-1 * X(152) ^ 0.3616680266e0 * X(32) ^ (-0.1204291244e0) * X(171) ^ 0.2408582488e0;  % ERG(26,1) = 0.1667327233e2 * X(25) ^ 0.3039999999e0 * X(32) ^ (-0.5000000000e0) * X(171) ^ 0.1e1 - 0.1071747524e5 * X(26) ^ 0.9977827049e0 * X(172) ^ 0.1e1;  % ERG(27,1) = 0.1071747524e5 * X(26) ^ 0.9977827049e0 * X(172) ^ 0.1e1 - 0.9819471929e5 * X(27) ^ 0.9975669099e0 * X(173) ^ 0.1e1;  % ERG(28,1) = 0.9819471929e5 * X(27) ^ 0.9975669099e0 * X(173) ^ 0.1e1 - 0.1721061472e5 * X(28) ^ 0.5000000001e0 * X(174) ^ 0.6077493696e0 * X(179) ^ 0.3922506304e0;  % ERG(29,1) = 0.2422697172e5 * X(28) ^ 0.5e0 * X(174) ^ 0.1e1 - 0.7622542641e5 * X(29) ^ 0.597e0 * X(175) ^ 0.1e1;  % ERG(30,1) = 0.7531227415e5 * X(29) ^ 0.5726476131e0 * X(175) ^ 0.9592087324e0 * X(33) ^ 0.1570463804e-1 * X(180) ^ 0.4079126765e-1 - 0.7033006492e8 * X(30) ^ 0.6687305590e0 * X(176) ^ 0.9592087324e0 * X(12) ^ 0.4078535674e-1 * X(181) ^ 0.4079126765e-1;  % ERG(31,1) = 0.3560489456e8 * X(30) ^ 0.5840124738e0 * X(176) ^ 0.8821940693e0 * X(34) ^ 0.4535528330e-1 * X(180) ^ 0.1178059307e0 - 0.1607784867e5 * X(31) ^ 0.5888076013e0 * X(177) ^ 0.8821940693e0 * X(12) ^ 0.1177888599e0 * X(181) ^ 0.1178059307e0;  % ERG(32,1) = 0.1823209797e3 * X(31) ^ 0.2765797253e0 * X(177) ^ 0.4965524691e0 * X(39) ^ 0.4024308260e0 * X(20) ^ 0.3447530873e-2 * X(21) ^ 0.3447530873e-2 * X(22) ^ 0.3447530873e-2 * X(37) ^ 0.3447530873e-2 * X(35) ^ 0.3623683263e-1 * X(180) ^ 0.9412164317e-1 - 0.1202389353e3 * X(32) ^ 0.7319931904e0 * X(12) ^ 0.9410800434e-1 * X(183) ^ 0.9412164317e-1 * X(8) ^ 0.3447530873e-2 * X(18) ^ 0.3447530873e-2 * X(19) ^ 0.3447530873e-2 * X(186) ^ 0.4965524691e0;  % ERG(33,1) = 0.3543221570e4 * X(12) ^ 0.9998550936e0 * X(30) ^ 0.827e0 * X(181) ^ 0.1e1 - 0.8690384668e3 * X(33) ^ 0.385e0 * X(180) ^ 0.1e1;  % ERG(34,1) = 0.4075356854e4 * X(12) ^ 0.9998550936e0 * X(31) ^ 0.827e0 * X(181) ^ 0.1e1 - 0.1623520875e4 * X(34) ^ 0.385e0 * X(180) ^ 0.1e1;  % ERG(35,1) = 0.7296807376e2 * X(12) ^ 0.4999275466e0 * X(32) ^ 0.4134999999e0 * X(183) ^ 0.4999999999e0 * X(40) ^ 0.2499999999e0 - 0.9923162237e1 * X(35) ^ 0.6924999999e0 * X(180) ^ 0.4999999999e0;  % ERG(36,1) = 0.1293842177e0 * X(39) ^ 0.5000000000e0 * X(37) ^ 0.5000000000e0 - 0.1021486386e1 * X(36) ^ 0.1115000000e1 * X(20) ^ 0.2504191618e0 * X(21) ^ 0.3437125747e0 * X(22) ^ 0.2086826348e-1;  % ERG(37,1) = 0.4727668252e0 * X(20) ^ 0.4924017422e0 * X(36) ^ 0.1209280749e1 * X(21) ^ 0.6758455279e0 * X(22) ^ 0.4103347851e-1 * X(8) ^ 0.8422459894e-2 * X(18) ^ 0.8422459894e-2 * X(19) ^ 0.8422459894e-2 * X(32) ^ 0.8422459894e-2 - 0.7306649498e-1 * X(20) ^ 0.8422459894e-2 * X(21) ^ 0.8422459894e-2 * X(22) ^ 0.8422459894e-2 * X(37) ^ 0.9915775401e0;  % ERG(38,1) = 0.8525512814e-2 * X(125) ^ 0.1477628753e0 * X(122) ^ 0.8579203122e0 * X(124) ^ 0.5972136044e0 - 0.9272832387e-2 * X(12) ^ (-0.111e0) * X(38) ^ 0.6314511210e-1 * X(128) ^ 0.5000000001e0 * X(161) ^ 0.7986577182e0 * X(163) ^ 0.1e1;  % ERG(39,1) = 0.8679357180e0 * X(32) ^ 0.5000000000e0 * X(36) ^ 0.5000000000e0 - 0.1227446464e0 * X(39) ^ 0.1000000000e1;  % ERG(40,1) = 0.1659444132e-1 * X(35) - 0.3192737735e0 * X(40) ^ 0.4999999999e0; |
| --- |

**Reference.**

1. MATLAB (2010). 7.10.0.499 ed. Natick, Massachusetts: The MathWorks Inc.
